# A circular RNA vaccine induces durable and cross-protective immunity against *Neisseria meningitidis* serogroup B in mice

**DOI:** 10.1371/journal.ppat.1013741

**Published:** 2026-05-11

**Authors:** Qiqi Zhang, Dongyu Niu, Yusheng Jia, Lirong Lu, Wenting Wu, Changbin Qu, Zeyun Sun, Shixiong Li, Zhining Liu, Jing Lv, Wentao Wang, Jingyou Yu, Long Zhang, Chuwen Li, Wei Peng, Qingqing Zhang, Shuai Wei

**Affiliations:** 1 State Key Laboratory of Respiratory Disease, the First Affiliated Hospital of Guangzhou Medical University, Guangzhou Medical University, Guangzhou, China; 2 Guangzhou National Laboratory, Guangzhou, China; 3 Guangzhou Municipal and Guangdong Provincial Key Laboratory of Molecular Target & Clinical Pharmacology, the NMPA and State Key Laboratory of Respiratory Disease, School of Pharmaceutical Sciences, Guangzhou Medical University, Guangzhou, China‌‌; 4 Graduate School of Guangzhou Medical University, Guangzhou Medical University, Guangzhou, China; 5 Division of Life Sciences and Medicine, University of Science and Technology of China, Hefei, Anhui, China; 6 KingMed School of Laboratory Medicine, Guangzhou Medical University, Guangzhou, China; 7 College of Life Sciences, Nankai University, Tianjin, China; 8 Institute of Pathogenic Biology, School of Basic Medical Sciences, Hengyang Medical School, University of South China, Hengyang, Hunan, China; 9 Institute of Health Inspection and Testing, Hubei Provincial Centre for Disease Control and Prevention (Hubei CDC), Wuhan, China; 10 SCIEX, Beijing, China; INSERM, FRANCE

## Abstract

*Neisseria meningitidis* serogroup B (MenB) remains a major global cause of meningitis and septicemia. However, MenB vaccine development is hindered by antigenic diversity and manufacturing challenges associated with outer membrane vesicles (OMVs) or lipoproteins in resource-limited settings. Here, we engineered a high-purity circular RNA (circRNA) vaccine encoding a bacterial-derived, sequence-optimized fusion antigen of factor H binding protein (fHbp) and Neisserial heparin-binding antigen (NHBA) (fHbp-NHBA), which preserves key bactericidal epitopes in eukaryotic systems. The vaccine elicited potent functional antibody responses by serum bactericidal assay (SBA) using human complement and potent IFN-γ-secreting CD8 + T-cell responses. Crucially, the circRNA vaccine at a low dose provided complete protection and reduced bacteremia against lethal MenB challenge and demonstrated cross protection against a panel of prevalent strains in China. Both single- and two-dose regimens of the circRNA vaccine induced durable immune responses, and the two-dose immunization achieved strain coverage comparable to that of licensed MenB vaccines. Moreover, sera adoptively transferred from the circRNA-immunized group to neonatal rat pups dramatically reduced bacterial loads in blood. This study establishes a foundation for circRNA-based vaccines against bacterial pathogens.

## Author summary

*Neisseria meningitidis* serogroup B (MenB) is a major global cause of life-threatening meningitis and blood poisoning, yet vaccine development remains difficult. MenB strains are highly diverse, and current approaches often rely on outer membrane vesicles or lipoproteins that can be hard to manufacture in low-resource settings. This study describes a high-purity circular RNA (circRNA) vaccine that instructs host cells to make a fusion protein combining factor H binding protein (fHbp) and Neisserial heparin-binding antigen (NHBA), two key MenB surface components. The fusion design preserves important bactericidal sites and enables production in eukaryotic cells. In mice, the circRNA vaccine induced strong functional antibodies able to kill bacteria in the presence of human complement, as well as IFN-γ-secreting CD8^+^ T cells. At a low dose, the vaccine completely protected animals from a lethal MenB challenge and reduced bacteria in the bloodstream. The circRNA vaccine also showed cross-protection against a panel of prevalent MenB strains from China. One- or two-dose schedules generated durable immunity, with two doses achieving strain coverage comparable to licensed MenB vaccines. Antibodies transferred from vaccinated animals also protected newborn rats, supporting circRNA as a promising platform for bacterial vaccines.

## Introduction

*Neisseria meningitidis* serogroup B (MenB), one of the dominant disease-causing invasive meningococcal serogroups (A, B, C, W, X, and Y) [1,2], remains a formidable global health threat. In high-income countries and regions, MenB accounts for up to 90% of meningococcal disease in children under five [3]. Early diagnosis is often difficult to achieve, and immunization remains the only convenient and effective approach to reducing the disease burden. The development of polysaccharide-based and polysaccharide-protein conjugate vaccines against MenB is precluded due to structural similarity between the MenB capsular polysaccharide and polysialylated proteins on human neural cells [4,5]. Antigens were discovered by reverse vaccinology [6], including GNA2132 (also known as NHBA) [7], GNA1870 (fHbp) [8], neisserial adhesin A (NadA), GNA1030, and GNA2091, paving the way for developing of recombinant protein-based vaccines. Nowadays, there are only two commercial authorized recombinant protein vaccines available. 4CMenB (marketed as Bexsero, developed by GlaxoSmithKline) is a multicomponent vaccine containing two recombinant fusion proteins (NHBA-GNA1030 and fHbp-GNA2091), NadA, and detergent-extracted outer membrane vesicles (OMVs) derived from the *Neisseria meningitidis* NZ98/254 strain isolated during the New Zealand epidemic [9–11]. Recombinant lipoprotein-based vaccine Trumenba (Pfizer) contains two fHbp variants, V1.55 (subfamily B01) and V3.45 (subfamily A05), to confer broad protection against diverse *Neisseria meningitidis* strains [12,13]. Although these two licensed vaccines have demonstrated substantial effectiveness in reducing the incidence of MenB disease [9,14,15], the protective ability for prevalent strains in China warrants further investigation [13,16,17]. Worldwide strain divergence and the complexity of manufacturing OMVs or lipoproteins persist as obstacles, especially in resource-limited settings. These challenges underscore the need for next-generation vaccine platforms with improved coverage, stability, and simplified composition [18–20].

Circular RNA (circRNA) is a type of covalently closed single-stranded RNA molecule primarily generated through pre-mRNA back-splicing [21,22]. Its lack of free termini confers resistance to exonuclease degradation both *in vivo* and *in vitro*. Compared with linear mRNA counterparts, circular RNA exhibits significantly extended half-lives in mammalian cells [23]. Most circRNAs are classified as non-coding RNA due to their lack of intact open reading frames (ORFs) and absence of ribosome association [24]. Only a small subset containing embedded internal ribosome entry site (IRES) or m6A modification demonstrates translation capability [25–28]. Wesselhoeft *et al.* pioneered *in vitro* synthesis of circRNA using a permuted intron-exon (PIE) splicing strategy, demonstrating their therapeutic potential through protein translation capabilities [29,30].

Divergent translation mechanisms and post-translational modifications (PTMs) pathways between prokaryotic and eukaryotic systems hinder the application of circRNA modality for expressing bacterial-derived proteins or antigens. Typically, the glycosylation of bacterial antigens can alter epitope conformation, potentially compromising immunogenicity [31–33].

Here, we pioneer the application of a single circRNA molecule encapsulated into lipid-nanoparticle (LNP) to express a bacterial-derived fHbp-NHBA fusion antigen while preserving critical epitopes. Through integrated size exclusion chromatography (SEC) and RNase R digestion, high-purity circRNA with minimal nicked RNA contamination was obtained. We immunized mice with the candidate circRNA vaccine and systematically evaluated the resulting humoral and cellular immune responses, as well as its ability to confer cross-protection against prevalent MenB strains in China. This work provides new insights that could inform the development of circRNA vaccines targeting bacterial pathogens.

## Results

### The circRNA robustly expressed bacterial-derived fHbp-NHBA fusion antigen without compromising critical epitopes

**Fig 1:**
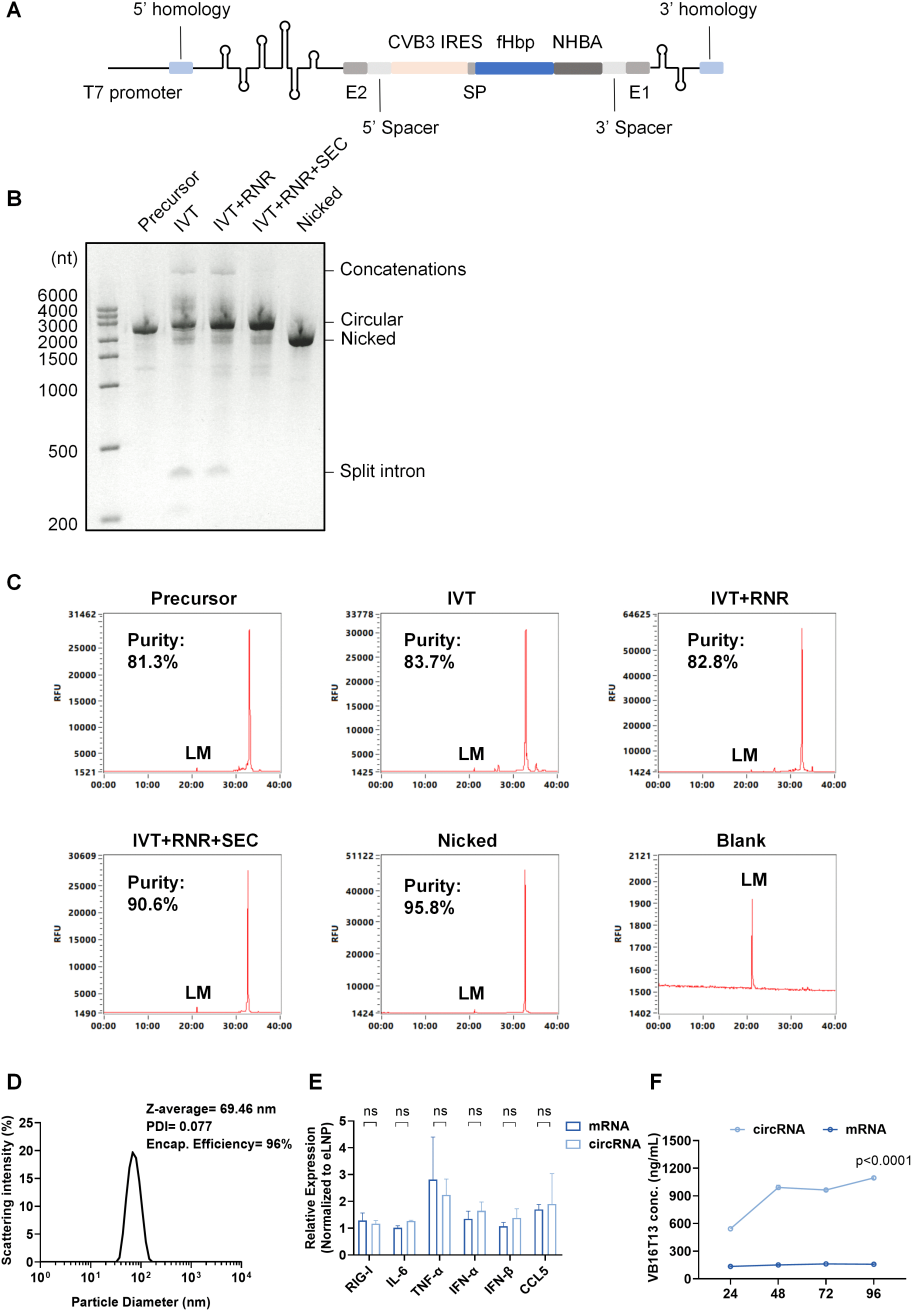
circVB16T13 robustly expresses the fHbp-NHBA fusion antigen. **(A)** Schematic of the circVB16T13 construct. SP: signal peptide derived from the immunoglobulin κ light chain variable region. **(B)** E-gel EX analysis of RNA samples collected at different purification stages. Precursor: linear IVT product; IVT: *in vitro* transcription (circularization was concurrently performed during IVT); RNR: RNase R-treated sample; SEC: size-exclusion chromatography fraction; Nicked: linearized RNA control with the same nucleotide sequence as circVB16T13. **(C)** Quantification of RNA integrity by capillary electrophoresis (CE) at each purification step. RNA purity was calculated as the percentage of the main peak area relative to the total peak area. **(D)** Characterization of LNP-encapsulated circVB16T13. Representative particle size distribution measured by dynamic light scattering (DLS). Encapsulation efficiency annotated in upper right corner was quantified using RiboGreen assay. **(E)** Relative expression of innate immune genes (*RIG-I*, *IL-6*, *TNF-α, IFN-α, IFN-β*, and *CCL5*) in circVB16T13-transfected cells compared with N1-methylpseudouridine-modified mRNA controls, measured by qRT-PCR. **(F)** Persistence of antigen expression mediated by circVB16T13 versus N1-methylpseudouridine-modified mRNA, analyzed by ELISA. Cells were transfected with 1 μg circRNA or modified mRNA.

Reverse vaccinology identified five candidate antigens that exhibited bactericidal activity or conferred protection in passive immunization assays [6]. Based on these findings, we rationally engineered fHbp and NHBA as a fusion protein (termed VB16T13) to enhance their immunogenicity. This fusion protein sequence was inserted into a classical group I intron autocatalysis framework to express VB16T13 in mammalian cells (circVB16T13) (Fig 1A). Sanger sequencing and RNase H digestion verified the integrity of circVB16T13 (S1A-S1B Fig). Residual contaminants in circRNA formulations trigger unintended immunogenic responses, consequently suppressing protein translation efficiency. To minimize the side effects, we used SEC to remove the impurities which typically derived from hydrolysis or endonucleolytic cleavage in reaction process (S1C Fig). To further enhance purity, linear RNA byproducts were digested with RNase R prior to SEC purification (Fig 1B). The efficiency of purification at each step was quantified by capillary electrophoresis (CE) and capillary gel electrophoresis with laser-induced fluorescence detection (CGE-LIF) [34] (Figs 1C and S1D). Results demonstrated that RNase R pretreatment coupled with SEC effectively purified circVB16T13 with minimal residual contaminants (90.7% by CE and 71.1% by CGE-LIF) (Figs 1C and S1D). Notably, while CE fails to resolve circRNA from nicked RNA, CGE-LIF achieved high-resolution separation of these species [34] (Figs 1C and S1D). Next, circVB16T13 was encapsulated into LNP (Fig 1D) and delivered into A549 cells to verify the innate immune responses. As expected, the innate immune responses elicited by circVB16T13 were comparable to those by 1mΨ-modified mRNA (Fig 1E).

The LinearDesign algorithm [35] was employed to optimize the VB16T13 sequence, enhancing translational efficiency and stability by balancing minimal free energy (MFE), codon adapt index (CAI), and GC content (S2A-B Fig). To evaluate whether codon optimization enhances protein expression in human cells, HEK293T cells were transfected with circRNA encoding VB16T13 either before or after optimization by LinearDesign. The concentration of secreted VB16T13 protein in the culture supernatant was quantified by ELISA using the 1A12 monoclonal antibody. LinearDesign optimization markedly increased protein expression levels, reaching 6.4-fold higher concentrations compared to the non-optimized construct (S2C-D Fig). Compared to linear mRNA counterparts, this high-purity LinearDesign-optimized circVB16T13 exhibited robust protein expression in HEK293T cells, sustaining VB16T13 secretion for at least 96 hours (Figs 1F and S2D). Resuspending circVB16T13 in nuclease-free water and storing at 4°C exhibited uncompromised structural stability and translational efficacy (95.7% of maximal expression) over 30 days (S1E-S1F Fig). In contrast, 37°C storage induced complete degradation at 14 days (S1E-S1F Fig).

**Fig 2:**
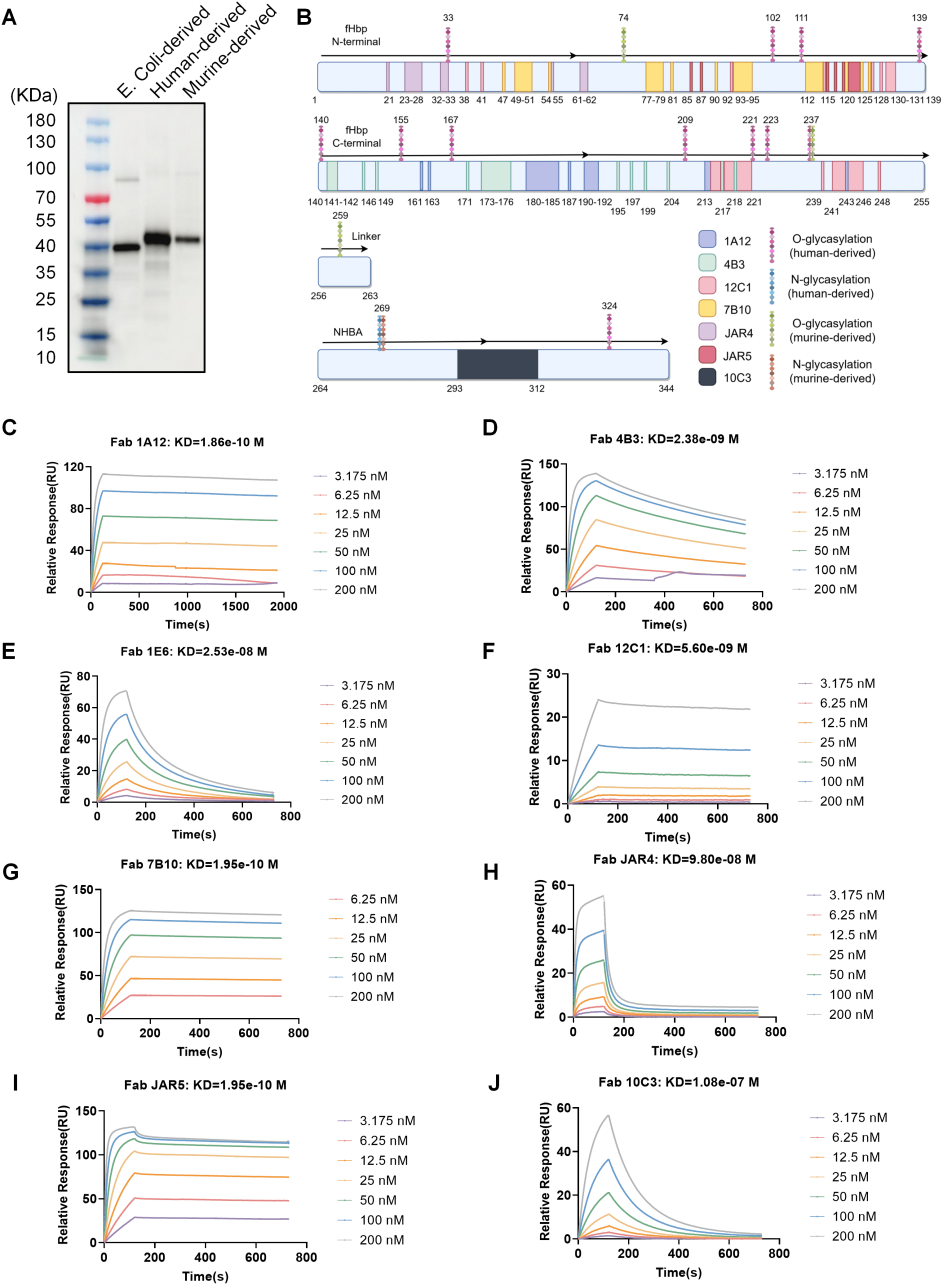
Eukaryotically expressed VB16T13 retains conformational epitopes. **(A)** Immunoblot analysis comparing molecular weights of *E. coli*-derived, human-derived (Expi293F cells), and murine-derived (NIH 3T3 cells) VB16T13. **(B)** Schematic diagram of the binding residues for the anti-fHbp Fabs (1A12, 4B3, 1E6, 12C1, 7B10, JAR4, and JAR5) and the anti-NHBA Fab (10C3), together with the glycosylation sites identified in human- and murine-derived VB16T13 by LC-MS/MS. Epitope annotations were compiled from published studies [36–44] and plotted schematically in this figure. This figure was created using Figdraw (www.figdraw.com) with permission. **(C-J)** Surface plasmon resonance (SPR) binding kinetics of human-derived VB16T13 with indicated Fabs. The equilibrium dissociation constants (KD) for VB16T13-Fab interactions are shown in the panels.

Given the divergence in translation and post-translational modification mechanisms between prokaryotic and eukaryotic systems, we systematically mapped key conformational epitopes in VB16T13 using eight well-characterized neutralizing fragment antigen-binding (Fab) antibodies (1A12, 4B3, 1E6, 12C1, 7B10, JAR4, JAR5, and 10C3) [36–44] targeting fHbp or NHBA domains. Previous studies have comprehensively defined the MenB fHbp epitopes recognized by these Fabs, mapping these epitopes is expected to largely account for the overall immunogenicity of the target antigen (Fig 2B). Because only the complex structure of 1E6 bound to fHbp variant 3 has been reported to date, whereas the fHbp used in our study is variant 1.1, the VB16T13 residues involved in 1E6 binding are not indicated. The approximately 5-kDa higher molecular weight of human-derived VB16T13 (expressed in Expi293F cells) and murine-derived VB16T13 (expressed in NIH 3T3 cells), compared with bacteria-derived VB16T13 (expressed in *E. coli*), indicated the presence of eukaryotic-specific modifications (Fig 2A). To identify these modifications, we performed liquid chromatography-mass spectrometry (LC-MS/MS) on the eukaryotically expressed antigens, namely human-derived VB16T13 and murine-derived VB16T13. The analysis revealed multiple N- and O-glycosylation sites distributed across the protein. In human-derived VB16T13, twelve O-glycosylation sites (S33, S102, T111, T139, S140, T155, T167, S209, S221, S223, S237, and S324) and one N-glycosylation site (N269) were identified. In murine-derived VB16T13, three O-glycosylation sites (S74, S237, and S259) and one N-glycosylation site (N269) were identified. Notably, S237 and N269 were observed in antigens from both cell lines. No glycosylation was detected in the *E. coli*-derived VB16T13 (Fig 2B). To further clarify the impact of glycosylation on epitope integrity, we quantified the binding affinities between *E. coli*-derived, human-derived, and murine-derived VB16T13 and the eight Fabs using surface plasmon resonance (SPR). All eight Fabs bound the three VB16T13 preparations with high affinity (Table 1 and Fig 2C-J). The affinities were highly similar across antigens from the three expression hosts. Notably, although O-glycosylation was observed at the JAR4 epitope residue S33 and the 12C1 epitope residue S221 in human-derived VB16T13, no reduction in binding affinity was detected in the SPR assays (Fig 2B, 2F and 2H and Table 1). Taken together, these results demonstrated that glycosylation of eukaryotically expressed VB16T13 did not mask the critical epitopes.

**Table 1:** *E. coli*-derived, human-derived, and murine-derived VB16T13 bind the indicated Fabs with comparable affinities. Binding of VB16T13 to the indicated fragment antigen-binding (Fab) molecules was measured by surface plasmon resonance (SPR), and affinities are reported as equilibrium dissociation constants (KD, M).

Fab	*E.coli*-derived	Human-derived	Murine-derived
1A12	5.00e-11	1.86e-10	7.10e-11
4B3	2.19e-09	2.38e-09	2.33e-09
1E6	2.24e-08	2.53e-08	2.35e-08
12C1	5.13e-09	5.60e-09	4.98e-09
7B10	3.66e-10	1.95e-10	1.88e-10
JAR4	8.53e-08	9.80e-08	1.06e-07
JAR5	1.71e-10	1.95e-10	1.59e-10
10C3	7.63e-08	1.08e-07	1.14e-07

### CircVB16T13 induced robust immune responses in mice

**Fig 3:**
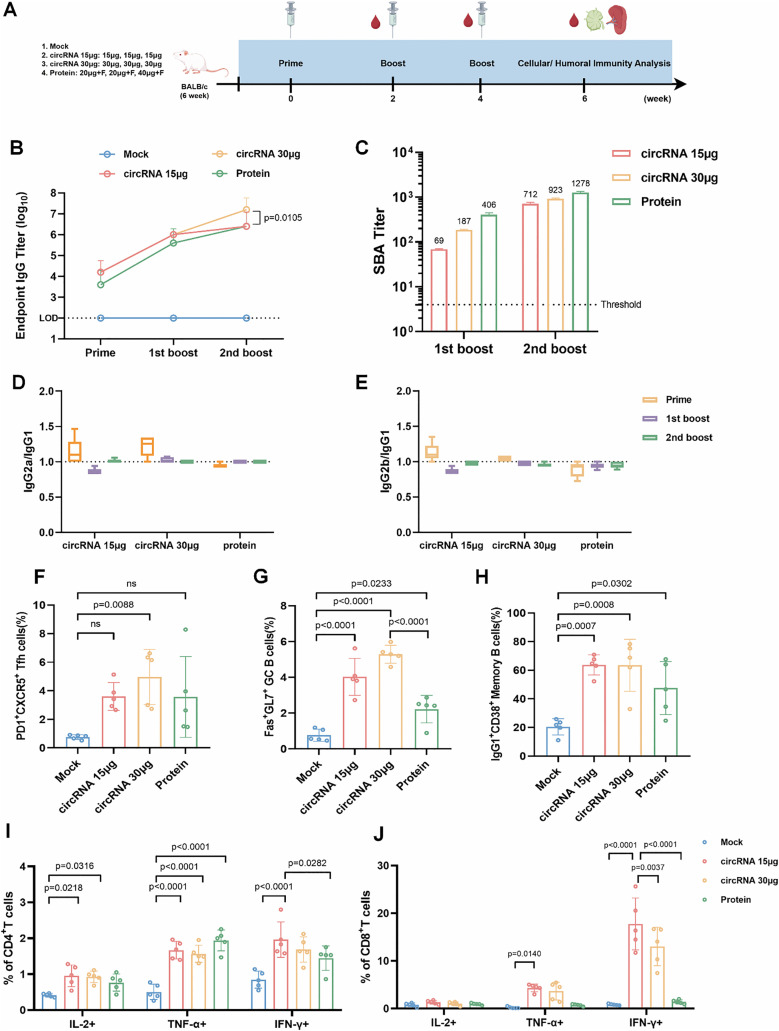
circVB16T13 elicits potent humoral and cellular immune responses in mice. **(A)** Immunization scheme. BALB/c mice (n = 5 per group) received three intramuscular or subcutaneous injections of circVB16T13 or recombinant VB16T13 protein emulsified with Freund’s adjuvant at 2-week intervals. Sera, spleens, and inguinal lymph nodes (ILNs) were harvested at indicated timepoints for immune analyses. This figure was created using Figdraw (www.figdraw.com) with permission. **(B)** Antigen-specific serum IgG endpoint titers measured 2 weeks after the prime, first boost, and second boost immunizations. Data are log10-transformed. Bars represent geometric mean titer (GMT) with 95% CI. The dashed line indicates the limit of detection. **(C)** Human serum bactericidal activity (hSBA) titers in pooled serum against *N. meningitidis* strain MC58 at 2 weeks after each immunization. The pooled serum samples were assayed in technical replicates (n = 3). Bars indicate the GMT and error bars indicate geometric SD of technical replicates. The dashed line denotes the protective threshold (hSBA = 4). **(D, E)** Serum IgG subclass ratios: IgG2a/IgG1(**D**), IgG2b/IgG1(**E**). Horizontal lines denote mean values. **(F-H)** Flow cytometry analysis of ILN immune subsets 2 weeks after the second boost. **(F)** Tfh cells (CD4^+^CD44^+^PD-1^+^CXCR5^+^). **(G)** Germinal center B cells (CD45^+^CD45R^+^GL7^+^Fas^+^). **(H)** Memory B cells (CD45^+^CD45R^+^IgG1^+^CD38^+^). **(I, J)** Cytokine production by splenic T cells collected 2 weeks after the second boost. Frequencies of CD4^+^ T cells (**I**) and CD8^+^ T cells (**J**) secreting IL-2, TNF-α, and IFN-γ after 24-hour stimulation with VB16T13 protein, measured by intracellular cytokine staining (ICS). Comparisons among more than two groups were conducted by one-way ANOVA **(F-H)** and Two-way ANOVA comparison tests **(B, I, J)**.

Immunogenicity of the candidate circRNA vaccine (circVB16T13) was evaluated in 6-week-old female BALB/c mice (n = 5/group) following the prime-boost-boost regimen (Fig 3A). In the preliminary study, we compared human serum bactericidal activity (hSBA) titers and antigen-specific IgG subclass titers elicited by the VB16T13 protein vaccine formulated with different adjuvants or doses in BALB/c mice after three immunizations, using the 4CMenB-immunized group as a comparator (S4A-B Fig). The alum-adjuvanted group induced an hSBA titer comparable to that of 4CMenB (184 versus 222). In contrast, at the same antigen dose as the alum formulation, Freund’s adjuvant induced a higher hSBA titer (701), and increasing the third-dose antigen amount further boosted the hSBA titer to 1278. Therefore, to effectively enhance immunogenicity for the protein antigen, Freund’s adjuvant was selected for the VB16T13 protein positive control group, as it is among the most potent adjuvants in mice. The antigen doses for circRNA vaccination were chosen with reference to previous studies of MenB protein vaccines, which commonly used 20 µg of protein per dose, to ensure comparability of antigen exposure between formulations [6,14,45]. The Protein group was immunized subcutaneously with recombinant VB16T13 protein, expressed in *E. coli* and purified to remove endotoxin (S4 Table), emulsified in Freund’s adjuvant (complete for priming, incomplete for boosts). For circVB16T13, LNP-encapsulated formulations were administered intramuscularly at doses of 15 μg (low-dose) and 30 μg (high-dose) per immunization (Fig 3A). Seroconversion was observed after the prime immunization in all three immunized groups (Fig 3B). After the second boost, the high-dose circVB16T13 group showed higher GMTs (1.58 × 10^7^) than the protein group (2.51 × 10^6^) and low-dose group (2.51 × 10^6^). All immunized groups elicited functional antibody responses, as demonstrated by the hSBA (Fig 3C). The protein group exhibited significantly higher hSBA titers than those of the circVB16T13-immunized groups at both the first and second boosts (Fig 3C), likely due to the strong adjuvant effect of Freund’s adjuvant. Therefore, in the subsequent animal challenge experiment, the amount of VB16T13 protein was reduced to 20 μg at the second boost. Bactericidal efficiency was assessed through IgG-mediated complement activity. However, IgG subclass composition and complement-fixation capacity can differ in mice. Therefore, IgG subclass titers were measured in all immunized groups. The results showed that IgG1, IgG2a, IgG2b, and IgG3 were all detected in the vaccine groups, and the titers of these subclasses significantly increased after each boost (S5A-S5D Fig). Notably, between the prime and the second boost, GMTs of IgG3 titers increased by 126-fold and 455-fold in the circRNA low-dose and high-dose groups, respectively, whereas only a 12-fold increase was observed in the protein group. Next, we analyzed the IgG2a/IgG1 and IgG2b/IgG1 ratios. In all immunized groups, both ratios were approximately 1 (Fig 3D-E), indicating a balanced Th1/Th2 immune response that reflects coordinated cellular and humoral immunity without significant polarization.

Given the critical role of germinal centers (GC) in antibody affinity maturation, inguinal lymph nodes (ILNs) were collected to quantify GC B-cell (CD45^+^CD45R^+^GL7^+^Fas^+^) and follicular helper T-cell (Tfh; CD4^+^CD44^+^PD1^+^CXCR5^+^) responses (S6A-B Fig). Two weeks after the third immunization, both Tfh and GC B-cell frequencies were significantly elevated in all immunized groups compared with controls. No significant difference in Tfh cell frequencies was observed between circRNA and protein vaccine groups (Fig 3F). In contrast, GC B-cell frequencies were markedly increased, with the high-dose circRNA group exhibiting a significantly higher ratio than the protein group (p < 0.0001) (Fig 3G). To evaluate the durability of the B-cell response, memory B cells (CD45^+^CD45R^+^IgG1^+^CD38^+^) in ILNs were analyzed (S6A Fig). Memory B cell frequencies were dramatically increased after three-dose immunization in both circRNA and protein vaccine groups (Fig 3H).

Although an hSBA titer ≥4 is widely accepted as a serological correlate of vaccine-mediated protection, this assay is limited to evaluating MenB vaccine immunogenicity against a small number of indicator strains. Given this constraint, we further examined the contribution of circRNA vaccine-induced cellular immunity to MenB protection. To analyze CD4⁺ and CD8 ⁺ T-cell immune responses, splenocytes from immunized mice were harvested and stimulated with VB16T13 antigens for 24 hours. The frequencies of IL-2 ⁺ , TNF-α ⁺ , and IFN-γ ⁺ CD4⁺ and CD8 ⁺ T cells were quantified by flow cytometry (S6C Fig). Compared with the untreated group, both circRNA and protein vaccine significantly increased IL-2 ⁺ , TNF-α ⁺ , and IFN-γ ⁺ CD4 ⁺ T cells (Fig 3I). Notably, elevated TNF-α⁺ and IFN-γ ⁺ CD8 ⁺ T cell responses were observed exclusively in circRNA vaccine groups, with IFN-γ ⁺ CD8 ⁺ T-cell frequencies showing 21-fold and 15.4-fold increases in the low- and high-dose circRNA groups, respectively, relative to untreated and protein vaccine groups (Fig 3J). Collectively, these data demonstrate that circVB16T13 elicited both functional antibody and T-cell-mediated immune responses.

### The low-dose circVB16T13 vaccine provided full protection against a lethal MenB infection in mice

**Fig 4:**
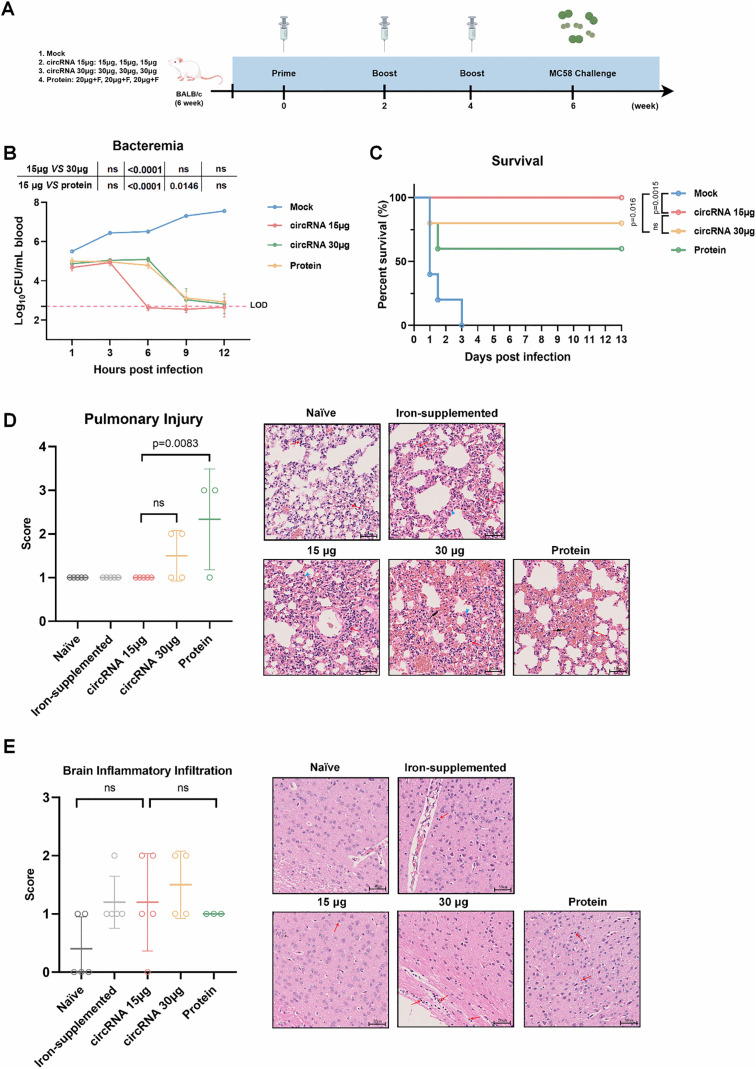
circVB16T13 confers protection against meningococcal challenge. **(A)** Experimental design for *N. meningitidis* serogroup B strain MC58 challenge. BALB/c mice (n = 5 per group) were infected intraperitoneally with 2 × 10^6^ CFU at 14 days after the final immunization. This figure was created using Figdraw (www.figdraw.com) with permission. **(B)** Kinetics of bacteremia in pooled whole blood following challenge. Bacterial loads were quantified at 1, 3, 6, 9, and 12 hours post infection. The dashed line indicates the limit of detection (LOD = 500 CFU/mL). Values under the LOD were assigned a value of 250 CFU/mL (LOD/2) for statistical analysis. **(C)** Survival curves of mice monitored for 13 days post infection. **(D, E)** Histopathological analysis of tissues at 13 days post infection. Naïve and iron-supplemented groups were uninfected and served as baseline controls. **(D)** Representative H&E-stained lung sections. Black arrows indicate hemorrhage, red arrows indicate inflammatory cells, and blue arrows indicate septal thickening. **(E)** Representative H&E-stained brain sections. Red arrows indicate inflammatory cells. Scale bars, 50 μm. Comparisons among more than two groups were conducted by one-way ANOVA comparison tests **(D,** **E)**. Comparisons of the survival data were conducted by Log-rank (Mantel-Cox) tests **(C)**.

To systematically evaluate the protective efficacy of circRNA vaccine, we established a murine infection model using a lethal challenge with the fHbp V1.1-expressing *N. meningitidis* strain MC58. Optimization of iron supplementation and bacterial inoculum was prioritized as key parameters, following a three-step procedure (S7A Fig): (1) Iron dextran (2.5-15 mg/mouse, i.m.) was administered 16 hours prior to MC58 challenge. Time-course bacteremia profiling and survival analysis identified 10 mg/mouse as optimal for subsequent experiments (S7B-C Fig); (2) The safety of 10 mg iron dextran per mouse was verified by monitoring body temperature and body weight (S7D-E Fig); (3) Bacterial challenge doses (2 × 10⁷-2 × 10⁵ CFU) were systematically evaluated, and 2 × 10⁶ CFU was selected as the minimum lethal dose used for challenge modeling (S7F-G Fig). Next, BALB/c mice were immunized with high- and low-dose circVB16T13 and recombinant protein vaccine and evaluated protection efficacy using the established challenging model (Fig 4A). Unimmunized controls developed progressive bacteremia, whereas vaccinated cohorts achieved rapid pathogen clearance, with blood bacterial loads falling below the detection threshold within 12 hours post infection (hpi), demonstrating vaccine-mediated protection. The low-dose circVB16T13 group showed 100% survival following lethal MC58 challenge, maintaining normal clinical appearance (including fur condition and mobility) indistinguishable from naïve animals. Mortality occurred in the comparator groups: 20% (1/5) in high-dose circRNA group and 40% (2/5) in protein group succumbed within 48 hpi (Fig 4C). Survivors were euthanized at day 13 for histopathological analysis of major organs (hearts, livers, spleens, lungs, kidneys, and brains). Histopathological analysis (H&E staining) revealed that both 15-μg and 30-μg circVB16T13 regimens conferred near-complete organ protection, except for mild splenic alterations (Fig 4D-E and S8A-S8D). In stark contrast, the protein vaccine groups exhibited disrupted tissue integrity, characterized by multifocal inflammatory cell infiltration in the lung post-challenge (Fig 4D-E and S8A-S8D). Whether the lesions and inflammatory cell infiltration in the protein-immunized group were caused by Freund’s adjuvant still needs further investigation.

In addition, to assess whether T-cell immunity contributes to protection during infection, we depleted CD8 + T cells in mice immunized three times with 15 μg circRNA by intraperitoneal administration of anti-CD8α antibodies prior to lethal challenge (S9A Fig). However, no significant differences in bacteremia or survival were observed between the CD8^+^ T-cell-depleted group and the isotype control group (S9B-C Fig). These results suggest that functional antibodies play a major role in protection against MenB infection in this model, while potential protective mechanisms of cellular immunity warrant further investigation.

### CircVB16T13 confers cross protection against prevalent strains in China

Both low-dose and high-dose circVB16T13 vaccines conferred protection against the indicator strain MC58. Subsequently, we analyzed potential cross protection against prevalent strains in China. Eighteen strains were collected, and their corresponding characteristics—including fHbp and NHBA genotypes, sequence types (ST), and clonal complexes (CC)—were analyzed. Pooled serum samples from three-dose circVB16T13-immunized groups were systematically evaluated for protective activity against these 18 strains (Table 2); pooled serum from the mock group was included as a control (Table 2). The annotated monoclonal antibodies (mAbs) 4B3 and 1E6 were tested as positive controls (S5 Table). Because the fHbp variant 1.1 in VB16T13 originates from MC58, 5 out of 6 fHbp var 1 strains (excluding MC58) showed protective activity with hSBA titers ≥4. Notably, 3 out of 10 fHbp var 2 strains elicited protective responses (hSBA), demonstrating cross protection. However, sera showed no bactericidal activity against fHbp var 3 strains. To further compare the bactericidal activity of the circRNA vaccine with that of licensed vaccines in a CD-1 mouse model, the vaccine candidate’s capacity to induce protection against 7 strains expressing different fHbp variants was assessed by hSBA using pooled sera collected after administration of two-dose circVB16T13 vaccine and licensed vaccines, 4CMenB and rLP2086 (Table 3); pooled serum from the mock group was included as a control (Table 3). Both the two-dose circVB16T13 and 4CMenB induced hSBA responses against fHbp 2.18 and 2.22 strains, even though 4CMenB contains multiple antigens whereas circVB16T13 comprises only fHbp and NHBA from the MC58 strain, highlighting the potential of the circRNA platform to elicit cross-protective immunity with simplified compositions. Both circVB16T13 and 4CMenB elicited hSBA responses against strains expressing fHbp variants 1.1, 1.276, and 1.5, but not against 1.1352. In contrast, two-dose rLP2086 showed no positive hSBA titers against the tested strains. After the third immunization, only low hSBA titers were observed against strains expressing fHbp variants 1.276, 1.5, 2.18, 2.22, and 3.1239, which may be due to the small number of strains assessed in this panel (Table 3). Overall, the breadth of strain coverage achieved by the two-dose circVB16T13 vaccination was comparable to that induced by 4CMenB and rLP2086 within this strain panel.

**Table 2:** circVB16T13 exhibits bactericidal activity against prevalent MenB strains in China. Pooled serum from BALB/c mice immunized with three doses of circVB16T13 were evaluated for bactericidal activity against 19 *N. meningitidis* serogroup B strains; pooled serum from the mock group was included as a control. Data are presented as GMTs calculated from two or three technical replicates. The corresponding fHbp and NHBA genotypes, sequence types (ST), and clonal complexes (CC) were determined by collaborators and are listed below. N/A: not available; UA: unassigned.

Target strain	fHbp variant	NHBA variant	Sequence Type	Clonal Complex	Mock SBA titer	circVB16T13 SBA titer
MC58	1.1	3	N/A	N/A	<4	923
SG-ST01–099	1.13	1236	ST-8913	UA	<4	29
SG-ST01–443	1.1352	688	ST-10052	UA	<4	<4
SG-ST01–527	1.276	N/A	N/A	N/A	25	160
SG-ST01–304	1.42	503	ST-4821	cc4821	<4	48
SG-ST01–420	1.5	9	ST-10051	cc4821	82	349
SG-ST01–343	1.89	688	ST-5664	cc4821	557	835
SG-ST01–105	2.16	73	ST-12786	UA	<4	<4
SG-ST01–201	2.16	669	ST-12316	cc4821	<4	<4
SG-ST01–075	2.16	910	ST-5664	cc4821	<4	<4
SG-ST01–101	2.18	20	ST-658	cc11	<4	<4
SG-ST01–202	2.18	1629	ST-12344	UA	9	414
SG-ST01–103	2.19	1238	ST-8928	UA	<4	10
SG-ST01–100	2.21	1237	ST-8916	UA	<4	<4
SG-ST01–119	2.22	1101	ST-8791	UA	94	304
SG-ST01–162	2.405	357	ST-10746	UA	<4	<4
SG-ST01–158	2.423	1086	ST-6934	UA	<4	<4
SG-ST01–401	3.1239	669	ST-10607	cc4821	<4	<4
SG-ST01–440	3.45	18	ST-213	cc213	<4	<4

**Table 3:** Serum bactericidal activity of circVB16T13 is comparable to that of licensed MenB vaccines. hSBA titers of pooled serum from CD-1 mice immunized with two doses of circVB16T13 (collected 2 weeks after dose 2), 4CMenB (collected 1 week after dose 2), or rLP2086 (collected 1 week after dose 2 and 6 weeks after dose 3). Pooled serum from the mock group was included as a control. Data are presented as GMTs calculated from three technical replicates. N/A, not available.

Target strain	fHbp variant	NHBA variant	Mock SBA titer	circVB16T13 2 weeks after dose 2	4CMenB 1 week after dose 2	rLP2086 1 week after dose 2	rLP2086 6 weeks after dose 3
MC58	1.1	3	<4	631	404	<4	<4
SG-ST01–443	1.1352	688	<4	<4	<4	<4	<4
SG-ST01–527	1.276	N/A	<4	122	386	<4	60
SG-ST01–420	1.5	9	55	159	426	15	186
SG-ST01–202	2.18	1629	<4	65	106	<4	20
SG-ST01–119	2.22	1101	12	79	402	5	108
SG-ST01–401	3.1239	669	<4	<4	6	<4	19

### The circVB16T13 vaccine effectively confers protection to individuals

**Fig 5:**
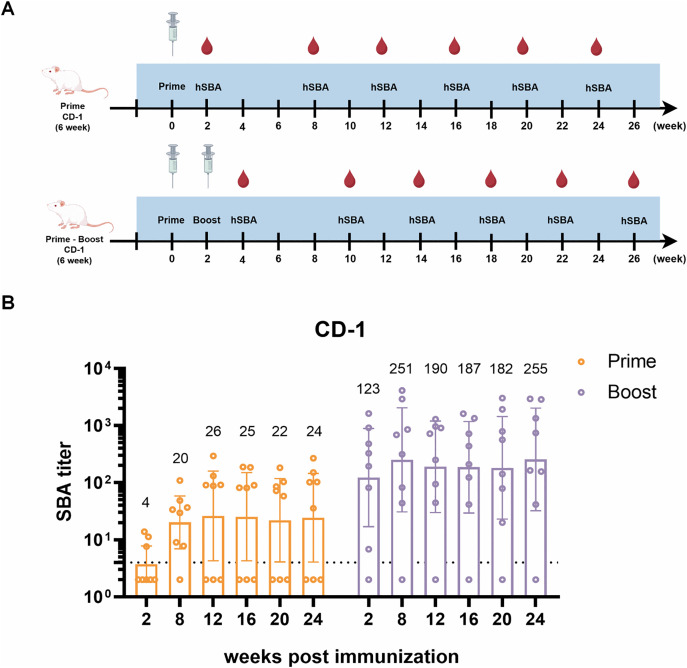
circVB16T13 confers protection to individuals. **(A)** Immunization scheme in outbred CD-1 mice (n = 8 per group) receiving 15 μg circVB16T13. This figure was created using Figdraw (www.figdraw.com) with permission. **(B)** Individual sera from mice immunized with either a single dose or a prime-boost regimen were analyzed for serum bactericidal activity at the indicated time points. Titers are presented as GMTs with 95% CIs. Values below the LOD (titer <4) were assigned a value of 2 (LOD/2) for calculation and statistical analysis.

To monitor the longevity of vaccine-induced antibodies and determine whether circVB16T13 provides inter-individual protection in outbred CD-1 mice, groups of mice (n = 8) were immunized with either a single dose or two doses of 15 μg circVB16T13. hSBA titers against MC58 strain were measured at indicated time points after immunization (Fig 5A). In the single-dose group, only 3 mice exhibited hSBA titers ≥4 at week 2. By week 24 post- prime, most mice achieved hSBA titers ≥4, with a GMT of 24. In the two-dose group, 7 mice showed SBA titers ≥4 (GMT = 123) 2 weeks after boost. At the final time point, only 1 mouse had SBA titer <4 (GMT = 255) (Fig 5B). These results demonstrate that circVB16T13 elicits functional antibody responses in 7 out of 8 immunized mice and induces durable immune response for at least 6 months. However, whether a single-dose regimen is sufficient for effective protection warrants further investigation.

### Sera from circVB16T13-immunized mice conferred effective protection in neonatal rat pups

To further evaluate vaccine efficacy, sera from low-dose circVB16T13-immunized mice and mice immunized with 20 μg VB16T13 formulated with Freund’s adjuvant were adoptively transferred via subcutaneous injection into 7-day-old Wistar rat pups (n = 5/group). The pups were then challenged with 1 × 10⁶ CFU of *N. meningitidis* strain MC58. Blood samples were collected 3 hours post challenge, and bacterial loads were quantified in four independent replicates (Table 4). High bacterial burdens were observed in the control group receiving non-immune sera. In contrast, no bacteria were detected in any replicate of the circVB16T13-sera-treated pups, whereas MC58 was detected in only one of four replicates in the protein vaccine group. These results demonstrate that protective components in the sera of circVB16T13-immunized mice (e.g., functional antibodies) confer robust protection in neonatal rats, highlighting the potential of circRNA vaccines for maternal-neonatal immunization against MenB.

**Table 4:** Sera from circVB16T13-immunized mice confer effective protection in neonatal rat pups. Pooled sera from the mock group (hSBA < 4), the low-dose circVB16T13 group (15 μg, three immunizations; hSBA 646), and the protein-immunized group (20 μg VB16T13 with Freund’s adjuvant, three immunizations; hSBA 701) were passively transferred to Wistar rat pups. Blood bacteria loads were measured 3 hours after *N. meningitidis* strain MC58 challenging. U/D: undetectable.

Strain	Challenged CFU	Treatment	Sera dilution	CFU/mL (3h)			
				Rep.1	Rep.2	Rep.3	Rep.4
MC58	1 × 10^6^	Mock mouse serum	1:10	500	1000	1000	500
		Anti-circRNA mouse serum		U/D	U/D	U/D	U/D
		Anti-Protein mouse serum		500	U/D	U/D	U/D

## Discussion

mRNA vaccines have played a pivotal role in combating SARS-CoV-2 owing to their rapid development and robust immunogenicity [46,47]. However, several challenges, such as limited stability, restrict their broader application in resource-limited settings [48]. As the next generation of mRNA technology, circular RNA (circRNA) offers distinct advantages in molecular stability and sustained protein expression [49]. Although circRNA vaccines have been evaluated preclinically against viral pathogens such as SARS-CoV-2 and Zika virus [50–52], their therapeutic and prophylactic use still faces key challenges, such as purification [53]. Impurities within circRNA preparations can activate innate immune sensors, thereby suppressing translation efficiency. For instance, nicked RNAs generated through non-specific hydrolysis of circRNAs can activate RNA sensors (e.g., Toll-like receptors TLR3, TLR7/8, PKR and OAS), triggering pro-inflammatory pathways [54]. While size exclusion chromatography (SEC) is widely employed for circRNA purification, its limited resolution impedes efficient separation of circRNAs from nicked RNAs, particularly for high-molecular-weight species. Combining RNase R digestion with SEC markedly improved the purity of circRNA preparations, increasing from 41.83% to 71.1% as quantified by CGE-LIF (S1C Fig). Both CE and CGE-LIF confirmed the improved integrity and purity. Unexpectedly, CE–typically regarded as the gold-standard method for circRNA analysis [55]–failed to distinguish LinearDesign-optimized circRNAs from nicked RNA contaminants, whereas CGE-LIF achieved high-resolution separation of these species (Figs 1C and S1D). Collectively, these findings highlight the need for continued development of advanced purification techniques to improve circRNA quality for therapeutic and prophylactic applications.

Glycosylation in eukaryotic systems must be carefully considered when expressing bacterial-derived antigens in mammalian cells. The addition of host glycans can shield critical epitopes and impede lymphocyte recognition, as these glycans are known to possess immunoregulatory properties [56]. For example, when the *Mycobacterium tuberculosis* surface protein Ag85A was expressed in mammalian cells, multiple glycans—including GalNAc, GlcNAc, mannose, galactose, and fucose—were detected [57]. The glycosylated Ag85A failed to elicit strong humoral immune responses or efficiently activate Ag85A-specific lymphocytes compared with its native prokaryotic counterpart [57]. When an antigen contains potential N-linked glycosylation motifs (Asn-X-Ser/Thr), synonymous amino acid substitutions should be considered to remove these sites while preserving key epitope integrity. For our rationally engineered VB16T13 antigen, glycosylation profiles were assessed by LC-MS/MS, and the binding affinities between specific Fabs and VB16T13 were quantified using surface plasmon resonance (SPR) (Fig 2C-J and Table 1). Immunization studies further demonstrated that VB16T13 retains strong immunogenicity, supporting its potential for development in nucleotide- and virus-based vaccine platforms.

Stringent manufacturing requirements and complex regulatory pathways for outer membrane vesicle (OMV)- and lipoprotein-based vaccines continue to limit MenB vaccine accessibility in resource-limited settings. In our study, a single circular RNA molecule encapsulated in LNPs elicited high levels of functional antibodies and conferred cross-protective immunity in mice. Importantly, LNPs can exhibit self-adjuvant properties, promoting immune activation by inducing robust T follicular helper (Tfh) cell, B-cell, and humoral responses [58]. Our data showed a significant increase in germinal center (GC) B-cell populations in both circRNA-immunized groups compared with the protein vaccine group (Fig 3G). Both Tfh and memory B-cell populations exhibited modest increases in circRNA-vaccinated mice, though these changes did not reach statistical significance (Fig 3F and 3H). The self-adjuvant effect of LNPs in circRNA vaccines may parallel the lipidation of fHbp in Trumenba or OMVs in 4CMenB. Trumenba, a bivalent recombinant vaccine (rLP2086-A05 and rLP2086-B01), elicits potent bactericidal antibodies against diverse MenB strains. This broad strain coverage is partially attributed to N-terminal lipidation of fHbp. Yin *et al.* demonstrated that such lipoproteins function as self-adjuvants through activation of the TLR2 signaling pathway, thereby enhancing protection against MenB [59].

FHbp, an outer membrane lipoprotein, promotes MenB survival in the bloodstream by interacting with the human complement inhibitor factor H [60]. Christina *et al.* demonstrated that incorporation of fHbp alone into an adenoviral vector induced robust antibody responses and durable bactericidal activity following a single immunization [17]. However, antigenic variability in fHbp expression and subfamily distribution among epidemic strains limits its protective breadth [13]. To overcome this limitation, fusing functional epitopes from other MenB antigens to fHbp may enhance strain coverage. In this study, we engineered a circRNA vaccine encoding a fusion antigen combining fHbp and NHBA epitopes, and evaluated its immunogenicity and protective potential against lethal *N. meningitidis* challenge. Sera from circVB16T13-immunized mice exhibited strong binding affinity not only to VB16T13 but also to individual fHbp and NHBA proteins (S5E Fig), suggesting that VB16T13 may confer protection against strains expressing NHBA in their outer membrane.

The SBA assay using human complement is the established correlate of protection for MenB vaccines, consistent with the low incidence of meningococcal disease. In this assay, antibodies bind to pathogens through their Fab regions, while the Fc regions recruit complement components, leading to complement-mediated bacterial killing. In contrast, evaluation of vaccines against intracellular bacteria such as *Mycobacterium tuberculosis* primarily focuses on cell-mediated immunity, particularly IFN-γ-secreting CD4 ⁺ Th1 cells and cytotoxic T lymphocytes (CTLs) [61,62]. However, for extracellular bacteria such as *Neisseria meningitidis*, the contribution of cell-mediated immunity to bacterial clearance remains relatively understudied. In an investigation of an adenovirus-vectored vaccine expressing the fHbp antigen, Christina *et al.* reported a significantly higher proportion of IFN-γ–secreting peripheral blood mononuclear cells (PBMCs) in immunized mice [17]. This vaccine also induced elevated serum IFN-γ levels in subjects during its Phase I clinical trial [63], whereas no comparable increase was detected in recipients of the 4CMenB vaccine. These findings suggest a potential role for IFN-γ in the clearance of MenB infection. In the present study, both low-dose and high-dose circRNA vaccine groups showed marked increases in IFN-γ ⁺ CD8 ⁺ T cells (Fig 3J). Notably, the low-dose group exhibited a higher proportion of IFN-γ ⁺ CD8 ⁺ T cells than the high-dose group (Fig 3J) and conferred complete protection with no observable clinical signs (e.g., ruffled fur) in mice. To further examine whether CD8^+^ T cells play an essential role in the rapid clearance of *N. meningitidis*, we challenged mice with a lethal bacterial dose after depleting CD8^+^ T cells. Although the results indicate that functional antibodies remain the major mediator of protection, the potential protective mechanisms of cellular immunity warrant further investigation. Previous work demonstrated that IFN-γ can mediate the killing of *Staphylococcus aureus*—an extracellular bacterium—in human whole-blood assays [64]. Furthermore, Emily J. *et al.* showed that IFN-γ enhances reactive oxygen species (ROS) production by mast cells, thereby promoting *S. aureus* clearance [65]. Whether a similar IFN-γ-mediated bactericidal mechanism contributes to MenB elimination warrants further investigation.

Broad protection against epidemic strains remains a major challenge in MenB vaccine development. In this study, we analyzed the protective coverage of circVB16T13 and licensed vaccines against a particular panel of prevalent strains in China. Sera from circVB16T13-immunized mice exhibited bactericidal activity against fHbp var 1 strains (5 of 6 strains, hSBA titer ≥4) and showed partial efficacy against fHbp var 2 strains (3 of 10 strains, hSBA titer ≥4) (Table 2). Previous studies demonstrated that the monoclonal antibody 1A12 recognizes epitope residues Ile181, Glu182, Leu184, Val191, and Tyr214, which are fully conserved across all three fHbp variants [36]. This suggests that 1A12 mediates broad cross-protection. Based on this observation, we retained the 1A12-recognized epitope in the VB16T13 design to enhance protective coverage (Fig 2B). To further broaden protection, future RNA- or viral vector-based platforms could be designed to co-express sequences encoding all three fHbp subfamilies.

Nucleic acid vaccines have been widely applied in viral vaccine development, yet their roles and mechanisms of action against bacterial pathogens remain poorly understood. Edo Kon *et al.* developed an mRNA vaccine encoding a cp-caf1 antigen fused with a human Fc fragment to prevent infection by *Yersinia pestis*, an intracellular bacterium [66]. This vaccine elicited robust cellular and humoral immune responses in mice and provided complete protection against a lethal bacterial challenge. Similarly, our circular RNA vaccine targeting MenB elicited potent humoral and cellular immunity, offering full protection against lethal infection. Together, these findings suggest that nucleic acid-based vaccines can mediate effective antibacterial immunity, potentially acting at multiple stages of bacterial invasion, colonization, proliferation, and clearance. The underlying mechanisms, however, warrant further investigation.

In summary, we designed a novel circRNA vaccine encoding a bacterial fHbp-NHBA fusion antigen that conferred protection against lethal MC58 infection at low doses and exhibited bactericidal activity against several prevalent Chinese strains. Our findings highlight the potential of circRNA technology for bacterial vaccine development.

## Materials and methods

### Ethics statement

The 6-week-old female BALB/c mice and ICR (CD-1) outbred mice were ordered from GemPharmatech Co., Ltd. and Zhiyuan Biopharmaceutical Technology Co., Ltd. 7-day-old pups of outbred Wistar rats were ordered from SPF (Beijing) Biotechnology Co., Ltd. All mice were bred and kept under specific pathogen-free (SPF) conditions in the Laboratory Animal Center of Guangzhou National Laboratory. Challenge assays involving *N. meningitidis* were conducted under animal biosafety level 2 (ABSL2) facilities at Guangzhou National Laboratory. The animal experiments were approved by Animal Ethics Committee of Guangzhou National Laboratory (GZLAB-AUCP-2024-08-A09).

### Study design

This controlled preclinical study prospectively evaluated the immunogenicity, cross-protective efficacy, and durability of a circular RNA vaccine encoding a bacterial fHbp-NHBA fusion antigen against *Neisseria meningitidis* serogroup B. The primary endpoints were human serum bactericidal activity (hSBA) titers and survival after lethal MC58 challenge. Secondary endpoints included antigen-specific IgG titers, IgG subclass ratios, bacteremia kinetics, frequencies of T follicular helper cells, germinal-center and memory B cells, and cytokine-producing CD4⁺ and CD8 ⁺ T-cell subsets. Six-week-old BALB/c mice were used for immunogenicity and challenge experiments, outbred CD-1 mice for inter-individual persistence analysis, and seven-day-old Wistar rat pups for passive-transfer assays. Animals were randomly assigned to circRNA-LNP vaccination, recombinant protein vaccination, or licensed-vaccine controls; dosing, routes, and time points were pre-specified. All data were included in the analysis. Group sizes were determined before study initiation based on effect sizes reported in comparable meningococcal vaccine studies and feasibility. The number of animals per group is indicated in the Figure legends. None of the analysis except the histopathology evaluation were performed in a blinded manner. Assays included either two (individual serum tested in SBA and ELISA) or three (pooled serum tested in SBA) replicates.

### Cell culture

All cells were maintained at 37°C in a humidified atmosphere of 5% CO₂. Adherent cells HEK293T, NIH 3T3 and A549 were cultured in Dulbecco’s modified Eagle medium (DMEM) supplemented with 10% fetal bovine serum (FBS) under static conditions. Suspension cells Expi293F were cultured in SMM 293-TII Expression Medium (SinoBiological, Beijing, China) with continuous shaking at 200 rpm.

### Bacterial strains and growth

*N. meningitidis* MC58 strain (ATCC BAA-335) was purchased from ATCC. The epidemic strains used in this study were kindly provided by Walvax Biotechnology Co., Ltd. (Tables 2 and 3). *N. meningitidis* strains were grown for 16-18 h on blood agar plate supplemented with 5% sheep blood (HuanKai Microbial, Guangzhou, China). Fifty individual colonies were then picked and either: (a) transferred to fresh blood agar plates for subculture, or (b) inoculated into Brain Heart Infusion (BHI) broth. All secondary cultures were incubated for 4 h at 37°C before harvesting the bacteria for subsequent experiments. Strains were stored at -80°C in BHI medium supplemented with 20% glycerol.

### Immunization

6-week-old female BALB/c mice were intramuscularly (i.m.) immunized three times at 2-week intervals with either 15 μg circRNA-LNP in 50 μL DPBS or 30 μg circRNA-LNP in 100 μL DPBS. In parallel, mice were immunized subcutaneously (s.c.) 3 times (at the same 2-week intervals) with VB16T13 protein purified from *E.* *coli* and formulated in Freund’s adjuvant. The protein vaccine was administered in a volume of 100 μL per dose, with antigen doses of 20 μg (formulated in complete Freund’s adjuvant), 20 μg (formulated in incomplete Freund’s adjuvant), and 40 μg (formulated in incomplete Freund’s adjuvant). Sera, spleens, and bilateral inguinal lymph nodes were harvested from all groups at indicated time points post-immunization and subjected to antibody or lymphocyte analysis.

To assess protective efficacy *in vivo*, a separate cohort of 6-week-old female BALB/c mice was immunized 3 times at 2-week intervals via i.m. injection with either 15 μg or 30 μg circRNA-LNP. In parallel, mice received s.c. immunizations with 20 μg protein vaccine formulated in Freund’s adjuvant on the same schedule. 2 weeks post-final immunization, all mice were challenged intraperitoneally (i.p.) with a lethal dose of *N. meningitidis* strain MC58. Clinical symptoms, mortality, and bacteremia were monitored.

To evaluate the immunogenicity persistence of circRNA-LNP, 6-week-old ICR (CD-1) outbred mice were i.m. immunized with either a single dose (15 μg) or 2 doses (15 μg each at a 2-week interval) of the vaccine. Serum samples were collected from all groups at indicated time points post-immunization to determine hSBA titers.

To evaluate the hSBA responses elicited by the licensed vaccines 4CMenB and rLP2086, 6-week-old outbred ICR (CD-1) mice were immunized i.p. with 2 doses (1/15 of the human dose) of each vaccine, and BALB/c mice were immunized s.c. with 3 doses (1/10 of the human dose). Serum samples were collected at the indicated time points.

### Serum bactericidal assay (SBA)

*N. meningitidis* was suspended in Hanks’ balanced salt solution (Gibco, Suzhou, China) supplemented with 0.5% BSA and 20 U/mL heparin sodium to a final concentration of 1.25 × 10^4^ CFU/mL. Human complement (Pel-Freez, Rogers, Arkansas) was diluted with SBA buffer mentioned above to a final dilution of 1/4. Sera, pooled or from individual mice, were heat inactivated for 30 min at 56°C and added to the wells in a serial 2-fold dilution, starting with a dilution of 1/4. Control wells contained no serum and heat-inactivated complement. Reaction mixtures were incubated for 1 h at 37°C with shaking (65 rpm) without CO₂ supplementation. Following incubation, 10 µL from each well was plated onto blood agar plates in triplicate and colonies from surviving bacteria counted. Bactericidal activity is quantified as the titer of serum required to kill 50% of bacteria in assays containing both complement and serum in comparison with control assays containing no serum and inactivated complement alone. SBA using pooled sera were repeated twice or three times, and assays using sera from individual mice were repeated twice.

### Challenge assay of active protection

BALB/c mice were challenged 2 weeks post-immunization with the following groups: naïve group (no treatment/uninfected), iron-supplemented control (10 mg iron dextran/uninfected), mock-immunized infected, circRNA-LNP 15 μg infected, circRNA-LNP 30 μg infected, and VB16T13 protein 20 μg infected groups. The infection was conducted in a biosafety cabinet under ABSL2 facilities. *N. meningitidis* strain MC58 was cultured on blood agar plate at 37°C/5% CO₂ for 18 h, followed by inoculation of 50 colonies into 15 mL BHI broth with shaking (37°C, 200 rpm, 4 h); bacterial density was adjusted to OD650 = 0.1 (~2 × 10^8^ CFU/mL) and diluted to 2 × 10^7^ CFU/mL in BHI for challenge, with concentration verified by plating serial dilutions on blood agar. 16 h prior to infection, mice received 10 mg iron dextran (100 μL volume) intramuscularly, followed by intraperitoneal challenge with 100 μL bacterial suspension (2 × 10^6^ CFU). Mortality was recorded at 12-hour intervals for 48 h and daily through Day 13; bacteremia was assessed at 1, 3, 6, 9, and 12 h post-infection by collecting pooled whole blood into heparinized BHI (5 U/mL) at an initial 1:5 dilution; this suspension was then serially diluted 10-fold 5 times, and plating 10 μL aliquots in quadruplicate on blood agar plate. Survivors were euthanized at Day 13 for histopathological analysis of hearts, livers, spleens, lungs, kidneys, and brains after 4% PFA fixation.

### Statistical analysis

Flow cytometry data were analyzed using FlowJo software v10.8.1. Comparisons between two groups were conducted by unpaired, two-tailed Student’s t-test. Comparisons among more than two groups were conducted by one-way ANOVA and Two-way ANOVA comparison tests. Comparisons of the survival data were conducted by Log-rank (Mantel-Cox) tests. Statistical analyses were performed using GraphPad Prism 8 and the details are provided in the figure legends.

## Supporting information

S1 Text Supplementary methods. (DOCX)

S1 Table Reagents used in this study. (DOCX)

S2 Table Details of antibodies used in this study. (DOCX)

S3 Table Detail primer sequences used in this study. (DOCX)

S4 Table Endotoxin testing of *E. coli*-expressed VB16T13 by the gel-clot TAL assay. TAL reagent sensitivity was λ = 0.25 EU/mL. Endotoxin-free water (0 EU/mL) and an endotoxin standard (0.5 EU/mL) were included as the negative and positive controls, respectively. A positive product control (PPC) was prepared by spiking the 1:2 diluted VB16T13 sample with endotoxin to a final concentration of 0.25 EU/mL. VB16T13 was tested at the indicated dilutions (1:2-1:8192). Results are shown as gelation (+) or no gelation (−) in two technical replicates (Rep 1-2). Based on a positive result at 1:2 and a negative result at 1:16, the endotoxin level in the stock formulation was reported as <4 EU/mL (i.e., < 0.4 EU per 100-µL dose).
(DOCX)

S5 Table Bactericidal activity by mAbs 4B3 and 1E6. The mixture of 4B3 and 1E6 were serially diluted, and subjected to serum bactericidal assay (SBA) against MC58, 50% bactericidal activity was observed at a concentration of 7.81 μg/mL.
(DOCX)

S1 Fig circVB16T13 exhibits defined biophysical properties. **(A)** Sanger sequencing spanning the backsplice junction (dotted line). Reverse primer confirmed circularization fidelity. **(B)** E-gel analysis of RNase H digestion products of precursor RNA, nicked control, and circVB16T13. * indicated the RNase H cleavage products. **(C)** Two-step purification workflow: Upper: Size-exclusion chromatography (SEC) profile (red line area presented the collected fractions). Lower: E-gel showing the contents of SEC fractions. **(D)** Capillary gel electrophoresis with laser-induced fluorescence detection (CGE-LIF) assessment of purification efficiency. Precursor: linear IVT product; IVT: *in vitro* transcription (circularization was concurrently performed during IVT); RNR: RNase R-treated sample; SEC: size-exclusion chromatography fraction; Nicked: linearized RNA control with the same nucleotide sequence as circVB16T13. **(E, F)** Thermal stability assessment. **(E)** E-gel of circVB16T13 stocked at 4°C/37°C for 0–30 days. **(F)** Quantification of VB16T13 expression from circRNA by ELISA after storage at the indicated temperatures. Comparisons among more than two groups were conducted by Two-way ANOVA comparison tests.
(TIF)

S2 Fig Sequence optimization enhances circVB16T13 antigen expression. **(A, B)** circVB16T13 secondary structures predicted by ViennaRNA Package 2.0. **(A)** Native circVB16T13 sequence (∆G = -364.0 kcal/mol). **(B)** LinearDesign-optimized sequence (∆G = -699.8 kcal/mol). **(C)** VB16T13 standard curve for quantitative ELISA (R² = 0.9934). **(D)** Antigen levels in cell culture supernatants at 24, 48, 72, and 96 h after transfection with 1 μg circRNA. The mean protein concentrations at 96 h are indicated in the figure. Comparisons among more than two groups were conducted by Two-way ANOVA comparison tests.
(TIF)

S3 Fig LC-MS/MS revealed the glycosylation modifications on eukaryotically expressed VB16T13. **(A)** Top 20 glycosylation modifications in human-derived VB16T13.
(TIF)

S4 Fig Humoral responses elicited by the VB16T13 protein vaccine formulated with different adjuvants or doses, compared with 4CMenB. **(A)** hSBA titers of pooled serum from BALB/c mice after three immunizations with VB16T13 formulated with different adjuvants, with 4CMenB included as a comparator (n = 5 per group). **(B)** VB16T13-specific IgG titers in individual mouse serum after three immunizations with VB16T13 formulated with different adjuvants or doses (n = 5 per group).
(TIF)

S5 Fig circVB16T13 induces comprehensive humoral and cellular immunity. **(A-D)** Antigen-specific IgG subclass kinetics at 2 weeks after prime, 1st boost, and 2nd boost administration. **(A)** IgG1, **(B)** IgG2a, **(C)** IgG2b, and **(D)** IgG3. The limit of detection in prime = 2.6, in 1st boost = 3.6, and in 2nd boost = 4.28. The fold changes in GMT between 2nd boost and prime in each immunized group were labeled above. **(E)** Cross-reactivity profiling by endpoint ELISA. Sera IgG titers from circVB16T13-immunized mice recognized with VB16T13, fHbp, and NHBA.
(TIF)

S6 Fig Gating strategies for lymphocyte analysis by flow cytometry. **(A)** Gating strategy for GC B cells and memory B cells in the ILNs. GC B cell designation criteria: CD45 + CD45R+GL7 + Fas + . Memory B cell designation criteria: CD45 + CD45R+IgG1 + CD38 + . **(B)** Gating strategy for Tfh cells in the ILNs. Tfh cell designation criteria: CD4 + CD44 + PD-1 + CXCR5 + . **(C)** Gating strategy for CD4 + T and CD8 + T cells that secrete IFN-γ, TNFα, or IL-2 in the spleens.
(TIF)

S7 Fig Establishing a MC58 meningococcal challenge model in BALB/c mice. **(A)** Schematic of challenge model construction procedure. This figure was created using Figdraw (www.figdraw.com) with permission. **(B)** Quantitative bacteremia kinetics in mice pretreated intramuscularly with 2.5, 5, 10, or 15 mg/mouse. Blood bacterial loads measured at 1-, 3-, 6-, and 9-hour post-infection (hpi). Dashed line indicates detection limit (500 CFU/mL). **(C)** Survival curves of mice pretreated with indicated doses (2.5-15 mg/mouse). **(D, E)** Clinical sign comparison between MC58-infected and BHI iron control groups: **(D)** Body temperature measurements at 0, 3, 6, and 9 hpi. **(E)** Body weight measurements at 17 h before (-17 h), and 0, 3, 6, 9 hpi. **(F)** Quantitative bacteremia kinetics at challenge doses of 2 × 10⁵-2 × 10⁷ CFU, measured at 3, 6, and 9 hpi. Dashed line indicates detection limit (500 CFU/mL). **(G)** Survival rates over 72 hours post-infection at challenge doses of 2 × 10⁵-2 × 10⁷ CFU. Comparisons among more than two groups were conducted by Two-way ANOVA comparison tests. Comparisons of the survival data were conducted by Log-rank (Mantel-Cox) tests.
(TIF)

S8 Fig Histopathological analysis of tissues at 13 dpi. **(A)** Representative heart sections. Red arrows indicate inflammatory cells. **(B)** Representative kidney sections. Black arrows indicate hemorrhage, red arrows indicate inflammatory cells, and green arrows indicate fibrosis. **(C)** Representative spleen sections. Black arrows indicate hyperemia, and red arrows indicate multinucleated giant cells. **(D)** Representative liver sections. Red arrows indicate inflammatory cells, and green arrows indicate fibrosis. Scale bars: 50 μm. Comparisons among more than two groups were performed using one-way ANOVA.
(TIF)

S9 Fig CD8^+^ T-cell depletion before lethal MenB challenge. **(A)** Experimental design for MC58 challenge. BALB/c mice (n = 5 per group) were challenged intraperitoneally with 1 × 10⁷ CFU at 14 days after the final immunization. Anti-CD8α antibody (200 μg per mouse) was administered intraperitoneally on days -3 and -1 before challenge. This figure was created using Figdraw (www.figdraw.com) with permission. **(B)** Kinetics of bacteremia in pooled whole blood after challenge. Bacterial loads were quantified at 1, 3, and 6 h post infection; the dashed line indicates the limit of detection (500 CFU/mL). **(C)** Survival of mice monitored for 3 days after challenge.
(TIF)

S1 Data Values used to build graphs. Images files. SPR PDF files. CE5200 PDF file. S1D Fig report. S1A Fig sequencing file.
(ZIP)
