# Supplementary material for: A circular RNA vaccine induces durable and cross-protective immunity against Neisseria meningitidis serogroup B in mice
Source: PLoS Pathog. 2026 May 11;22(5):e1013741. doi: 10.1371/journal.ppat.1013741 (PMC13160355; doi:10.1371/journal.ppat.1013741)
Supplement: S1 Text — (DOCX) [file ppat.1013741.s001.docx]

S1 Text

**Supplementary methods**

**Plasmid construction**

The circVB16T13 was constructed using the group I catalytic intron in the thymidylate synthase (Td) gene of the T4 phage[1]. The 5’ homology arm, 3’ group I intron-exon, an upstream spacer, CVB3 IRES, kozak sequence, signal peptide sequence originating from the Ig light chain variable region (GenBank, account number U43767.1), a downstream spacer, 5’ group I exon-intron and 3’ homology arm sequence were PCR amplified and cloned into a plasmid backbone via the Gibson assembly strategy, generating the empty pCircRNA backbone containing a T7 RNA polymerase promoter. Then, the LinearDesign algorithm[2] optimized fHbp-NHBA fusion protein-coding sequence was PCR amplified using 2× Phanta Flash Master Mix (Vazyme) and cloned just downstream of the SP sequence into the pCircRNA backbone using ClonExpress Ultra One Step Cloning Kit (Vazyme), and the corresponding pCircRNA plasmids was constructed for the following IVT reaction.

**circRNA synthesis and purification**

The production of circRNAs was performed according to previous reports with little modification[3]. Briefly, the circRNA precursors were synthesized via *in vitro* transcription (IVT) from the linearized circRNA plasmid templates with the T7 High Yield RNA Synthesis Kit (YEASEN). For control linear precursor production, IVT was performed identically but with Mg²⁺ concentration reduced to 7.5 mM to prevent circularization during transcription, yielding the linear precursor standard. After IVT, the RNA products were treated with DNase I (Thermo Fisher Scientific​) at 37℃ for 15 min to digest the DNA templates and extracted using lithium chloride (LiCl, 7.5M, Thermo Fisher Scientific​). RNA samples were then heated at 70℃ for 5 min and immediately cooled on ice. The reactions were treated with RNase R at 37℃ for 20 min to enrich the circRNAs. The RNase R-treated RNAs were extracted using lithium chloride. To further enrich the circRNAs, the purified RNase R-treated RNAs were resolved with high-performance liquid chromatography (Unimicro Easysep-3030) using a 250mm×20mm BIOBASIC size-exclusion column with a particle size of 5 μm and pore size of 1000 Å (Thermo Fisher Scientific) in RNase-free TE buffer (pH 8.0). The circRNA-enriched fractions were collected and extracted using lithium chloride and dissolved in nuclease-free water. Compared to circRNAs, the nicked linear RNA counterpart without 5’ intron and 3’ intron terminal cannot be circularized. RNA concentrations were determined using NanoDrop One (Thermo Fisher Scientific).

**RNase H cleavage assay**

The purified circRNAs, nicked linear RNAs and linear precursors were incubated with RNase H[1] (New England Biolabs​). Site-specific cleavage was performed in reactions containing 2 μg of the targeted RNAs, 3 μL of antisense ssDNA probe (10 μM) and RNase H buffer in a total volume of 49 μL. After incubation at 70℃ for 5 min, the reaction systems were then annealed at room temperature for 1 h, and 1 μL of RNase H was added to the reaction at 37℃ for 20 min. The sequence of the antisense primer is 5’-GTGAGCAGTCTATTGATACTCAGTC-3’.

**LNP encapsulation**

The circRNAs were encapsulated with lipid nanoparticles (LNPs). First, the circRNAs were diluted with sodium acetate (50mM, pH 4.0) to a final concentration of 150 μg/mL. Then, the lab-prepared LNP in the ethanol phase (SM102 ionizable lipid, DSPC, cholesterol, and DMG-PEG2000 at molar ratios of 50: 10: 38.5: 1.5)[4] were mixed with the circRNA solutions at the volume ratio of 1:3 through the Feather Microfluidic Chip (Apexbt) using Nanodispatcher L (RNACure RM1001). Then the LNP-circRNA formulations were diluted 5-fold with DPBS buffer (pH 7.4) and concentrated by ultrafiltration with Amicon Ultra Centrifugal Filter (100kDa). The concentration and encapsulation rate of circRNAs were measured by the Quant-iT RiboGreen RNA Assay Kit (Invitrogen). The size of LNP-circRNA particles were measured using dynamic light scattering on a Zetasizer Ultra (Malvern Panalytical).

**Transfection**

In brief, HEK293T cells were transfected with each circRNA or mRNA (for 12-well plates, 0.8 μg per well; for 6-well plates, 1 μg per well) using mRNA transfection reagent (YEASEN) according to the manufacturer’s instructions. To test the expression of VB16T13 antigen, the HEK293T cell supernatant were collected at 24, 48, 72 and 96 h after transfection for further analyzing by ELISA. A549 cells were transfected with circRNAs or mRNAs encapsulated with LNPs (for 6-well plates, 1 μg per well). To test the innate immunity stimulated by circRNAs and mRNAs, the total RNAs of A549 cells were harvested at 48 h after transfection for reverse transcription and qRT-PCR.

**Proteins, enzyme, and antibodies purification**

The VB16T13 fusion protein, consisting of fHbp variant 1.1 and NHBA variant P3 (from strain MC58) via a flexible linker, was produced in *E. coli*, Expi293F, and NIH 3T3 cells respectively. In brief, the coding sequence of VB16T13 was optimized according to *E. coli* codon usage and cloned into pET-30a with a C-terminal 6× His-tag. VB16T13 was expressed in *E. coli* BL21 (DE3) and purified with Ni-NTA affinity column chromatography (GenScript) followed by size-exclusion chromatography (Superdex 75 Increase 10/300 GL, Cytiva). Endotoxins were removed using a Pierce High-Capacity Endotoxin Removal Spin Column (Thermo Scientific). The coding sequence of VB16T13 was optimized according to human codon usage and cloned into pcDNA3.1 with an N-terminal signal sequence and a C-terminal 6 × His-tag. For murine-derived VB16T13, the coding sequence was optimized according to murine codon usage; all other constructs and procedures were identical. After transfection with pcDNA3.1-VB16T13, the culture supernatants of Expi293F and NIH 3T3 cells were harvested, and VB16T13 was purified with Ni-NTA affinity column chromatography followed by size-exclusion chromatography (Superdex 75 Increase 10/300 GL). The proteins were verified by western blot analysis using HRP-conjugated anti-6× His mouse monoclonal antibody. RNase R was expressed in *E. coli* BL21 (DE3) and purified with Ni-NTA affinity column chromatography followed by size-exclusion chromatography (Superdex 200 Increase 10/300 GL). For expression of monoclonal antibodies (mAbs) 1A12, 4B3, and 1E6, the variable regions of the light and heavy chains (with the heavy-chain variable region fused to a human IgG1 Fc) were codon-optimized for mammalian expression. For Fab expression (1A12, 4B3, 1E6, 12C1, 7B10, JAR4, JAR5, and 10C3), the variable regions of the light and heavy chains were codon-optimized for mammalian expression, and a C-terminal 6× His tag was fused to the heavy-chain variable region. The optimized sequences were synthesized and cloned into the pcDNA3.4. The recombinant antibody was transiently expressed in Expi293F cells by transfecting the cells with plasmids encoding the light chain (LC) and heavy chain (HC) at a 3:2 plasmid mass ratio. Six days after transfection, the culture supernatants of Expi293F cells were harvested, and mAbs were purified with Protein A resin (GenScript) followed by size-exclusion chromatography (Superdex 200 Increase 10/300 GL), whereas Fabs were purified by Ni-NTA affinity chromatography followed by size-exclusion chromatography (Superdex 75 Increase 10/300 GL). Protein concentration was determined using NanoDrop One (Thermo Fisher Scientific ​).

**Gel-clot TAL assay**

Endotoxin contamination in *E. coli*-expressed VB16T13 was assessed using a gel-clot tachypleus amebocyte lysate (TAL) assay (λ = 0.25 EU/mL). VB16T13 was serially diluted in endotoxin-free water (1:2-1:8192). Endotoxin-free water and an endotoxin standard (0.5 EU/mL) were run in parallel as negative and positive controls, respectively. To verify the absence of assay interference, a positive product control was prepared by spiking the diluted VB16T13 sample with endotoxin to a final concentration of 0.25 EU/mL. For each reaction, 100 μL of sample (or control) was mixed with 100 μL of TAL reagent. Reactions were incubated at 37°C for 60 min. Clot formation was visually scored, and each condition was tested in duplicate.

**Capillary gel electrophoresis with laser-induced fluorescence detection (CGE-LIF)**

The RNA 9000 Purity & Integrity kit containing the nucleic acid extended range gel, SYBR Green II RNA Gel Stain, acid wash (regenerating solution), CE-grade water and the ssRNA Ladder (0.05-9 kb) was from SCIEX (Framingham, MA). The pre-assembled capillary cartridge (30.2 cm total length, 20 cm effective length), and the sample loading solution (SLS) were also from SCIEX[5]. For the purity analysis of circRNA products, a working stock solution of the circRNA products was prepared at 2 ng/μL by serial dilution of the main stock (20 ng/μL) with the SLS solution. Samples were heated for 5 min at 70℃, and then immediately placed on ice and cooled for at least 10 min. SCIEX PA 800 plus pharmaceutical analysis system with laser-induced fluorescence (LIF) detector was used for the purity analysis of linear RNA and circRNA samples. The excitation and emission wavelength used were 488 nm and 520 nm, respectively. The sample temperature was set to 10℃ with the capillary temperature at 30℃. The prepared sample was injected under a voltage of 1 kV with reversed polarity for 3 s. Prior to analysis, the capillary was preconditioned by sequentially rinsing with water at 50 psi for 5 min, 0.1 N HCl at 20 psi for 5 min, water at 20 psi for 2 min, and a separation gel solution at 50 psi for 10 min. Finally, it was equilibrated under a reversed polarity voltage of 6 kV for 10 min. For the separation method, the capillary was rinsed with 0.1 N HCl at 50 psi for 5 min, followed by water at 50 psi for 5 min. Subsequently, it was rinsed with a separation gel solution at 50 psi for 5 min and separated under a reversed polarity voltage of 6 kV for 40 min.

**E-Gel electrophoresis**

RNA samples (200 ng total RNA per well) were added in a loading mixture consisting of formamide and 0.5 µL of 0.5 M EDTA. The total loading volume per sample was 20 µL. An RNA Ladder (Thermo Fisher Scientific​) was prepared similarly by mixing 1 µL of ladder with 19 µL of formamide. Samples and ladder were heated to 70℃ for 5 min, and chilled on ice for 5 min. Samples were loaded onto 2% E-Gel EX Agarose Gels (Invitrogen) and run on the E-Gel Powersnap Electrophoresis System (Invitrogen) using EX 1-2% program for 10-20 min. Images of the agarose gels were captured using a fully automated gel documentation system (BIO-OI 100).

**Capillary electrophoresis (CE)**

RNA samples were diluted to 200 ng/μL in nuclease-free water, heated at 70°C for 5 min, and immediately chilled on ice for 5 min to denature secondary structures. 2 μL of each denatured sample was loaded onto 5200 Fragment Analyzer (Agilent) using the DNF-471 (15 nt) RNA kit (Agilent), following the manufacturer’s protocol for capillary electrophoresis separation and detection.

**Reverse transcription and qRT-PCR**

Total RNA was isolated from transfected A549 cells 48 h after transfection. Briefly, cells were washed twice with ice-cold DPBS (pH 7.4) and lysed directly in the culture dish using 1 mL of TRIZOL Reagent (Invitrogen) for 1 min. 200 μL of Chloroform was added to the lysate, followed by centrifugation (12000×g for 15 min at 4℃) to separate the aqueous and organic phases. The RNA-containing aqueous phase was transferred to a new tube, mixed with an equal volume of room-temperature isopropanol. The mixture was incubated at -20℃ for 30 min to precipitate the RNA, followed by centrifugation (12000×g for 10 min at 4℃) to harvest the RNA precipitation. The RNA precipitation was washed once with 80% ethanol, air-dried, and finally resuspended in RNase-free water. RNA concentration and purity were assessed spectrophotometrically using NanoDrop One (Thermo Fisher Scientific). qRT-PCR was carried out using HiScript II One Step qRT-PCR SYBR Green Kit (Vazyme). The primers used in this study are listed in S3 Table.

**CircRNA stability *in vitro***

CircRNA stability was assessed by storing RNase R-treated circRNA in nuclease-free water at 4℃ (long-term stability) and 37℃ (accelerated degradation) for 0, 3, 7, 14, and 30 days. At each timepoint, RNA samples underwent the following analyses: (a) integrity verification via E-gel electrophoresis to confirm purity and structural integrity of the transfected RNA; (b) functional testing where 0.8 μg RNA was transfected into HEK293T cells seeded in 12-well plates. After 48 h incubation, cell supernatants were harvested for quantification of circRNA-encoded antigen expression by ELISA.

**Quantitative determination of VB16T13 expression *in vitro***

VB16T13 protein levels in cell supernatants were quantified by ELISA. Briefly, 96-well plates (Nest) were coated with capture antibody 1A12 (1 μg/mL, 100 μL/well) overnight at 4℃, followed by blocking with 2% BSA in PBST (100 μL/well, 2 h, 37°C). After washing, cell supernatants diluted at proper ratio and VB16T13 standards (2-fold serial dilutions from 2500 to 39 ng/mL in DPBS, 100 μL/well) were added and incubated at 37℃ for 1 h. Plates were then probed with HRP-conjugated anti-6× His mouse monoclonal antibody (1:5000 dilution in PBST, 100 μL/well, 1 h, 37℃). Subsequent TMB substrate development (5 min, RT) and absorbance measurement at 450 nm were performed. VB16T13 concentrations were interpolated from the standard curve.

**Surface plasmon resonance (SPR)**

Biacore 8K+ (Cytiva) was used to determine the binding affinity of recombinant VB16T13 antigens and Fabs (1A12, 4B3, 1E6, 12C1, 7B10, JAR4, JAR5, and 10C3). Antigens were immobilized via amine coupling on Seriers S Sensor Chip CM5 using 10 mM sodium acetate (pH 5.0), achieving approximately 300 RU coupling density per flow channel. Fabs were serially diluted in PBST (pH 7.4) across an 8-point concentration gradient (0, 3.125, 6.25, 12.5, 25, 50, 100, and 200 nM). Binding assays were conducted at 30 μL/min flow rate with a 120 s association phase and a 600 s dissociation phase (1800 s for 1A12), followed by surface regeneration using 10 mM glycine-HCl (pH 1.5). After fitting the experimental data to a 1:1 Langmuir binding model in Biacore 8K+ evaluation software, the binding dynamics were obtained.

**Liquid chromatography-tandem mass spectrometry (LC-MS/MS)**

Sample preparation was essentially performed as previously described with slightly modification[6]. In brief, excised gel bands were cut into cubes of 1-2 mm. For coomassive-stained gels, gel cubes were destained using 50 mM ammonium bicarbonate (Sigma)/acetonitrile (Fisher chemical) (2:3, vol/vol). Subsequently, gel cubes were dehydrated with neat acetonitrile with shaking, until gel pieces become white and shrink and then remove acetonitrile. Next, protein gel cubes were reduced with 10 mM dithiothreitol (Sigma) (30min at 56℃), alkylated with 55 mM iodoacetamide (Sigma) (45 min at RT in the dark), digested with trypsin (Promega) (1h, 50℃) in 50 mM ammonium bicarbonate containing 0.01% ProteaseMAX Surfactant (Promega). Peptide digestion products were collected from the gel supernatant. Further extracted the gel pieces with 0.1% Formic acid (Fisher chemical) with 5 min sonication in a water bath. All supernatants were combined in a fresh tube, treated with trifluoroacetic acid to stop the digestion, and then desalted with home-made C18 StageTips.

Peptide samples were analyzed by liquid chromatography-tandem mass spectrometry (LC-MS/MS) by combining a Vanquish Neo connected online to an Orbitrap Exploris 480 mass spectrometer (Thermo Fisher Scientific). A 250mm Acclaim PepMap100 C18 column (Thermo Fisher Scientific) with internal diameter of 75 μm was used to separate the peptides with mobile phase A (0.1% FA in water) and mobile phase B (0.1% FA in 80% ACN) at a 60 min gradient: 4-4.5% B (400 nL/min) in 1.8min, 4.5-5% B (400 nL/min to 300 nL/min) in 0.2 min, 5-23% B (300 nL/min) in 39 min, 23-40% B (300 nL/min) in 16 min, 40-55% B (300 nL/min to 400 nL/min) in 0.5 min, 55-90% B (400 nL/min) in 0.5 min and then kept B (400 nL/min) at 90% for 2 min. The Orbitrap Exploris 480 mass spectrometer was operated in a data-dependent acquisition mode. MS1 data were collected using the Orbitrap (120,000 resolution; AGC target Standard, maximum injection time 50 ms). Determined charge states between 2 and 7 were required for sequencing and a 45 s dynamic exclusion window was used. The MS2 stage consisted of fragmentation by HCD (normalized collision energy 30%) and analysis using the Orbitrap (30,000 resolution, AGC target 100%, maximum injection time 54 ms, isolation window 1.6 m/z). The cycle time was set at 2 s.

**Enzyme-linked immunosorbent assay (ELISA)**

Antigen-specific antibody titers were determined by ELISA. For detection of total IgG against specific antigens, sera underwent serial 10-fold dilutions in PBST starting at 1:100, applied to 96-well plates (100 μl/well, Nest) coated with VB16T13 (50 ng/well) and blocked with 2% BSA (2 h, 37℃). After incubation, plates were washed and probed with HRP-conjugated rabbit anti-mouse IgG (1:5000 in PBST, 1 h, 37℃), followed by TMB substrate development and absorbance measurement at 450 nm. Titers were defined as the highest dilution yielding OD450 ≥2.1× background controls (without serum but the secondary antibody was added). For specific IgG profiling against individual antigens (VB16T13, fHbp, and NHBA), plates were separately coated with each antigen (50 ng/well), sera were subjected to 5-fold serial dilutions starting at 1:5000, and detection followed the same secondary antibody and titer calculation protocol. For IgG subclass analysis (IgG1, IgG2a, IgG2b, and IgG3), plates coated with VB16T13 (50 ng/well) were incubated with 4-fold serially diluted sera, followed by subclass-specific HRP-conjugated secondary antibodies (1:10000 dilution in PBST, 30 min, 37℃) prior to substrate reaction and titer determination.

**Intracellular cytokine staining (ICS) assay**

Lymphocytes were isolated from spleens by mechanical dissociation. Briefly, the spleen capsule was disrupted by puncturing it multiple times with a sterile needle attached to a 1 mL syringe. Cells were then gently flushed from the parenchyma through the puncture sites using RPMI-1640 medium until the capsule appeared translucent. The resulting cell suspension was subjected to density gradient centrifugation with a Mouse Spleen Lymphocyte Isolation Kit (TBD) according to the manufacturer’s protocol. For intracellular cytokine staining, cells were seeded into 24-well plates (2 × 10⁶ cells per well) and subjected to the following treatments: Experimental groups were stimulated with VB16T13 protein (30 μg/ml) for 18 h; Positive control group was stimulated with Leukocyte Activation Cocktail (2 μL/well, BD) for 2 h; unstimulated cells were used as negative controls. Following stimulation, all groups were treated with protein transport inhibitor containing brefeldin A (1 μL/well, BD) and incubated at 37℃ for 6 h. After washing once with DPBS, cells were stained for viability using Fixable Viability Stain 510 (1 μL in 1 mL DPBS, 15 min, RT, light-protected), then blocked with mouse Fc blocker (Purified Rat Anti-Mouse CD16/CD32, BD) on ice for 5 min. Surface staining was performed in DPBS containing 2% FBS on ice for 30 min using: CD3e (FITC, clone 145-2C11, BD), CD4 (APC, clone RM4-5, BD), and CD8α (PerCP-Cy5.5, clone 53-6.7, BD). Cells were fixed/permeabilized with Cytofix/Cytoperm buffer (BD, 4℃, 20 min), stained intracellularly with antibodies against IFN-γ (PE, clone XMG1.2, BD), TNF-α (BV421, clone MP6-XT22, BD) and IL-2 (BV605, clone JES6-5H4, BD), and analyzed on a Beckman Coulter CytoFlex S.

**Flow cytometry of Tfh, GC B and memory B cells**

In brief, lymphocytes were isolated from the ILNs by passing through 70 μm Cell Strainer (Biosharp). Cells were stained for viability using Fixable Viability Stain 780 (BD) and then blocked with an Fc receptor antibody α-CD16/32 (clone: 93, BL) on 4℃ for 20 min, followed by staining with fluorochrome-conjugated antibodies in DPBS with 2% FBS: for the Tfh cell panel, CD4 (BUV737, clone: SK3, BD), CD44 (BV421, clone: IM7, BD), CXCR5 (FITC, clone: L138D7, BL), PD-1 (PE, clone: 29F.1A12, BL); for the GC B cell and memory B cell panels, CD45 (Alexa Fluor 700, clone:30-F11, BD), CD45R (BV650, clone: RA3-6B2, BL), CD38 (BV605, clone: 90/CD38, BD), CD95(Fas) (PE, clone: Jo2, BD), GL7 (PE-CY7, clone: GL7, BL), IgG1 (BV421, clone: RMG1-1, BL) .The staining was performed on 4℃ for 45 min. Finally, cells were fixed and analyzed by Sony ID7000.

**Passive protection in infant rats**

7-day-old Wistar rat pups (12-15 g) were used for passive immunization. Pooled sera collected 2 weeks post second boost immunization from 3 donor groups—mock-immunized mice, circRNA-LNP-immunized mice (15 µg per dose), and VB16T13 protein-immunized mice (20 µg per dose)—were heat-inactivated (56℃, 40 min), diluted 1:10 in DPBS, and administered via subcutaneous injection (100 μL) 2 h pre-infection. 16 h prior to infection, all pups received 5 mg iron dextran intramuscularly (50 μL volume). *N. meningitidis* strain MC58 was prepared as previously described and subcutaneously challenged with 100 μL bacterial suspension (1×10^6^ CFU). Bacteremia quantification at 3 h post-infection: pooled whole blood was diluted 1:5 in heparinized BHI (5 U/mL), serially diluted 10-fold 5 times, and 10 μL aliquots plated in quadruplicate on blood agar.

**References**

1. Wesselhoeft RA, Kowalski PS, Parker-Hale FC, Huang Y, Bisaria N, Anderson DG. RNA Circularization Diminishes Immunogenicity and Can Extend Translation Duration In Vivo. Molecular cell. 2019;74(3):508-20.e4.

2. Zhang H, Zhang L, Lin A, Xu C, Li Z, Liu K, et al. Algorithm for optimized mRNA design improves stability and immunogenicity. Nature. 2023;621(7978):396-403.

3. Wesselhoeft RA, Kowalski PS, Anderson DG. Engineering circular RNA for potent and stable translation in eukaryotic cells. Nature communications. 2018;9(1):2629.

4. Hassett KJ, Benenato KE, Jacquinet E, Lee A, Woods A, Yuzhakov O, et al. Optimization of Lipid Nanoparticles for Intramuscular Administration of mRNA Vaccines. Molecular therapy Nucleic acids. 2019;15:1-11.

5. Camperi J, Lippold S, Ayalew L, Roper B, Shao S, Freund E, et al. Comprehensive Impurity Profiling of mRNA: Evaluating Current Technologies and Advanced Analytical Techniques. Analytical chemistry. 2024;96(9):3886-97.

6. Shevchenko A, Tomas H, Havlis J, Olsen JV, Mann M. In-gel digestion for mass spectrometric characterization of proteins and proteomes. Nature protocols. 2006;1(6):2856-60.
