# Supplementary material for: A circular RNA vaccine induces durable and cross-protective immunity against Neisseria meningitidis serogroup B in mice
Source: PLoS Pathog. 2026 May 11;22(5):e1013741. doi: 10.1371/journal.ppat.1013741 (PMC13160355; doi:10.1371/journal.ppat.1013741)
Supplement: S1 Table — (DOCX) [file ppat.1013741.s002.docx]

**S1 Table.** Regents used in this study.

| **REAGENT or RESOURCE** | **SOURCE** | **IDENTIFIER** |
| --- | --- | --- |
| DMEM basic (1×) | Gibco | Cat#C11995500BT |
| SMM 293-TII Expression Medium | SinoBiological | Cat#M293TII-N |
| Fetal Bovine Serum | Sigma | Cat#F0193 |
| Blood Agar Plate | HuanKai Microbial | Cat#024070 |
| Brain Heart Infusion Broth | Hopebio | Cat#HB8297-5 |
| Glycerol | Beyotime | Cat#ST1348-1L |
| 2× Phanta Flash Master Mix | Vazyme | Cat#P510 |
| ClonExpress Ultra One Step Cloning Kit | Vazyme | Cat#C115 |
| HiScript III RT SuperMix | Vazyme | Cat#R323-01 |
| DNase I | Thermo Fisher Scientific | Cat#EN0521 |
| High Affinity Ni-Charged Resin FF | GenScript | Cat#L00666-5 |
| Protein A resin | GenScript | Cat#L00210-10 |
| Superdex 200 Increase 10/300 GL | cytiva | Cat#28990944 |
| Superdex 75 Increase 10/300 GL | cytiva | Cat#29148721 |
| LiCl 7.5M | Thermo Fisher Scientific | Cat#AM9480 |
| BIOBASIC SEC 1000, 5μm | Thermo Fisher Scientific | PN73605-259270A |
| RNase H | New England Biolabs | Cat#M0297S |
| Feather Microfluidic Chip | Apexbt | Cat#RM1002-1 |
| SM102 | TargetMol | Cat#T9410 |
| DSPC | AVT | Cat#S01005 |
| Cholesterol | AVT | Cat#001001 |
| DMG-PEG2000 | AVT | Cat#O02005 |
| Amicon Ultra Centrifugal Filter | Merck Millipore | Cat#UFC910024 |
| mRNA Transfection Reagent | Yeasen | Cat#40809ES03 |
| TRIZOL | Invitrogen | Cat#10296028CN |
| 2% E-gel EX agarose gels | Invitrogen | Cat#G401002 |
| RNA Ladder | Thermo Fisher Scientific | Cat#SM1823 |
| Ammonium bicarbonate | Sigma | Cat#A6141 |
| Acetonitrile | Fisher chemical | Cat#A955-4 |
| Dithiothreitol | Sigma | Cat#D9779 |
| Iodoacetamide | Sigma | Cat#I6125 |
| Trypsin | Promega | Cat#V5111 |
| ProteaseMAX Surfactant | Promega | Cat#V2071 |
| Formic acid | Fisher chemical | Cat#LS118-1 |
| Freund’s Adjuvant, Incomplete | Sigma | Cat#F5506-10ML |
| Freund’s Adjuvant, Complete | Sigma | Cat#F5881-10ML |
| ELISA 96-well plates | Nest | Cat#514201 |
| ELISA coating buffer (10×) | Solarbio | Cat#C1055 |
| BSA (for ELISA) | ABCone | Cat#B24726-250G |
| PBST (10×, pH 7.4) | Biosharp | Cat#BL314B |
| TMB | Solarbio | Cat#PR1200 |
| ELISA stop solution | Solarbio | Cat#C1058 |
| Hanks’ Balanced Salt Solution | Gibco | Cat#C14175500BT |
| BSA (for SBA) | Sigma | Cat#A1933 |
| Heparin sodium | Sigma | Cat#H3393 |
| Human complement | Pel-Freez | Cat#34010-10 |
| RPMI-1640 | Gibco | Cat#C11875500BT |
| Leukocyte Activation Cocktail | BD Biosciences | Cat#550583 |
| Protein Transport Inhibitor | BD Biosciences | Cat#555029 |
| Cytofix/Cytoperm | BD Biosciences | Cat#554722 |
| 70μm Cell Strainer | Biosharp | Cat#BS-70-ECS |
| CM5 | Cytiva | Cat#BR100530 |
| Fixable Viability Stain 780 | BD Biosciences | Cat#565388 |
| Fixable Viability Stain 510 | BD Biosciences | Cat#564406 |
| T7 High Yield RNA Synthesis Kit | Yeasen | Cat#10623ES50 |
| RNA 9000 Purity & Integrity Kit | Sciex | Cat#C48231 |
| DNF-471 (15nt) RNA kit | Agilent | Cat#5191-6572 |
| Mouse Spleen Lymphocyte Isolation Kit | TBD | Cat#LTS1092PK |
| HiScript II One Step qRT-PCR SYBR Green Kit | Vazyme | Cat#Q221 |
| Quant-iT RiboGreen RNA Assay Kit | Invitrogen | Cat#R11490 |
