# Supplementary material for: A circular RNA vaccine induces durable and cross-protective immunity against Neisseria meningitidis serogroup B in mice
Source: PLoS Pathog. 2026 May 11;22(5):e1013741. doi: 10.1371/journal.ppat.1013741 (PMC13160355; doi:10.1371/journal.ppat.1013741)
Supplement: S2 Table — (DOCX) [file ppat.1013741.s003.docx]

**S2 Table.** Details of antibodies used in this study.

| **REAGENT or RESOURCE** | **SOURCE** | **IDENTIFIER** |
| --- | --- | --- |
| HRP-conjugated anti-6× His mouse McAb | Proteintech | Cat#HRP-66005 |
| Goat Anti-Mouse IgG (H+L) HRP | ELGbio | Cat#EA0102 |
| HRP-Goat Anti-Mouse IgG1 | Finetest | Cat#FNSA-0086 |
| HRP-Goat Anti-Mouse IgG2a | Finetest | Cat#FNSA-0087 |
| Goat pAb to Ms IgG2b (HRP) | Abcam | Cat#AB97250 |
| Goat pAb to Ms IgG3 (HRP) | Abcam | Cat#AB97260 |
| Purified Rat Anti-Mouse CD16/CD32 | BD Biosciences | Cat#553141 |
| FITC Hamster Anti-Mouse CD3e | BD Biosciences | Cat#553061 |
| APC Rat Anti-Mouse CD4 | BD Biosciences | Cat#553051 |
| PerCP-Cy5.5 Rat Anti-Mouse CD8α | BD Biosciences | Cat#551162 |
| PE Rat Anti-Mouse IFN-γ | BD Biosciences | Cat#554412 |
| BV421 Rat Anti-Mouse TNF | BD Biosciences | Cat#563387 |
| BV605 Rat Anti-Mouse IL-2 | BD Biosciences | Cat#563911 |
| Purified anti-mouse CD16/32 Antibody | BioLegend | Cat#101302 |
| BUV737 Mouse Anti-Human CD4 | BD Biosciences | Cat#612748 |
| BV421 Rat Anti-Mouse CD44 | BD Biosciences | Cat#563970 |
| FITC anti-mouse CD185 (CXCR5) Antibody | BioLegend | Cat#145520 |
| PE anti-mouse CD279 (PD-1) Antibody | BioLegend | Cat#135206 |
| Alexa Fluor 700 Rat Anti-Mouse CD45 | BD Biosciences | Cat#135206 |
| Brilliant Violet 650 anti-mouse/human CD45R/B220 Antibody | BioLegend | Cat#103241 |
| BV605 Rat Anti-Mouse CD38 | BD Biosciences | Cat#740361 |
| PE Hamster Anti-Mouse CD95 | BD Biosciences | Cat#561985 |
| PE/Cyanine7 anti-mouse/human GL7 Antigen (T and B cell Activation Marker) Antibody | BioLegend | Cat#144620 |
| Brilliant Violet 421 anti-mouse IgG1 Antibody | BioLegend | Cat#406616 |
| Anti-Mouse CD8α in vivo antibody, Clone 2.43 | InvivoCrown | IV0104 |
| Rat IgG2b in vivo Isotype control | InvivoCrown | IV0106 |
