# Supplementary material for: A circular RNA vaccine induces durable and cross-protective immunity against Neisseria meningitidis serogroup B in mice
Source: PLoS Pathog. 2026 May 11;22(5):e1013741. doi: 10.1371/journal.ppat.1013741 (PMC13160355; doi:10.1371/journal.ppat.1013741)
Supplement: S3 Table — (DOCX) [file ppat.1013741.s004.docx]

**S3 Table.** Detail primer sequences used in this study.

| **GENE** | **DIRECTION** | **SEQUENCE (5’-3’)** |
| --- | --- | --- |
| GAPDH | Forward | AGCTCATTTCCTGGTATGACA |
|  | Reverse | AGGGGAGATTCAGTGTGGTG |
| RIG-1 | Forward | TGTGGGCAATGTCATCAAAA |
|  | Reverse | GAAGCACTTGCTACCTCTTGC |
| IL-6 | Forward | AGCCACTCACCTCTTCAGAAC |
|  | Reverse | GCCTCTTTGCTGCTTTCACAC |
| TNF-α | Forward | CTGCACTTTGGAGTGATCG |
|  | Reverse | GGGTTCGAGAAGATGATCTGAC |
| IFN-α | Forward | ACATCTGGTCCAACATGAAAAC |
|  | Reverse | GGTCATAGTTATAGCAGGGGTG |
| IFN-β | Forward | TCTAGCACTGGCTGGAATGAG |
|  | Reverse | GTTTCGGAGGTAACCTGTAAG |
| CCL-5 | Forward | CACAGGTACCATGAAGGTCTC |
|  | Reverse | GGTGTCCGAGGAATATGGG |
| CircVB16T13 | Forward | AGTTGTCCGACGCGGACA |
|  | Reverse | GTGTGCCACGCTGACTGTT |
