# Supplementary material for: A circular RNA vaccine induces durable and cross-protective immunity against Neisseria meningitidis serogroup B in mice
Source: PLoS Pathog. 2026 May 11;22(5):e1013741. doi: 10.1371/journal.ppat.1013741 (PMC13160355; doi:10.1371/journal.ppat.1013741)
Supplement: S4 Table — TAL reagent sensitivity was λ = 0.25 EU/mL. Endotoxin-free water (0 EU/mL) and an endotoxin standard (0.5 EU/mL) were included as the negative and positive controls, respectively. A positive product control (PPC) was prepared by spiking the 1:2 diluted VB16T13 sample with endotoxin to a final concentration of 0.25 EU/mL. VB16T13 was tested at the indicated dilutions (1:2-1:8192). Results are shown as gelation (+) or no gelation (−) in two technical replicates (Rep 1-2). Based on a positive result at 1:2 and a negative result at 1:16, the endotoxin level in the stock formulation was reported as <4 EU/mL (i.e., < 0.4 EU per 100-µL dose). (DOCX) [file ppat.1013741.s005.docx]

**S4 Table. Endotoxin testing of *E. coli*-expressed VB16T13 by the gel-clot TAL assay.**

| **Samples** | **Concentration/Dilution** | **Rep 1** | **Rep 2** |
| --- | --- | --- | --- |
| Negative Control | 0 EU/mL | - | - |
| Positive Control | 0.5 EU/mL | + | + |
| Positive Product Control | 0.25 EU/mL  1:2 | + | + |
| *E.coli*-expressed VB16T13 | 1:2 | + | + |
|  | 1:16 | - | - |
|  | 1:128 | - | - |
|  | 1:1024 | - | - |
|  | 1:8192 | - | - |

TAL reagent sensitivity was λ = 0.25 EU/mL. Endotoxin-free water (0 EU/mL) and an endotoxin standard (0.5 EU/mL) were included as the negative and positive controls, respectively. A positive product control (PPC) was prepared by spiking the 1:2 diluted VB16T13 sample with endotoxin to a final concentration of 0.25 EU/mL. VB16T13 was tested at the indicated dilutions (1:2-1:8192). Results are shown as gelation (+) or no gelation (−) in two technical replicates (Rep 1-2). Based on a positive result at 1:2 and a negative result at 1:16, the endotoxin level in the stock formulation was reported as <4 EU/mL (i.e., <0.4 EU per 100-µL dose).
