# Supplementary material for: A circular RNA vaccine induces durable and cross-protective immunity against Neisseria meningitidis serogroup B in mice
Source: PLoS Pathog. 2026 May 11;22(5):e1013741. doi: 10.1371/journal.ppat.1013741 (PMC13160355; doi:10.1371/journal.ppat.1013741)
Supplement: S5 Table — The mixture of 4B3 and 1E6 were serially diluted, and subjected to serum bactericidal assay (SBA) against MC58, 50% bactericidal activity was observed at a concentration of 7.81 μg/mL. (DOCX) [file ppat.1013741.s006.docx]

**S5 Table. Bactericidal activity by mAbs 4B3 and 1E6.**

| **Antibody 4B3 with 1E6** | **Antibody conc. (μg/mL)** | | | | | | **Inactivated**  **Complement** |
| --- | --- | --- | --- | --- | --- | --- | --- |
|  | **125** | **62.5** | **31.25** | **15.63** | **7.81** | **3.91** |  |
| clone_ Rep 1 | 16 | 20 | 11 | 47 | 68 | 103 | 138 |
| clone_ Rep 2 | 13 | 11 | 16 | 35 | 64 | 104 | 148 |
| clone_ Rep 3 | 17 | 16 | 23 | 35 | 74 | 107 | 134 |
| clone_average | 15 | 16 | 17 | 39 | 69 | 105 | 140 |
| Bactericidal rate | 89.05% | 88.81% | 88.10% | 72.14% | 50.95% | 25.24% | - |

The mixture of 4B3 and 1E6 were serially diluted, and subjected to serum bactericidal assay (SBA) against MC58, 50% bactericidal activity was observed at a concentration of 7.81 μg/mL.
