# Supplementary figures and images for: A circular RNA vaccine induces durable and cross-protective immunity against Neisseria meningitidis serogroup B in mice
Source: PLoS Pathog. 2026 May 11;22(5):e1013741. doi: 10.1371/journal.ppat.1013741 (PMC13160355; doi:10.1371/journal.ppat.1013741)

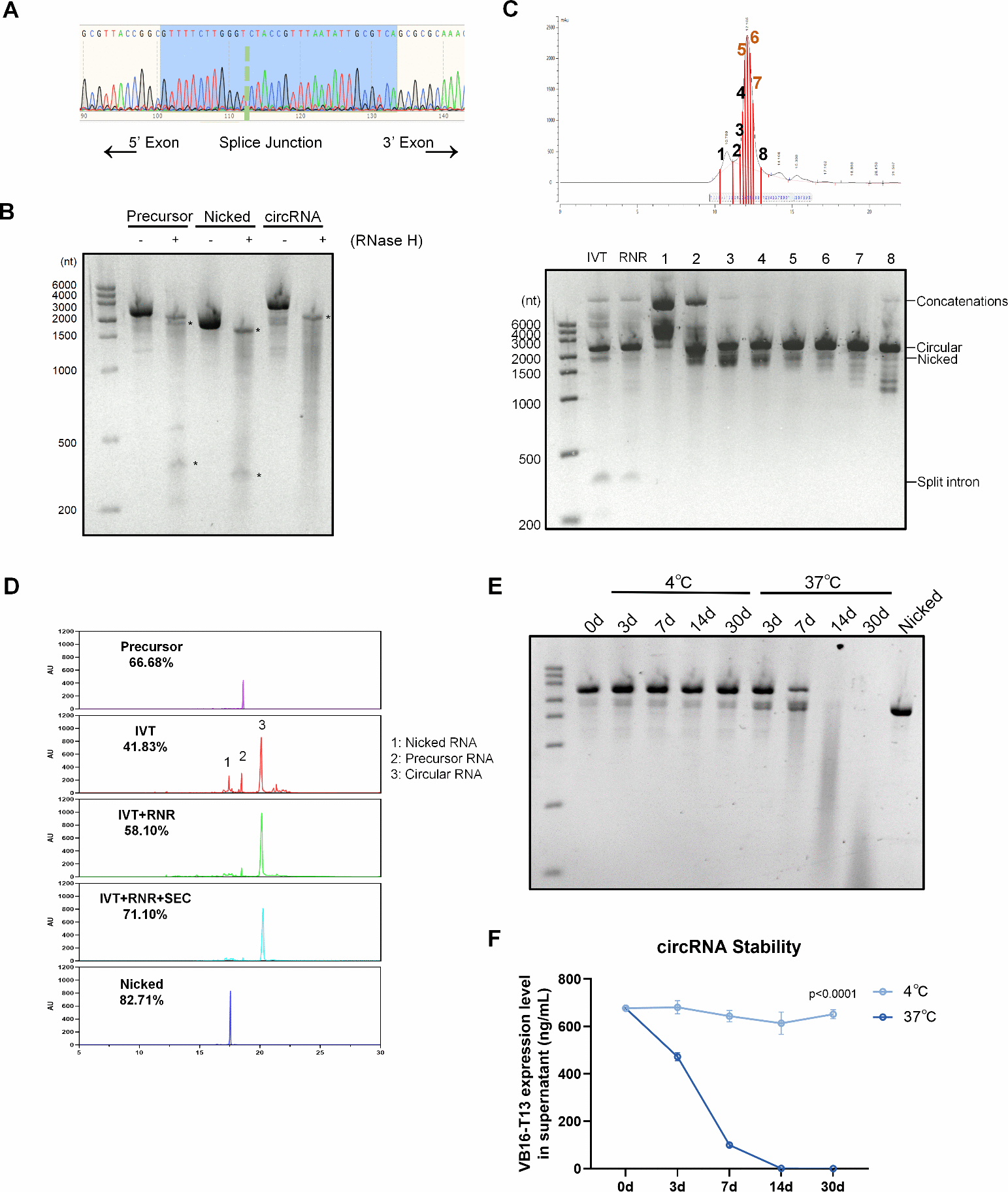

Supplement: S1 Fig — (A) Sanger sequencing spanning the backsplice junction (dotted line). Reverse primer confirmed circularization fidelity. (B) E-gel analysis of RNase H digestion products of precursor RNA, nicked control, and circVB16T13. * indicated the RNase H cleavage products. (C) Two-step purification workflow: Upper: Size-exclusion chromatography (SEC) profile (red line area presented the collected fractions). Lower: E-gel showing the contents of SEC fractions. (D) Capillary gel electrophoresis with laser-induced fluorescence detection (CGE-LIF) assessment of purification efficiency. Precursor: linear IVT product; IVT: in vitro transcription (circularization was concurrently performed during IVT); RNR: RNase R-treated sample; SEC: size-exclusion chromatography fraction; Nicked: linearized RNA control with the same nucleotide sequence as circVB16T13. (E, F) Thermal stability assessment. (E) E-gel of circVB16T13 stocked at 4°C/37°C for 0–30 days. (F) Quantification of VB16T13 expression from circRNA by ELISA after storage at the indicated temperatures. Comparisons among more than two groups were conducted by Two-way ANOVA comparison tests. (TIF) [file ppat.1013741.s007.tif]

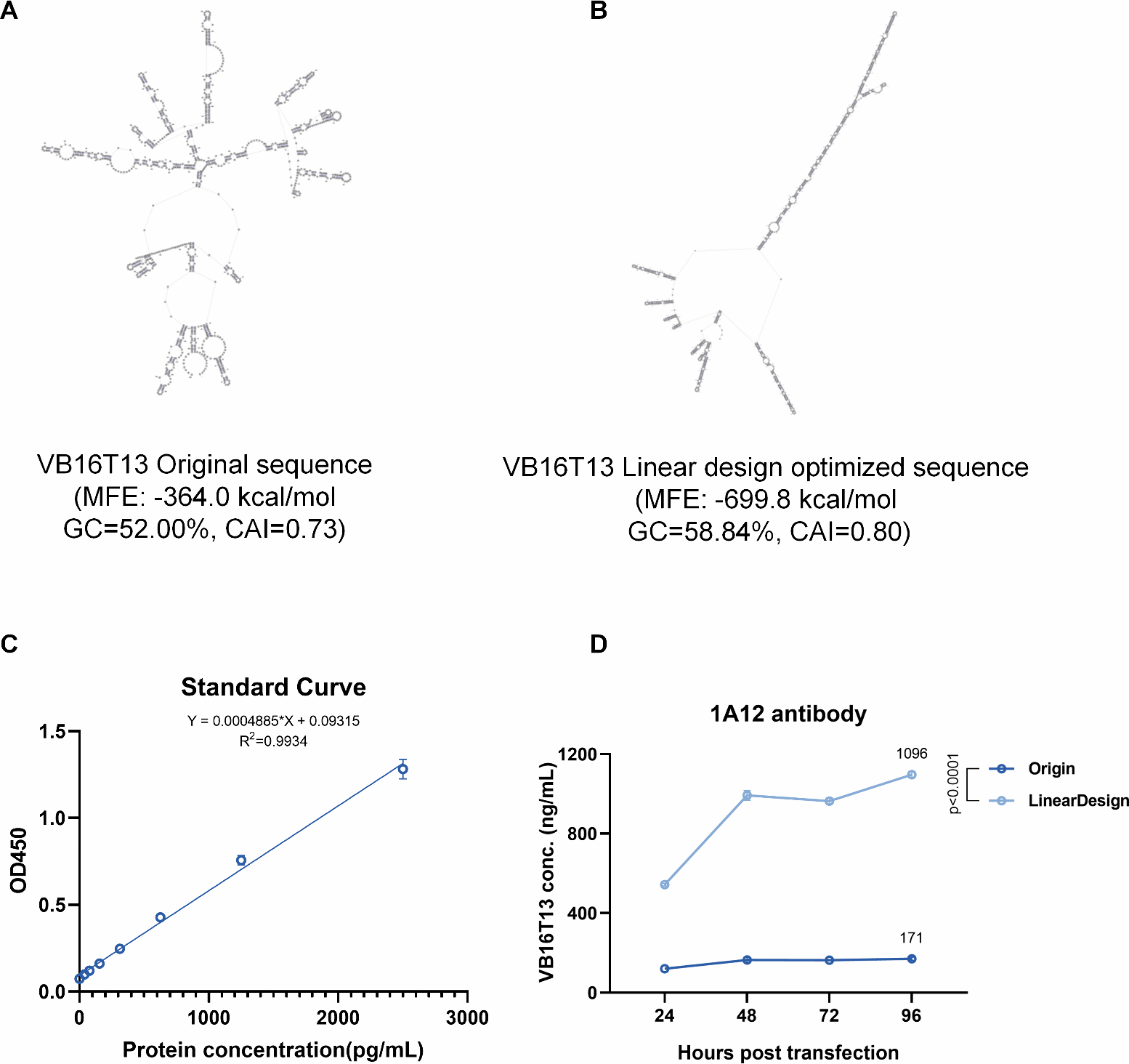

Supplement: S2 Fig — (A, B) circVB16T13 secondary structures predicted by ViennaRNA Package 2.0. (A) Native circVB16T13 sequence (∆G = -364.0 kcal/mol). (B) LinearDesign-optimized sequence (∆G = -699.8 kcal/mol). (C) VB16T13 standard curve for quantitative ELISA (R² = 0.9934). (D) Antigen levels in cell culture supernatants at 24, 48, 72, and 96 h after transfection with 1 μg circRNA. The mean protein concentrations at 96 h are indicated in the figure. Comparisons among more than two groups were conducted by Two-way ANOVA comparison tests. (TIF) [file ppat.1013741.s008.tif]

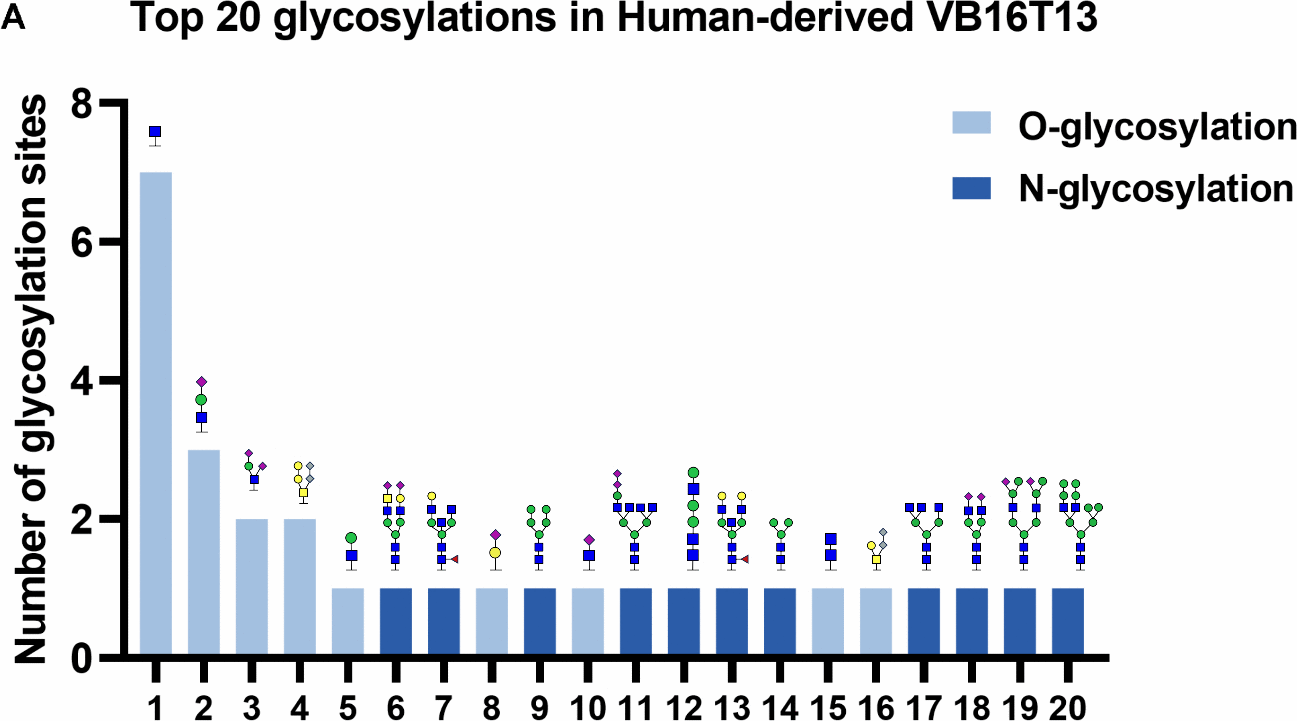

Supplement: S3 Fig — (A) Top 20 glycosylation modifications in human-derived VB16T13. (TIF) [file ppat.1013741.s009.tif]

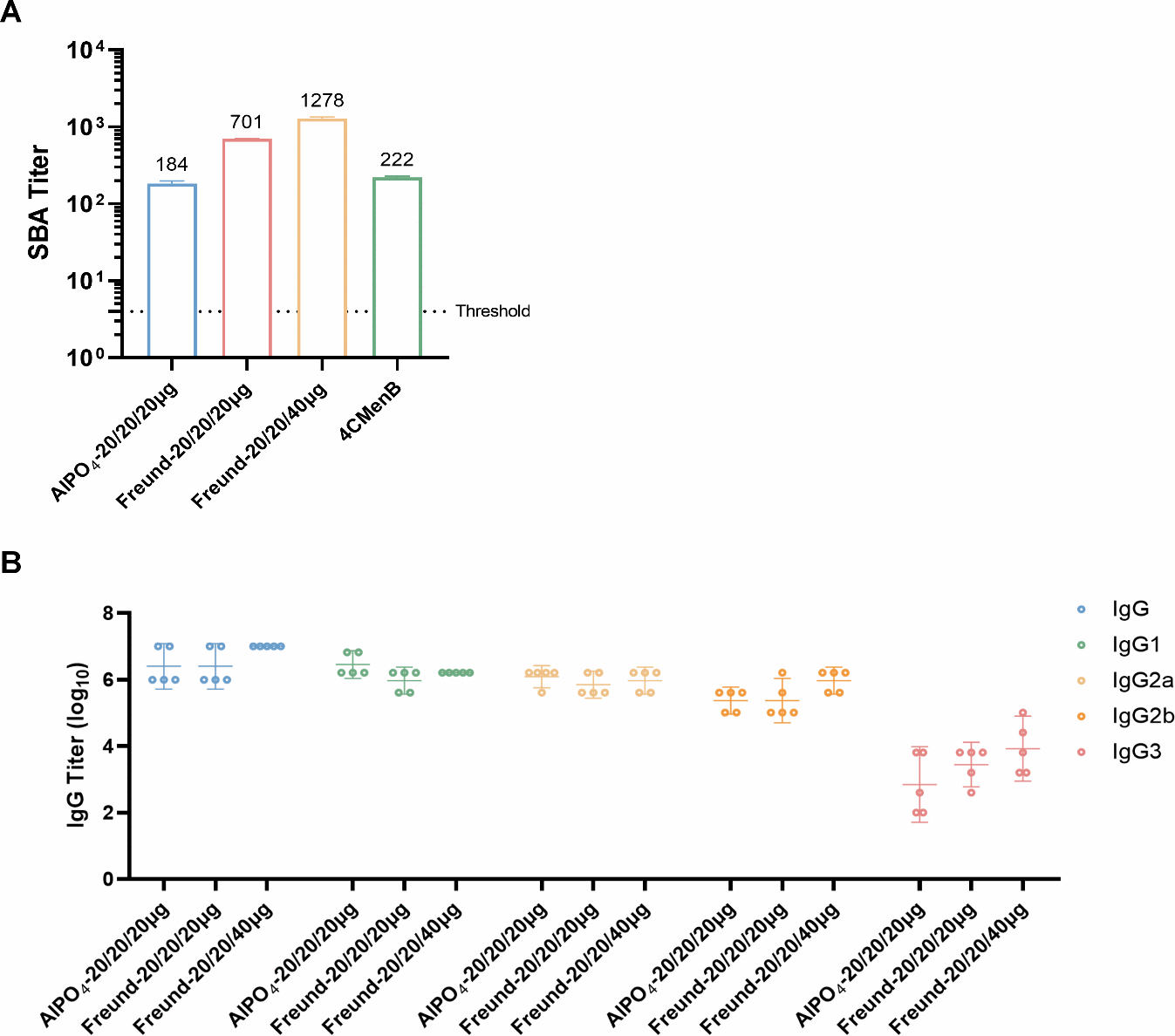

Supplement: S4 Fig — (A) hSBA titers of pooled serum from BALB/c mice after three immunizations with VB16T13 formulated with different adjuvants, with 4CMenB included as a comparator (n = 5 per group). (B) VB16T13-specific IgG titers in individual mouse serum after three immunizations with VB16T13 formulated with different adjuvants or doses (n = 5 per group). (TIF) [file ppat.1013741.s010.tif]

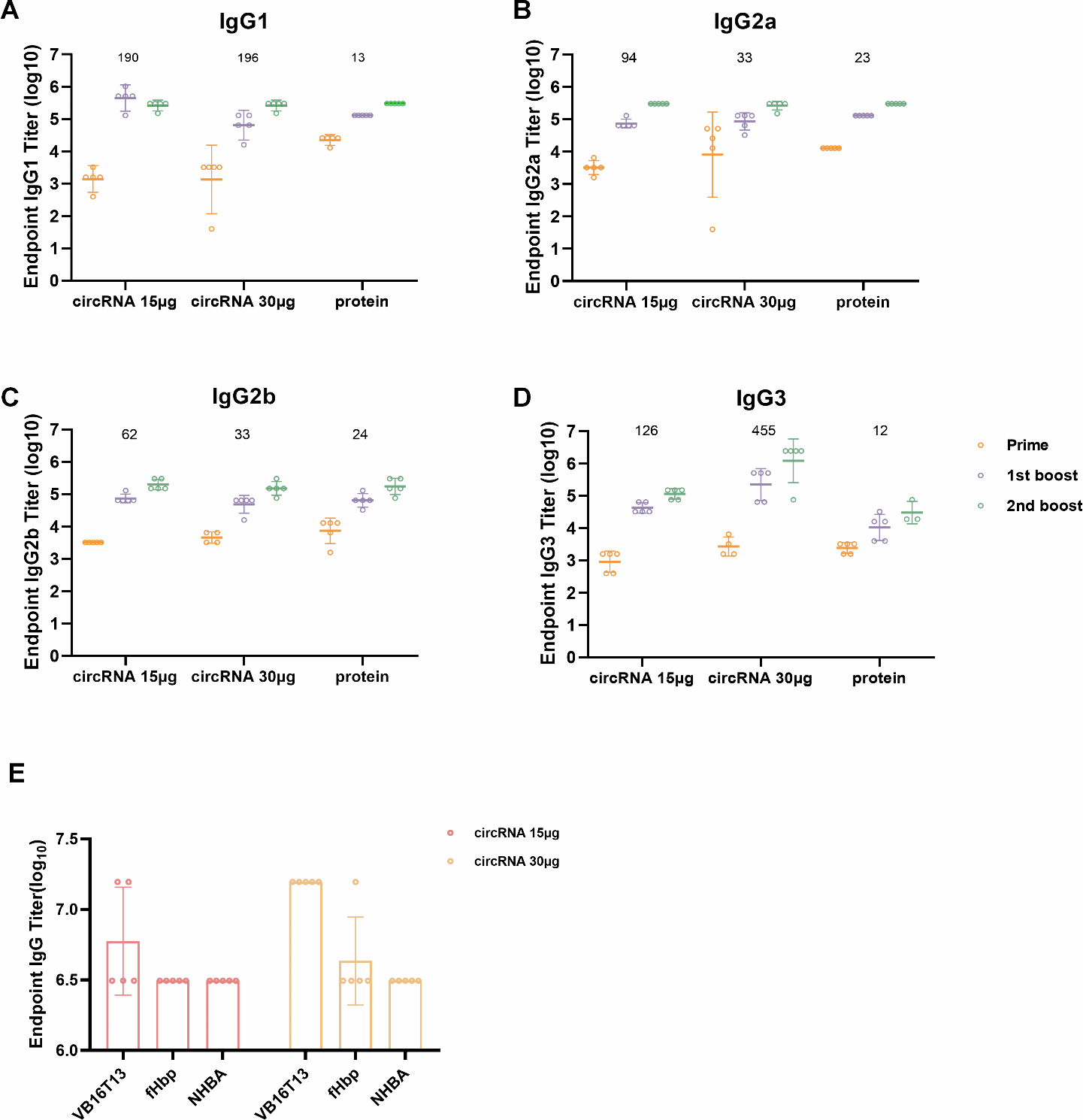

Supplement: S5 Fig — (A-D) Antigen-specific IgG subclass kinetics at 2 weeks after prime, 1st boost, and 2nd boost administration. (A) IgG1, (B) IgG2a, (C) IgG2b, and (D) IgG3. The limit of detection in prime = 2.6, in 1st boost = 3.6, and in 2nd boost = 4.28. The fold changes in GMT between 2nd boost and prime in each immunized group were labeled above. (E) Cross-reactivity profiling by endpoint ELISA. Sera IgG titers from circVB16T13-immunized mice recognized with VB16T13, fHbp, and NHBA. (TIF) [file ppat.1013741.s011.tif]

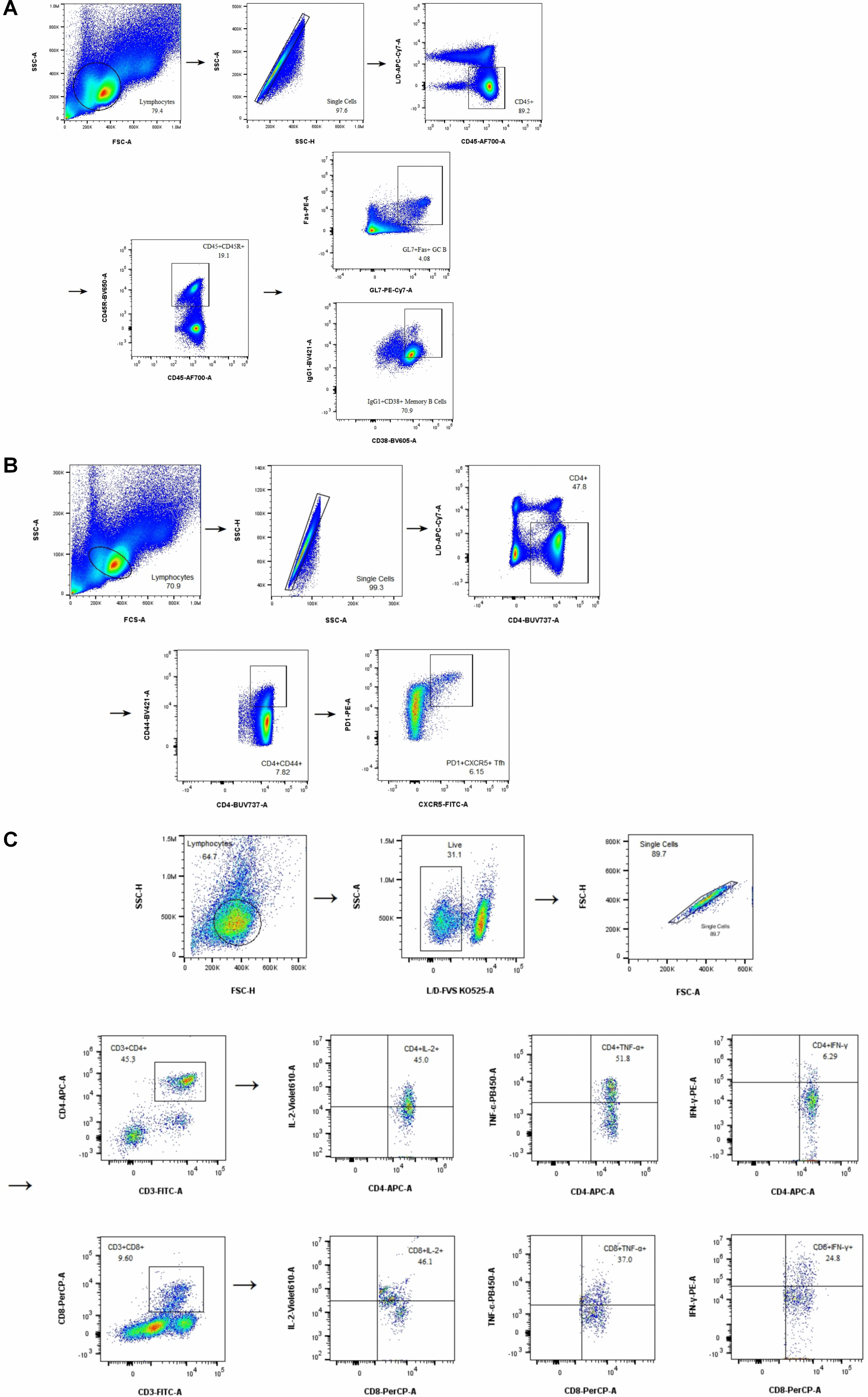

Supplement: S6 Fig — (A) Gating strategy for GC B cells and memory B cells in the ILNs. GC B cell designation criteria: CD45 + CD45R+GL7 + Fas + . Memory B cell designation criteria: CD45 + CD45R+IgG1 + CD38 + . (B) Gating strategy for Tfh cells in the ILNs. Tfh cell designation criteria: CD4 + CD44 + PD-1 + CXCR5 + . (C) Gating strategy for CD4 + T and CD8 + T cells that secrete IFN-γ, TNFα, or IL-2 in the spleens. (TIF) [file ppat.1013741.s012.tif]

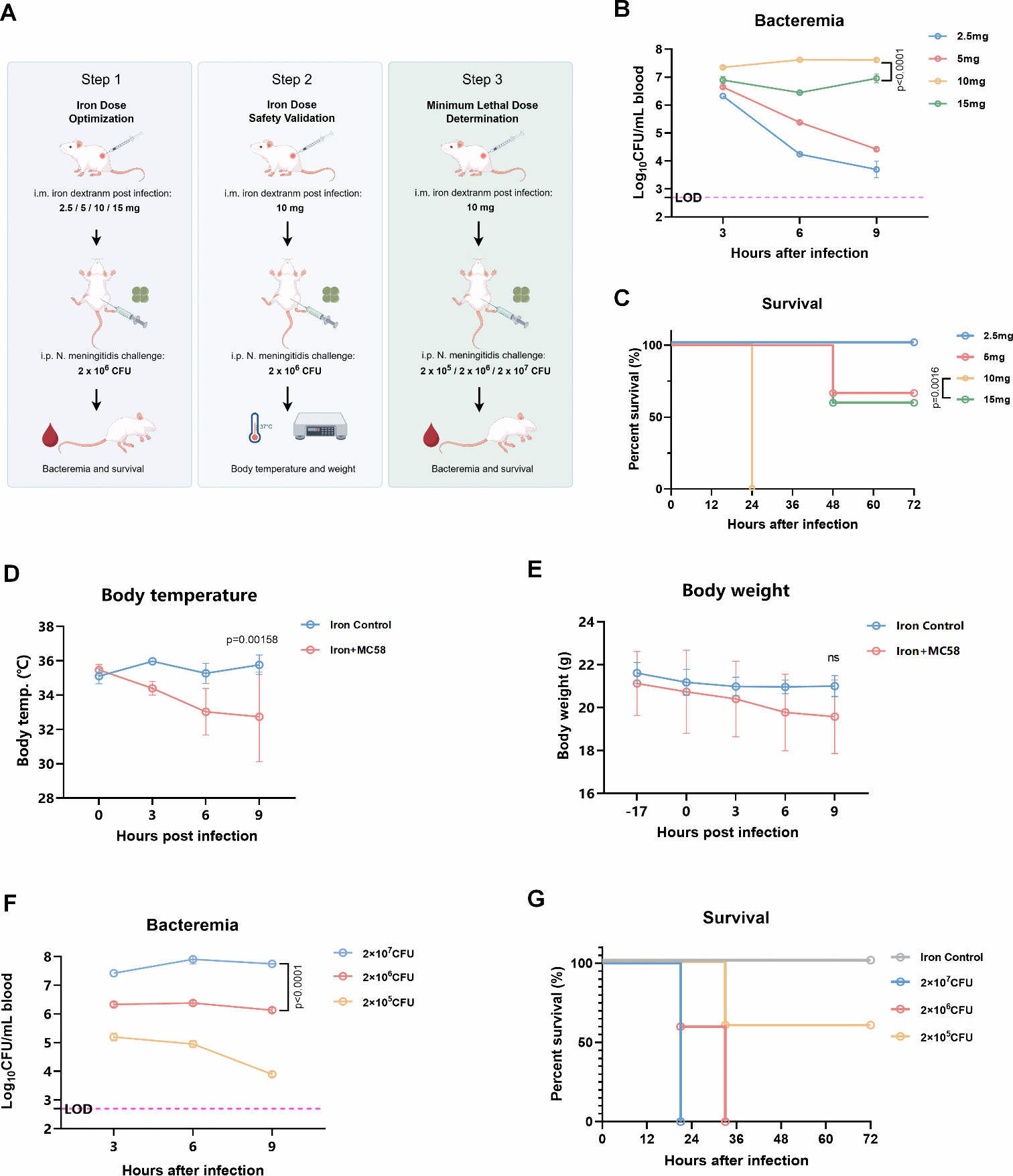

Supplement: S7 Fig — (A) Schematic of challenge model construction procedure. This figure was created using Figdraw (www.figdraw.com) with permission. (B) Quantitative bacteremia kinetics in mice pretreated intramuscularly with 2.5, 5, 10, or 15 mg/mouse. Blood bacterial loads measured at 1-, 3-, 6-, and 9-hour post-infection (hpi). Dashed line indicates detection limit (500 CFU/mL). (C) Survival curves of mice pretreated with indicated doses (2.5-15 mg/mouse). (D, E) Clinical sign comparison between MC58-infected and BHI iron control groups: (D) Body temperature measurements at 0, 3, 6, and 9 hpi. (E) Body weight measurements at 17 h before (-17 h), and 0, 3, 6, 9 hpi. (F) Quantitative bacteremia kinetics at challenge doses of 2 × 10⁵-2 × 10⁷ CFU, measured at 3, 6, and 9 hpi. Dashed line indicates detection limit (500 CFU/mL). (G) Survival rates over 72 hours post-infection at challenge doses of 2 × 10⁵-2 × 10⁷ CFU. Comparisons among more than two groups were conducted by Two-way ANOVA comparison tests. Comparisons of the survival data were conducted by Log-rank (Mantel-Cox) tests. (TIF) [file ppat.1013741.s013.tif]

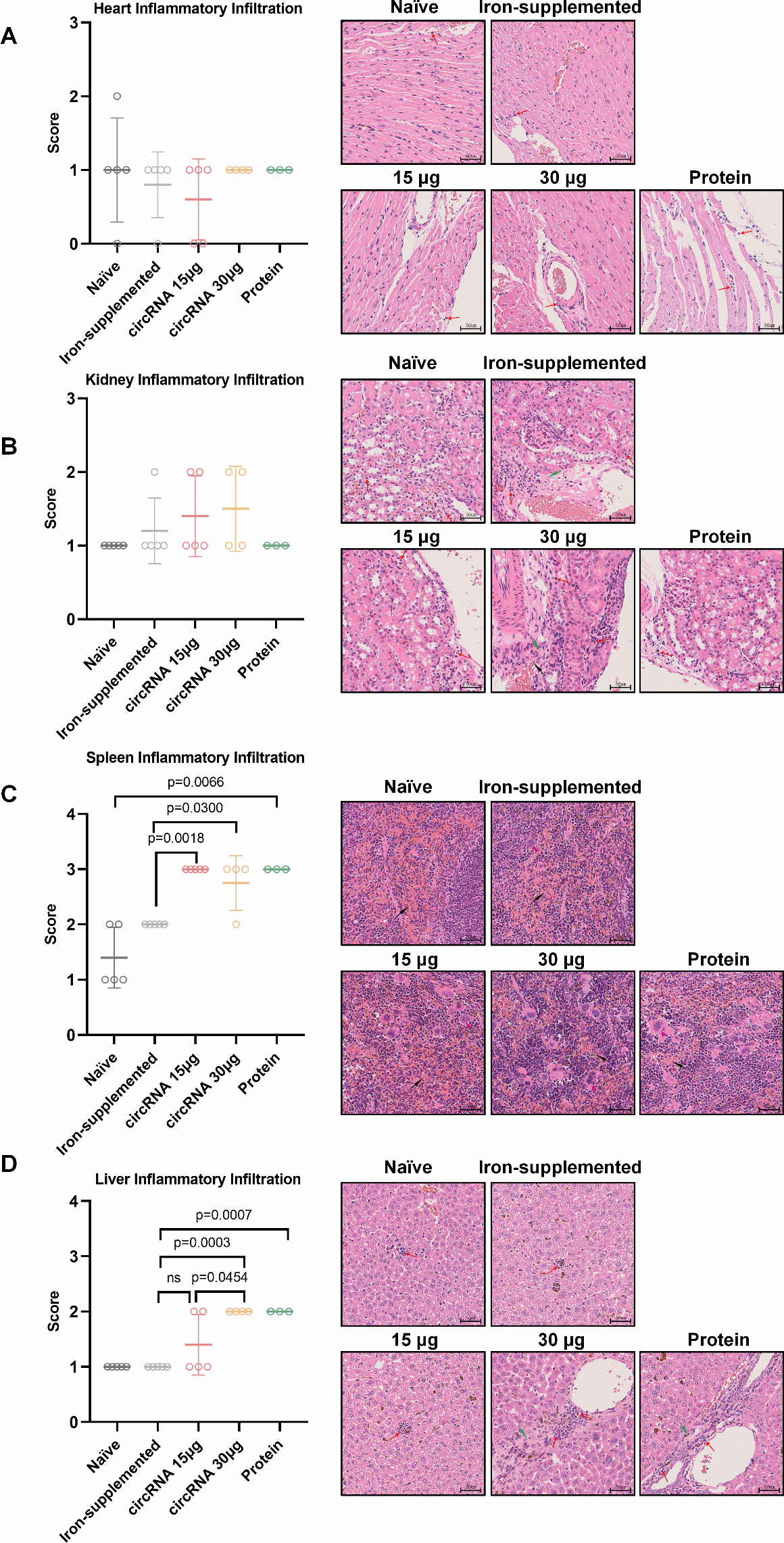

Supplement: S8 Fig — (A) Representative heart sections. Red arrows indicate inflammatory cells. (B) Representative kidney sections. Black arrows indicate hemorrhage, red arrows indicate inflammatory cells, and green arrows indicate fibrosis. (C) Representative spleen sections. Black arrows indicate hyperemia, and red arrows indicate multinucleated giant cells. (D) Representative liver sections. Red arrows indicate inflammatory cells, and green arrows indicate fibrosis. Scale bars: 50 μm. Comparisons among more than two groups were performed using one-way ANOVA. (TIF) [file ppat.1013741.s014.tif]

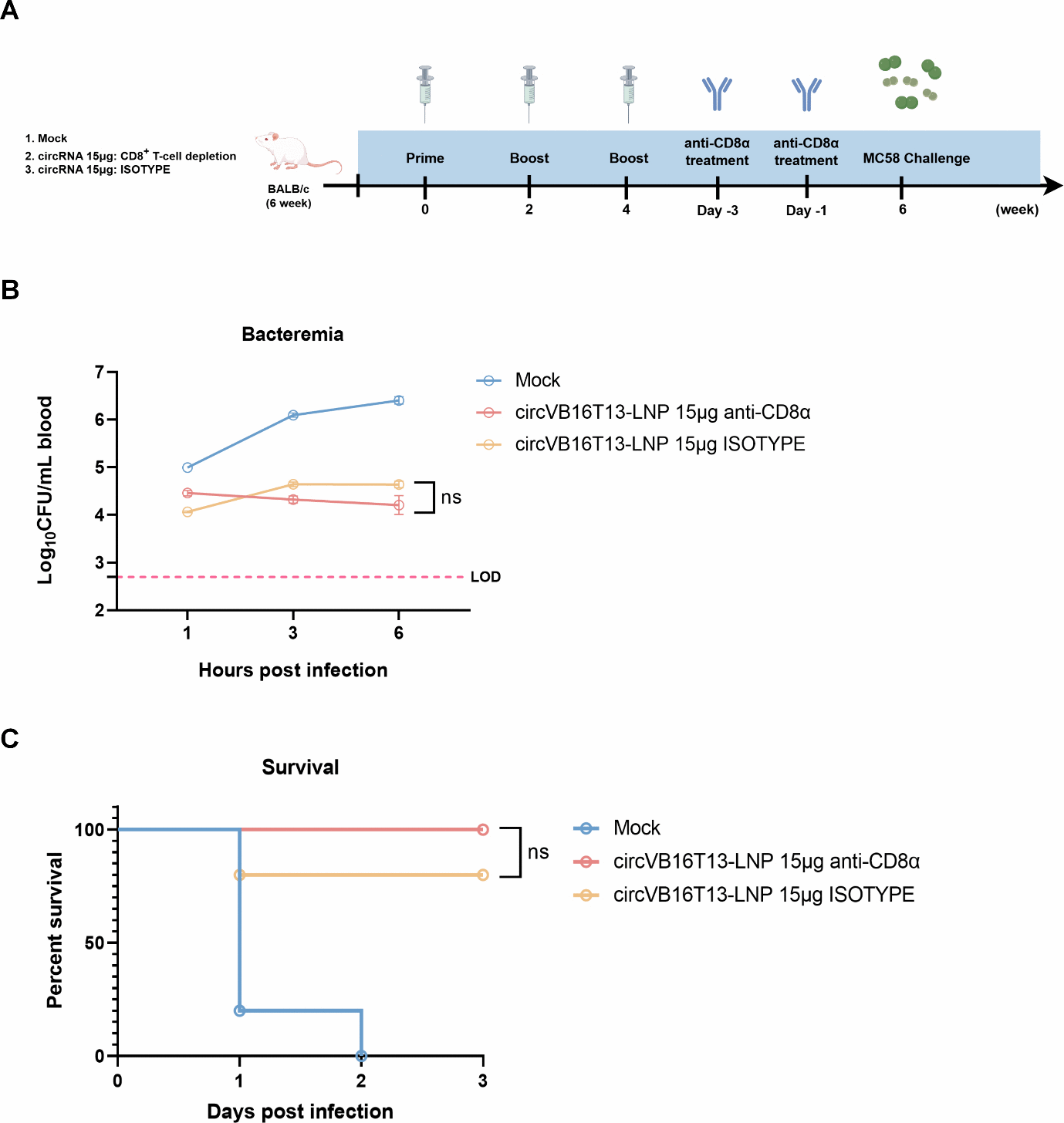

Supplement: S9 Fig — (A) Experimental design for MC58 challenge. BALB/c mice (n = 5 per group) were challenged intraperitoneally with 1 × 10⁷ CFU at 14 days after the final immunization. Anti-CD8α antibody (200 μg per mouse) was administered intraperitoneally on days -3 and -1 before challenge. This figure was created using Figdraw (www.figdraw.com) with permission. (B) Kinetics of bacteremia in pooled whole blood after challenge. Bacterial loads were quantified at 1, 3, and 6 h post infection; the dashed line indicates the limit of detection (500 CFU/mL). (C) Survival of mice monitored for 3 days after challenge. (TIF) [file ppat.1013741.s015.tif]

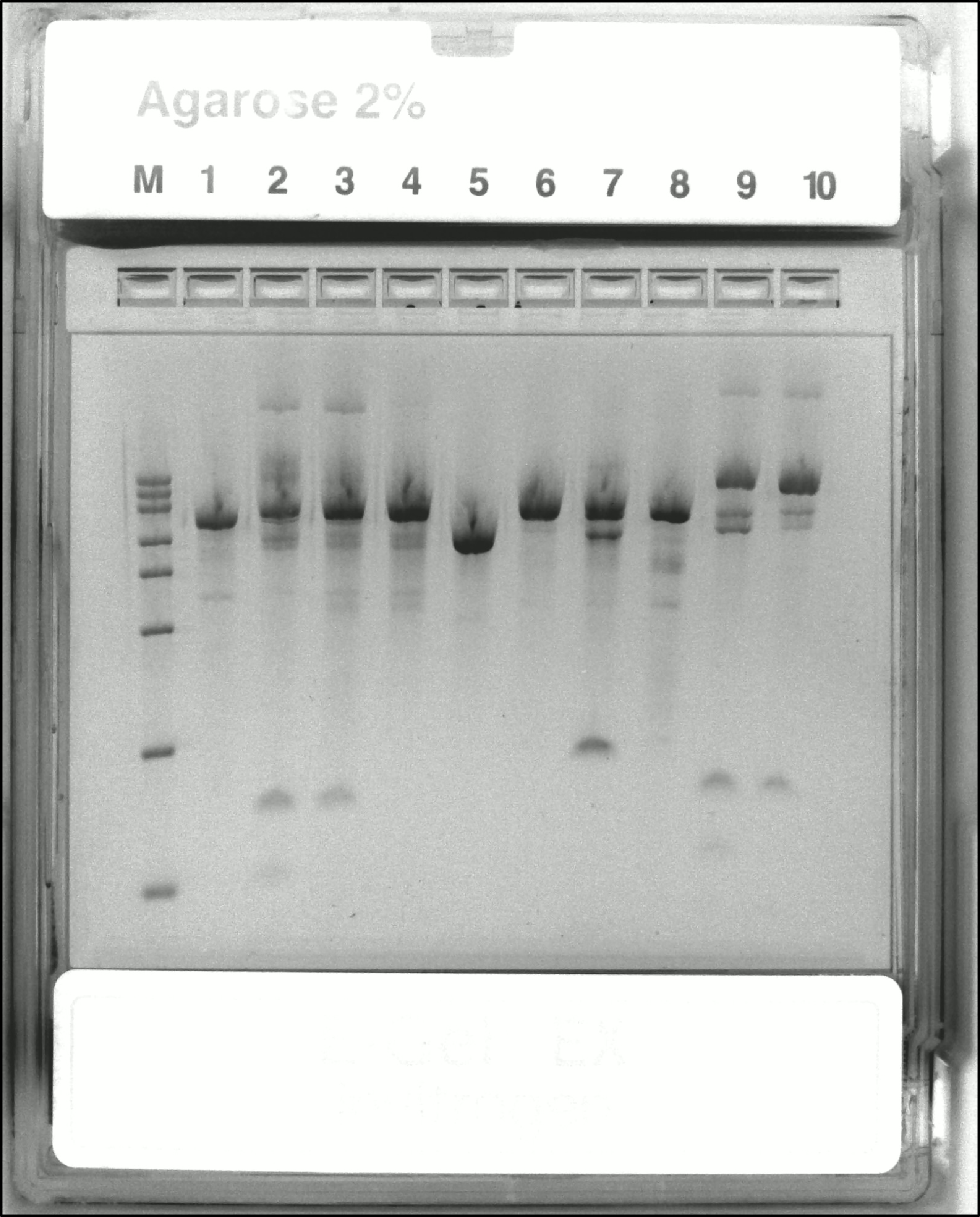

Supplement: S1 Data — Images files. SPR PDF files. CE5200 PDF file. S1D Fig report. S1A Fig sequencing file. (ZIP) [file ppat.1013741.s016.zip › Raw data/Images files/Fig 1B/Fig 1B.tif]

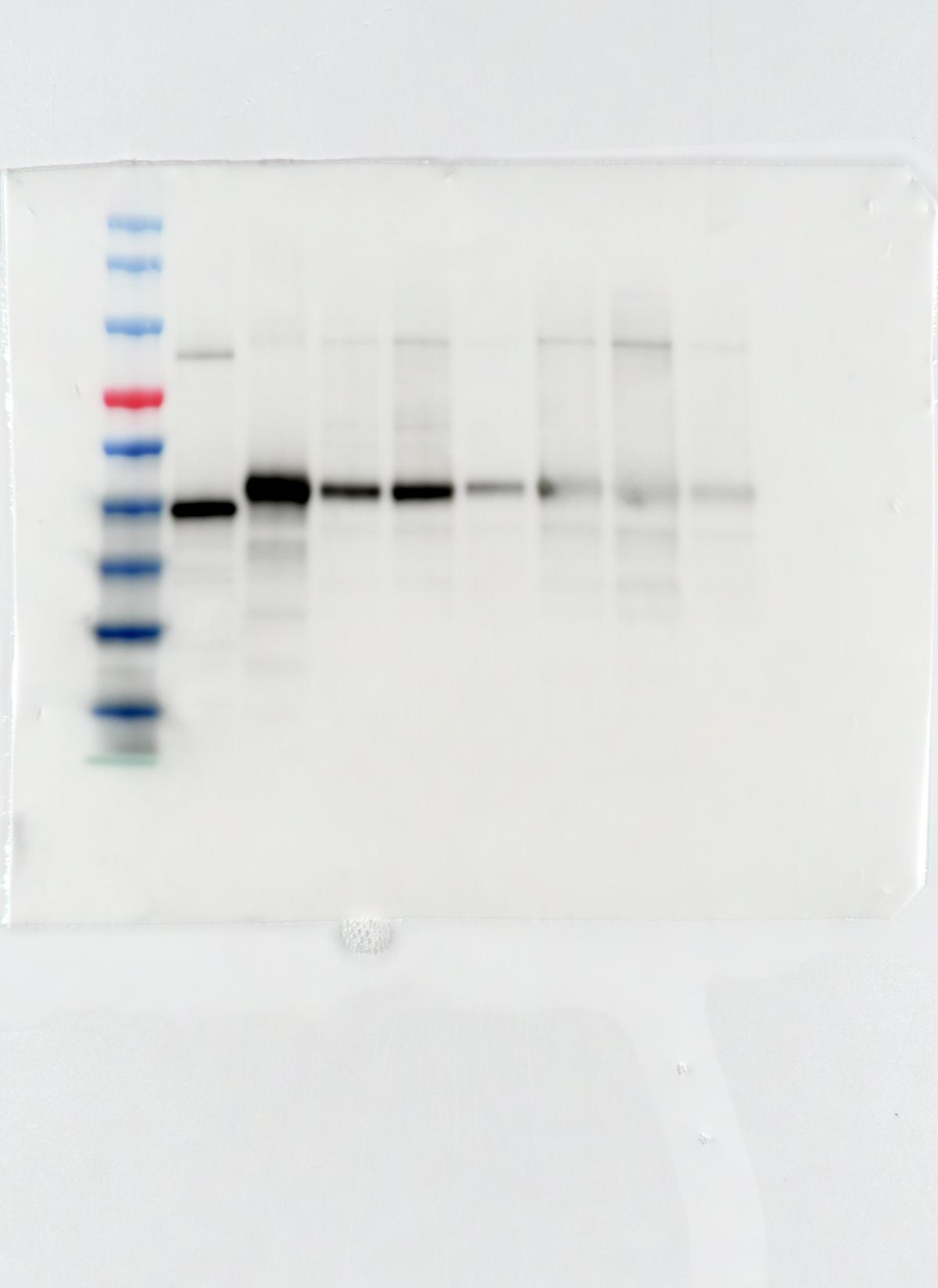

Supplement: S1 Data — Images files. SPR PDF files. CE5200 PDF file. S1D Fig report. S1A Fig sequencing file. (ZIP) [file ppat.1013741.s016.zip › Raw data/Images files/Fig 2A/Fig 2A.tif]

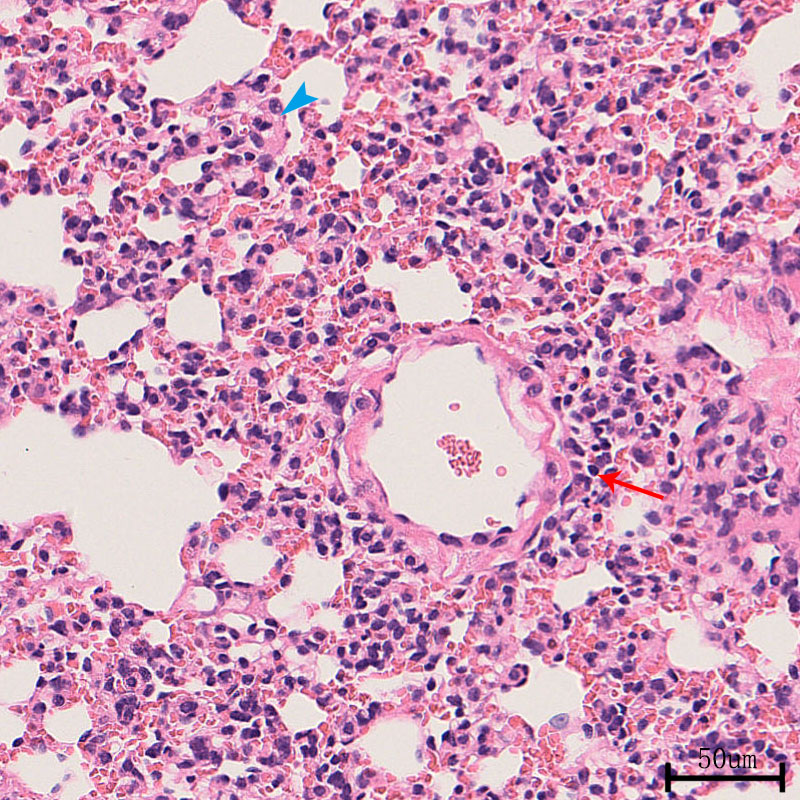

Supplement: S1 Data — Images files. SPR PDF files. CE5200 PDF file. S1D Fig report. S1A Fig sequencing file. (ZIP) [file ppat.1013741.s016.zip › Raw data/Images files/Fig 4D/circRNA 15μg.jpg]

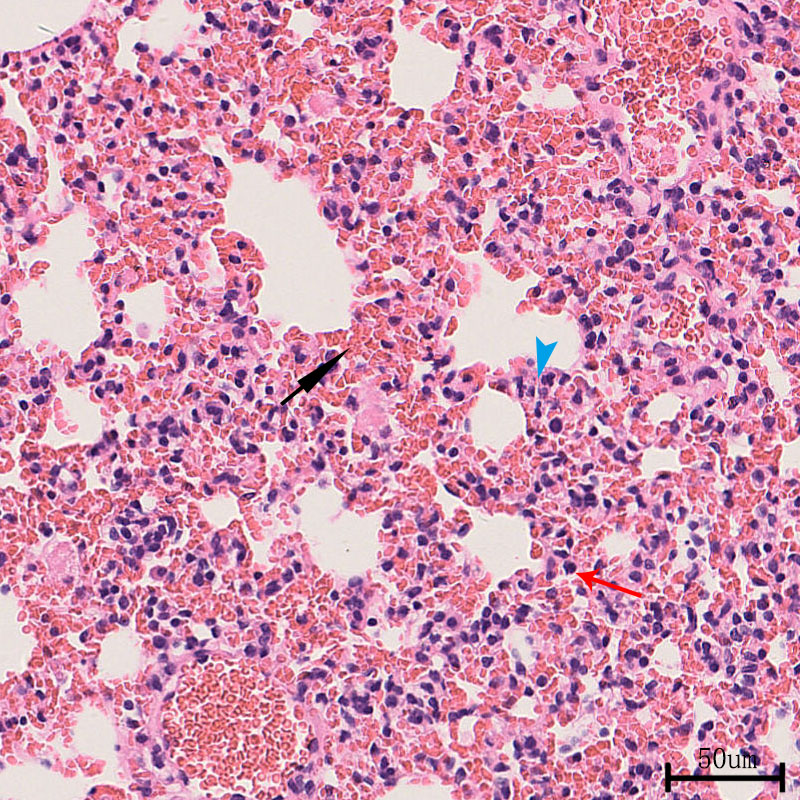

Supplement: S1 Data — Images files. SPR PDF files. CE5200 PDF file. S1D Fig report. S1A Fig sequencing file. (ZIP) [file ppat.1013741.s016.zip › Raw data/Images files/Fig 4D/circRNA 30μg.jpg]

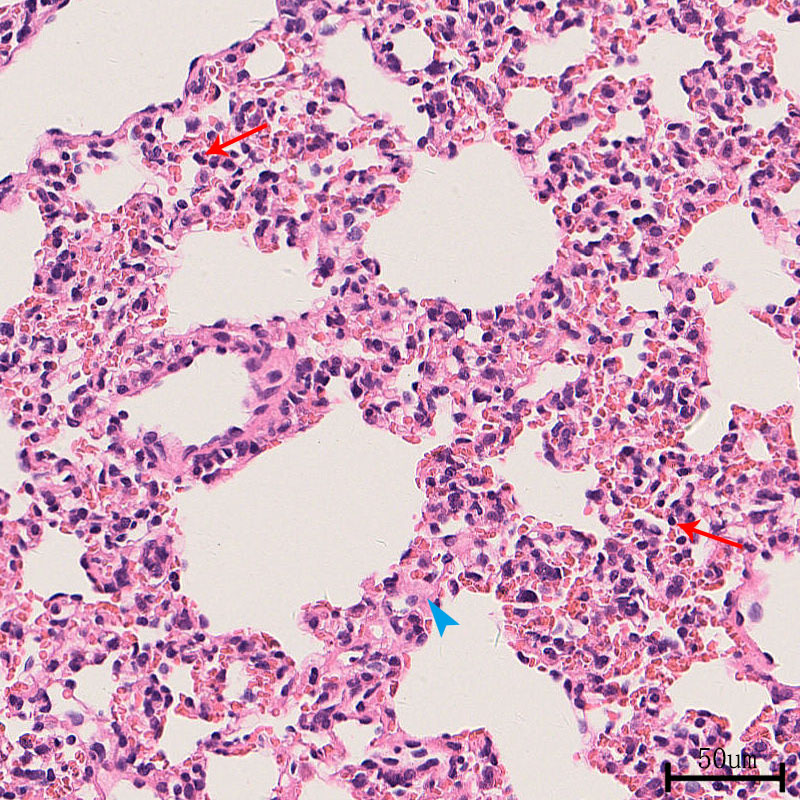

Supplement: S1 Data — Images files. SPR PDF files. CE5200 PDF file. S1D Fig report. S1A Fig sequencing file. (ZIP) [file ppat.1013741.s016.zip › Raw data/Images files/Fig 4D/Iron-supplemented.jpg]

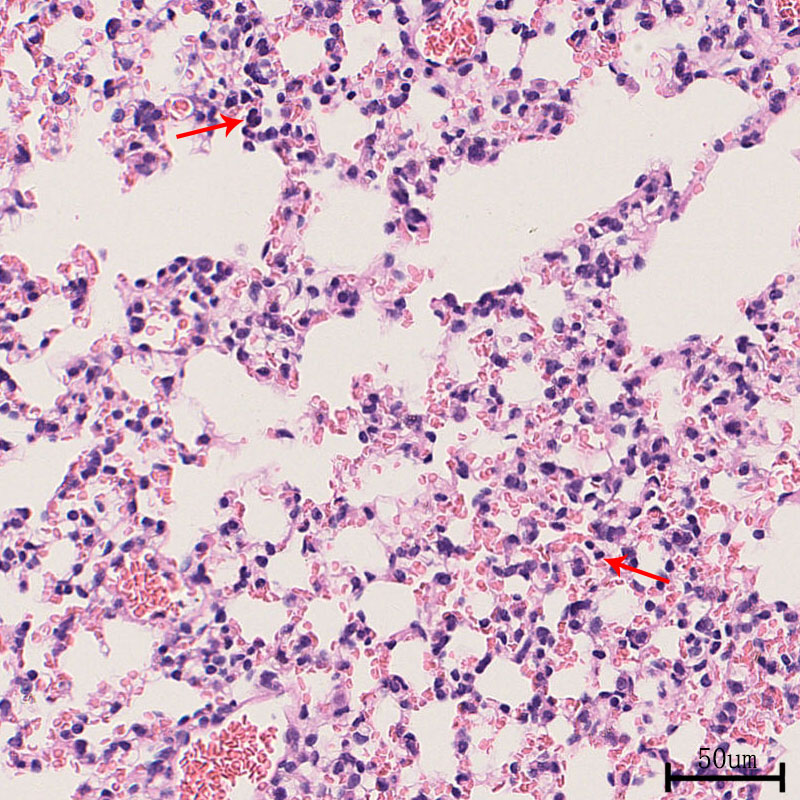

Supplement: S1 Data — Images files. SPR PDF files. CE5200 PDF file. S1D Fig report. S1A Fig sequencing file. (ZIP) [file ppat.1013741.s016.zip › Raw data/Images files/Fig 4D/Naïve.jpg]

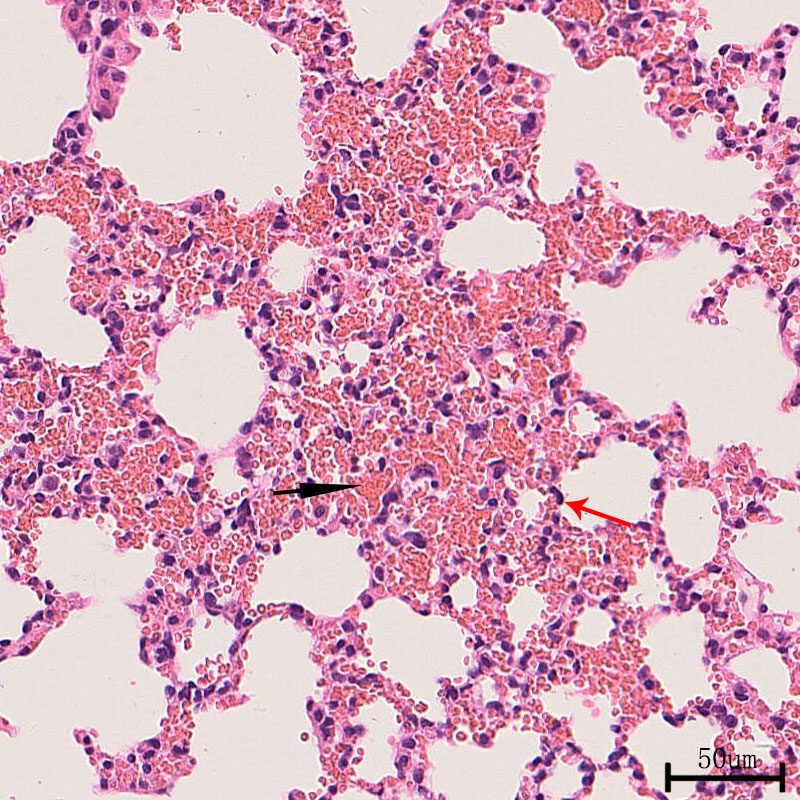

Supplement: S1 Data — Images files. SPR PDF files. CE5200 PDF file. S1D Fig report. S1A Fig sequencing file. (ZIP) [file ppat.1013741.s016.zip › Raw data/Images files/Fig 4D/Protein.jpg]

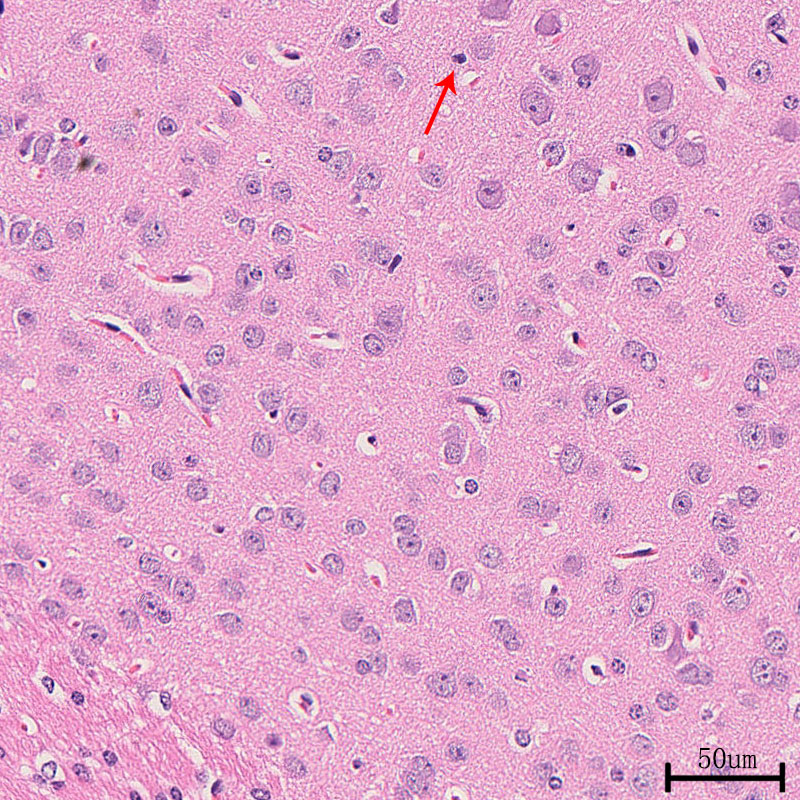

Supplement: S1 Data — Images files. SPR PDF files. CE5200 PDF file. S1D Fig report. S1A Fig sequencing file. (ZIP) [file ppat.1013741.s016.zip › Raw data/Images files/Fig 4E/circRNA 15μg.jpg]

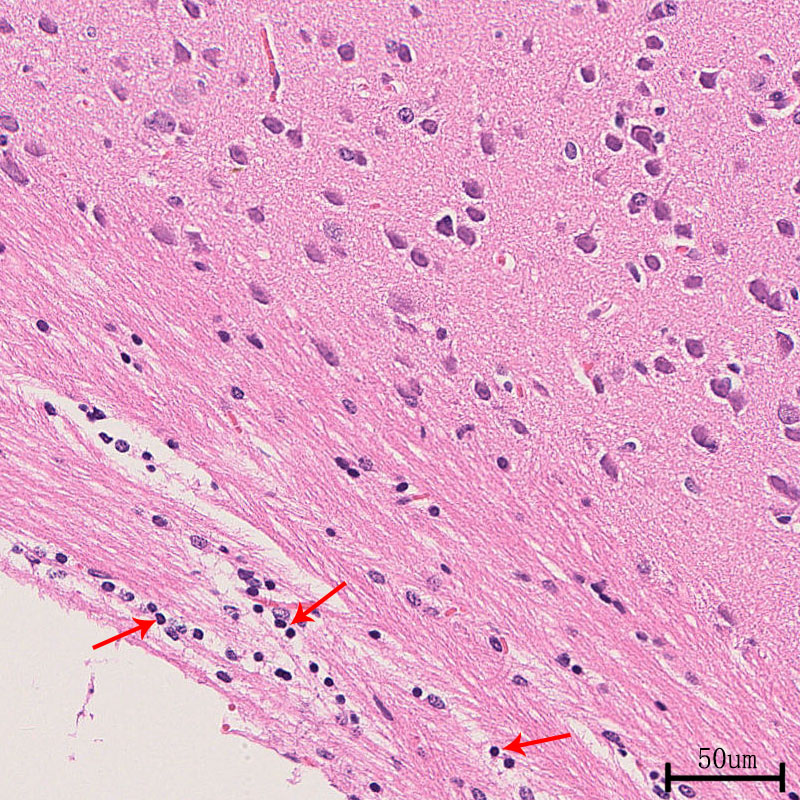

Supplement: S1 Data — Images files. SPR PDF files. CE5200 PDF file. S1D Fig report. S1A Fig sequencing file. (ZIP) [file ppat.1013741.s016.zip › Raw data/Images files/Fig 4E/circRNA 30μg.jpg]

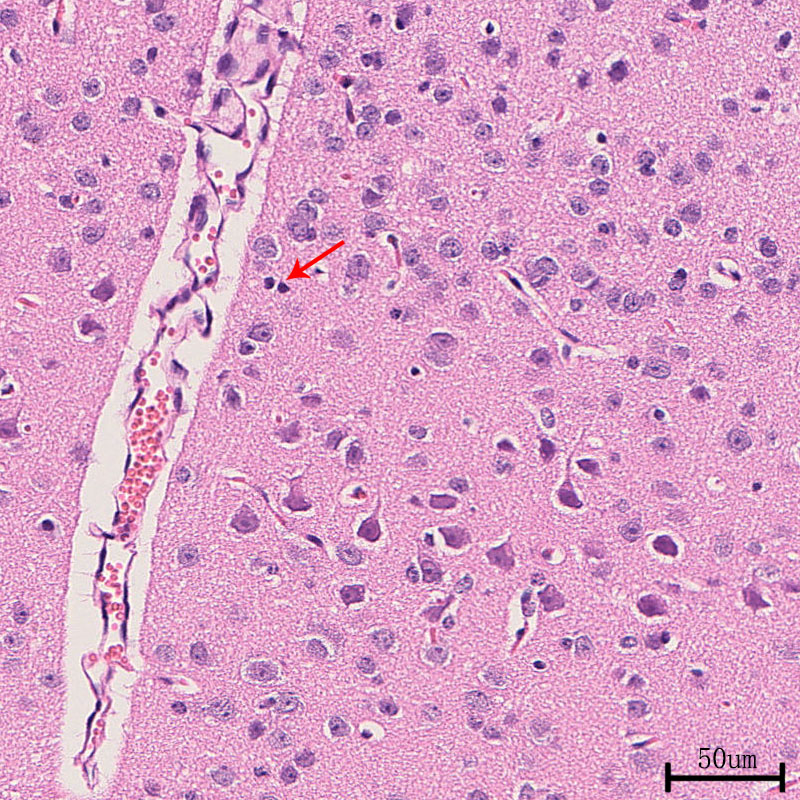

Supplement: S1 Data — Images files. SPR PDF files. CE5200 PDF file. S1D Fig report. S1A Fig sequencing file. (ZIP) [file ppat.1013741.s016.zip › Raw data/Images files/Fig 4E/Iron-supplemented.jpg]

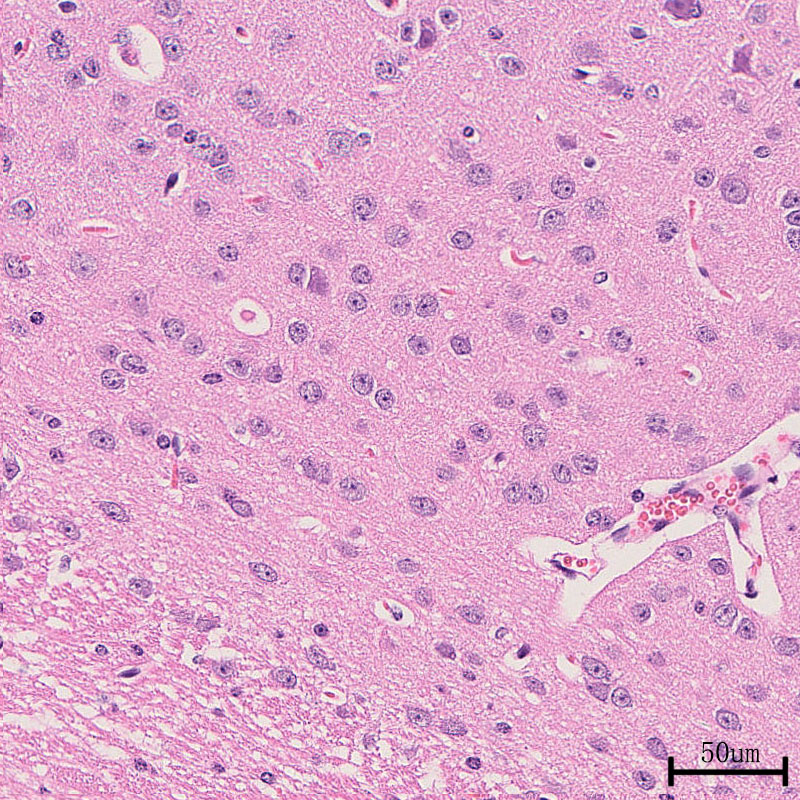

Supplement: S1 Data — Images files. SPR PDF files. CE5200 PDF file. S1D Fig report. S1A Fig sequencing file. (ZIP) [file ppat.1013741.s016.zip › Raw data/Images files/Fig 4E/Naïve.jpg]

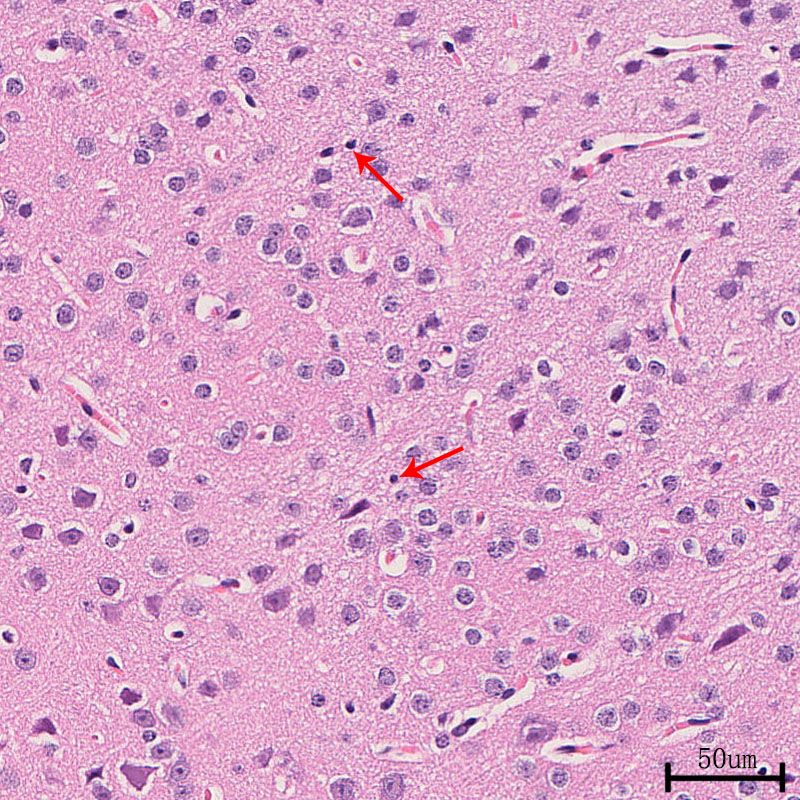

Supplement: S1 Data — Images files. SPR PDF files. CE5200 PDF file. S1D Fig report. S1A Fig sequencing file. (ZIP) [file ppat.1013741.s016.zip › Raw data/Images files/Fig 4E/Protein.jpg]

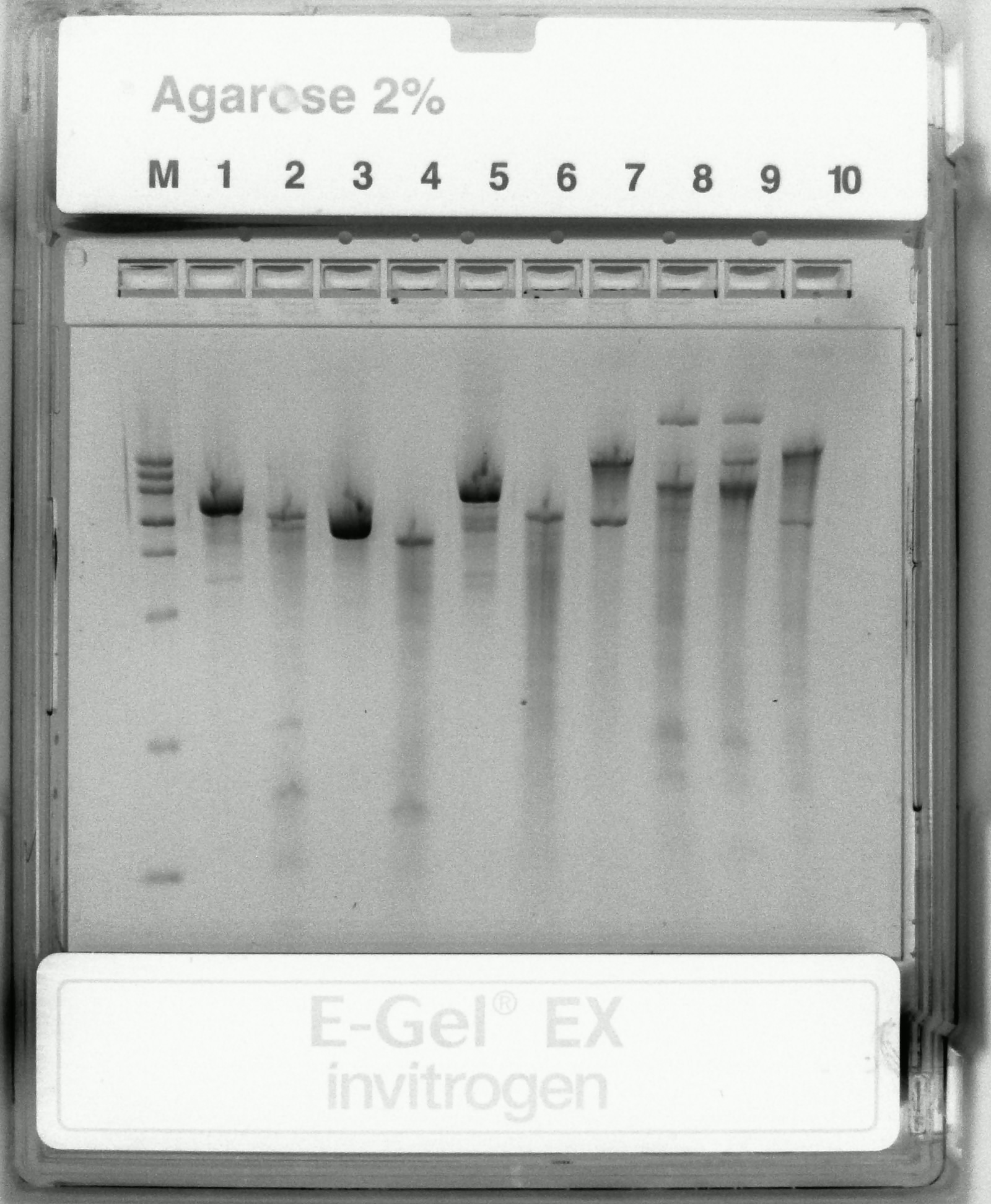

Supplement: S1 Data — Images files. SPR PDF files. CE5200 PDF file. S1D Fig report. S1A Fig sequencing file. (ZIP) [file ppat.1013741.s016.zip › Raw data/Images files/S1B Fig/S1B Fig.jpg]

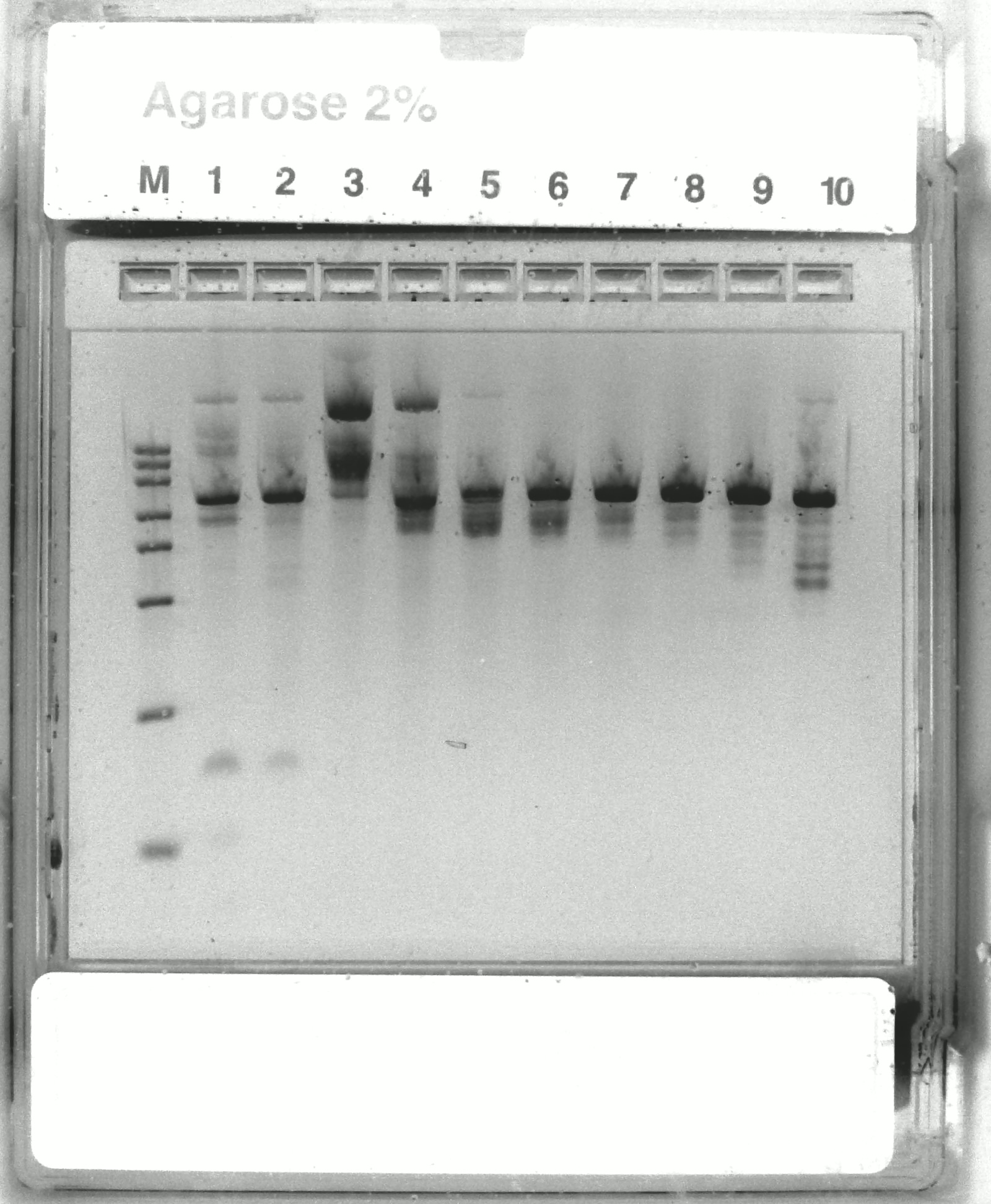

Supplement: S1 Data — Images files. SPR PDF files. CE5200 PDF file. S1D Fig report. S1A Fig sequencing file. (ZIP) [file ppat.1013741.s016.zip › Raw data/Images files/S1C Fig/S1C Fig Bottom.jpg]

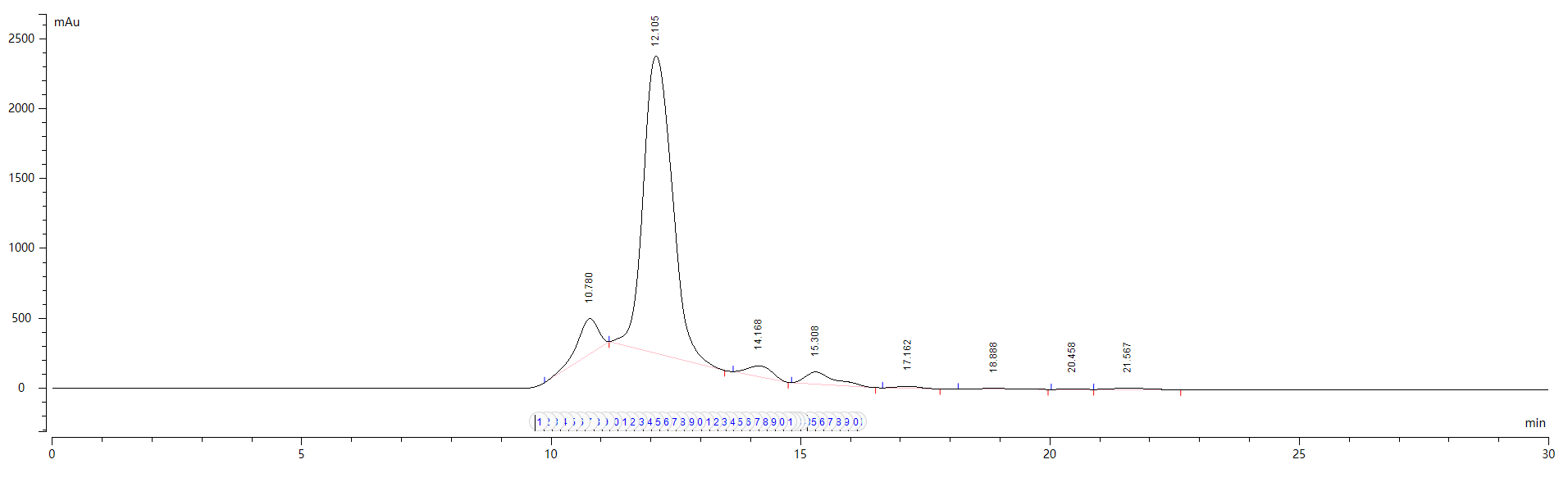

Supplement: S1 Data — Images files. SPR PDF files. CE5200 PDF file. S1D Fig report. S1A Fig sequencing file. (ZIP) [file ppat.1013741.s016.zip › Raw data/Images files/S1C Fig/S1C Fig Top.jpg]

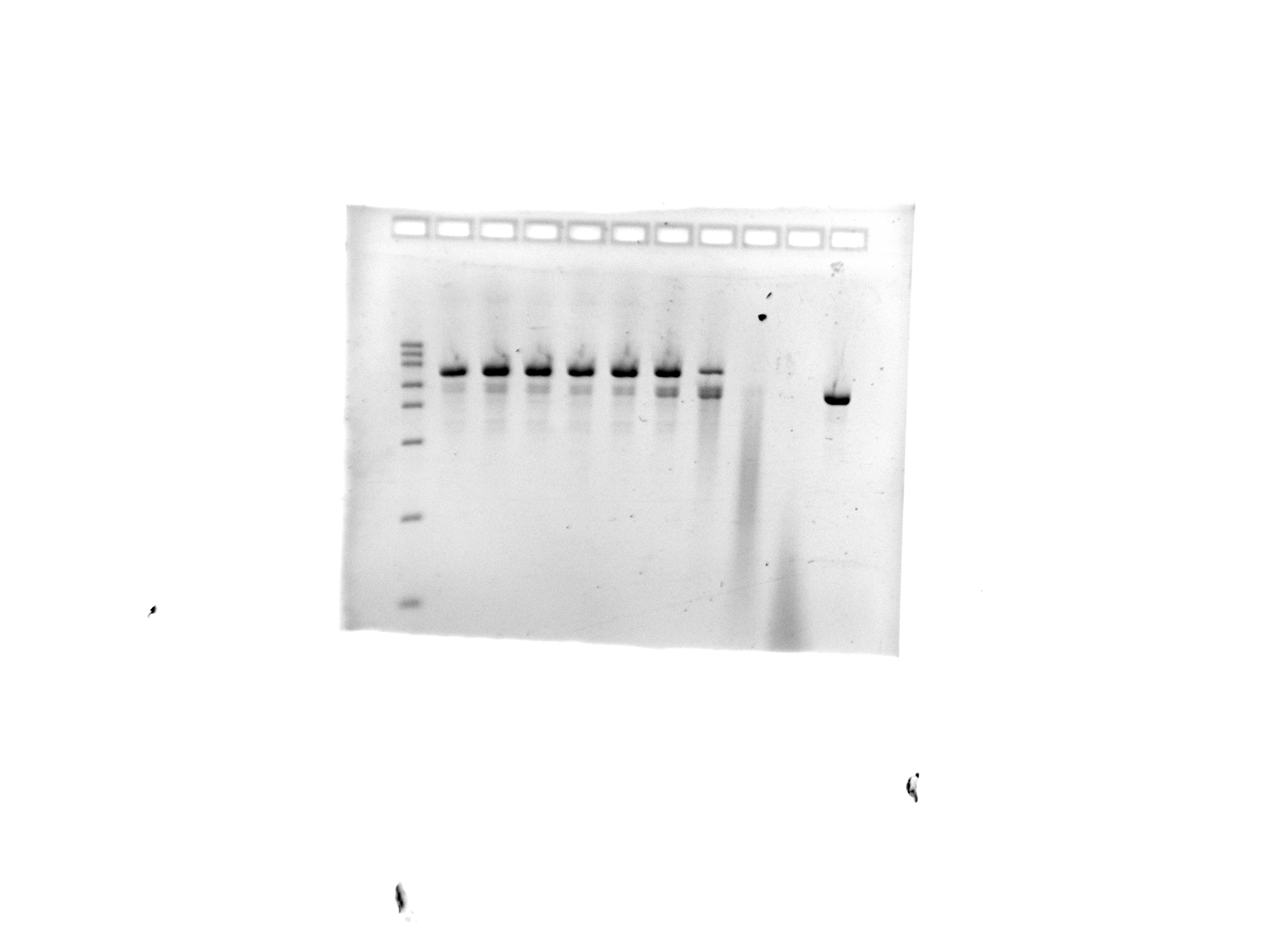

Supplement: S1 Data — Images files. SPR PDF files. CE5200 PDF file. S1D Fig report. S1A Fig sequencing file. (ZIP) [file ppat.1013741.s016.zip › Raw data/Images files/S1E Fig/S1E.tif]

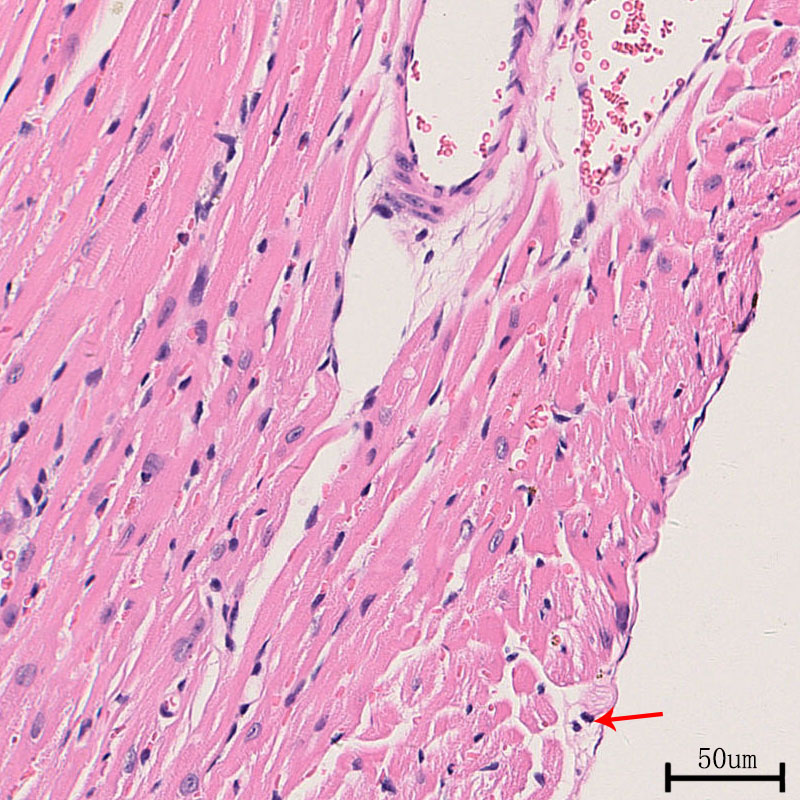

Supplement: S1 Data — Images files. SPR PDF files. CE5200 PDF file. S1D Fig report. S1A Fig sequencing file. (ZIP) [file ppat.1013741.s016.zip › Raw data/Images files/S8A Fig/circRNA 15μg.jpg]

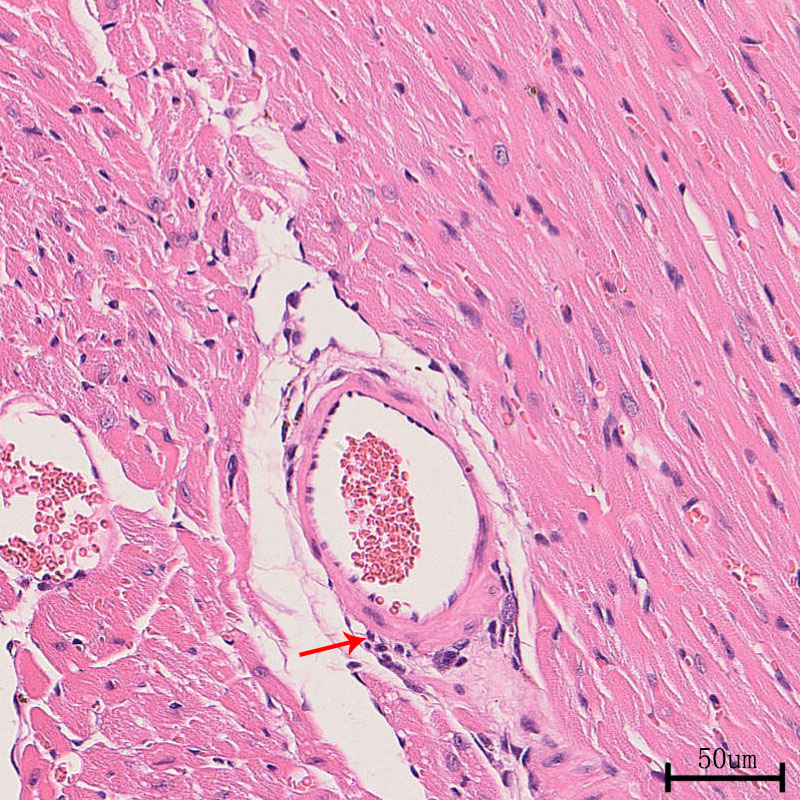

Supplement: S1 Data — Images files. SPR PDF files. CE5200 PDF file. S1D Fig report. S1A Fig sequencing file. (ZIP) [file ppat.1013741.s016.zip › Raw data/Images files/S8A Fig/circRNA 30μg.jpg]

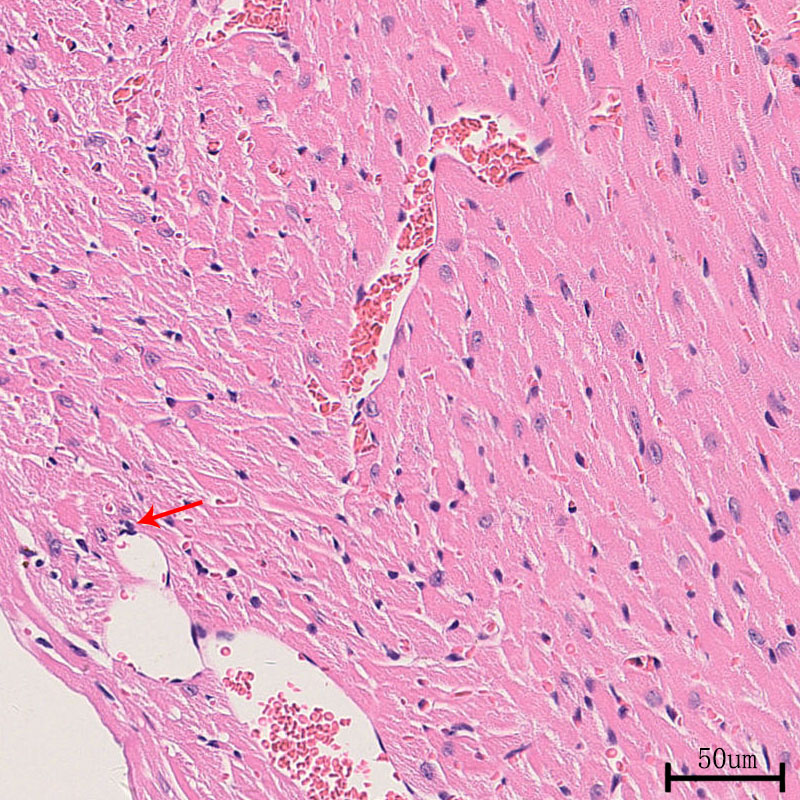

Supplement: S1 Data — Images files. SPR PDF files. CE5200 PDF file. S1D Fig report. S1A Fig sequencing file. (ZIP) [file ppat.1013741.s016.zip › Raw data/Images files/S8A Fig/Iron-supplemented.jpg]

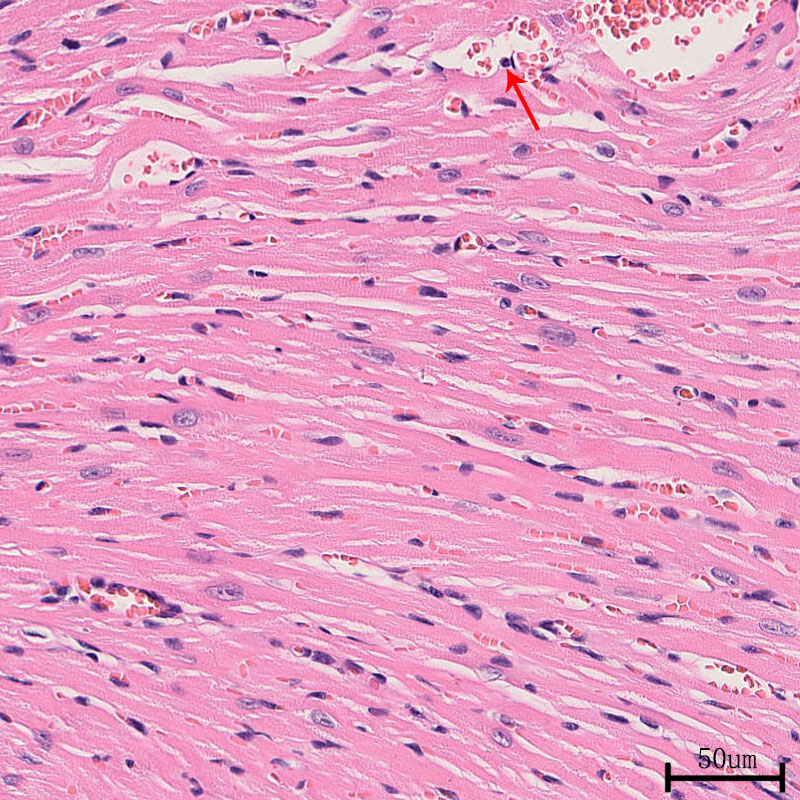

Supplement: S1 Data — Images files. SPR PDF files. CE5200 PDF file. S1D Fig report. S1A Fig sequencing file. (ZIP) [file ppat.1013741.s016.zip › Raw data/Images files/S8A Fig/Naïve.jpg]

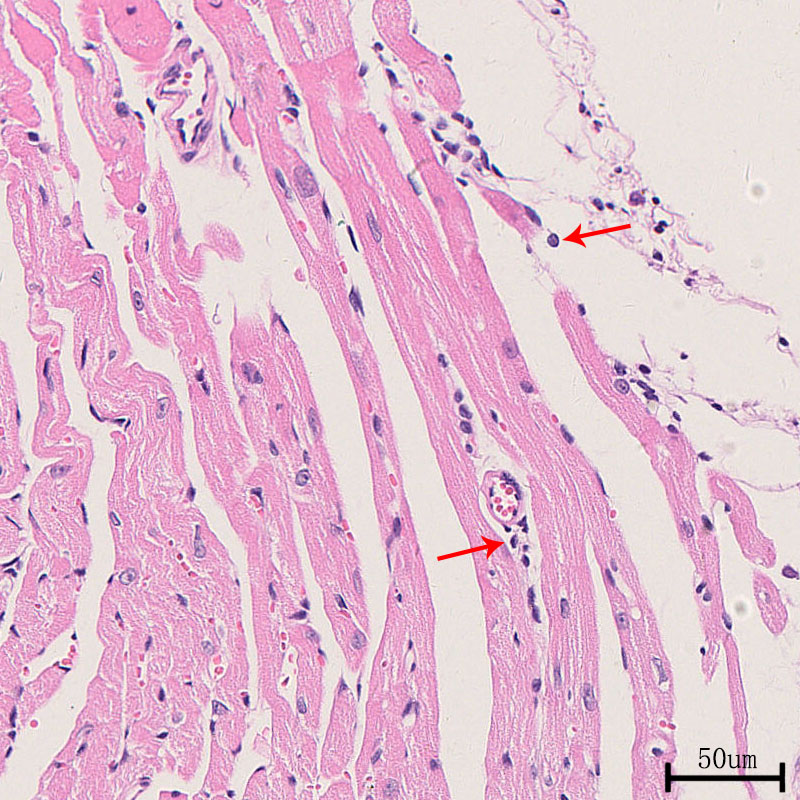

Supplement: S1 Data — Images files. SPR PDF files. CE5200 PDF file. S1D Fig report. S1A Fig sequencing file. (ZIP) [file ppat.1013741.s016.zip › Raw data/Images files/S8A Fig/Protein.jpg]

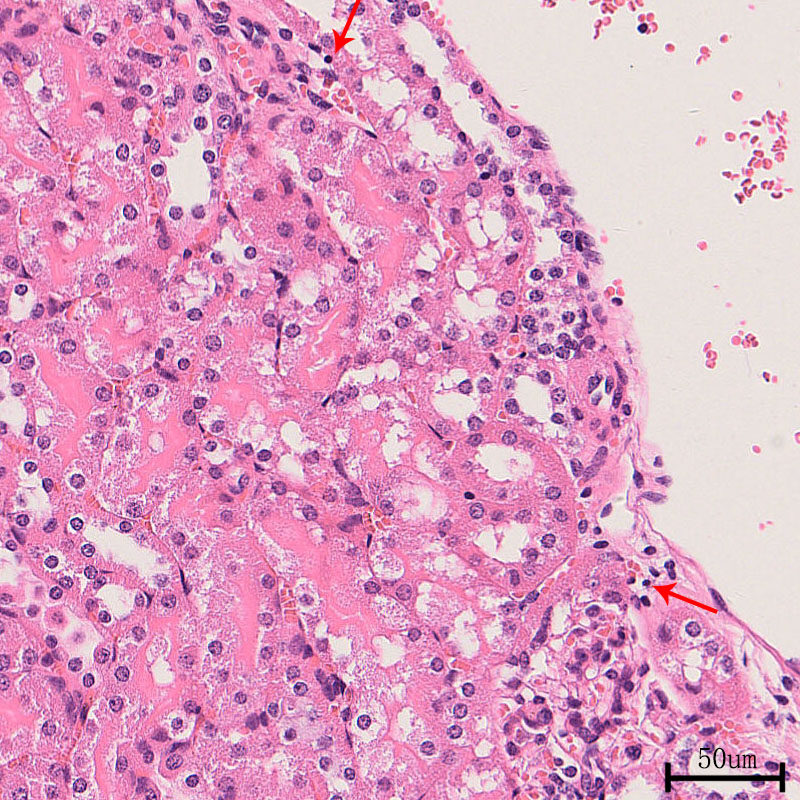

Supplement: S1 Data — Images files. SPR PDF files. CE5200 PDF file. S1D Fig report. S1A Fig sequencing file. (ZIP) [file ppat.1013741.s016.zip › Raw data/Images files/S8B Fig/circRNA 15μg.jpg]

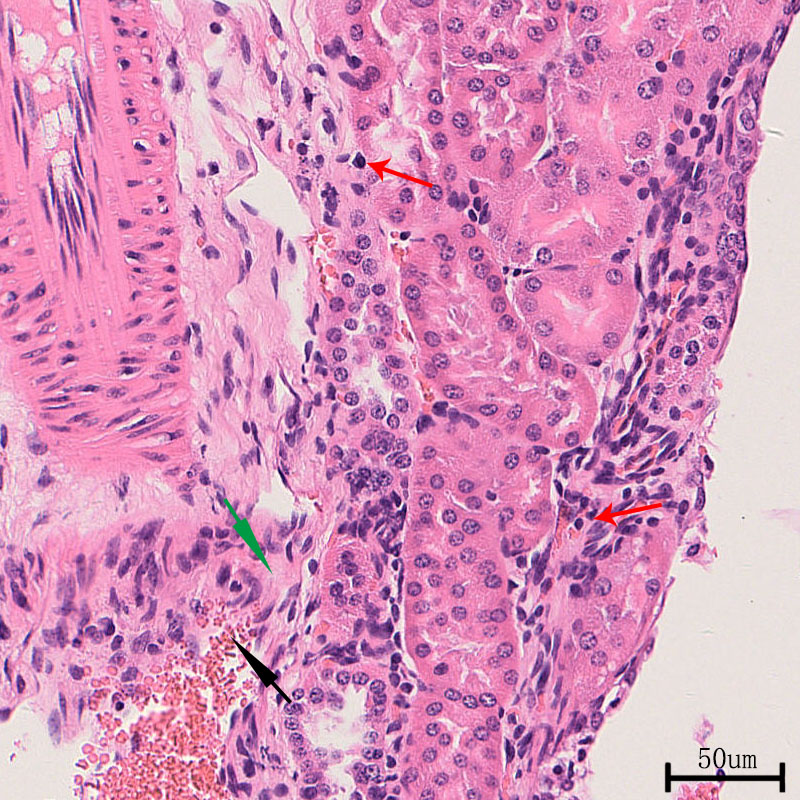

Supplement: S1 Data — Images files. SPR PDF files. CE5200 PDF file. S1D Fig report. S1A Fig sequencing file. (ZIP) [file ppat.1013741.s016.zip › Raw data/Images files/S8B Fig/circRNA 30μg.jpg]

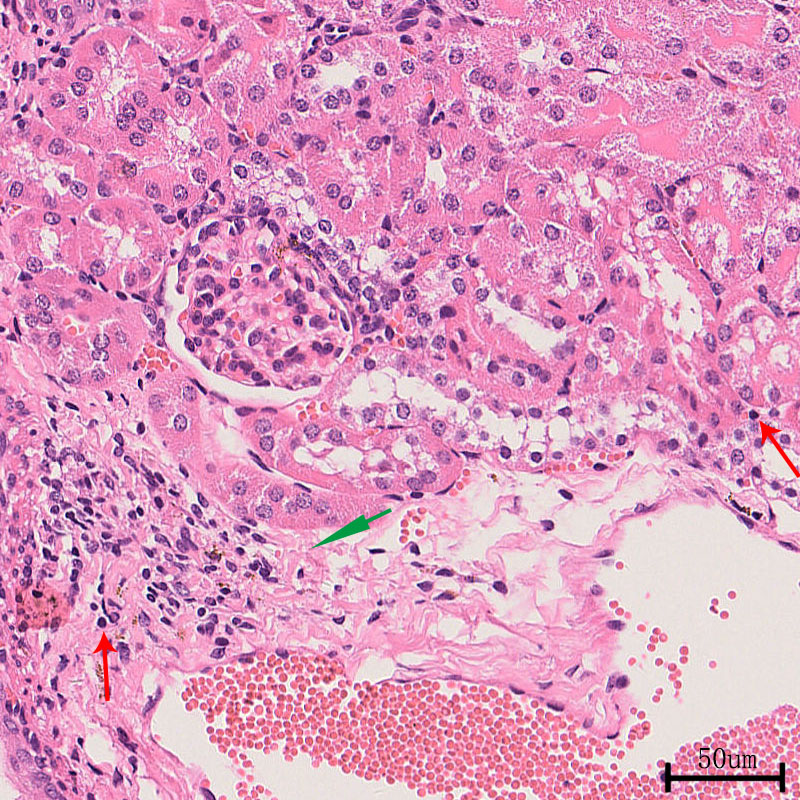

Supplement: S1 Data — Images files. SPR PDF files. CE5200 PDF file. S1D Fig report. S1A Fig sequencing file. (ZIP) [file ppat.1013741.s016.zip › Raw data/Images files/S8B Fig/Iron-supplemented.jpg]

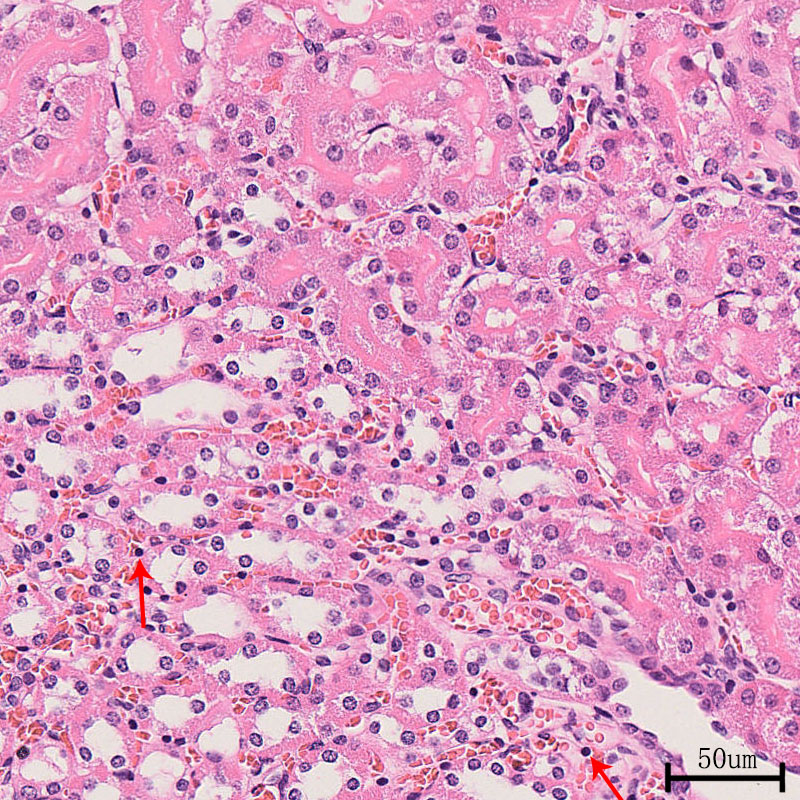

Supplement: S1 Data — Images files. SPR PDF files. CE5200 PDF file. S1D Fig report. S1A Fig sequencing file. (ZIP) [file ppat.1013741.s016.zip › Raw data/Images files/S8B Fig/Naïve.jpg]

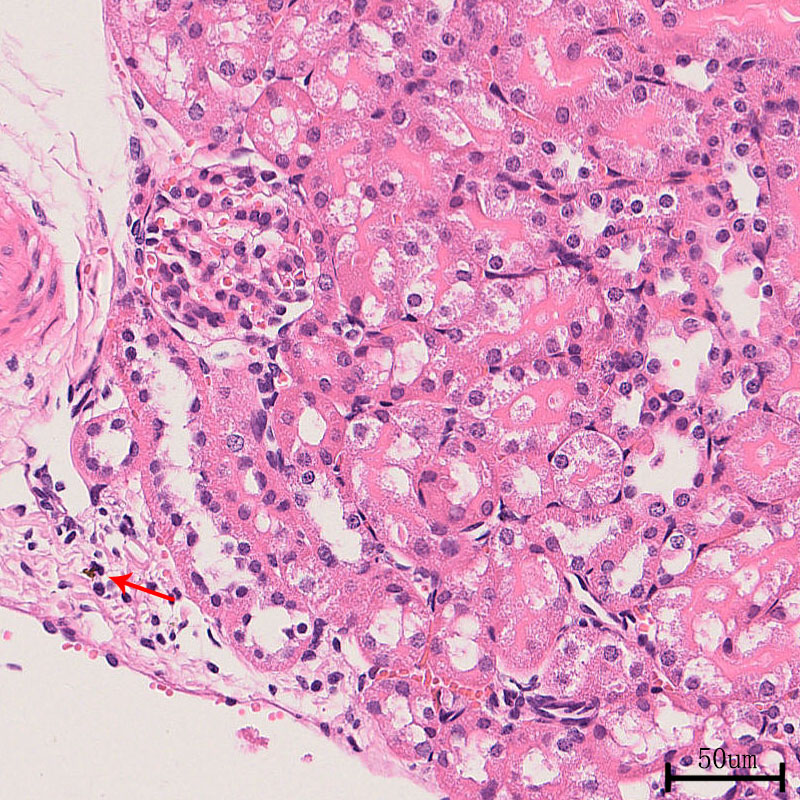

Supplement: S1 Data — Images files. SPR PDF files. CE5200 PDF file. S1D Fig report. S1A Fig sequencing file. (ZIP) [file ppat.1013741.s016.zip › Raw data/Images files/S8B Fig/Protein.jpg]

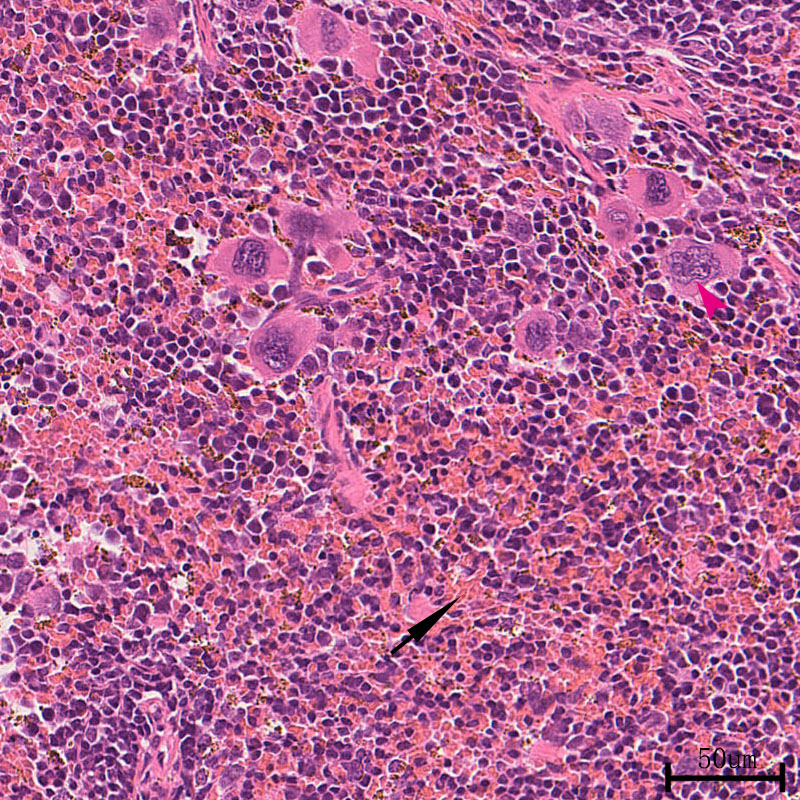

Supplement: S1 Data — Images files. SPR PDF files. CE5200 PDF file. S1D Fig report. S1A Fig sequencing file. (ZIP) [file ppat.1013741.s016.zip › Raw data/Images files/S8C Fig/circRNA 15μg.jpg]

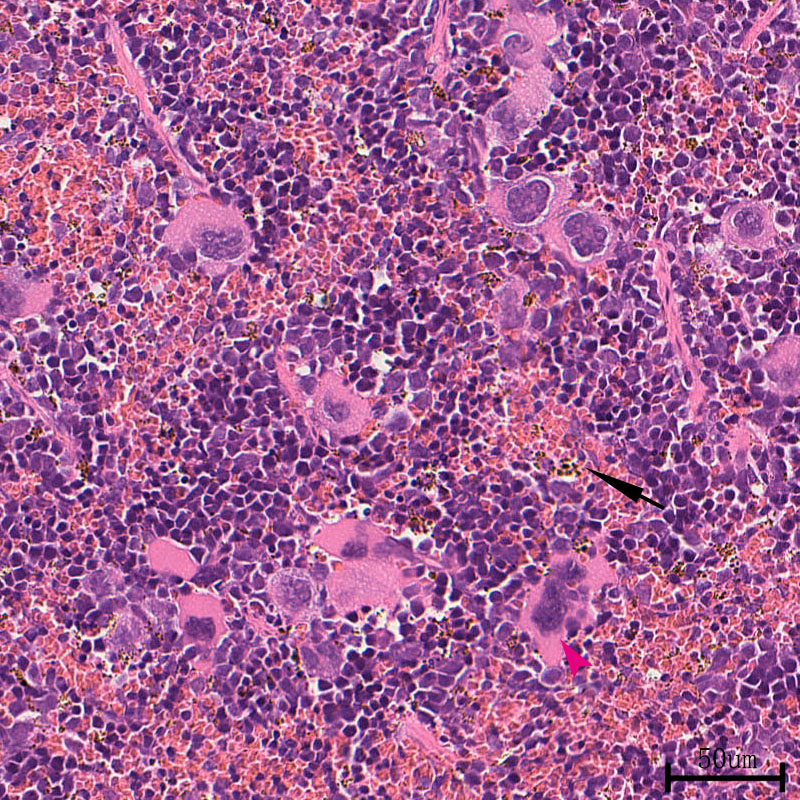

Supplement: S1 Data — Images files. SPR PDF files. CE5200 PDF file. S1D Fig report. S1A Fig sequencing file. (ZIP) [file ppat.1013741.s016.zip › Raw data/Images files/S8C Fig/circRNA 30μg.jpg]

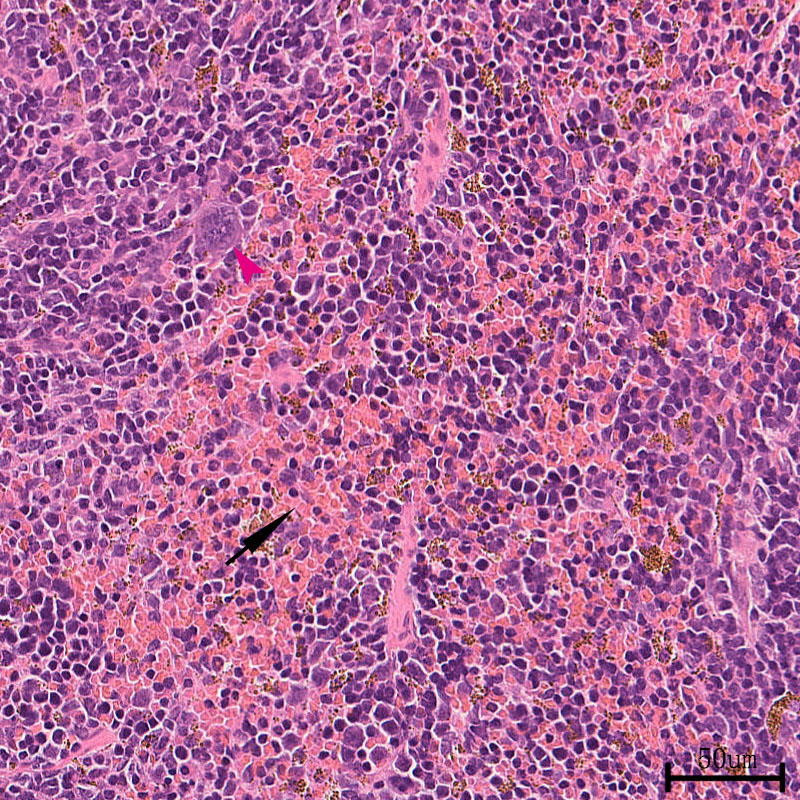

Supplement: S1 Data — Images files. SPR PDF files. CE5200 PDF file. S1D Fig report. S1A Fig sequencing file. (ZIP) [file ppat.1013741.s016.zip › Raw data/Images files/S8C Fig/Iron-supplemented.jpg]

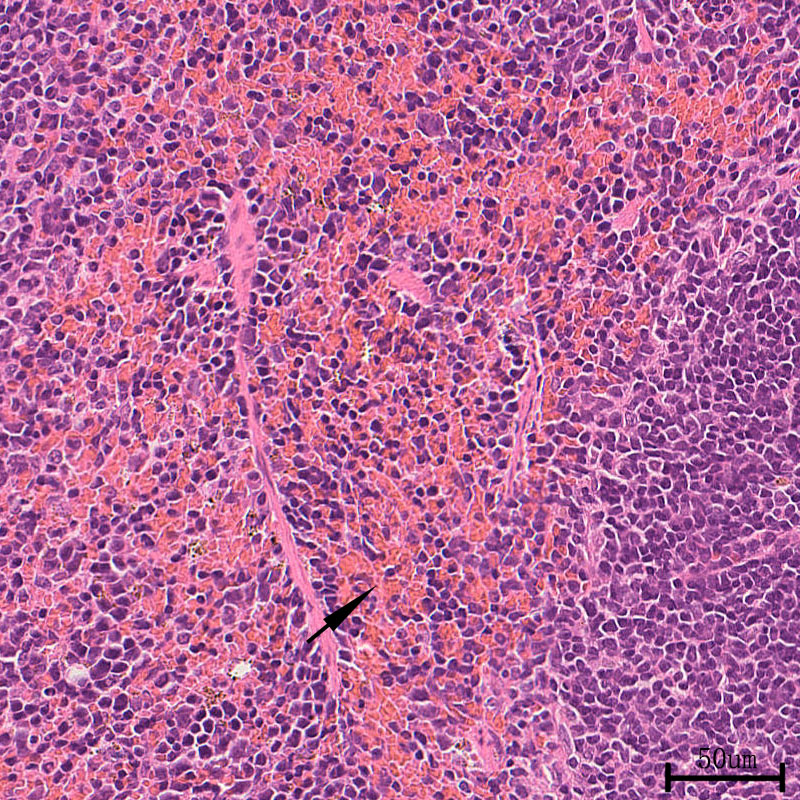

Supplement: S1 Data — Images files. SPR PDF files. CE5200 PDF file. S1D Fig report. S1A Fig sequencing file. (ZIP) [file ppat.1013741.s016.zip › Raw data/Images files/S8C Fig/Naïve.jpg]

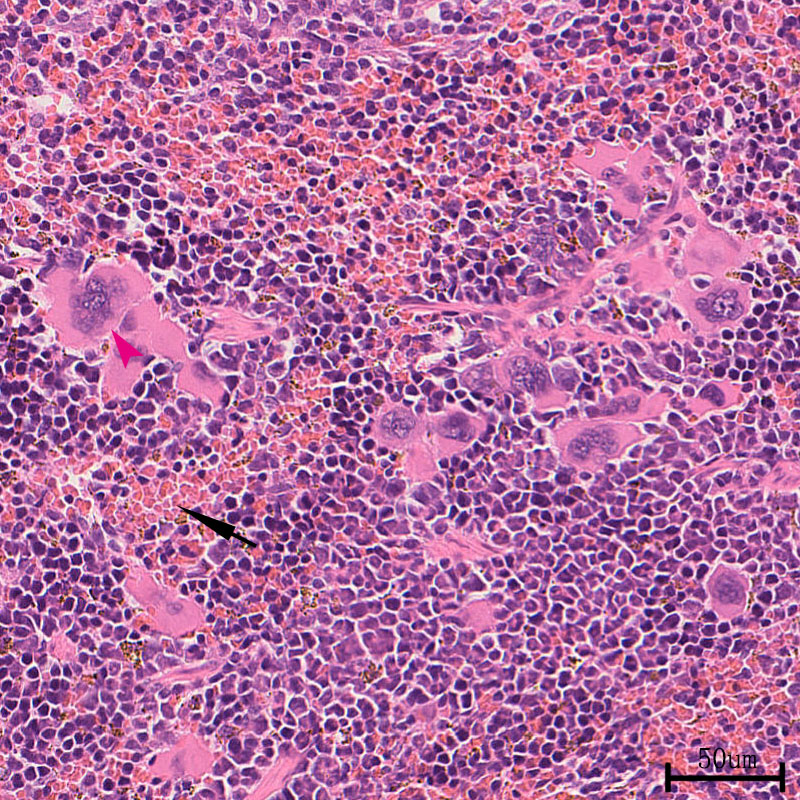

Supplement: S1 Data — Images files. SPR PDF files. CE5200 PDF file. S1D Fig report. S1A Fig sequencing file. (ZIP) [file ppat.1013741.s016.zip › Raw data/Images files/S8C Fig/Protein.jpg]

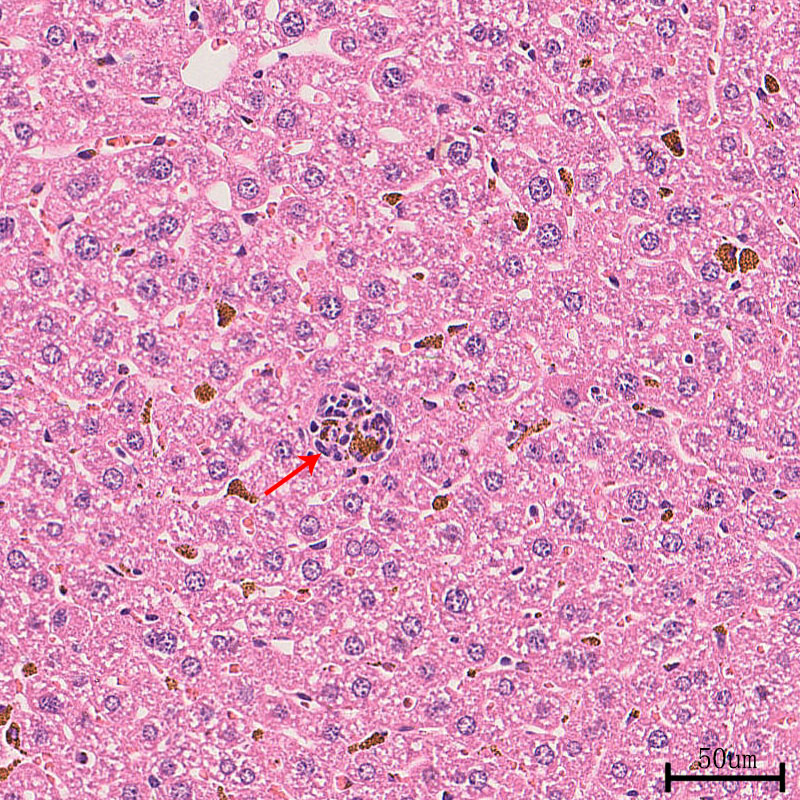

Supplement: S1 Data — Images files. SPR PDF files. CE5200 PDF file. S1D Fig report. S1A Fig sequencing file. (ZIP) [file ppat.1013741.s016.zip › Raw data/Images files/S8D Fig/circRNA 15μg.jpg]

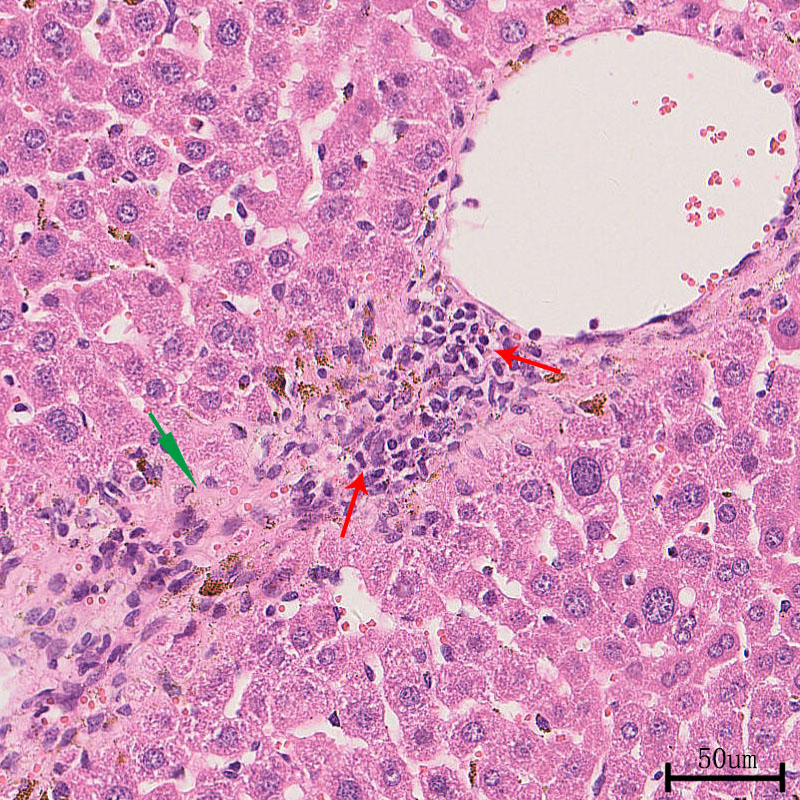

Supplement: S1 Data — Images files. SPR PDF files. CE5200 PDF file. S1D Fig report. S1A Fig sequencing file. (ZIP) [file ppat.1013741.s016.zip › Raw data/Images files/S8D Fig/circRNA 30μg.jpg]

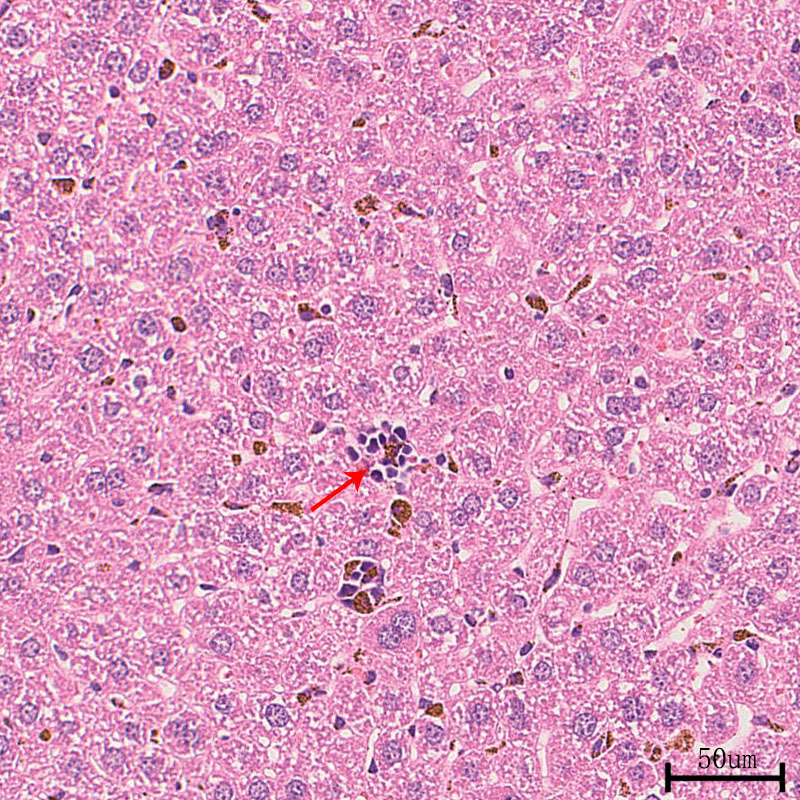

Supplement: S1 Data — Images files. SPR PDF files. CE5200 PDF file. S1D Fig report. S1A Fig sequencing file. (ZIP) [file ppat.1013741.s016.zip › Raw data/Images files/S8D Fig/Iron-supplemented.jpg]

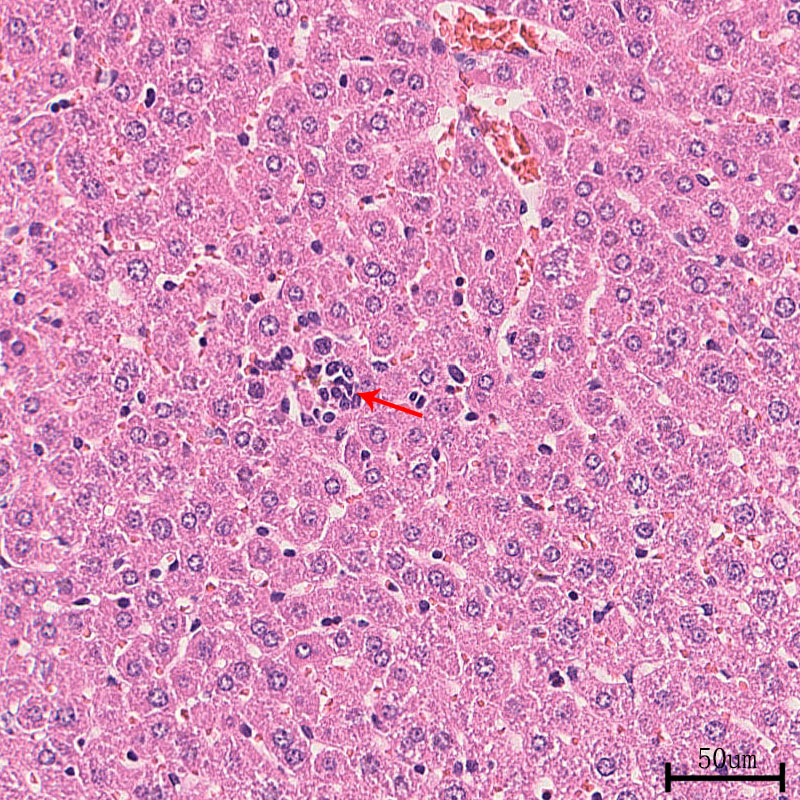

Supplement: S1 Data — Images files. SPR PDF files. CE5200 PDF file. S1D Fig report. S1A Fig sequencing file. (ZIP) [file ppat.1013741.s016.zip › Raw data/Images files/S8D Fig/Naïve.jpg]

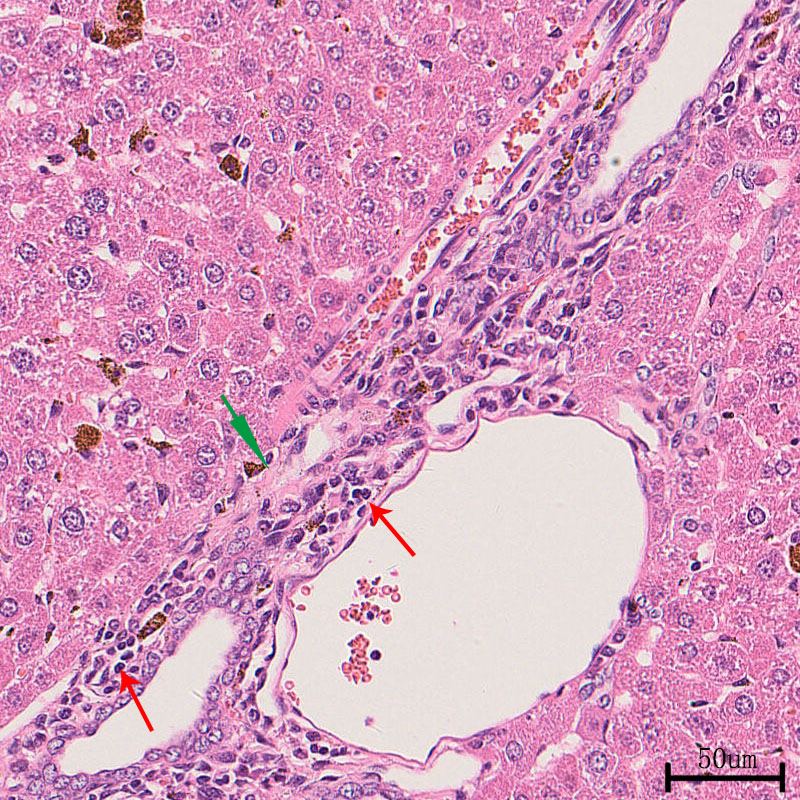

Supplement: S1 Data — Images files. SPR PDF files. CE5200 PDF file. S1D Fig report. S1A Fig sequencing file. (ZIP) [file ppat.1013741.s016.zip › Raw data/Images files/S8D Fig/Protein.jpg]
