# Supplementary material for: A circular RNA vaccine induces durable and cross-protective immunity against Neisseria meningitidis serogroup B in mice
Source: PLoS Pathog. 2026 May 11;22(5):e1013741. doi: 10.1371/journal.ppat.1013741 (PMC13160355; doi:10.1371/journal.ppat.1013741)

Instrument controller software run summary:

**Filename and data path:**	C:\Agilent Technologies\Data\2025 06 04\11-49-23\2025 06 04 11H 49M.raw
**Created:**	Wednesday, 4 June, 2025 12:10:03 PM
**Number of capillaries:**	12
**Array serial number:**	022124-20SFS
**Effective length:**	33 cm
**Array usage count:**	20
**Instrument type:**	5200 Fragment Analyzer
**Instrument controller software version:**	4.0.0.11
**Device serial number:**	MY2340AA54

Method Information

**Method name:**	DNF-471-33 - SS Total RNA 15nt.mthds
**Gel prime:**	No
**Full conditioning:**	Yes
**Gel prime to buffer:**	Yes
**Gel selection:**	Gel 1
**Prerun:**	8.0 kV, 30 sec.
**Rinse:**	No
**Marker 1:**	No
**Rinse:**	Tray: Marker, Row: A, Number of dips: 2
**Sample injection:**	5.0 kV, 4 sec.
**Separation:**	8.0 kV, 40.0 min.
**Tray Name:**	Tray-1

**Analysis mode:**	DNA

Report Generated on 04-Jun-2025 at 01:20:09 PM

Notes

Gel Image

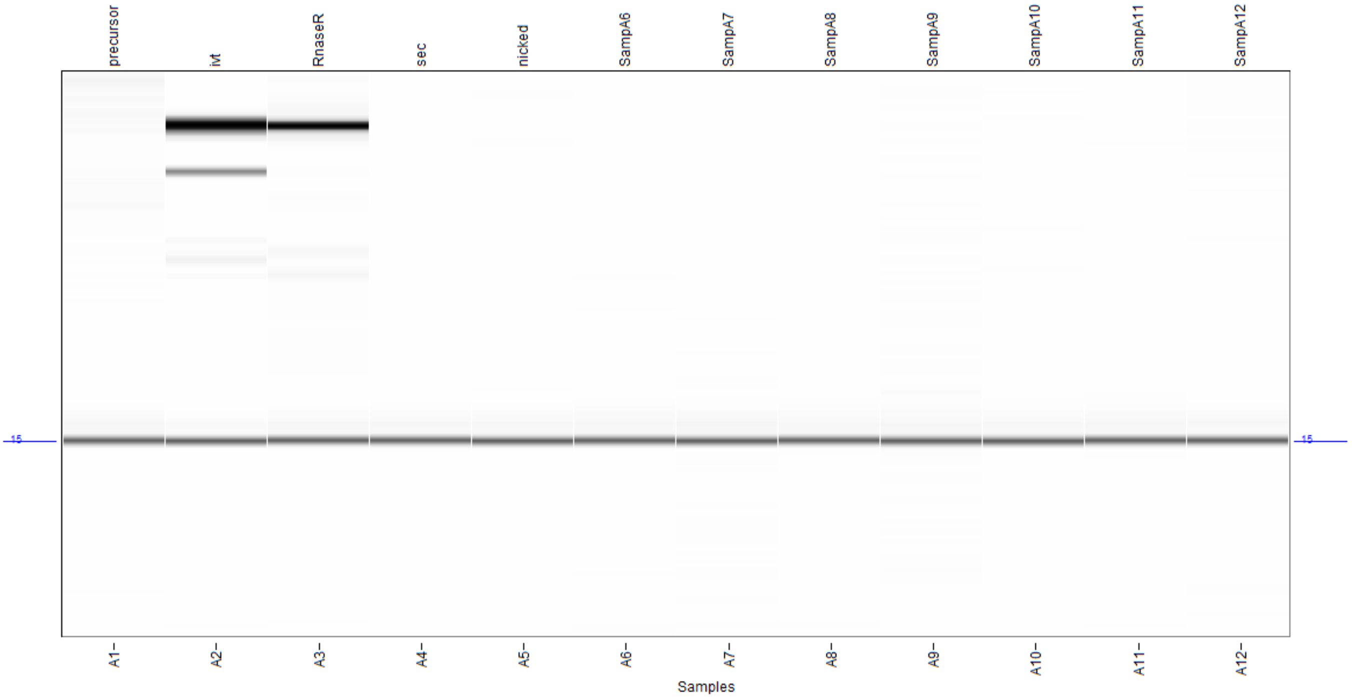
precursor
ivt
RnaseR
sec
nicked
SampA6
SampA7
SampA8
SampA9
SampA10
SampA11
SampA12
15
15
A1
A2
A3
A4
A5
A6
A7
A8
A9
A10
A11
A12
Samples

Filename and data path: C:\Agilent Technologies\Data\2025 06 04\11-49-23\2025 06 04 11H 49M.raw

A1: precursor

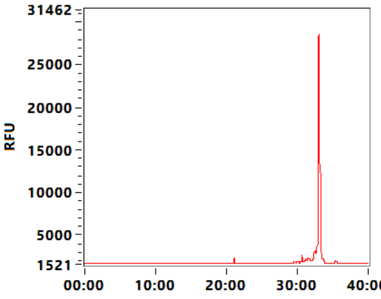

A2: ivt

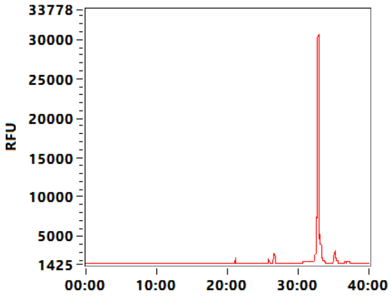

A3: RnaseR

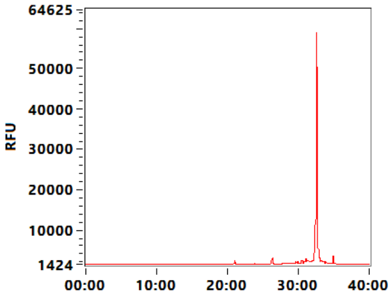

A4: sec

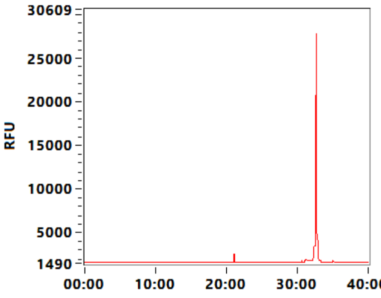

A5: nicked

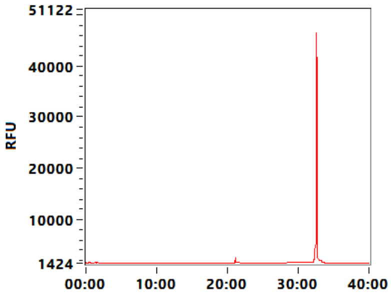

A6: SampA6

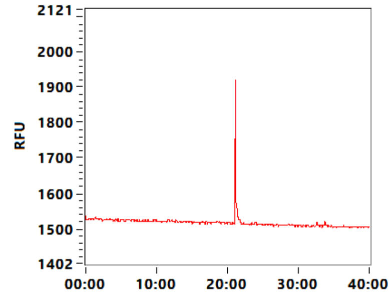

A7: SampA7

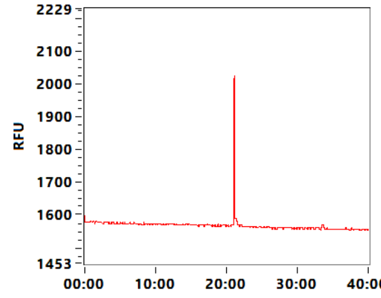

A8: SampA8

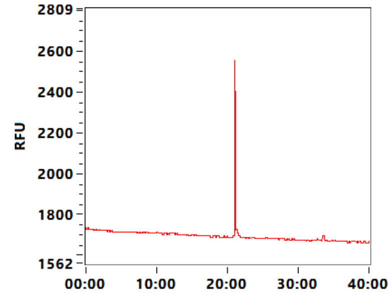

A9: SampA9

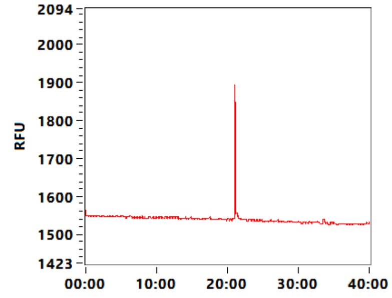

A10: SampA10

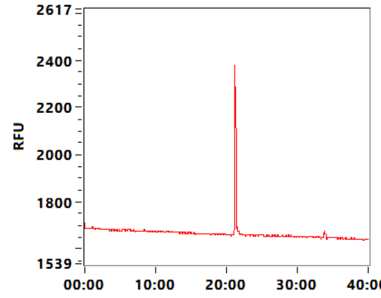

A11: SampA11

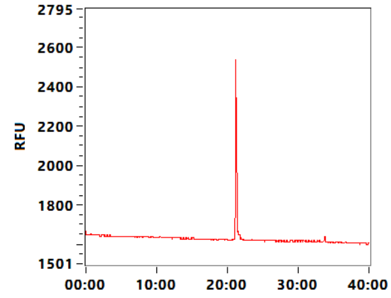

A12: SampA12

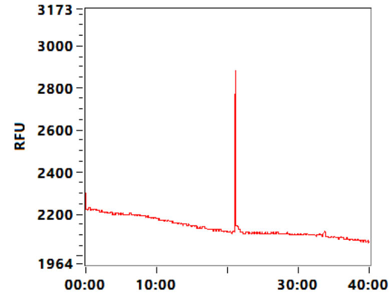

Sample: precursor
Well location: A1
Created: Wednesday, 4 June, 2025 12:10:03 PM

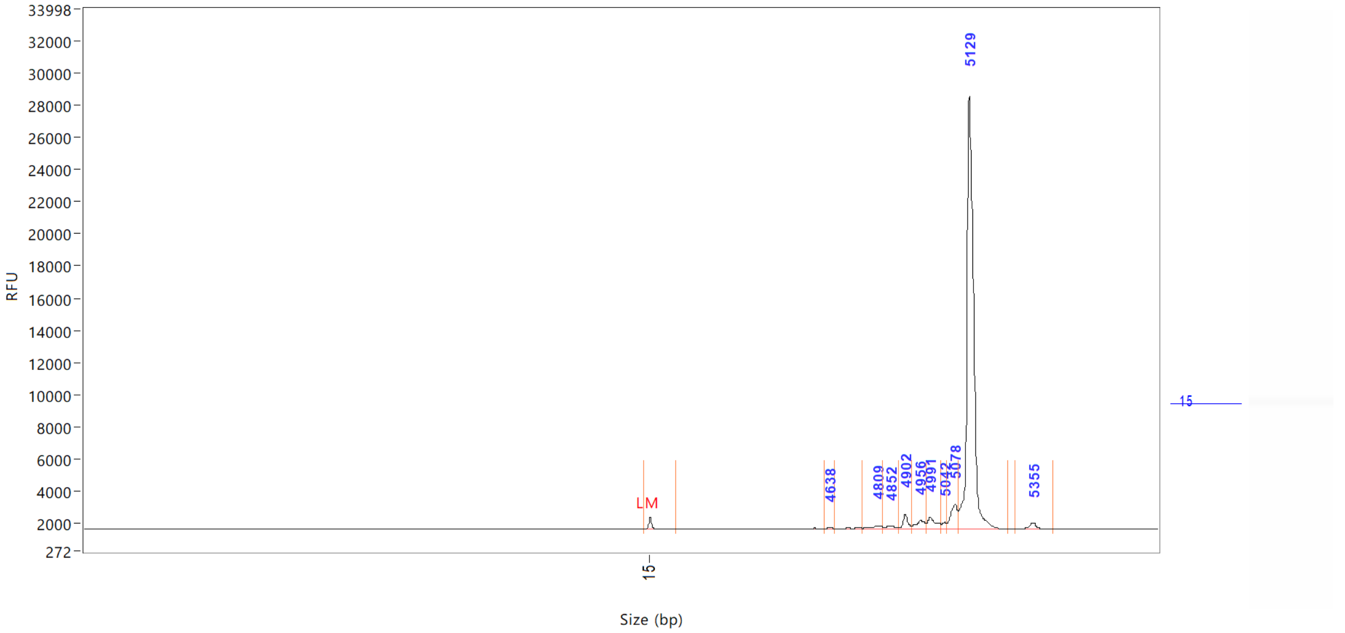

Sample Peak Width (sec): 6	Sample Min Peak Height: 50	Sample Baseline V to V?: Y	Sample Baseline V to V pts: 3
Sample filter: Binomial	Number of points for filter: 9	Sample start region (min): 0	Sample end region (min): 40
Manual baseline start (min): 18	Manual baseline end (min): 38	Marker peak width (sec): 6	Marker min peak height: 100
Marker baseline V to V?: Y	Marker baseline V to V points: 3	Lower marker selection: First peak > 100 RFU	Upper marker selection: Last peak > 100 RFU
Ladder size (bp)15, 200, 500, 1000, 1500, 2000, 3000, 4000, 6000	Quantification using: Ladder<br>Final concentration (ng/uL): 8.0000<br>Dilution factor: 12.0	Minimum RFU for data processing: 2	

Sample: precursor
Well location: A1
Created: Wednesday, 4 June, 2025 12:10:03 PM

Table Information

Peak Table

Peak	Size	Concentration	From	To	Average size	Percent CV	RFU	Corrected peak area
	(bp)	(ng/uL)	(bp)	(bp)	(bp)			
1	15 (LM)	Nan	Nan	4093	Nan	Nan	761	4.745
2	4638	Nan	4615	4654	4635	0.22	81	0.776
3	4809	Nan	4751	4822	4794	0.39	229	3.130
4	4852	Nan	4822	4880	4849	0.33	178	2.958
5	4902	Nan	4880	4923	4903	0.18	947	6.291
6	4956	Nan	4923	4975	4953	0.29	528	6.590
7	4991	Nan	4975	5029	4999	0.30	712	8.434
8	5042	Nan	5029	5048	5039	0.11	414	2.518
9	5078	Nan	5048	5089	5072	0.21	1538	14.452
10	5129	Nan	5089	5266	5134	0.31	26973	231.492
11	5355	Nan	5291	5424	5352	0.27	394	3.355

TIC:	Nan	ng/uL
TIM:	Nan	nmole/L
Total concentration:	Nan	ng/uL

Sample: ivt
Well location: A2
Created: Wednesday, 4 June, 2025 12:10:03 PM

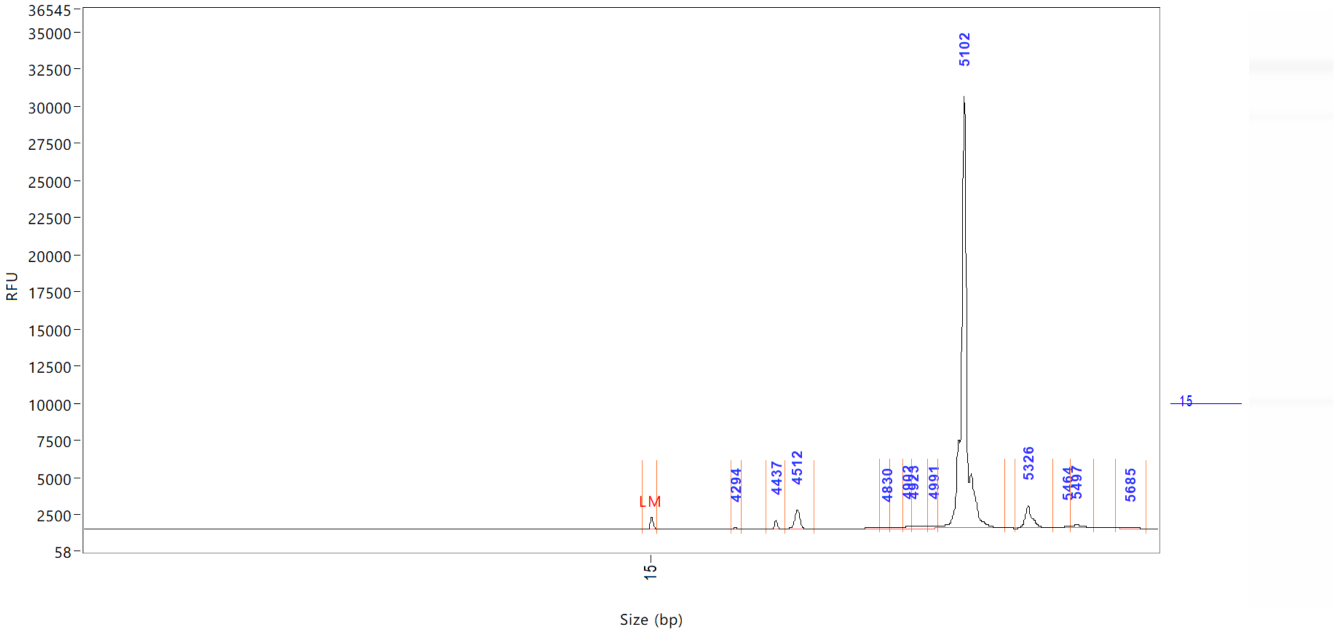

Sample Peak Width (sec): 6	Sample Min Peak Height: 50	Sample Baseline V to V?: Y	Sample Baseline V to V pts: 3
Sample filter: Binomial	Number of points for filter: 9	Sample start region (min): 0	Sample end region (min): 40
Manual baseline start (min): 18	Manual baseline end (min): 38	Marker peak width (sec): 6	Marker min peak height: 100
Marker baseline V to V?: Y	Marker baseline V to V points: 3	Lower marker selection: First peak > 100 RFU	Upper marker selection: Last peak > 100 RFU
Ladder size (bp)15, 200, 500, 1000, 1500, 2000, 3000, 4000, 6000	Quantification using: Ladder<br>Final concentration (ng/uL): 8.0000<br>Dilution factor: 12.0	Minimum RFU for data processing: 2	

Sample: ivt
Well location: A2
Created: Wednesday, 4 June, 2025 12:10:03 PM

Table Information

Peak Table

Peak	Size (bp)	Concentration (ng/uL)	From (bp)	To (bp)	Average size (bp)	Percent CV	RFU	Corrected peak area
1	15 (LM)	Nan	Nan	4017	Nan	Nan	763	4.238
2	4294	Nan	4278	4314	4295	0.15	66	0.408
3	4437	Nan	4403	4471	4437	0.10	567	2.594
4	4512	Nan	4471	4573	4512	0.19	1246	10.408
5	4830	Nan	4801	4839	4824	0.22	70	0.569
6	4902	Nan	4883	4914	4903	0.14	192	1.210
7	4923	Nan	4914	4972	4941	0.35	194	2.910
8	4991	Nan	4972	5010	4991	0.21	189	2.003
9	5102	Nan	5010	5247	5103	0.42	29122	214.555
10	5326	Nan	5280	5415	5332	0.34	1475	13.255
11	5464	Nan	5415	5477	5454	0.30	131	1.165
12	5497	Nan	5477	5556	5505	0.28	193	2.362
13	5685	Nan	5635	5744	5686	0.32	54	0.709

TIC:	Nan	ng/uL
TIM:	Nan	nmole/L
Total concentration:	Nan	ng/uL

Sample: RnaseR
Well location: A3
Created: Wednesday, 4 June, 2025 12:10:03 PM

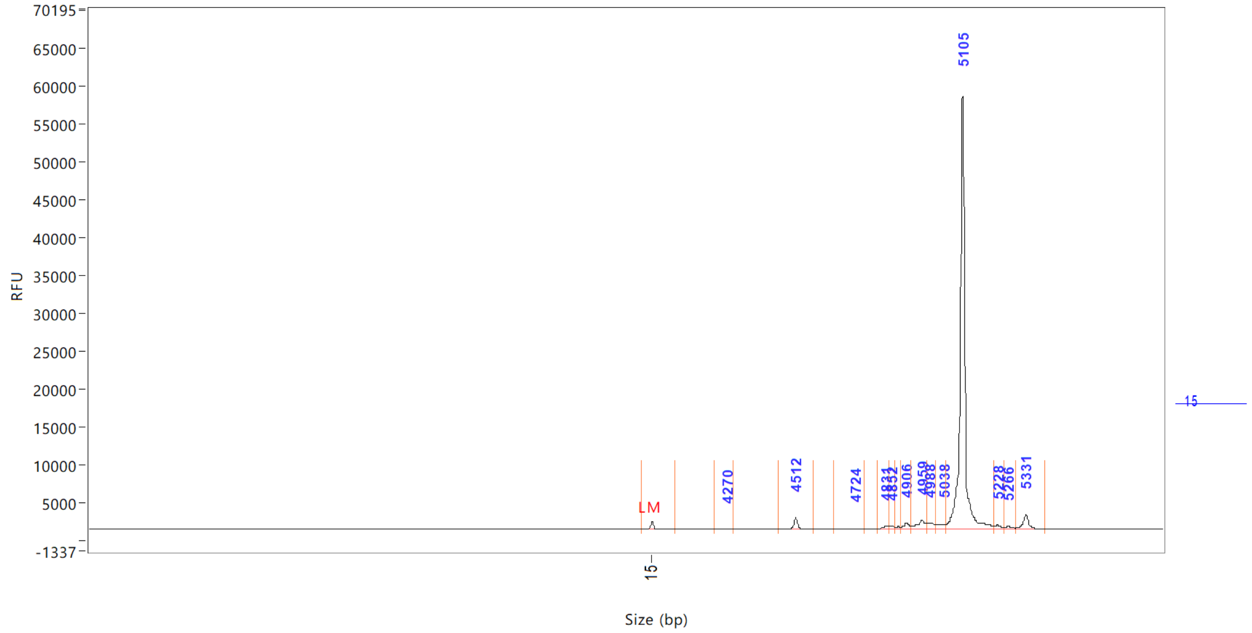

Sample Peak Width (sec): 6
Sample Min Peak Height: 50
Sample Baseline V to V?: Y
Sample Baseline V to V pts: 3
Sample filter: Binomial
Number of points for filter: 9
Sample start region (min): 0
Sample end region (min): 40
Manual baseline start (min): 18
Manual baseline end (min): 38
Marker peak width (sec): 6
Marker min peak height: 100
Marker baseline V to V?: Y
Marker baseline V to V points: 3
Lower marker selection: First peak > 100 RFU
Upper marker selection: Last peak > 100 RFU
Ladder size (bp)15, 200, 500, 1000, 1500, 2000, 3000, 4000, 6000
Quantification using: Ladder
Final concentration (ng/uL): 8.0000
Dilution factor: 12.0
Minimum RFU for data processing: 2

Sample: RnaseR

Well location: A3

Created: Wednesday, 4 June, 2025 12:10:03 PM

Table Information

Peak Table

Peak	Size (bp)	Concentration (ng/uL)	From (bp)	To (bp)	Average size (bp)	Percent CV	RFU	Corrected peak area
1	15 (LM)	Nan	Nan	4080	Nan	Nan	934	5.831
2	4270	Nan	4222	4289	4266	0.31	51	0.433
3	4512	Nan	4453	4575	4512	0.22	1525	9.305
4	4724	Nan	4646	4755	4700	0.67	84	2.867
5	4831	Nan	4800	4841	4827	0.22	373	2.920
6	4852	Nan	4841	4866	4853	0.15	402	3.116
7	4906	Nan	4883	4923	4905	0.21	763	6.772
8	4959	Nan	4923	4975	4953	0.28	1191	12.662
9	4988	Nan	4975	5012	4992	0.21	803	8.418
10	5038	Nan	5012	5046	5030	0.21	643	6.880
11	5105	Nan	5046	5216	5106	0.38	57184	374.949
12	5228	Nan	5216	5250	5231	0.18	481	3.676
13	5266	Nan	5250	5291	5268	0.20	301	2.374
14	5331	Nan	5291	5397	5329	0.22	1858	12.512

TIC:	Nan	ng/uL
TIM:	Nan	nmole/L
Total concentration:	Nan	ng/uL

Sample: sec
Well location: A4
Created: Wednesday, 4 June, 2025 12:10:03 PM

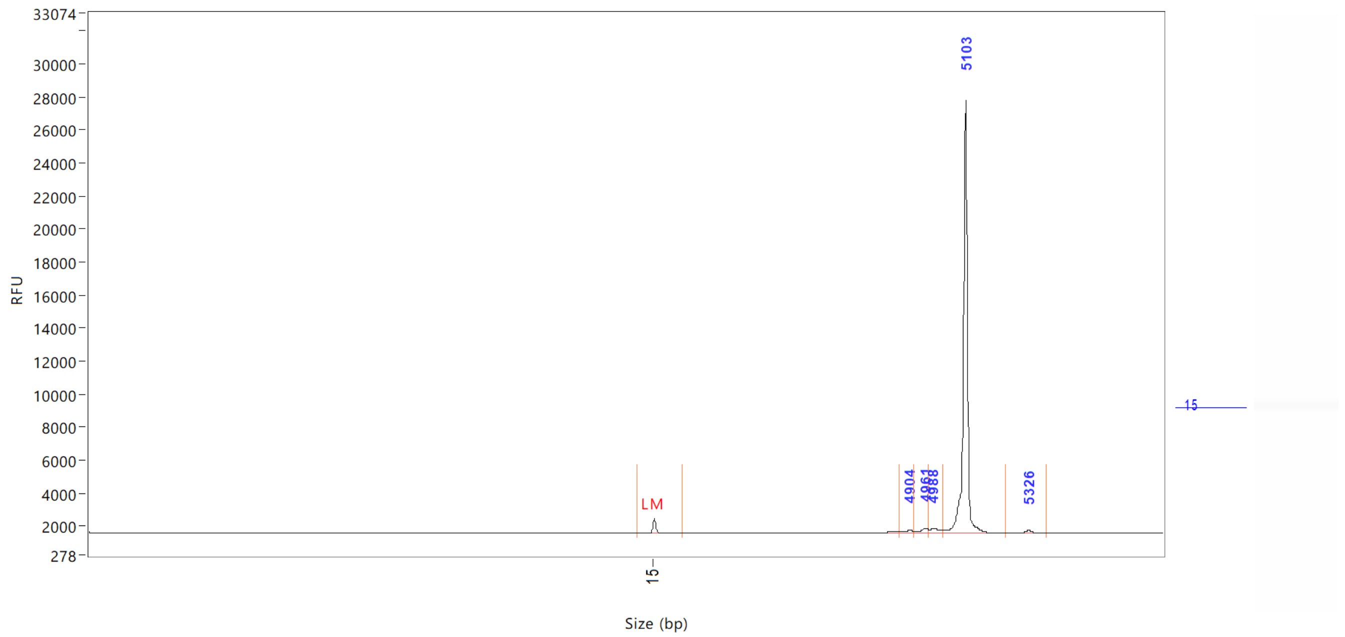

Sample Peak Width (sec): 6	Sample Min Peak Height: 50	Sample Baseline V to V?: Y	Sample Baseline V to V pts: 3
Sample filter: Binomial	Number of points for filter: 9	Sample start region (min): 0	Sample end region (min): 40
Manual baseline start (min): 18	Manual baseline end (min): 38	Marker peak width (sec): 6	Marker min peak height: 100
Marker baseline V to V?: Y	Marker baseline V to V points: 3	Lower marker selection: First peak > 100 RFU	Upper marker selection: Last peak > 100 RFU
Ladder size (bp)15, 200, 500, 1000, 1500, 2000, 3000, 4000, 6000	Quantification using: Ladder<br>Final concentration (ng/uL): 8.0000<br>Dilution factor: 12.0	Minimum RFU for data processing: 2	

Sample: sec
Well location: A4
Created: Wednesday, 4 June, 2025 12:10:03 PM

Table Information

Peak Table

Peak	Size	Concentration	From	To	Average size	Percent CV	RFU	Corrected peak area
	(bp)	(ng/uL)	(bp)	(bp)	(bp)			
1	15 (LM)	Nan	Nan	4101	Nan	Nan	909	5.718
2	4904	Nan	4866	4921	4900	0.28	207	1.874
3	4961	Nan	4921	4974	4953	0.28	288	2.986
4	4988	Nan	4974	5024	4997	0.29	255	3.474
5	5103	Nan	5024	5246	5100	0.28	26216	146.489
6	5326	Nan	5246	5386	5328	0.25	165	1.137

TIC:	Nan	ng/uL
TIM:	Nan	nmole/L
Total concentration:	Nan	ng/uL

Sample: nicked
Well location: A5
Created: Wednesday, 4 June, 2025 12:10:03 PM

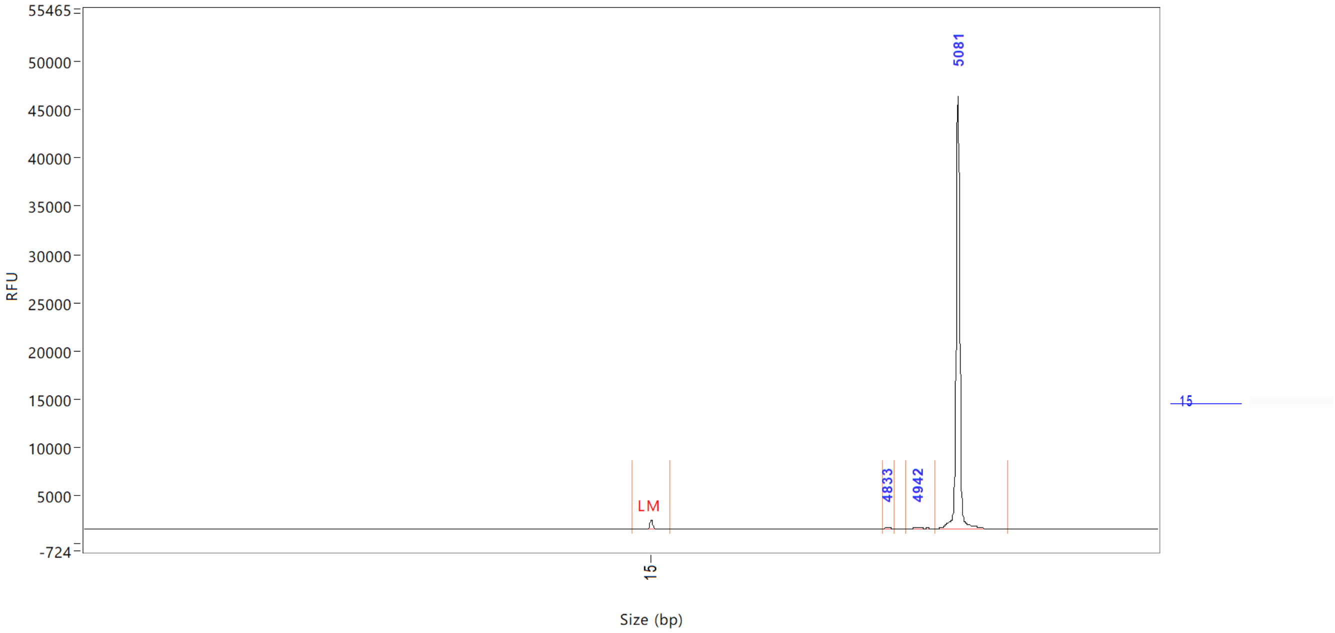

Sample Peak Width (sec): 6	Sample Min Peak Height: 50	Sample Baseline V to V?: Y	Sample Baseline V to V pts: 3
Sample filter: Binomial	Number of points for filter: 9	Sample start region (min): 0	Sample end region (min): 40
Manual baseline start (min): 18	Manual baseline end (min): 38	Marker peak width (sec): 6	Marker min peak height: 100
Marker baseline V to V?: Y	Marker baseline V to V points: 3	Lower marker selection: First peak > 100 RFU	Upper marker selection: Last peak > 100 RFU
Ladder size (bp)15, 200, 500, 1000, 1500, 2000, 3000, 4000, 6000	Quantification using: Ladder<br>Final concentration (ng/uL): 8.0000<br>Dilution factor: 12.0	Minimum RFU for data processing: 2	

Sample: nicked
Well location: A5
Created: Wednesday, 4 June, 2025 12:10:03 PM

Table Information

Peak Table

Peak	Size	Concentration	From	To	Average size	Percent CV	RFU	Corrected peak area
	(bp)	(ng/uL)	(bp)	(bp)	(bp)			
1	15 (LM)	Nan	Nan	4069	Nan	Nan	921	5.712
2	4833	Nan	4815	4860	4836	0.22	153	1.342
3	4942	Nan	4898	5002	4947	0.52	154	2.903
4	5081	Nan	5002	5257	5081	0.25	44927	229.679

TIC:	Nan	ng/uL
TIM:	Nan	nmole/L
Total concentration:	Nan	ng/uL

Sample: SampA6
Well location: A6
Created: Wednesday, 4 June, 2025 12:10:03 PM

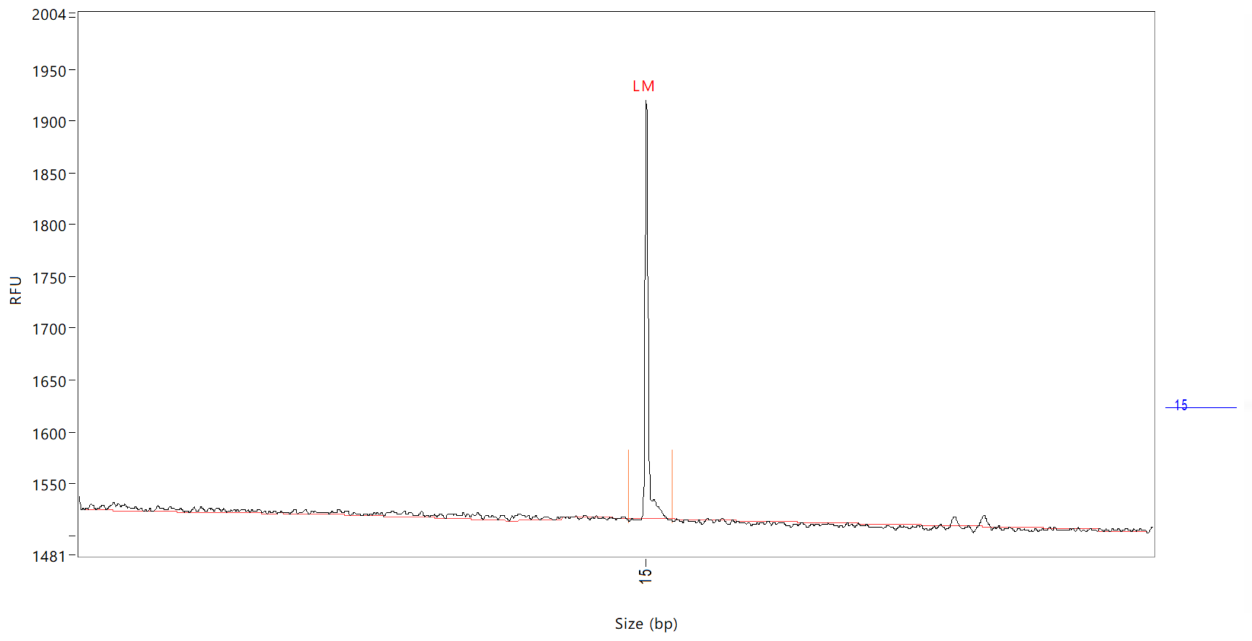

Sample Peak Width (sec): 6	Sample Min Peak Height: 50	Sample Baseline V to V?: Y	Sample Baseline V to V pts: 3
Sample filter: Binomial	Number of points for filter: 9	Sample start region (min): 0	Sample end region (min): 40
Manual baseline start (min): 18	Manual baseline end (min): 38	Marker peak width (sec): 6	Marker min peak height: 100
Marker baseline V to V?: Y	Marker baseline V to V points: 3	Lower marker selection: First peak > 100 RFU	Upper marker selection: Last peak > 100 RFU
Ladder size (bp)15, 200, 500, 1000, 1500, 2000, 3000, 4000, 6000	Quantification using: Ladder<br>Final concentration (ng/uL): 8.0000<br>Dilution factor: 12.0	Minimum RFU for data processing: 2	

Sample: SampA6
Well location: A6
Created: Wednesday, 4 June, 2025 12:10:03 PM

Table Information

Peak Table

Peak	Size	Concentration	From	To	Average size	Percent CV	RFU	Corrected peak area
	(bp)	(ng/uL)	(bp)	(bp)	(bp)			
1	15 (LM)	Nan	Nan	4094	Nan	Nan	402	2.530

TIC:	0.0000	ng/uL
TIM:	0.0000	nmole/L
Total concentration:	Nan	ng/uL

Sample: SampA7
Well location: A7
Created: Wednesday, 4 June, 2025 12:10:03 PM

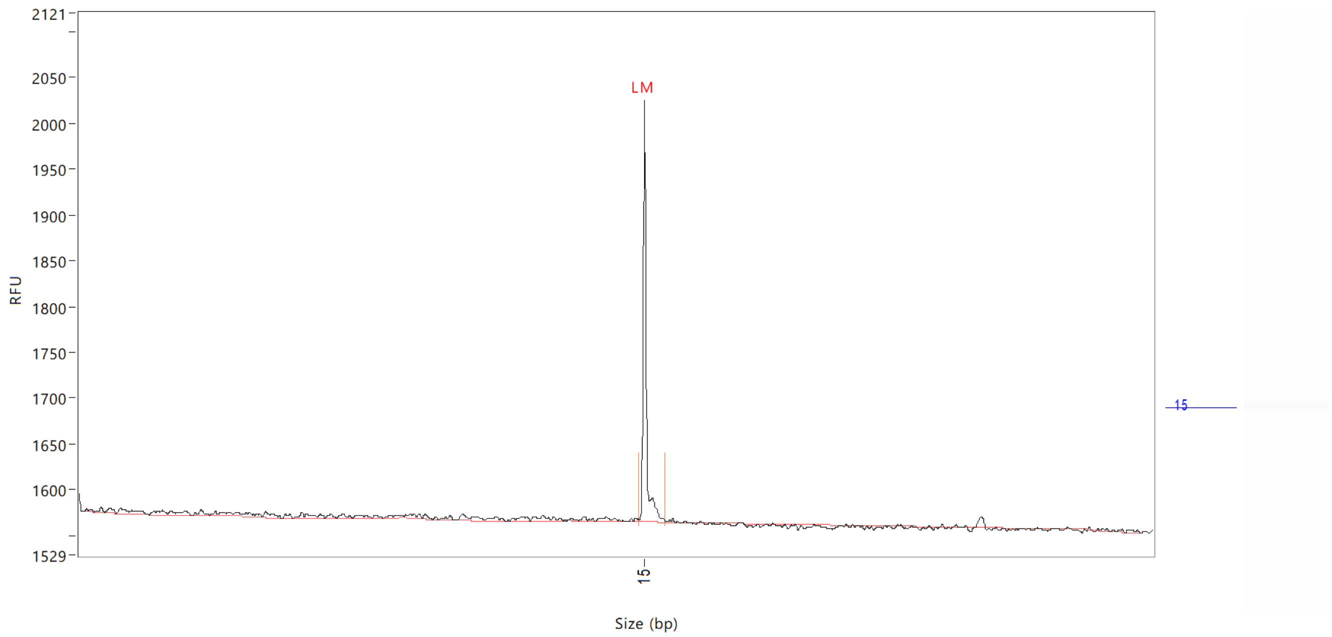

Sample Peak Width (sec): 6	Sample Min Peak Height: 50	Sample Baseline V to V?: Y	Sample Baseline V to V pts: 3
Sample filter: Binomial	Number of points for filter: 9	Sample start region (min): 0	Sample end region (min): 40
Manual baseline start (min): 18	Manual baseline end (min): 38	Marker peak width (sec): 6	Marker min peak height: 100
Marker baseline V to V?: Y	Marker baseline V to V points: 3	Lower marker selection: First peak > 100 RFU	Upper marker selection: Last peak > 100 RFU
Ladder size (bp)15, 200, 500, 1000, 1500, 2000, 3000, 4000, 6000	Quantification using: Ladder<br>Final concentration (ng/uL): 8.0000<br>Dilution factor: 12.0	Minimum RFU for data processing: 2	

Sample: SampA7
Well location: A7
Created: Wednesday, 4 June, 2025 12:10:03 PM

Table Information

Peak Table

Peak	Size	Concentration	From	To	Average size	Percent CV	RFU	Corrected peak area
	(bp)	(ng/uL)	(bp)	(bp)	(bp)			
1	15 (LM)	Nan	Nan	4075	Nan	Nan	459	2.945

TIC:	0.0000	ng/uL
TIM:	0.0000	nmole/L
Total concentration:	Nan	ng/uL

Sample: SampA8

Well location: A8

Created: Wednesday, 4 June, 2025 12:10:03 PM

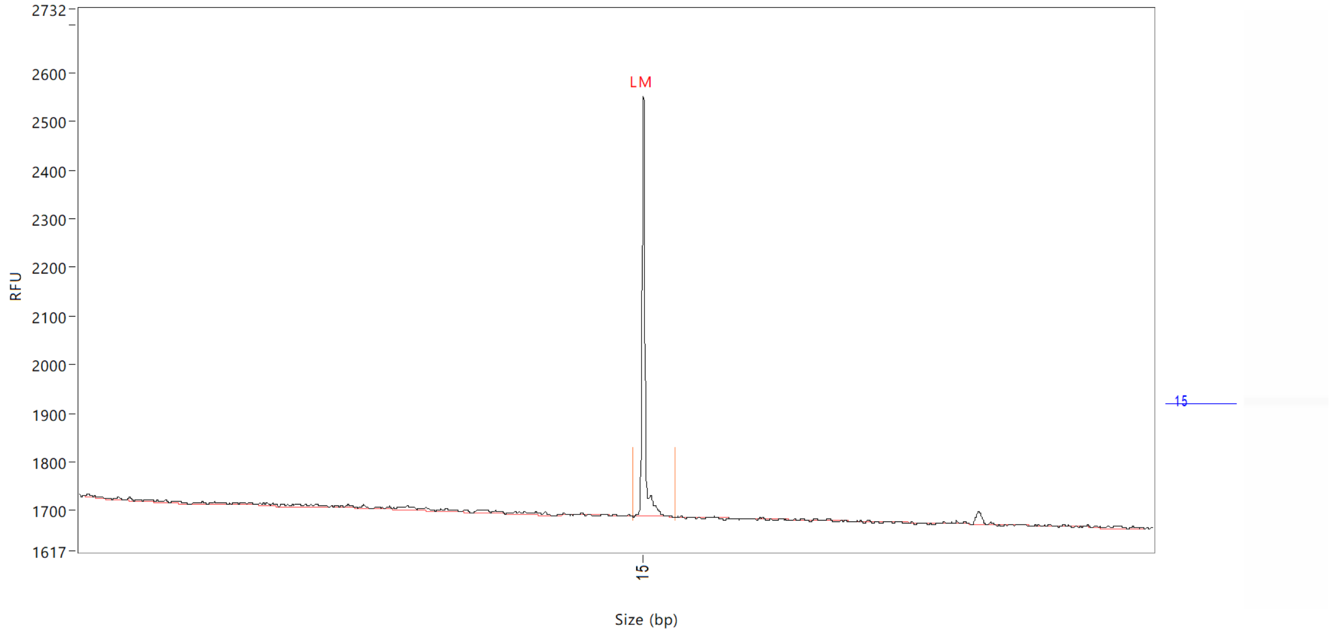

Sample Peak Width (sec): 6	Sample Min Peak Height: 50	Sample Baseline V to V?: Y	Sample Baseline V to V pts: 3
Sample filter: Binomial	Number of points for filter: 9	Sample start region (min): 0	Sample end region (min): 40
Manual baseline start (min): 18	Manual baseline end (min): 38	Marker peak width (sec): 6	Marker min peak height: 100
Marker baseline V to V?: Y	Marker baseline V to V points: 3	Lower marker selection: First peak > 100 RFU	Upper marker selection: Last peak > 100 RFU
Ladder size (bp)15, 200, 500, 1000, 1500, 2000, 3000, 4000, 6000	Quantification using: Ladder<br>Final concentration (ng/uL): 8.0000<br>Dilution factor: 12.0	Minimum RFU for data processing: 2	

Sample: SampA8
Well location: A8
Created: Wednesday, 4 June, 2025 12:10:03 PM

Table Information

Peak Table

Peak	Size	Concentration	From	To	Average size	Percent CV	RFU	Corrected peak area
	(bp)	(ng/uL)	(bp)	(bp)	(bp)			
1	15 (LM)	Nan	Nan	4115	Nan	Nan	863	5.497

TIC:	0.0000	ng/uL
TIM:	0.0000	nmole/L
Total concentration:	Nan	ng/uL

Sample: SampA9
Well location: A9
Created: Wednesday, 4 June, 2025 12:10:03 PM

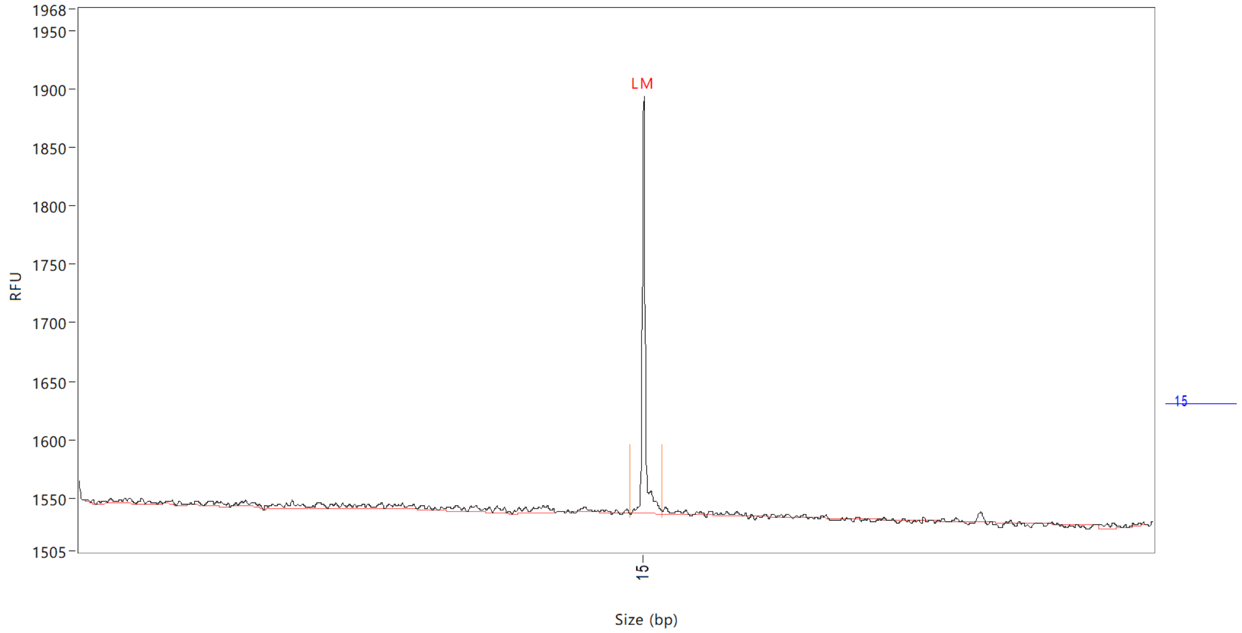

Sample Peak Width (sec): 6	Sample Min Peak Height: 50	Sample Baseline V to V?: Y	Sample Baseline V to V pts: 3
Sample filter: Binomial	Number of points for filter: 9	Sample start region (min): 0	Sample end region (min): 40
Manual baseline start (min): 18	Manual baseline end (min): 38	Marker peak width (sec): 6	Marker min peak height: 100
Marker baseline V to V?: Y	Marker baseline V to V points: 3	Lower marker selection: First peak > 100 RFU	Upper marker selection: Last peak > 100 RFU
Ladder size (bp)15, 200, 500, 1000, 1500, 2000, 3000, 4000, 6000	Quantification using: Ladder<br>Final concentration (ng/uL): 8.0000<br>Dilution factor: 12.0	Minimum RFU for data processing: 2	

Sample: SampA9
Well location: A9
Created: Wednesday, 4 June, 2025 12:10:03 PM

Table Information

Peak Table

Peak	Size	Concentration	From	To	Average size	Percent CV	RFU	Corrected peak area
	(bp)	(ng/uL)	(bp)	(bp)	(bp)			
1	15 (LM)	Nan	Nan	4066	Nan	Nan	355	2.280

TIC:	0.0000	ng/uL
TIM:	0.0000	nmole/L
Total concentration:	Nan	ng/uL

Sample: SampA10
Well location: A10
Created: Wednesday, 4 June, 2025 12:10:03 PM

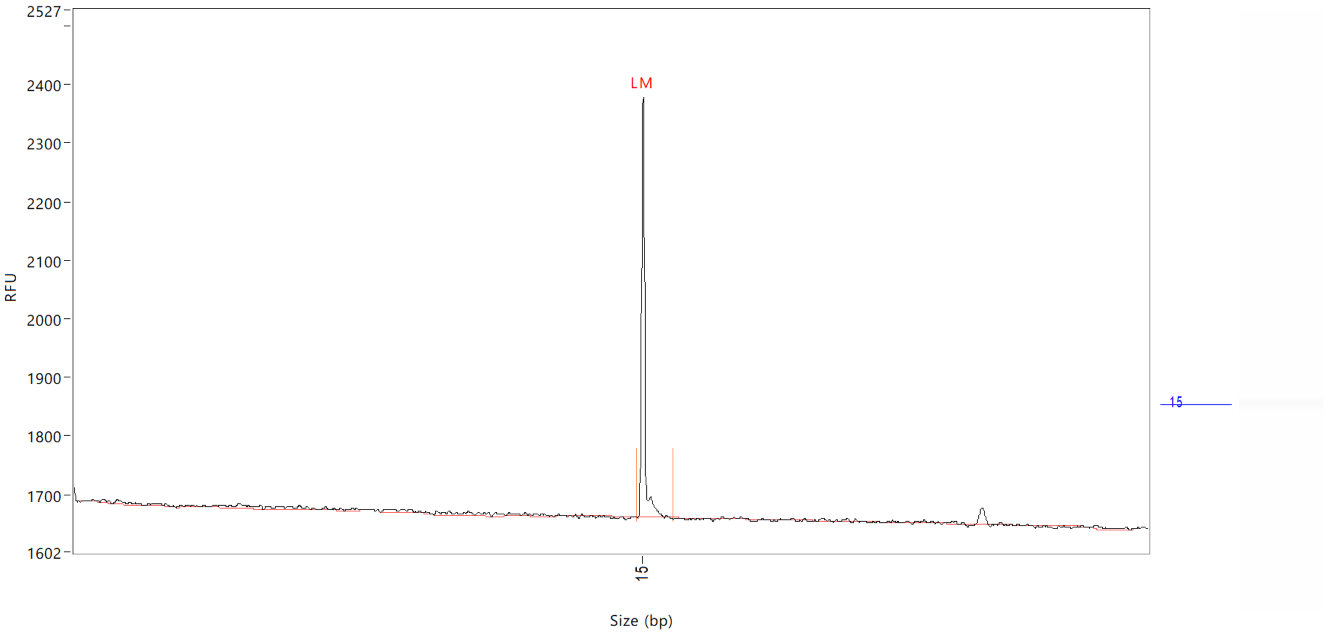

Sample Peak Width (sec): 6	Sample Min Peak Height: 50	Sample Baseline V to V?: Y	Sample Baseline V to V pts: 3
Sample filter: Binomial	Number of points for filter: 9	Sample start region (min): 0	Sample end region (min): 40
Manual baseline start (min): 18	Manual baseline end (min): 38	Marker peak width (sec): 6	Marker min peak height: 100
Marker baseline V to V?: Y	Marker baseline V to V points: 3	Lower marker selection: First peak > 100 RFU	Upper marker selection: Last peak > 100 RFU
Ladder size (bp)15, 200, 500, 1000, 1500, 2000, 3000, 4000, 6000	Quantification using: Ladder<br>Final concentration (ng/uL): 8.0000<br>Dilution factor: 12.0	Minimum RFU for data processing: 2	

Sample: SampA10
Well location: A10
Created: Wednesday, 4 June, 2025 12:10:03 PM

Table Information

Peak Table

Peak	Size	Concentration	From	To	Average size	Percent CV	RFU	Corrected peak area
	(bp)	(ng/uL)	(bp)	(bp)	(bp)			
1	15 (LM)	Nan	Nan	4105	Nan	Nan	715	4.479

TIC:	0.0000	ng/uL
TIM:	0.0000	nmole/L
Total concentration:	Nan	ng/uL

Sample: SampA11
Well location: A11
Created: Wednesday, 4 June, 2025 12:10:03 PM

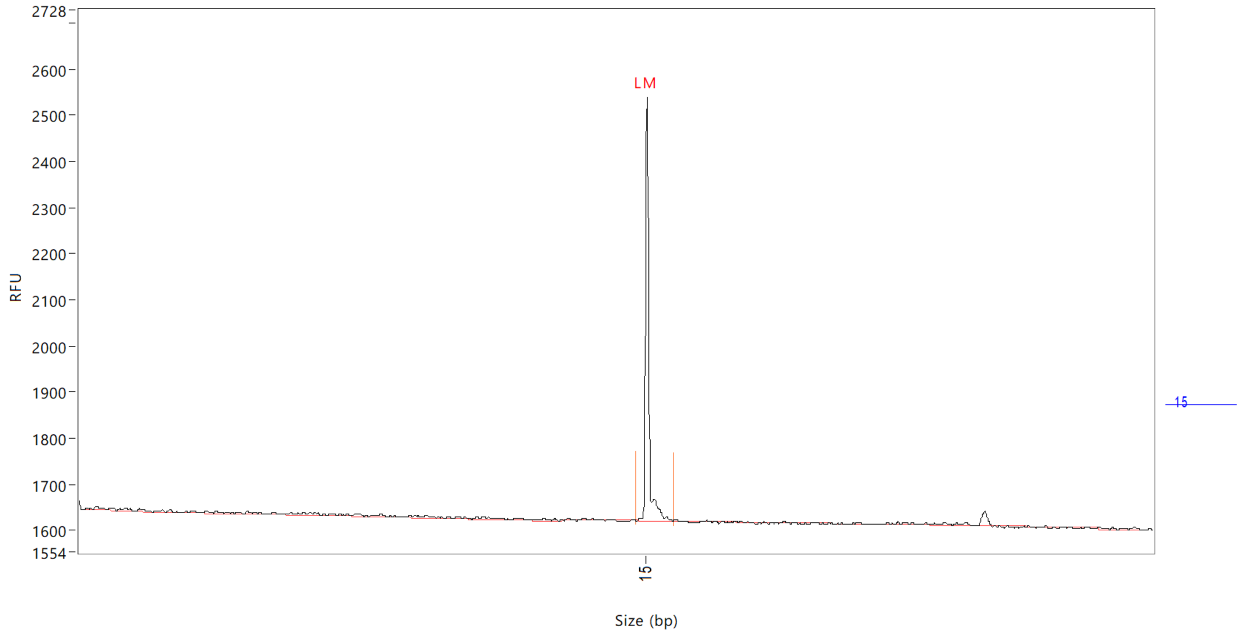

Sample Peak Width (sec): 6	Sample Min Peak Height: 50	Sample Baseline V to V?: Y	Sample Baseline V to V pts: 3
Sample filter: Binomial	Number of points for filter: 9	Sample start region (min): 0	Sample end region (min): 40
Manual baseline start (min): 18	Manual baseline end (min): 38	Marker peak width (sec): 6	Marker min peak height: 100
Marker baseline V to V?: Y	Marker baseline V to V points: 3	Lower marker selection: First peak > 100 RFU	Upper marker selection: Last peak > 100 RFU
Ladder size (bp)15, 200, 500, 1000, 1500, 2000, 3000, 4000, 6000	Quantification using: Ladder<br>Final concentration (ng/uL): 8.0000<br>Dilution factor: 12.0	Minimum RFU for data processing: 2	

Sample: SampA11
Well location: A11
Created: Wednesday, 4 June, 2025 12:10:03 PM

Table Information

Peak Table

Peak	Size	Concentration	From	To	Average size	Percent CV	RFU	Corrected peak area
	(bp)	(ng/uL)	(bp)	(bp)	(bp)			
1	15 (LM)	Nan	Nan	4096	Nan	Nan	917	5.919

TIC:	0.0000	ng/uL
TIM:	0.0000	nmole/L
Total concentration:	Nan	ng/uL

Sample: SampA12
Well location: A12
Created: Wednesday, 4 June, 2025 12:10:03 PM

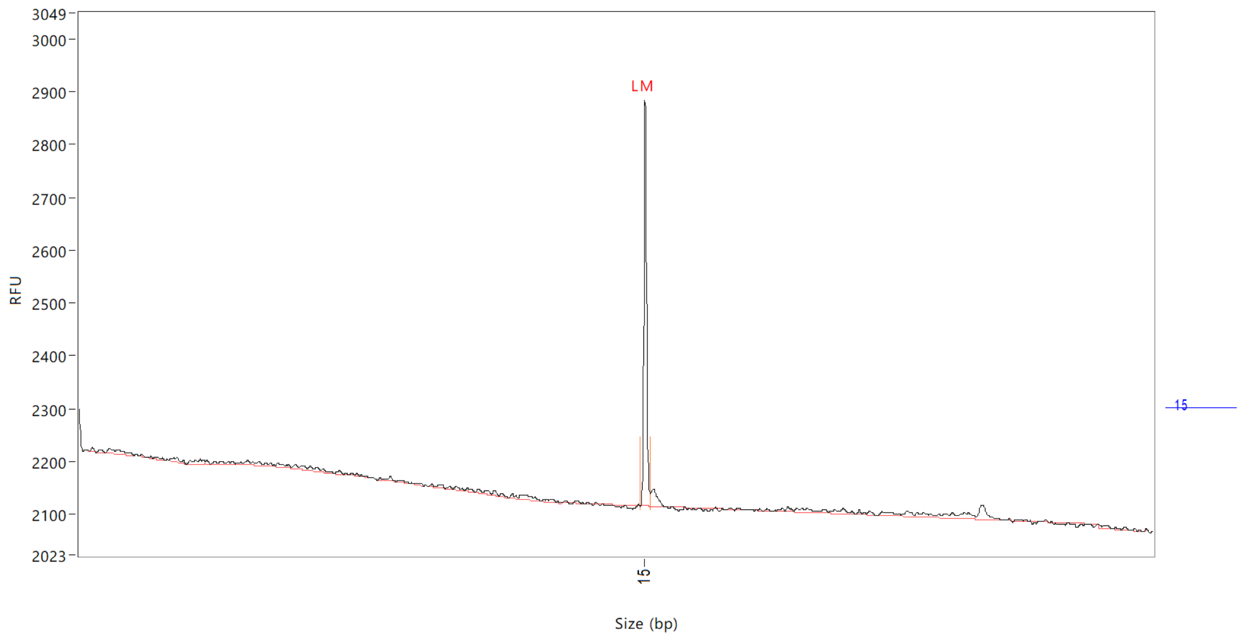

Sample Peak Width (sec): 6	Sample Min Peak Height: 50	Sample Baseline V to V?: Y	Sample Baseline V to V pts: 3
Sample filter: Binomial	Number of points for filter: 9	Sample start region (min): 0	Sample end region (min): 40
Manual baseline start (min): 18	Manual baseline end (min): 38	Marker peak width (sec): 6	Marker min peak height: 100
Marker baseline V to V?: Y	Marker baseline V to V points: 3	Lower marker selection: First peak > 100 RFU	Upper marker selection: Last peak > 100 RFU
Ladder size (bp)15, 200, 500, 1000, 1500, 2000, 3000, 4000, 6000	Quantification using: Ladder<br>Final concentration (ng/uL): 8.0000<br>Dilution factor: 12.0	Minimum RFU for data processing: 2	

Sample: SampA12
Well location: A12
Created: Wednesday, 4 June, 2025 12:10:03 PM

Table Information

Peak Table

Peak	Size	Concentration	From	To	Average size	Percent CV	RFU	Corrected peak area
	(bp)	(ng/uL)	(bp)	(bp)	(bp)			
1	15 (LM)	Nan	Nan	4018	Nan	Nan	767	4.397

TIC:	0.0000	ng/uL
TIM:	0.0000	nmole/L
Total concentration:	96.0000	ng/uL

Sample: SampA12
Well location: A12
Created: Wednesday, 4 June, 2025 12:10:03 PM
Fit type: Point to Point

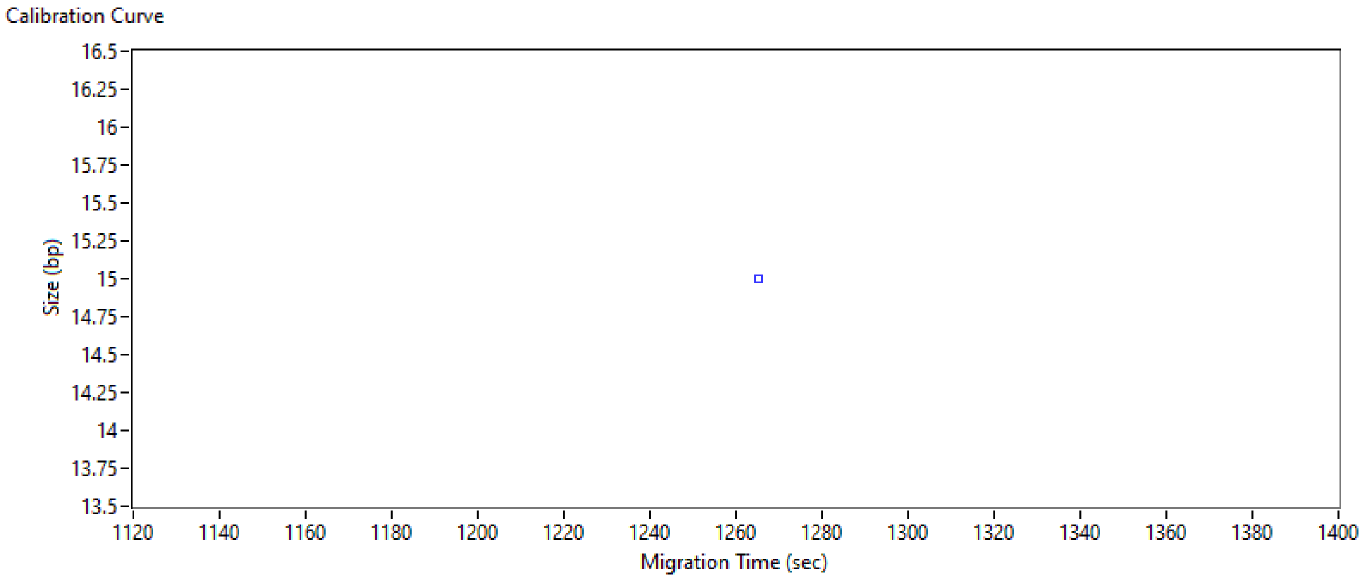

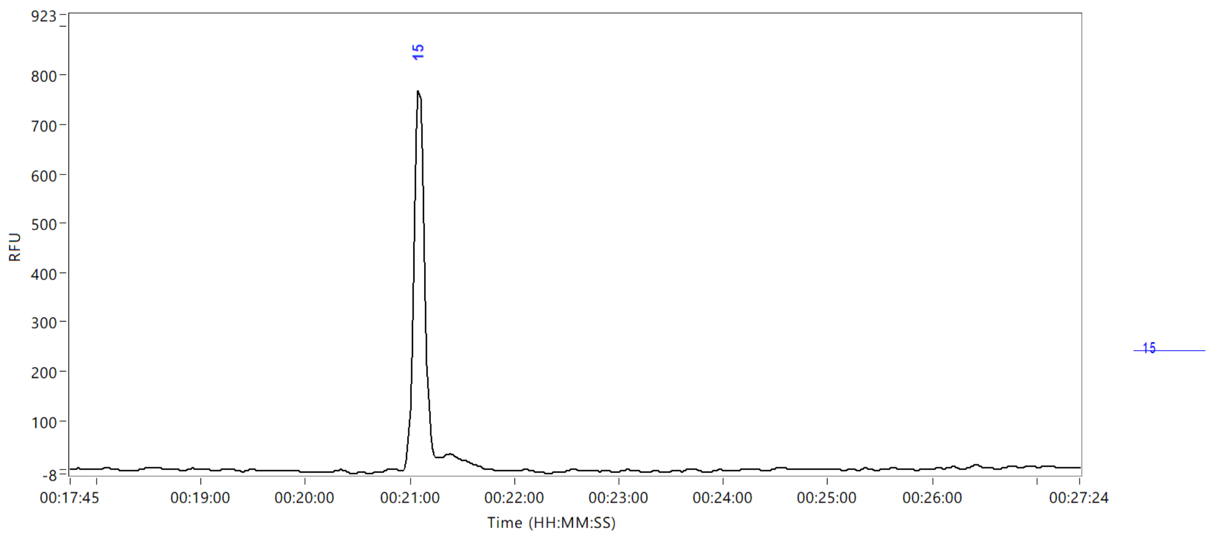

Supplement: S1 Data — Images files. SPR PDF files. CE5200 PDF file. S1D Fig report. S1A Fig sequencing file. (ZIP) [file ppat.1013741.s016.zip › Raw data/CE5200 PDF file/Fig 1C_CE.pdf]
