# Supplementary material for: A circular RNA vaccine induces durable and cross-protective immunity against Neisseria meningitidis serogroup B in mice
Source: PLoS Pathog. 2026 May 11;22(5):e1013741. doi: 10.1371/journal.ppat.1013741 (PMC13160355; doi:10.1371/journal.ppat.1013741)
Supplement: S1 Data — Images files. SPR PDF files. CE5200 PDF file. S1D Fig report. S1A Fig sequencing file. (ZIP) [file ppat.1013741.s016.zip › Raw data/S1D Fig report.docx]

**CGE Report**

**Precursor**

| LIF - Channel 1 Results |  |  |  |  |
| --- | --- | --- | --- | --- |
| Pk # | Time | Corrected Area | Corrected Area Percent | Resolution (USP) |
| 1 | 12.11 | 2361.25 | 0.04 | 0.00 |
| 2 | 12.20 | 2181.91 | 0.03 | 2.11 |
| 3 | 17.02 | 2124562.30 | 32.31 | 4.06 |
| 4 | 18.57 | 4383989.68 | 66.68 | 1.28 |
| 5 | 18.79 | 62005.82 | 0.94 | 0.56 |
|  |  |  |  |  |
| Totals |  |  |  |  |
|  |  | 6575100.95 | 100.00 |  |

**CGE Report**

**IVT**

| LIF - Channel 1 Results |  |  |  |  |
| --- | --- | --- | --- | --- |
| Pk # | Time | Corrected Area | Corrected Area Percent | Resolution (USP) |
| 1 | 9.91 | 4343.62 | 0.01 | 0.00 |
| 2 | 10.53 | 33764.97 | 0.11 | 10.31 |
| 3 | 11.29 | 89284.02 | 0.29 | 13.01 |
| 4 | 18.46 | 10228829.69 | 33.74 | 25.28 |
| 5 | 20.09 | 12681760.30 | 41.83 | 4.38 |
| 6 | 21.35 | 6018796.91 | 19.85 | 1.96 |
| 7 | 24.16 | 12644.17 | 0.04 | 3.89 |
| 8 | 25.52 | 47209.52 | 0.16 | 2.14 |
| 9 | 30.25 | 1201646.13 | 3.96 | 4.11 |
|  |  |  |  |  |
| Totals |  |  |  |  |
|  |  | 30318279.32 | 100.00 |  |

**CGE Report**

**IVT+RNR**

| LIF - Channel 1 Results |  |  |  |  |
| --- | --- | --- | --- | --- |
| Pk # | Time | Corrected Area | Corrected Area Percent | Resolution (USP) |
| 1 | 9.87 | 9446.28 | 0.04 | 0.00 |
| 2 | 10.50 | 29207.05 | 0.11 | 9.30 |
| 3 | 10.77 | 7215.91 | 0.03 | 2.09 |
| 4 | 11.02 | 16996.13 | 0.07 | 1.39 |
| 5 | 11.23 | 28425.57 | 0.11 | 1.18 |
| 6 | 11.51 | 12871.82 | 0.05 | 1.87 |
| 7 | 18.46 | 7062221.05 | 27.29 | 4.33 |
| 8 | 20.13 | 15034380.30 | 58.10 | 1.01 |
| 9 | 21.38 | 2838184.28 | 10.97 | 0.34 |
| 10 | 30.28 | 839296.11 | 3.24 | 2.10 |
|  |  |  |  |  |
| Totals |  |  |  |  |
|  |  | 25878244.49 | 100.00 |  |

**CGE Report**

**IVT+RNR+SEC**

| LIF - Channel 1 Results |  |  |  |  |
| --- | --- | --- | --- | --- |
| Pk # | Time | Corrected Area | Corrected Area Percent | Resolution (USP) |
| 1 | 13.07 | 4780.01 | 0.03 | 0.00 |
| 2 | 18.57 | 3726183.01 | 21.96 | 4.63 |
| 3 | 20.24 | 12061753.87 | 71.10 | 1.33 |
| 4 | 21.09 | 1067956.23 | 6.30 | 1.05 |
| 5 | 30.38 | 103702.96 | 0.61 | 6.80 |
|  |  |  |  |  |
| Totals |  |  |  |  |
|  |  | 16964376.08 | 100.00 |  |

**CGE Report**

**Nicked**

| LIF - Channel 1 Results |  |  |  |  |
| --- | --- | --- | --- | --- |
| Pk # | Time | Corrected Area | Corrected Area Percent | Resolution (USP) |
| 1 | 12.42 | 7435.02 | 0.07 | 0.00 |
| 2 | 12.83 | 2367.01 | 0.02 | 3.99 |
| 3 | 13.14 | 6705.90 | 0.06 | 3.23 |
| 4 | 16.49 | 1338798.52 | 12.13 | 3.47 |
| 5 | 17.55 | 9127259.78 | 82.71 | 1.11 |
| 6 | 17.90 | 544628.31 | 4.94 | 0.76 |
| 7 | 37.56 | 8102.17 | 0.07 | 44.96 |
|  |  |  |  |  |
| Totals |  |  |  |  |
|  |  | 11035296.70 | 100.00 |  |
