# Supplementary material for: A circular RNA vaccine induces durable and cross-protective immunity against Neisseria meningitidis serogroup B in mice
Source: PLoS Pathog. 2026 May 11;22(5):e1013741. doi: 10.1371/journal.ppat.1013741 (PMC13160355; doi:10.1371/journal.ppat.1013741)
Supplement: S1 Data — Images files. SPR PDF files. CE5200 PDF file. S1D Fig report. S1A Fig sequencing file. (ZIP) [file ppat.1013741.s016.zip › Raw data/SPR PDF files/FAB 12C1-5 VB16T13 ECOLI-6 VB16T13 293F-7 VB16T13 3t3-20260205 252026 65829 PM.pdf]

Biacore Insight Evaluation

FAB 12C1-5 VB16T13 ECOLI-6 VB16T13 293F-7 VB16T13 3t3-20260205 2/5/2026 6:58:29 PM

Evaluation

Name	N/A (unsaved evaluation)
Path	N/A (unsaved evaluation)
Modified by	()
Date modified	
Created with	Biacore Insight Evaluation 4.0.8.19879
Server name	.\SQLEXPRESS
Database id	Biacore Insight Database

Run(s) in this evaluation

Run 1

Name	FAB 12C1-5 VB16T13 ECOLI-6 VB16T13 293F-7 VB16T13 3t3-20260205 2/5/2026 6:58:29 PM
Path	Root\ZW\QIQI ZHANG\FAB 12C1-5 VB16T13 ECOLI-6 VB16T13 293F-7 VB16T13 3t3-20260205 2/5/2026 6:58:29 PM
Created by	DESKTOP-22AQC8N\Dell (DESKTOP-22AQC8N\Dell)
Method	FAB 12C1-5 VB16T13 ECOLI-6 VB16T13 293F-7 VB16T13 3t3-20260205
Instrument type/ID	Biacore 8K+/2902034
Cycles	10
Data collection rate	10 Hz
Running buffer	Buffer
Start time	2/6/2026 7:08:55 AM
End time	2/6/2026 9:31:43 AM

Chip information

Chip id	10/20/2025 1:22:02 PM
Chip type	CM5
Lot number	10369187
First dock date	10/20/2025 1:23:00 PM

Run 1 - Immobilization results

Flow cell 1

	Channel 1	Channel 2	Channel 3	Channel 4
Sensorgram type	Not used in run	Not used in run	Not used in run	Not used in run
Ligand			SA	SA
MW (Da)				
Level (RU)			6142.6	6088.1
Run name			Immobilization 1/8/2026 10:43:22 AM	Immobilization 1/8/2026 10:43:22 AM
Date and time			1/8/2026 10:44:03 AM	1/8/2026 10:44:03 AM

	Channel 5	Channel 6	Channel 7	Channel 8
Sensorgram type	Reference	Reference	Reference	Not used in run
Ligand	Activation/Deactivation	Activation/Deactivation	Activation/Deactivation	
MW (Da)				
Level (RU)				
Run name	Immobilization 20260205 12:22:47 PM 2/5/2026 6:14:22 PM	Immobilization 20260205 12:22:47 PM 2/5/2026 6:14:22 PM	Immobilization 20260205 12:22:47 PM 2/5/2026 6:14:22 PM	
Date and time	2/5/2026 6:14:26 PM	2/5/2026 6:14:26 PM	2/5/2026 6:14:26 PM	

Run 1 - Immobilization results

Flow cell 2

	Channel 1	Channel 2	Channel 3	Channel 4
Sensorgram type	Not used in run	Not used in run	Not used in run	Not used in run
Ligand	Ligand 1	Ligand 2	SA	SA
MW (Da)				
Level (RU)	3483.5	5468.2	5936.3	6024.2
Run name	Immobilization 10/20/2025 1:40:59 PM	Immobilization 10/20/2025 1:40:59 PM	Immobilization 1/8/2026 10:43:22 AM	Immobilization 1/8/2026 10:43:22 AM
Date and time	10/20/2025 1:41:04 PM	10/20/2025 1:41:04 PM	1/8/2026 10:44:03 AM	1/8/2026 10:44:03 AM

	Channel 5	Channel 6	Channel 7	Channel 8
Sensorgram type	Active	Active	Active	Not used in run
Ligand	VB16T13 ECOLI	VB16T13 293F	VB16T13 3T3	
MW (Da)				
Level (RU)	288.4	256.3	323.2	
Run name	Immobilization 20260205 12:22:47 PM 2/5/2026 6:14:22 PM	Immobilization 20260205 12:22:47 PM 2/5/2026 6:14:22 PM	Immobilization 20260205 12:22:47 PM 2/5/2026 6:14:22 PM	
Date and time	2/5/2026 6:14:26 PM	2/5/2026 6:14:26 PM	2/5/2026 6:14:26 PM	

Run 1 - Method summary

2 Startup

Analyte 1

Buffer, Contact time 120 s, Diss time 60 s, Flow rate 30 µl/min, Both flow cells

Regeneration 1

1.5, Contact time 30 s, Flow rate 30 µl/min, Both flow cells

8 Analysis

Analyte 1

Variable solution, Contact time 120 s, Diss time 600 s, Flow rate 30 µl/min, Variable conc, Both flow cells

Regeneration 1

1.5, Contact time 90 s, Flow rate 30 µl/min, Both flow cells

QC - Sensorgram

Thumbnails

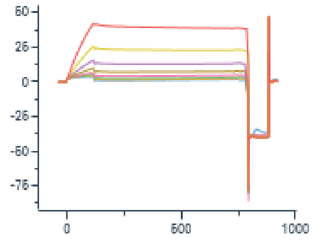

Channel 5

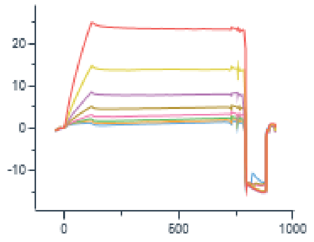

Channel 6

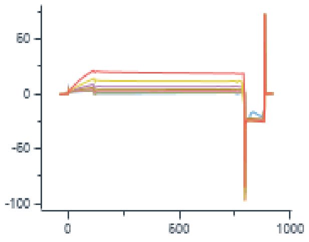

Channel 7

Charts

Channel 5

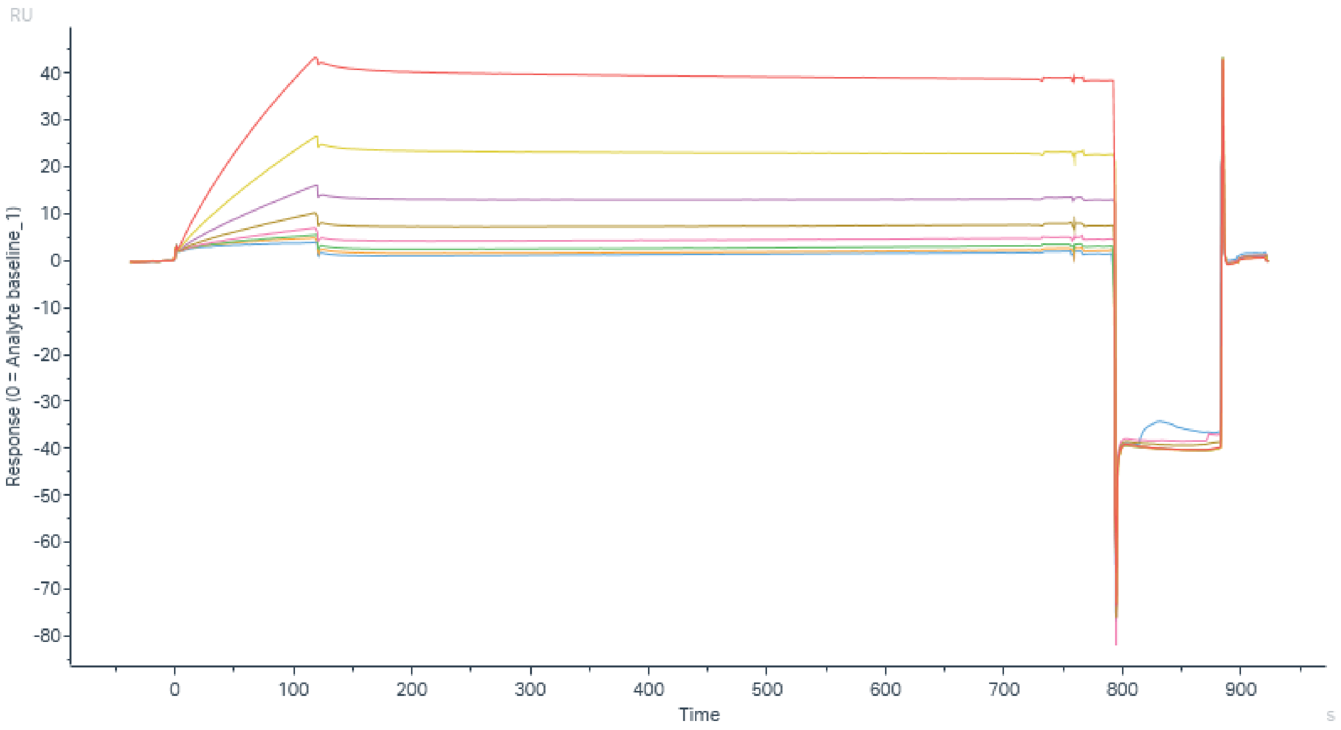

Channel 6

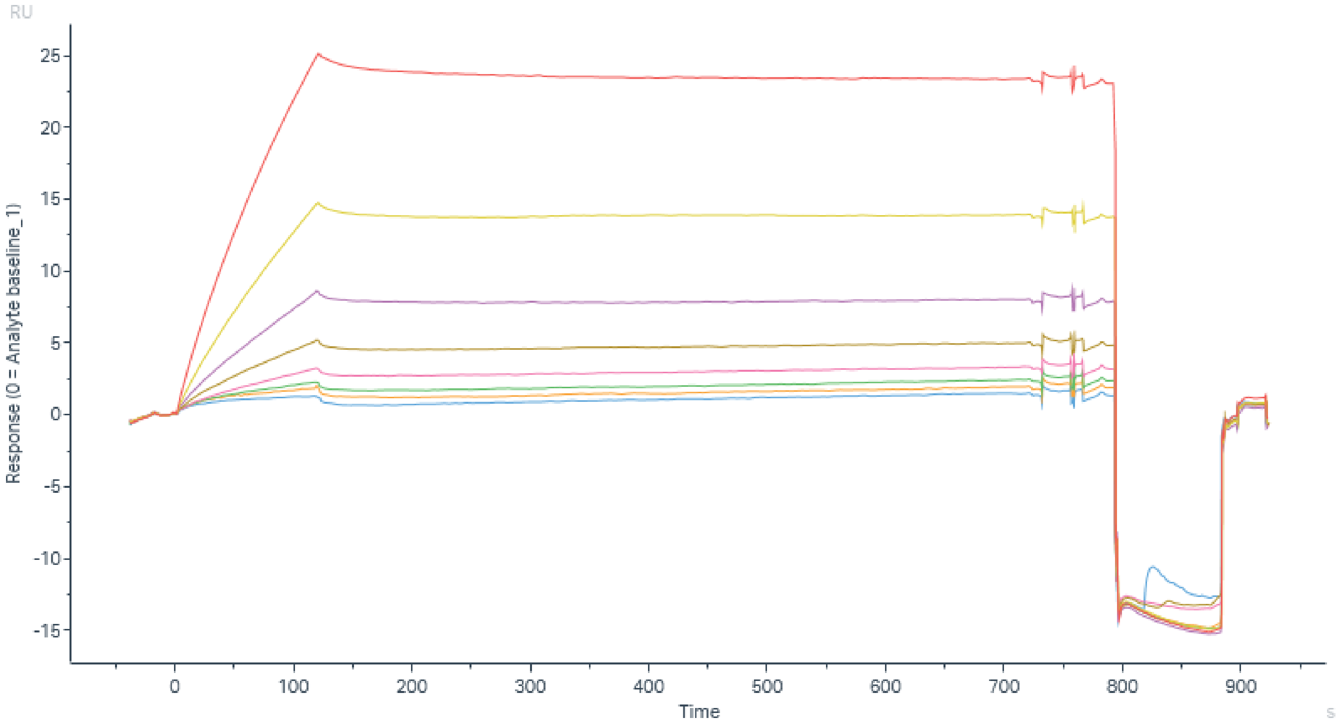

Channel 7

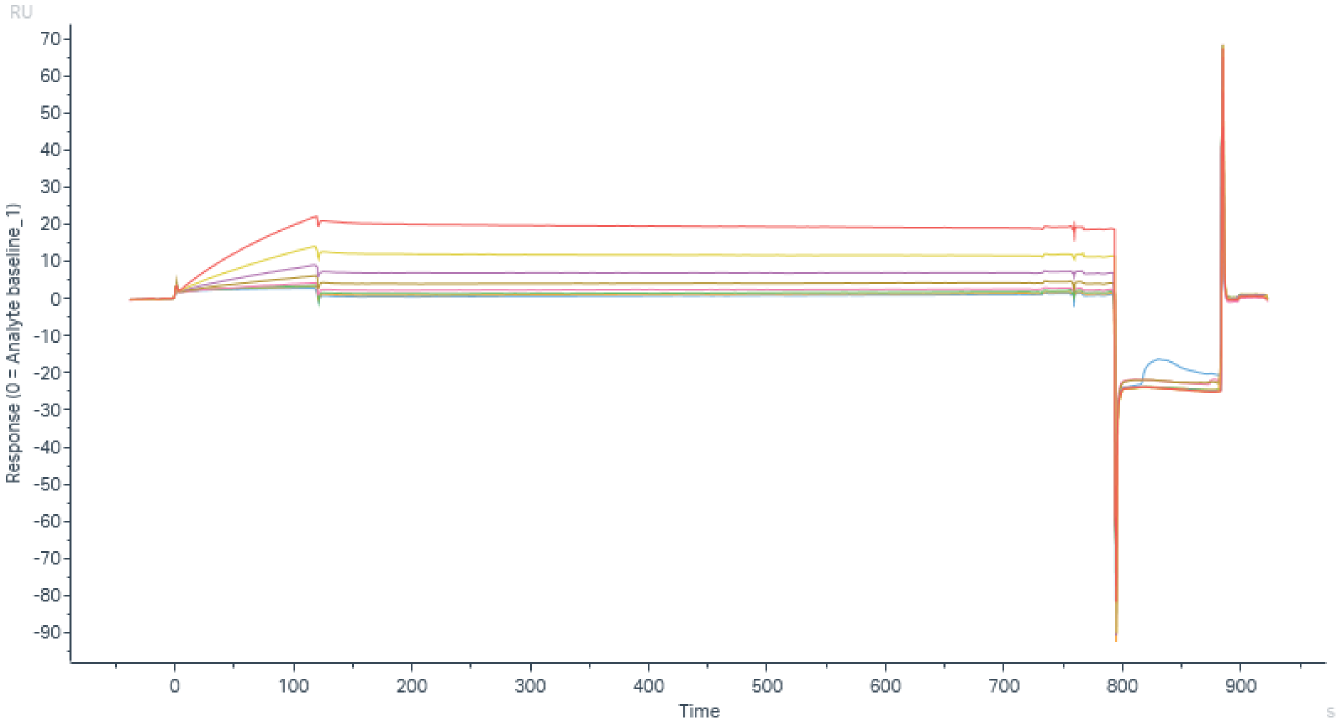
RU
70
60
50
40
30
20
10
0
-10
-20
-30
-40
-50
-60
-70
-80
-90
Response (0 = Analyte baseline_1)
0
100
200
300
400
500
600
700
800
900
Time
s

Sensorgram table

#	Cycle	Channel	Sensorgram type	Analysis step purpose	Analysis step name	Excluded
1	3	5	Reference subtracted	Analysis	Analysis	
2	4	5	Reference subtracted	Analysis	Analysis	
3	5	5	Reference subtracted	Analysis	Analysis	
4	6	5	Reference subtracted	Analysis	Analysis	
5	7	5	Reference subtracted	Analysis	Analysis	
6	8	5	Reference subtracted	Analysis	Analysis	
7	9	5	Reference subtracted	Analysis	Analysis	
8	10	5	Reference subtracted	Analysis	Analysis	
9	3	6	Reference subtracted	Analysis	Analysis	
10	4	6	Reference subtracted	Analysis	Analysis	
11	5	6	Reference subtracted	Analysis	Analysis	
12	6	6	Reference subtracted	Analysis	Analysis	
13	7	6	Reference subtracted	Analysis	Analysis	
14	8	6	Reference subtracted	Analysis	Analysis	
15	9	6	Reference subtracted	Analysis	Analysis	
16	10	6	Reference subtracted	Analysis	Analysis	
17	3	7	Reference subtracted	Analysis	Analysis	
18	4	7	Reference subtracted	Analysis	Analysis	
19	5	7	Reference subtracted	Analysis	Analysis	
20	6	7	Reference subtracted	Analysis	Analysis	
21	7	7	Reference subtracted	Analysis	Analysis	
22	8	7	Reference subtracted	Analysis	Analysis	
23	9	7	Reference subtracted	Analysis	Analysis	
24	10	7	Reference subtracted	Analysis	Analysis	

Sensorgram table (continued)

#	Curve markers	Analyte 1 Solution	Analyte 1 Concentration (nM)	Analyte binding late_1 Relative (RU)
1		FAB 12C1	0	3.9
2		FAB 12C1	3.175	4.8
3		FAB 12C1	6.25	5.5
4		FAB 12C1	12.5	6.9
5		FAB 12C1	25	10.0
6		FAB 12C1	50	15.7
7		FAB 12C1	100	25.9
8		FAB 12C1	200	42.5
9		FAB 12C1	0	1.3
10		FAB 12C1	3.175	1.8
11		FAB 12C1	6.25	2.2
12		FAB 12C1	12.5	3.2
13		FAB 12C1	25	5.0
14		FAB 12C1	50	8.3
15		FAB 12C1	100	14.2
16		FAB 12C1	200	24.3
17		FAB 12C1	0	2.8
18		FAB 12C1	3.175	3.3
19		FAB 12C1	6.25	3.5
20		FAB 12C1	12.5	4.2
21		FAB 12C1	25	6.0
22		FAB 12C1	50	8.9
23		FAB 12C1	100	13.9
24		FAB 12C1	200	21.7

QC - Baseline

Thumbnails

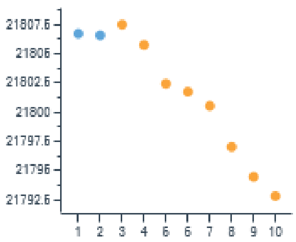

Channel 5

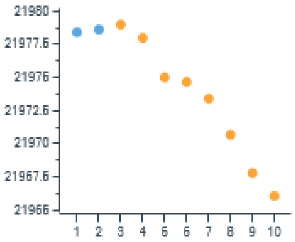

Channel 6

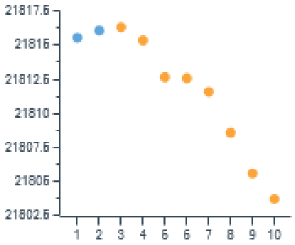

Channel 7

Charts

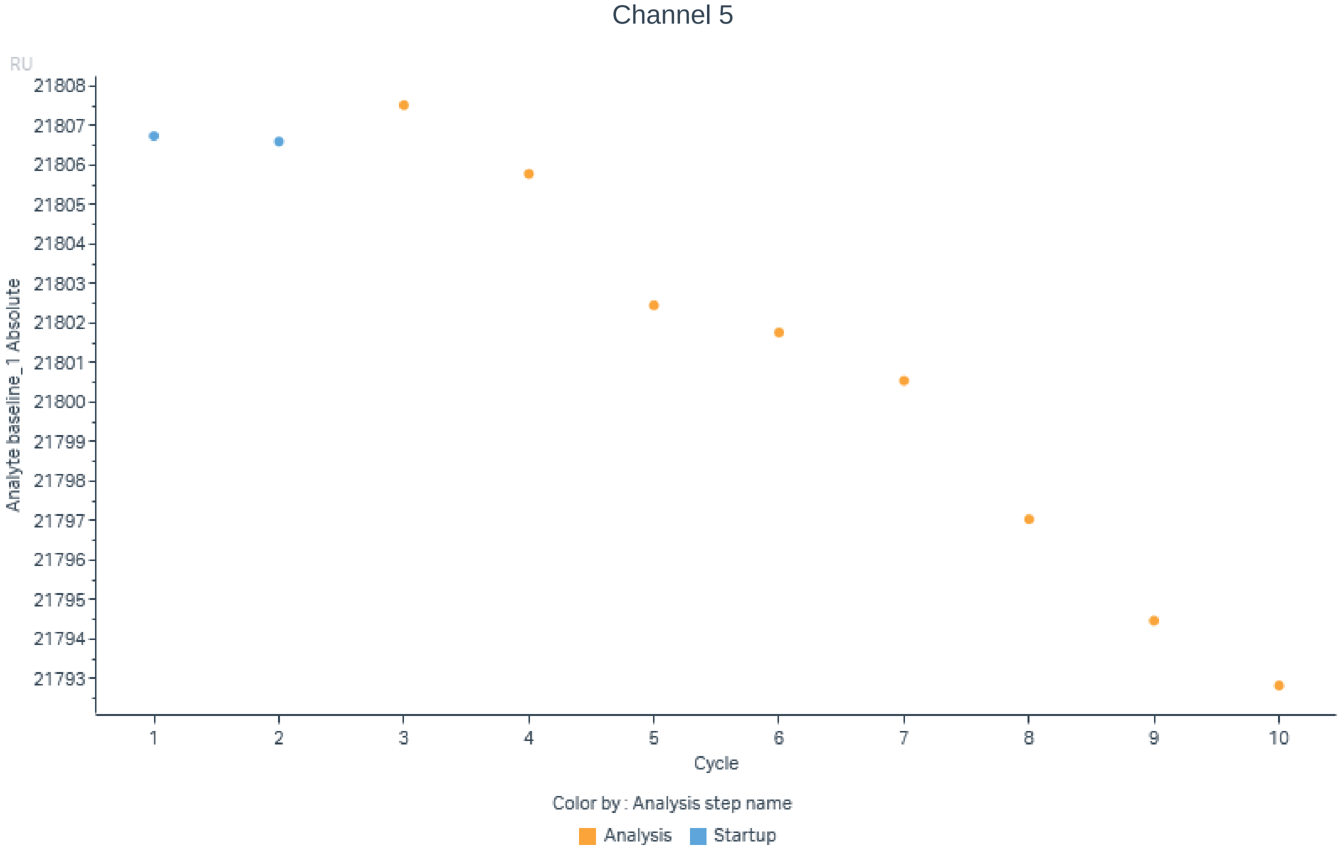
Channel 5
RU
21808
21807
21806
21805
21804
21803
21802
21801
21800
21799
21798
21797
21796
21795
21794
21793
Analyte baseline_1 Absolute
1
2
3
4
5
6
7
8
9
10
Cycle
Color by : Analysis step name
Analysis
Startup

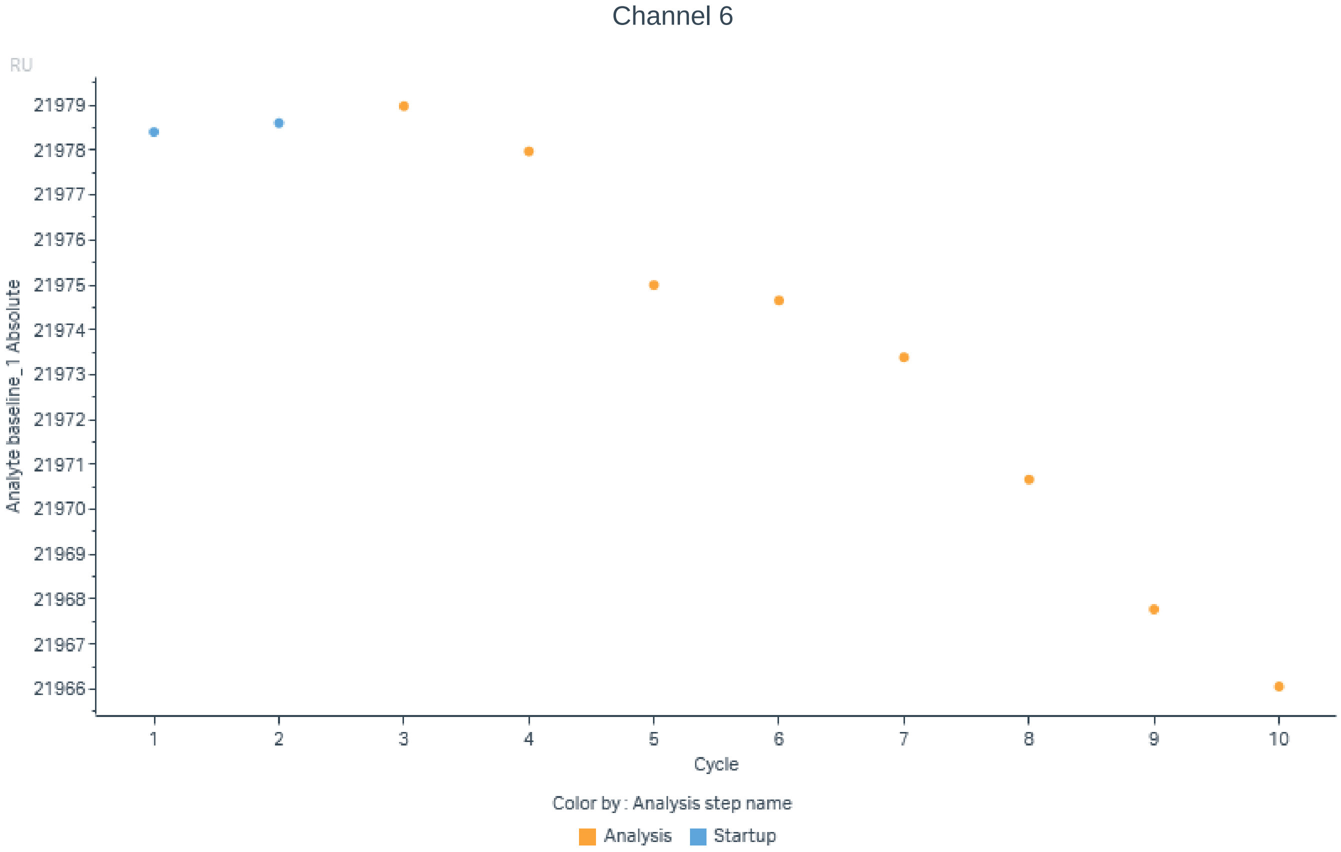
Channel 6
RU
21979
21978
21977
21976
21975
21974
21973
21972
21971
21970
21969
21968
21967
21966
Analyte baseline_1 Absolute
1
2
3
4
5
6
7
8
9
10
Cycle
Color by : Analysis step name
Analysis
Startup

Channel 7

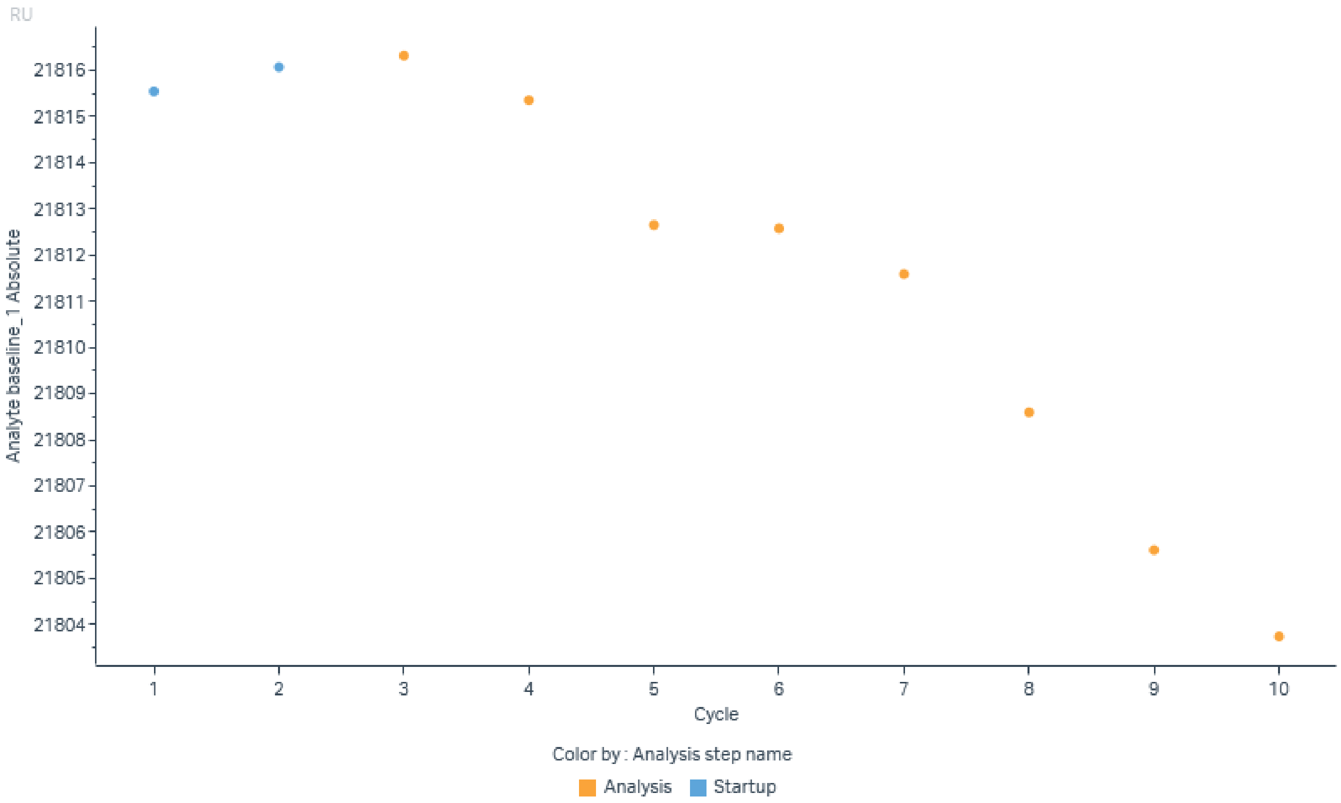
RU
Analyte baseline_1 Absolute
21816
21815
21814
21813
21812
21811
21810
21809
21808
21807
21806
21805
21804
1
2
3
4
5
6
7
8
9
10
Cycle
Color by : Analysis step name
Analysis
Startup

Plot table

#	Cycle	Channel	Sensorgram type	Analysis step purpose	Analysis step name	Excluded
1	1	5	Active	Startup	Startup	
2	2	5	Active	Startup	Startup	
3	3	5	Active	Analysis	Analysis	
4	4	5	Active	Analysis	Analysis	
5	5	5	Active	Analysis	Analysis	
6	6	5	Active	Analysis	Analysis	
7	7	5	Active	Analysis	Analysis	
8	8	5	Active	Analysis	Analysis	
9	9	5	Active	Analysis	Analysis	
10	10	5	Active	Analysis	Analysis	
11	1	6	Active	Startup	Startup	
12	2	6	Active	Startup	Startup	
13	3	6	Active	Analysis	Analysis	
14	4	6	Active	Analysis	Analysis	
15	5	6	Active	Analysis	Analysis	
16	6	6	Active	Analysis	Analysis	
17	7	6	Active	Analysis	Analysis	
18	8	6	Active	Analysis	Analysis	
19	9	6	Active	Analysis	Analysis	
20	10	6	Active	Analysis	Analysis	
21	1	7	Active	Startup	Startup	
22	2	7	Active	Startup	Startup	
23	3	7	Active	Analysis	Analysis	
24	4	7	Active	Analysis	Analysis	
25	5	7	Active	Analysis	Analysis	
26	6	7	Active	Analysis	Analysis	
27	7	7	Active	Analysis	Analysis	
28	8	7	Active	Analysis	Analysis	
29	9	7	Active	Analysis	Analysis	
30	10	7	Active	Analysis	Analysis	

Plot table (continued)

#	Curve markers	Analyte 1 Solution	Analyte baseline_1 Absolute (RU)
1		Buffer	21806.7
2		Buffer	21806.6
3		FAB 12C1	21807.5
4		FAB 12C1	21805.8
5		FAB 12C1	21802.4
6		FAB 12C1	21801.8
7		FAB 12C1	21800.5
8		FAB 12C1	21797.0
9		FAB 12C1	21794.5
10		FAB 12C1	21792.8
11		Buffer	21978.4
12		Buffer	21978.6
13		FAB 12C1	21979.0
14		FAB 12C1	21978.0
15		FAB 12C1	21975.0
16		FAB 12C1	21974.7
17		FAB 12C1	21973.4
18		FAB 12C1	21970.7
19		FAB 12C1	21967.8
20		FAB 12C1	21966.1
21		Buffer	21815.6
22		Buffer	21816.1
23		FAB 12C1	21816.3
24		FAB 12C1	21815.4
25		FAB 12C1	21812.7
26		FAB 12C1	21812.6
27		FAB 12C1	21811.6
28		FAB 12C1	21808.6
29		FAB 12C1	21805.6
30		FAB 12C1	21803.7

QC - Binding to reference

Thumbnails

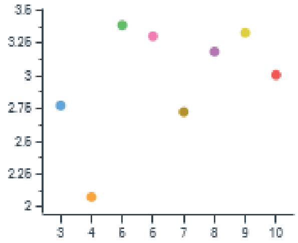

Channel 5

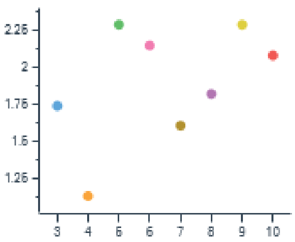

Channel 6

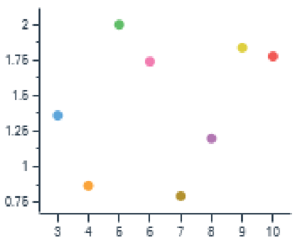

Channel 7

Charts

Channel 5

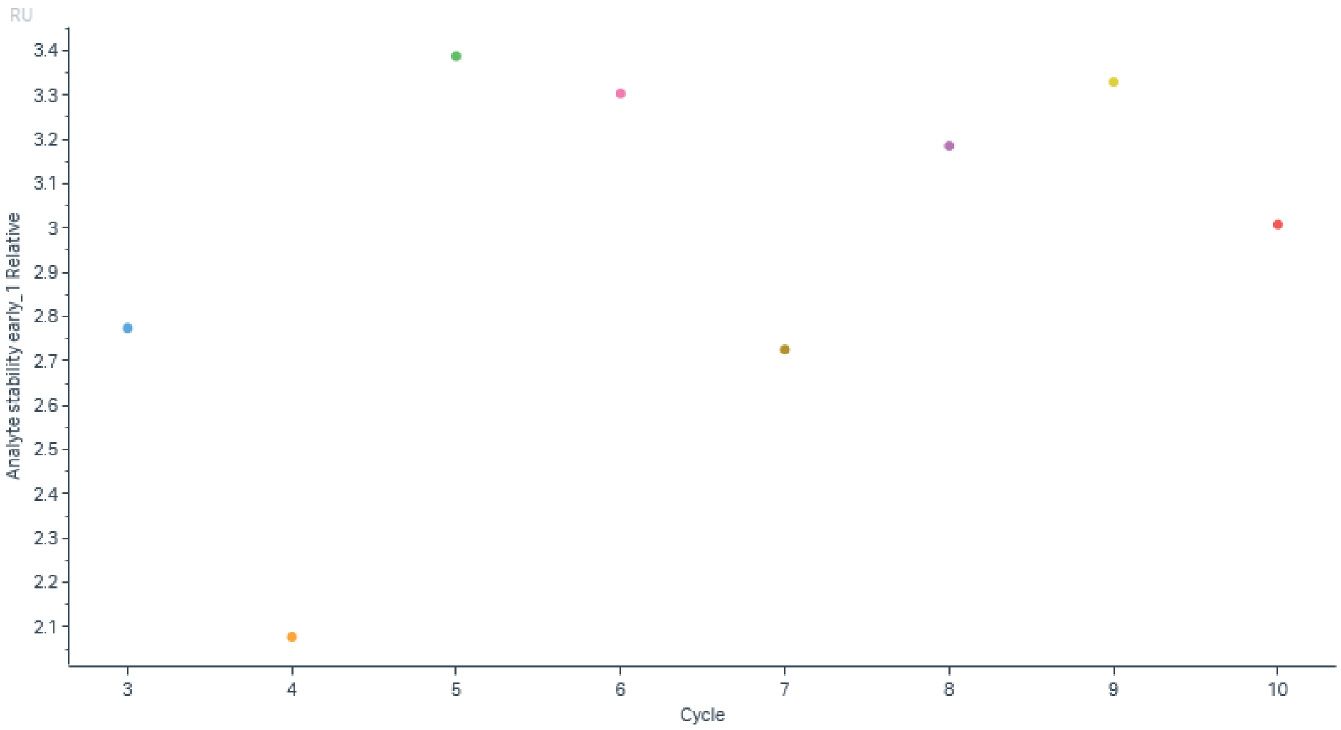

Channel 6

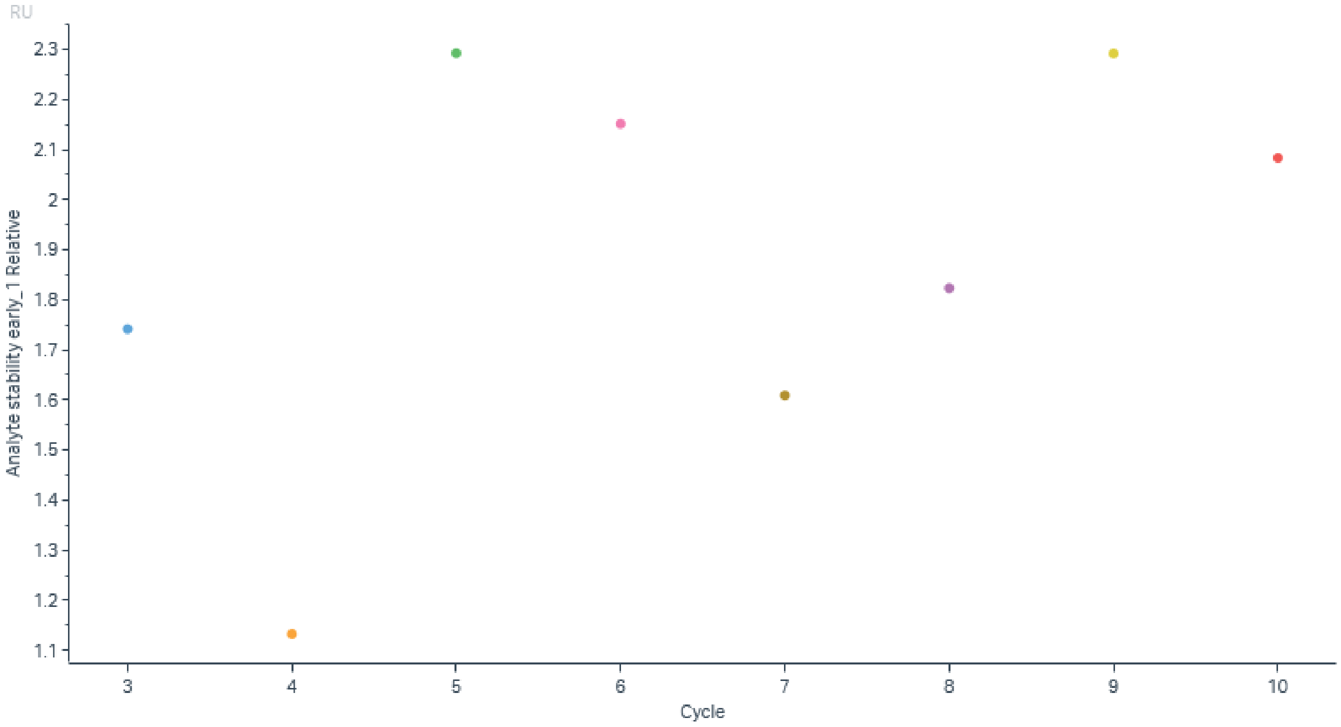

Channel 7

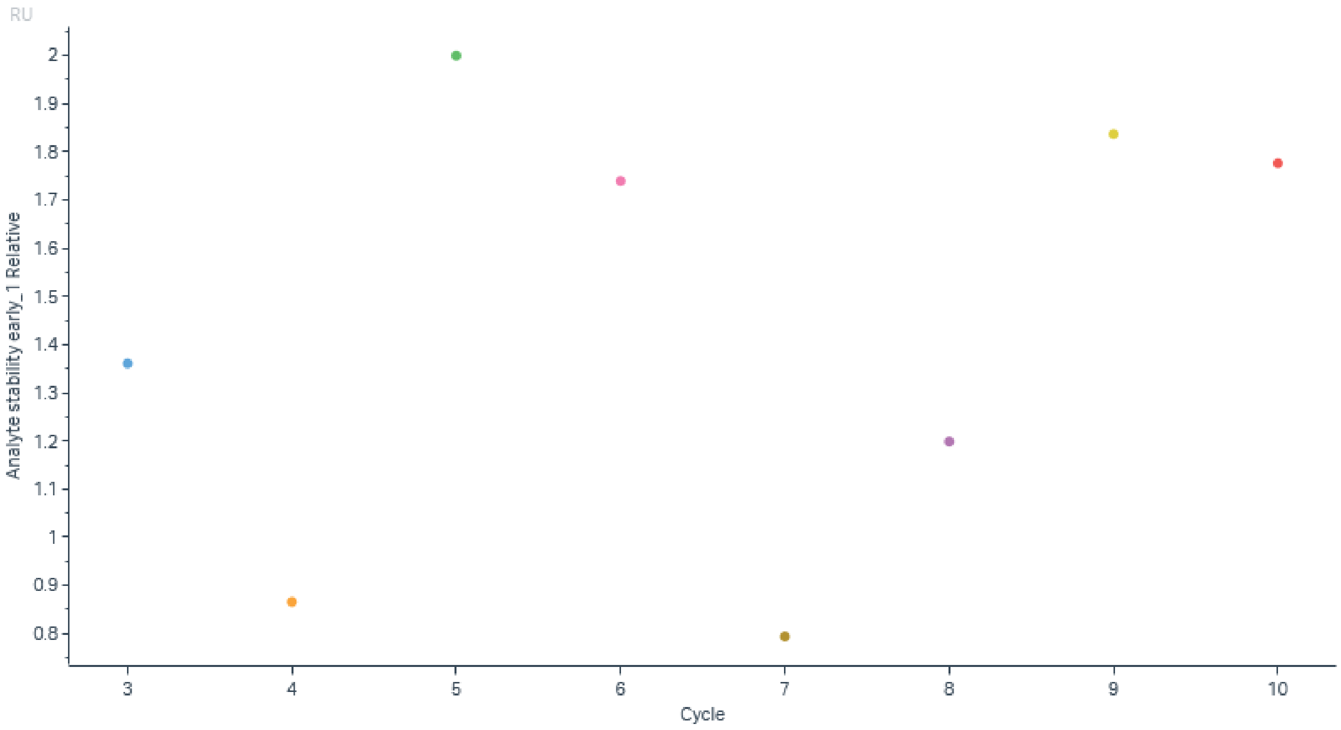
RU
Analyte stability early_1 Relative
2
1.9
1.8
1.7
1.6
1.5
1.4
1.3
1.2
1.1
1
0.9
0.8
3
4
5
6
7
8
9
10
Cycle

Plot table

#	Cycle	Channel	Sensorgram type	Analysis step purpose	Analysis step name	Excluded
1	3	5	Reference	Analysis	Analysis	
2	4	5	Reference	Analysis	Analysis	
3	5	5	Reference	Analysis	Analysis	
4	6	5	Reference	Analysis	Analysis	
5	7	5	Reference	Analysis	Analysis	
6	8	5	Reference	Analysis	Analysis	
7	9	5	Reference	Analysis	Analysis	
8	10	5	Reference	Analysis	Analysis	
9	3	6	Reference	Analysis	Analysis	
10	4	6	Reference	Analysis	Analysis	
11	5	6	Reference	Analysis	Analysis	
12	6	6	Reference	Analysis	Analysis	
13	7	6	Reference	Analysis	Analysis	
14	8	6	Reference	Analysis	Analysis	
15	9	6	Reference	Analysis	Analysis	
16	10	6	Reference	Analysis	Analysis	
17	3	7	Reference	Analysis	Analysis	
18	4	7	Reference	Analysis	Analysis	
19	5	7	Reference	Analysis	Analysis	
20	6	7	Reference	Analysis	Analysis	
21	7	7	Reference	Analysis	Analysis	
22	8	7	Reference	Analysis	Analysis	
23	9	7	Reference	Analysis	Analysis	
24	10	7	Reference	Analysis	Analysis	

Plot table (continued)

#	Curve markers	Analyte 1 Solution	Analyte stability early_1 Relative (RU)
1		FAB 12C1	2.8
2		FAB 12C1	2.1
3		FAB 12C1	3.4
4		FAB 12C1	3.3
5		FAB 12C1	2.7
6		FAB 12C1	3.2
7		FAB 12C1	3.3
8		FAB 12C1	3.0
9		FAB 12C1	1.7
10		FAB 12C1	1.1
11		FAB 12C1	2.3
12		FAB 12C1	2.2
13		FAB 12C1	1.6
14		FAB 12C1	1.8
15		FAB 12C1	2.3
16		FAB 12C1	2.1
17		FAB 12C1	1.4
18		FAB 12C1	0.9
19		FAB 12C1	2.0
20		FAB 12C1	1.7
21		FAB 12C1	0.8
22		FAB 12C1	1.2
23		FAB 12C1	1.8
24		FAB 12C1	1.8

Evaluation - Kinetics

Thumbnails

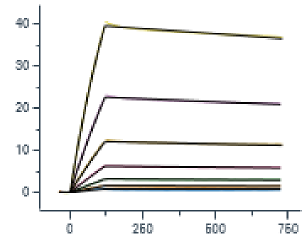

FAB 12C1; VB16T13 ECOLI; 1:1 binding

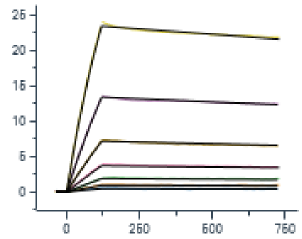

FAB 12C1; VB16T13 293F; 1:1 binding

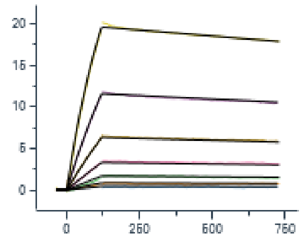

FAB 12C1; VB16T13 3T3; 1:1 binding

Charts

FAB 12C1; VB16T13 ECOLI; 1:1 binding

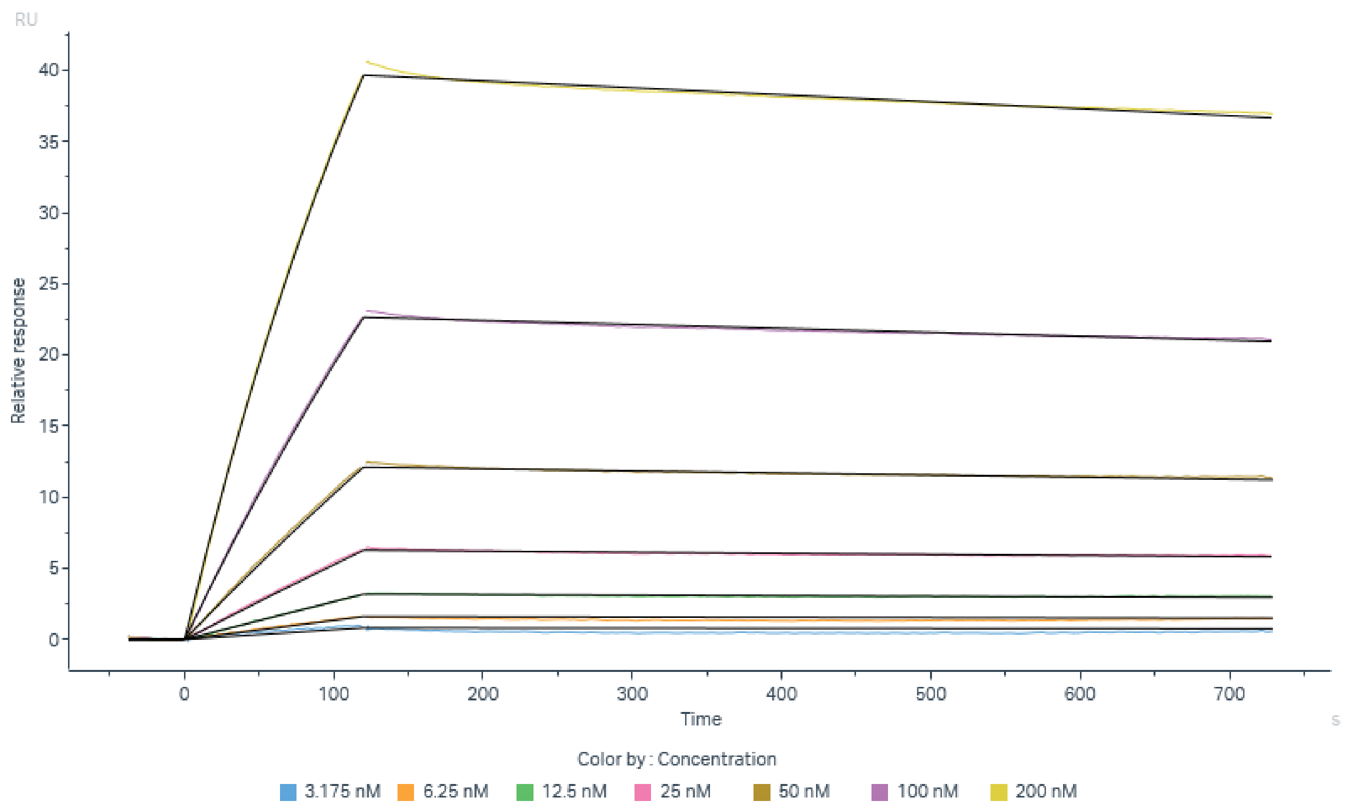

FAB 12C1; VB16T13 293F; 1:1 binding

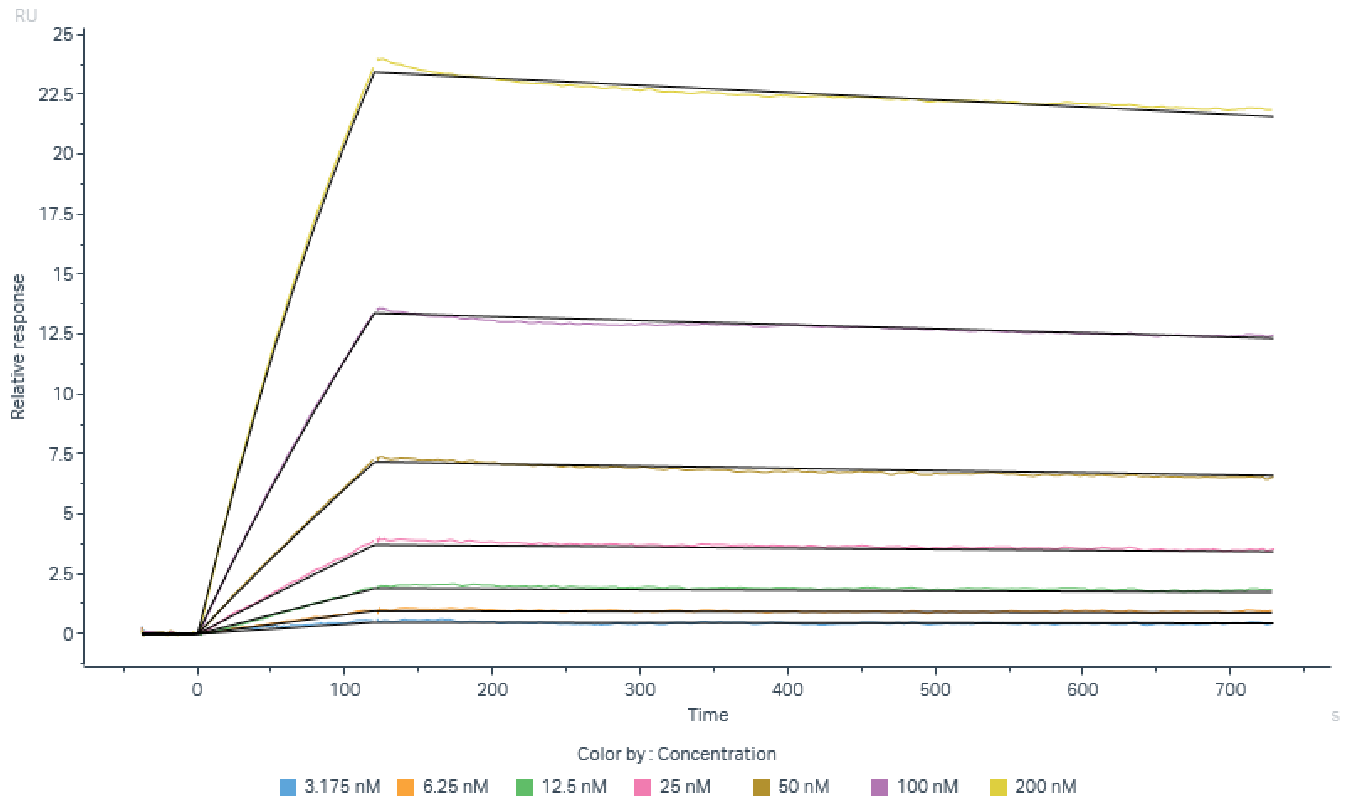

FAB 12C1; VB16T13 3T3; 1:1 binding

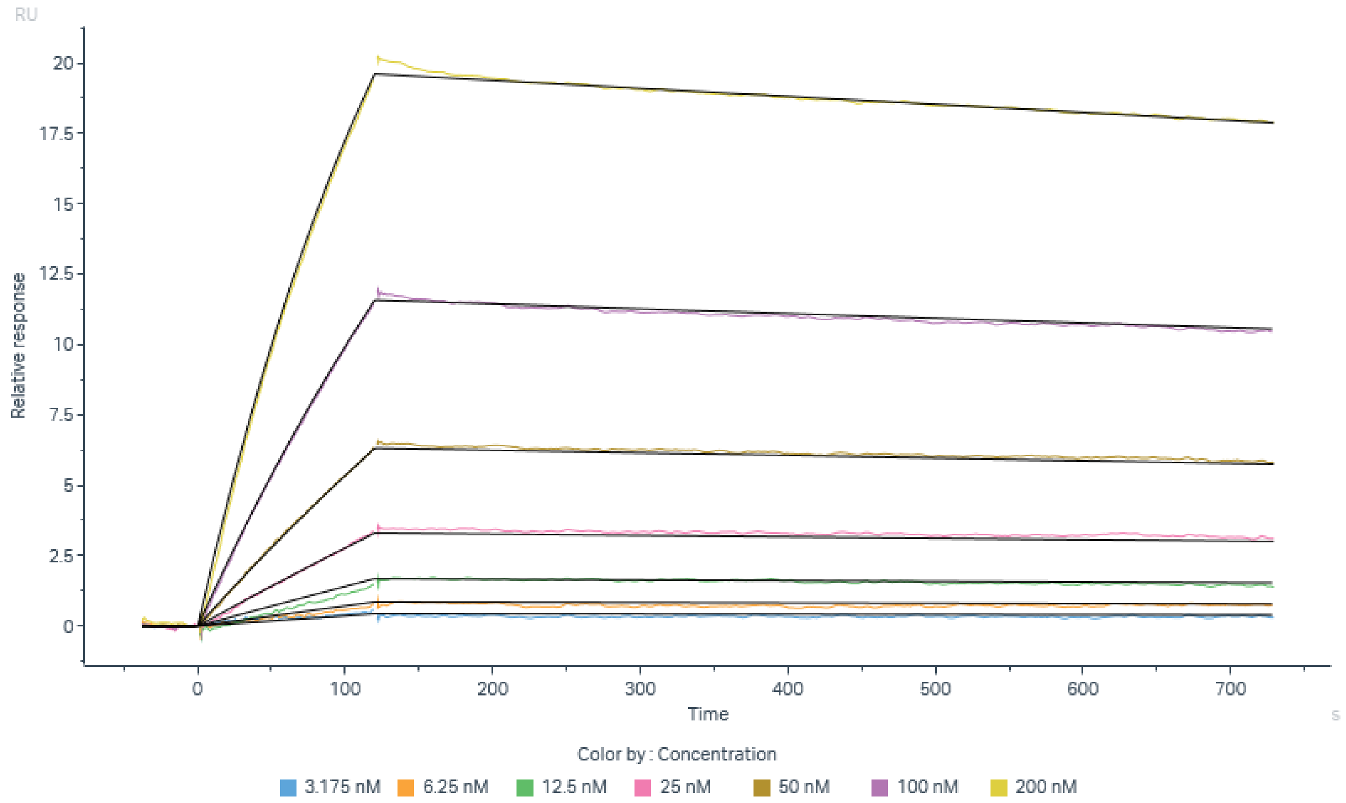

On-off rate chart

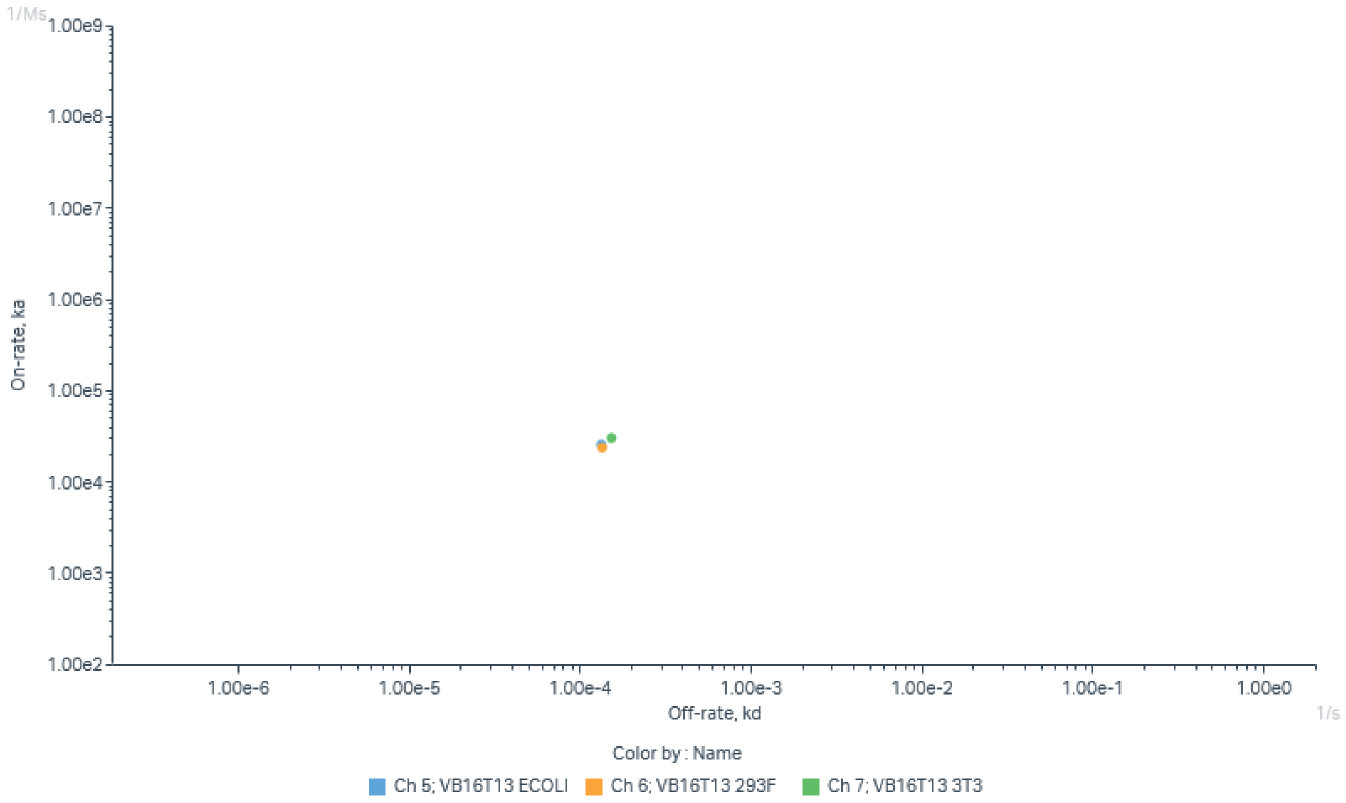

Result table

#	Group	General Kinetics model	Curve markers	Channel	Immobilized ligand	Injection variables Analyte 1 Solution
1	1	1:1 binding		5	VB16T13 ECOLI	FAB 12C1
2	2	1:1 binding		6	VB16T13 293F	FAB 12C1
3	3	1:1 binding		7	VB16T13 3T3	FAB 12C1

#	Quality Kinetics Chi² (RU²)	1:1 binding ka (1/Ms)	kd (1/s)	KD (M)	Rmax (RU)	tc
1	2.47e-02	2.59e+04	1.33e-04	5.13e-09	89.0	1.40e+07
2	1.02e-02	2.40e+04	1.34e-04	5.60e-09	54.0	4.58e+10
3	1.31e-02	3.05e+04	1.52e-04	4.98e-09	38.1	2.04e+10
