# Supplementary material for: A circular RNA vaccine induces durable and cross-protective immunity against Neisseria meningitidis serogroup B in mice
Source: PLoS Pathog. 2026 May 11;22(5):e1013741. doi: 10.1371/journal.ppat.1013741 (PMC13160355; doi:10.1371/journal.ppat.1013741)
Supplement: S1 Data — Images files. SPR PDF files. CE5200 PDF file. S1D Fig report. S1A Fig sequencing file. (ZIP) [file ppat.1013741.s016.zip › Raw data/SPR PDF files/FAB 1A12-5 VB16T13 ECOLI-6 VB16T13 293F-7 VB16T13 3t3-20260206 262026 51738 PM.pdf]

Biacore Insight Evaluation

FAB 1A12-5 VB16T13 ECOLI-6 VB16T13 293F-7 VB16T13 3t3-20260206 2/6/2026 5:17:38 PM

Evaluation

Name	FAB 1A12-5 VB16T13 ECOLI-6 VB16T13 293F-7 VB16T13 3t3-20260206 2/6/2026 5:17:38 PM
Path	Root\FAB 1A12-5 VB16T13 ECOLI-6 VB16T13 293F-7 VB16T13 3t3-20260206 2/6/2026 5:17:38 PM
Modified by	DESKTOP-22AQC8N\Dell (DESKTOP-22AQC8N\Dell)
Date modified	2/6/2026 10:22:46 PM
Created with	Biacore Insight Evaluation 4.0.8.19879
Server name	.\SQLEXPRESS
Database id	Biacore Insight Database

Run(s) in this evaluation

Run 1

Name	FAB 1A12-5 VB16T13 ECOLI-6 VB16T13 293F-7 VB16T13 3t3-20260206 2/6/2026 5:17:38 PM
Path	Root\ZW\QIQI ZHANG\FAB 1A12-5 VB16T13 ECOLI-6 VB16T13 293F-7 VB16T13 3t3-20260206 2/6/2026 5:17:38 PM
Created by	DESKTOP-22AQC8N\Dell (DESKTOP-22AQC8N\Dell)
Method	FAB 1A12-5 VB16T13 ECOLI-6 VB16T13 293F-7 VB16T13 3t3-20260206
Instrument type/ID	Biacore 8K+/2902034
Cycles	10
Data collection rate	10 Hz
Running buffer	Buffer
Start time	2/6/2026 5:17:43 PM
End time	2/6/2026 10:21:06 PM

Chip information

Chip id	10/20/2025 1:22:02 PM
Chip type	CM5
Lot number	10369187
First dock date	10/20/2025 1:23:00 PM

Run 1 - Immobilization results

Flow cell 1

	Channel 1	Channel 2	Channel 3	Channel 4
Sensorgram type	Not used in run	Not used in run	Not used in run	Not used in run
Ligand			SA	SA
MW (Da)				
Level (RU)			6142.6	6088.1
Run name			Immobilization 1/8/2026 10:43:22 AM	Immobilization 1/8/2026 10:43:22 AM
Date and time			1/8/2026 10:44:03 AM	1/8/2026 10:44:03 AM

	Channel 5	Channel 6	Channel 7	Channel 8
Sensorgram type	Reference	Reference	Reference	Not used in run
Ligand	Activation/Deactivation	Activation/Deactivation	Activation/Deactivation	
MW (Da)				
Level (RU)				
Run name	Immobilization 20260205 12:22:47 PM 2/5/2026 6:14:22 PM	Immobilization 20260205 12:22:47 PM 2/5/2026 6:14:22 PM	Immobilization 20260205 12:22:47 PM 2/5/2026 6:14:22 PM	
Date and time	2/5/2026 6:14:26 PM	2/5/2026 6:14:26 PM	2/5/2026 6:14:26 PM	

Run 1 - Immobilization results

Flow cell 2

	Channel 1	Channel 2	Channel 3	Channel 4
Sensorgram type	Not used in run	Not used in run	Not used in run	Not used in run
Ligand	Ligand 1	Ligand 2	SA	SA
MW (Da)				
Level (RU)	3483.5	5468.2	5936.3	6024.2
Run name	Immobilization 10/20/2025 1:40:59 PM	Immobilization 10/20/2025 1:40:59 PM	Immobilization 1/8/2026 10:43:22 AM	Immobilization 1/8/2026 10:43:22 AM
Date and time	10/20/2025 1:41:04 PM	10/20/2025 1:41:04 PM	1/8/2026 10:44:03 AM	1/8/2026 10:44:03 AM

	Channel 5	Channel 6	Channel 7	Channel 8
Sensorgram type	Active	Active	Active	Not used in run
Ligand	VB16T13 ECOLI	VB16T13 293F	VB16T13 3T3	
MW (Da)				
Level (RU)	288.4	256.3	323.2	
Run name	Immobilization 20260205 12:22:47 PM 2/5/2026 6:14:22 PM	Immobilization 20260205 12:22:47 PM 2/5/2026 6:14:22 PM	Immobilization 20260205 12:22:47 PM 2/5/2026 6:14:22 PM	
Date and time	2/5/2026 6:14:26 PM	2/5/2026 6:14:26 PM	2/5/2026 6:14:26 PM	

Run 1 - Method summary

2 Startup

Analyte 1

Buffer, Contact time 120 s, Diss time 60 s, Flow rate 30 µl/min, Both flow cells

Regeneration 1

1.5, Contact time 30 s, Flow rate 30 µl/min, Both flow cells

8 Analysis

Analyte 1

Variable solution, Contact time 120 s, Diss time 1800 s, Flow rate 30 µl/min, Variable conc, Both flow cells

Regeneration 1

1.5, Contact time 90 s, Flow rate 30 µl/min, Both flow cells

QC - Sensorgram

Thumbnails

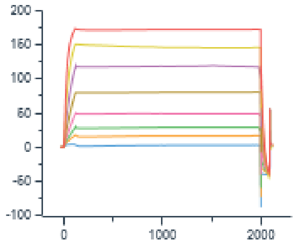

Channel 5

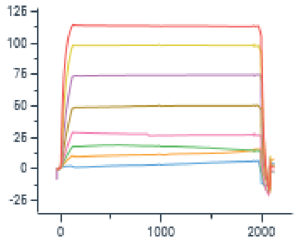

Channel 6

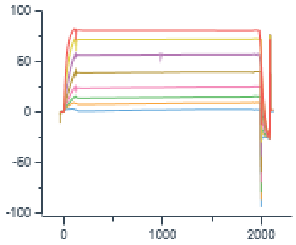

Channel 7

Charts

Channel 5

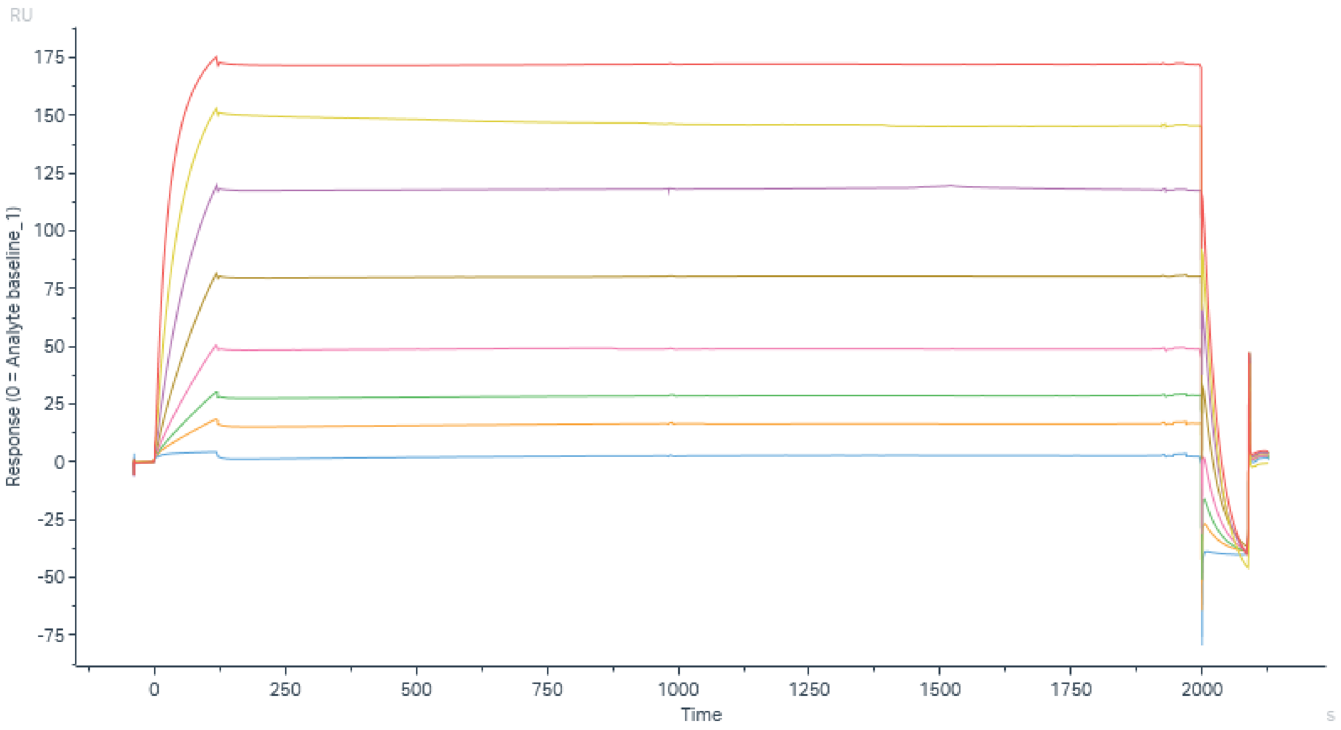

Channel 6

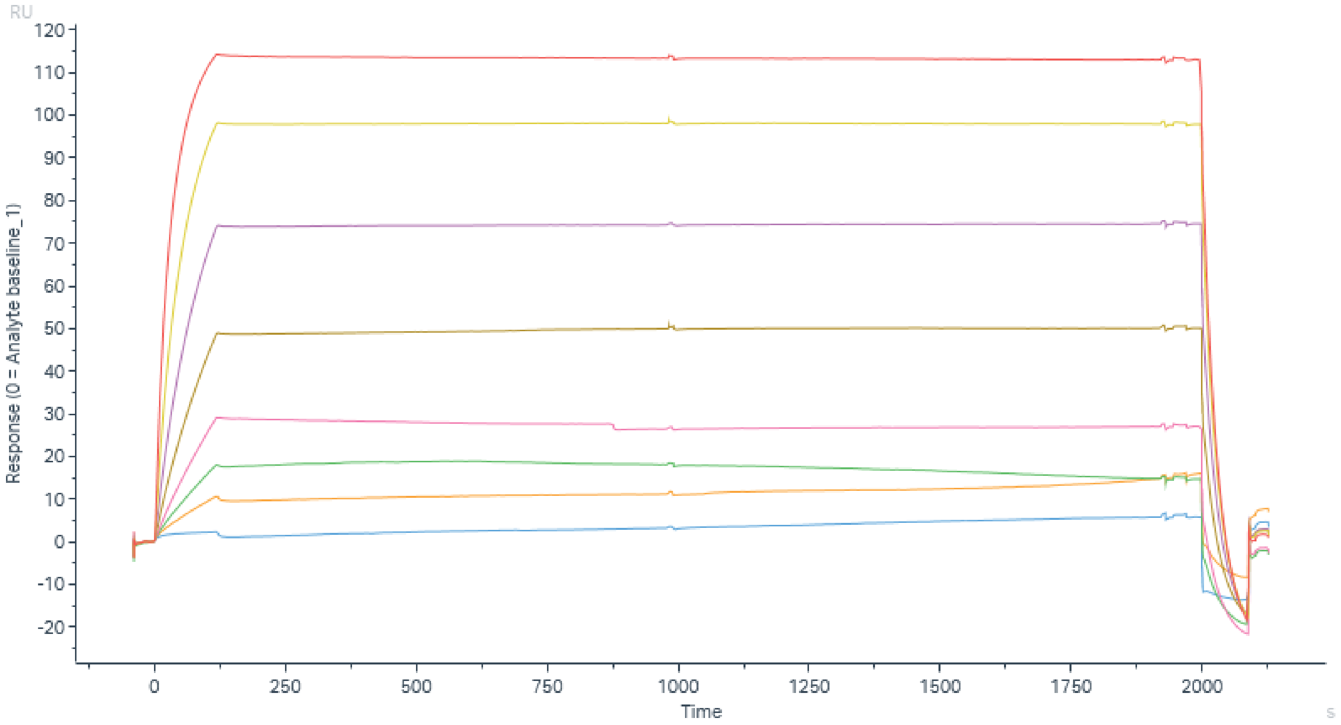

Channel 7

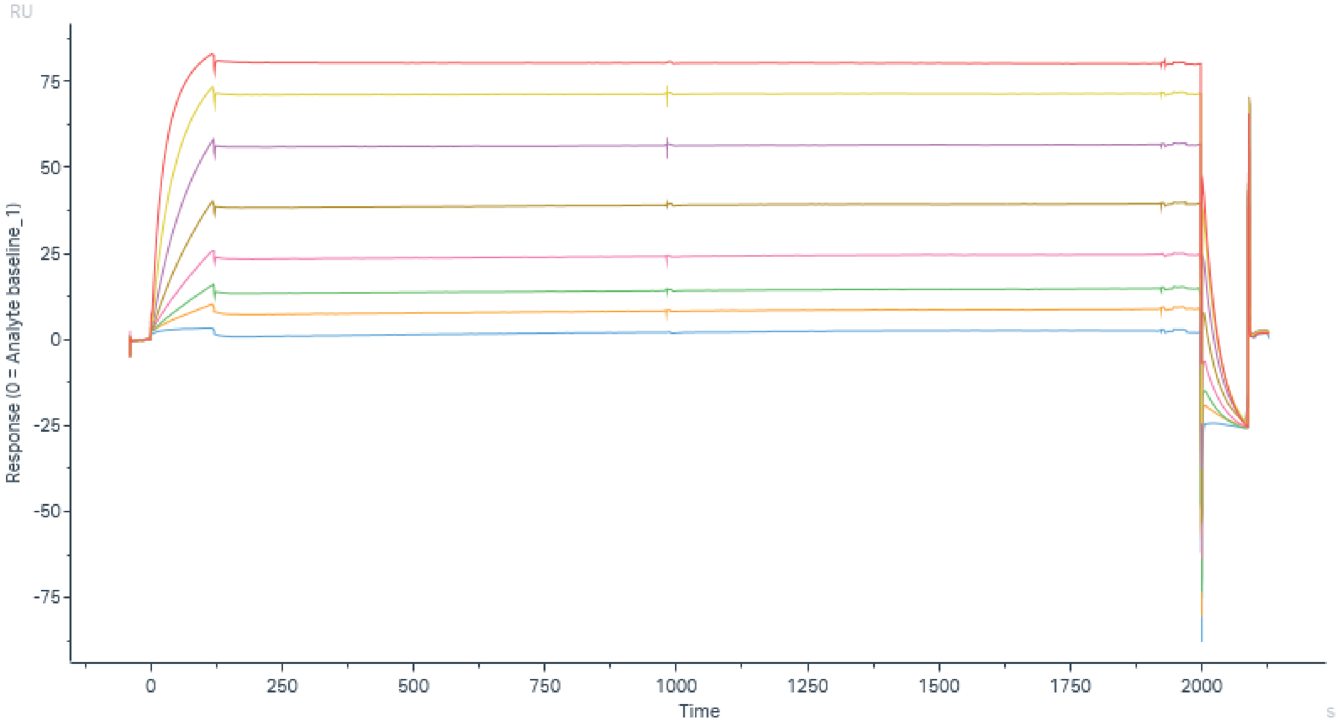
RU
75
50
25
0
-25
-50
-75
Response (0 = Analyte baseline_1)
0
250
500
750
1000
1250
1500
1750
2000
Time
s

Sensorgram table

#	Cycle	Channel	Sensorgram type	Analysis step purpose	Analysis step name	Excluded
1	3	5	Reference subtracted	Analysis	Analysis	
2	4	5	Reference subtracted	Analysis	Analysis	
3	5	5	Reference subtracted	Analysis	Analysis	
4	6	5	Reference subtracted	Analysis	Analysis	
5	7	5	Reference subtracted	Analysis	Analysis	
6	8	5	Reference subtracted	Analysis	Analysis	
7	9	5	Reference subtracted	Analysis	Analysis	
8	10	5	Reference subtracted	Analysis	Analysis	
9	3	6	Reference subtracted	Analysis	Analysis	
10	4	6	Reference subtracted	Analysis	Analysis	
11	5	6	Reference subtracted	Analysis	Analysis	
12	6	6	Reference subtracted	Analysis	Analysis	
13	7	6	Reference subtracted	Analysis	Analysis	
14	8	6	Reference subtracted	Analysis	Analysis	
15	9	6	Reference subtracted	Analysis	Analysis	
16	10	6	Reference subtracted	Analysis	Analysis	
17	3	7	Reference subtracted	Analysis	Analysis	
18	4	7	Reference subtracted	Analysis	Analysis	
19	5	7	Reference subtracted	Analysis	Analysis	
20	6	7	Reference subtracted	Analysis	Analysis	
21	7	7	Reference subtracted	Analysis	Analysis	
22	8	7	Reference subtracted	Analysis	Analysis	
23	9	7	Reference subtracted	Analysis	Analysis	
24	10	7	Reference subtracted	Analysis	Analysis	

Sensorgram table (continued)

#	Curve markers	Analyte 1 Solution	Analyte 1 Concentration (nM)	Analyte binding late_1 Relative (RU)
1		FAB 1A12	0	4.4
2		FAB 1A12	3.175	18.3
3		FAB 1A12	6.25	29.8
4		FAB 1A12	12.5	49.9
5		FAB 1A12	25	80.4
6		FAB 1A12	50	118.0
7		FAB 1A12	100	152.0
8		FAB 1A12	200	174.8
9		FAB 1A12	0	2.3
10		FAB 1A12	3.175	10.5
11		FAB 1A12	6.25	17.7
12		FAB 1A12	12.5	28.5
13		FAB 1A12	25	47.8
14		FAB 1A12	50	72.8
15		FAB 1A12	100	97.1
16		FAB 1A12	200	113.8
17		FAB 1A12	0	3.3
18		FAB 1A12	3.175	10.2
19		FAB 1A12	6.25	15.7
20		FAB 1A12	12.5	25.6
21		FAB 1A12	25	39.8
22		FAB 1A12	50	57.3
23		FAB 1A12	100	73.1
24		FAB 1A12	200	83.0

QC - Baseline

Thumbnails

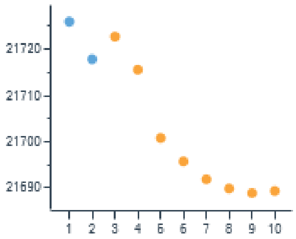

Channel 5

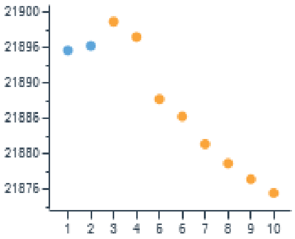

Channel 6

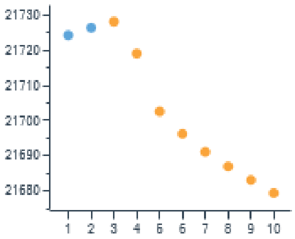

Channel 7

Charts

Channel 5

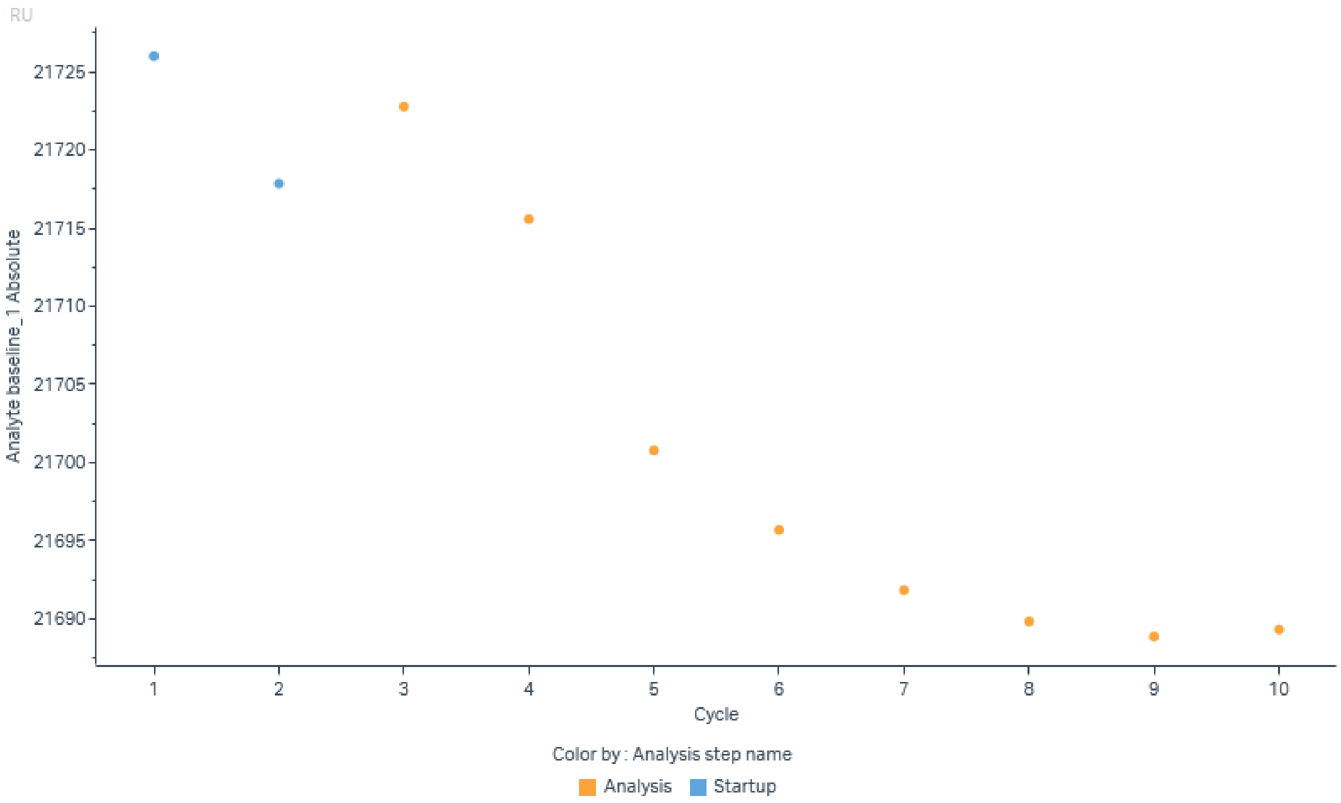

Channel 6

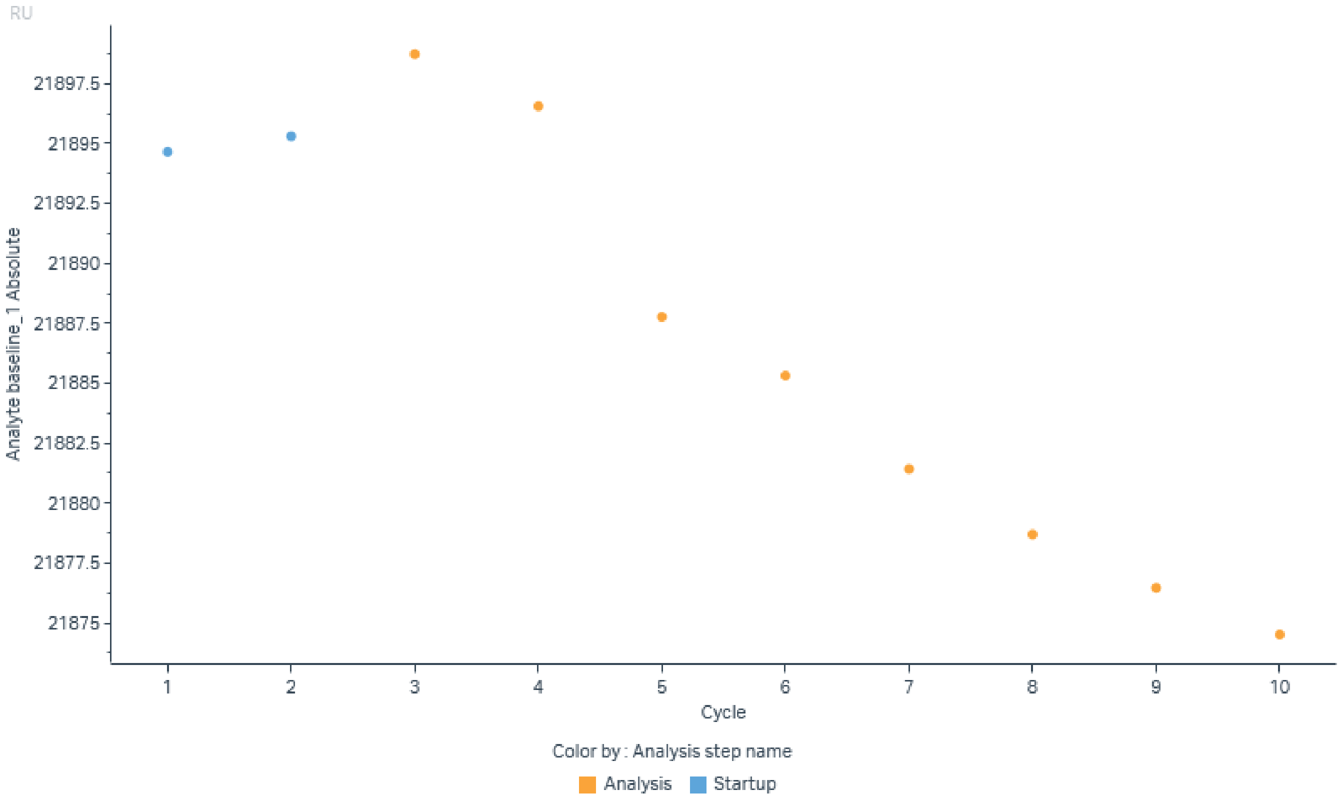

Channel 7

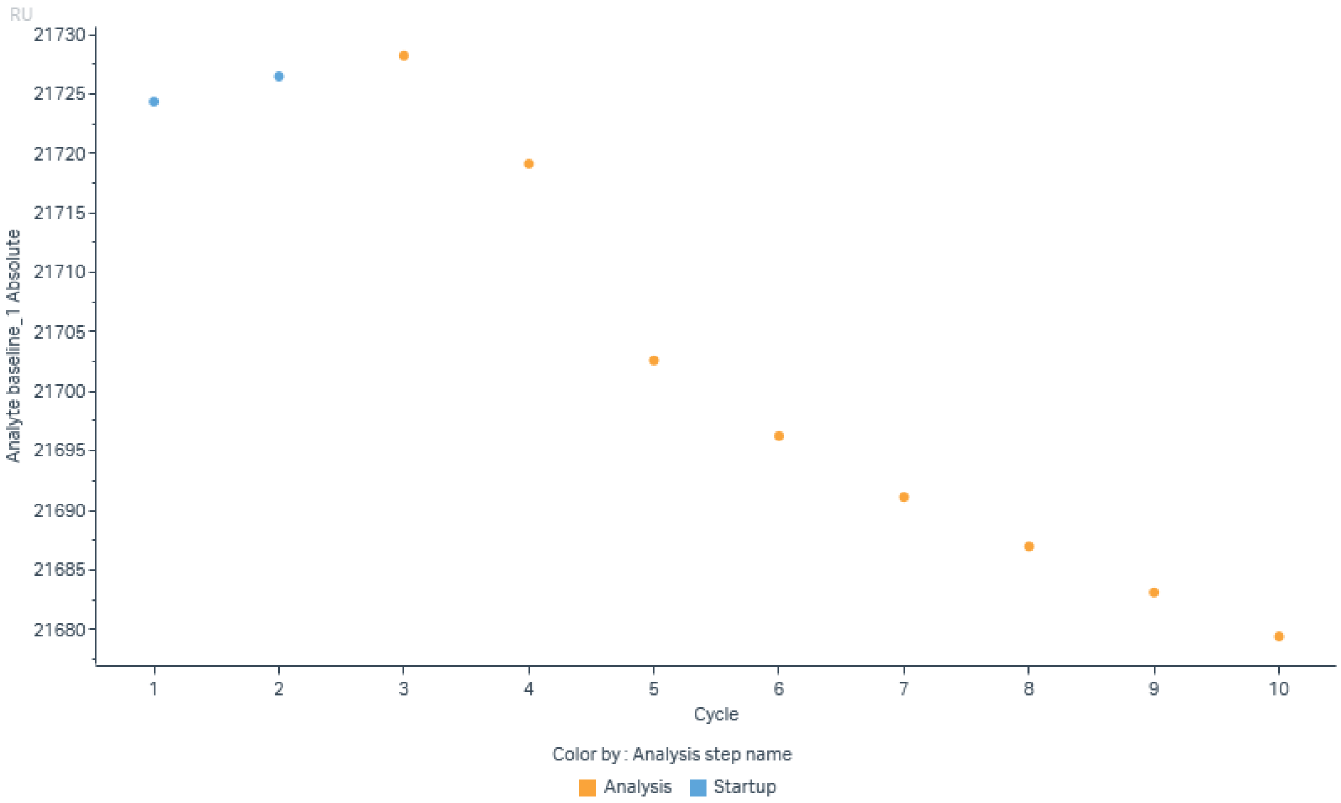
RU
21730
21725
21720
21715
21710
21705
21700
21695
21690
21685
21680
Analyte baseline_1 Absolute
1
2
3
4
5
6
7
8
9
10
Cycle
Color by : Analysis step name
Analysis
Startup

Plot table

#	Cycle	Channel	Sensorgram type	Analysis step purpose	Analysis step name	Excluded
1	1	5	Active	Startup	Startup	
2	2	5	Active	Startup	Startup	
3	3	5	Active	Analysis	Analysis	
4	4	5	Active	Analysis	Analysis	
5	5	5	Active	Analysis	Analysis	
6	6	5	Active	Analysis	Analysis	
7	7	5	Active	Analysis	Analysis	
8	8	5	Active	Analysis	Analysis	
9	9	5	Active	Analysis	Analysis	
10	10	5	Active	Analysis	Analysis	
11	1	6	Active	Startup	Startup	
12	2	6	Active	Startup	Startup	
13	3	6	Active	Analysis	Analysis	
14	4	6	Active	Analysis	Analysis	
15	5	6	Active	Analysis	Analysis	
16	6	6	Active	Analysis	Analysis	
17	7	6	Active	Analysis	Analysis	
18	8	6	Active	Analysis	Analysis	
19	9	6	Active	Analysis	Analysis	
20	10	6	Active	Analysis	Analysis	
21	1	7	Active	Startup	Startup	
22	2	7	Active	Startup	Startup	
23	3	7	Active	Analysis	Analysis	
24	4	7	Active	Analysis	Analysis	
25	5	7	Active	Analysis	Analysis	
26	6	7	Active	Analysis	Analysis	
27	7	7	Active	Analysis	Analysis	
28	8	7	Active	Analysis	Analysis	
29	9	7	Active	Analysis	Analysis	
30	10	7	Active	Analysis	Analysis	

Plot table (continued)

#	Curve markers	Analyte 1 Solution	Analyte baseline_1 Absolute (RU)
1		Buffer	21726.0
2		Buffer	21717.9
3		FAB 1A12	21722.8
4		FAB 1A12	21715.6
5		FAB 1A12	21700.8
6		FAB 1A12	21695.7
7		FAB 1A12	21691.8
8		FAB 1A12	21689.8
9		FAB 1A12	21688.9
10		FAB 1A12	21689.3
11		Buffer	21894.7
12		Buffer	21895.3
13		FAB 1A12	21898.7
14		FAB 1A12	21896.6
15		FAB 1A12	21887.8
16		FAB 1A12	21885.3
17		FAB 1A12	21881.4
18		FAB 1A12	21878.7
19		FAB 1A12	21876.5
20		FAB 1A12	21874.5
21		Buffer	21724.4
22		Buffer	21726.5
23		FAB 1A12	21728.2
24		FAB 1A12	21719.1
25		FAB 1A12	21702.6
26		FAB 1A12	21696.3
27		FAB 1A12	21691.1
28		FAB 1A12	21687.0
29		FAB 1A12	21683.1
30		FAB 1A12	21679.4

QC - Binding to reference

Thumbnails

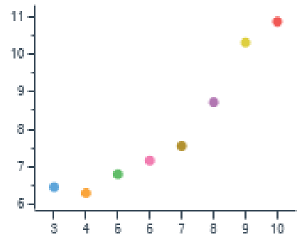

Channel 5

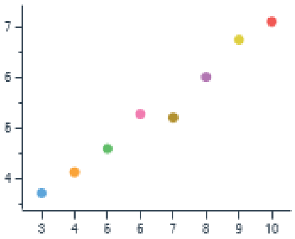

Channel 6

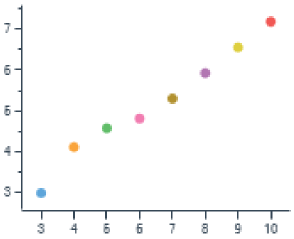

Channel 7

Charts

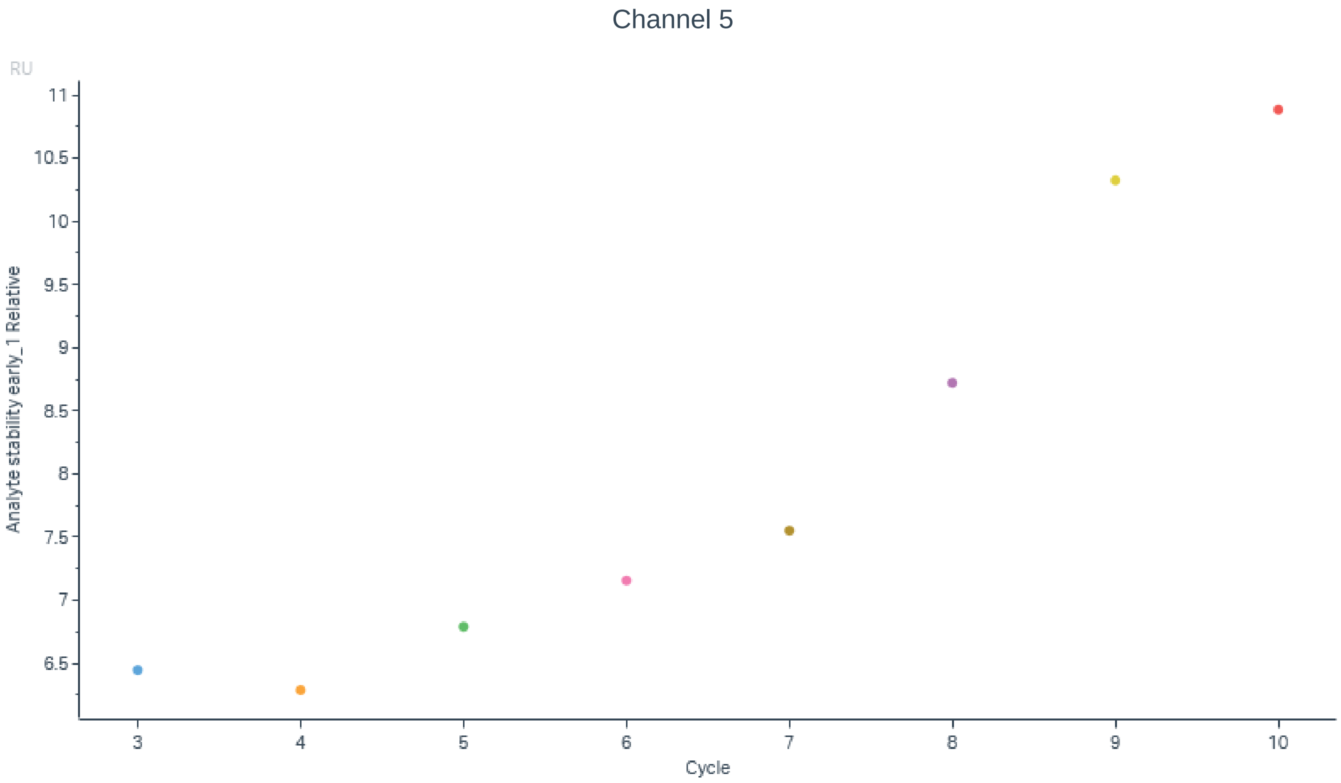
Channel 5
RU
Analyte stability early_1 Relative
11
10.5
10
9.5
9
8.5
8
7.5
7
6.5
3
4
5
6
7
8
9
10
Cycle

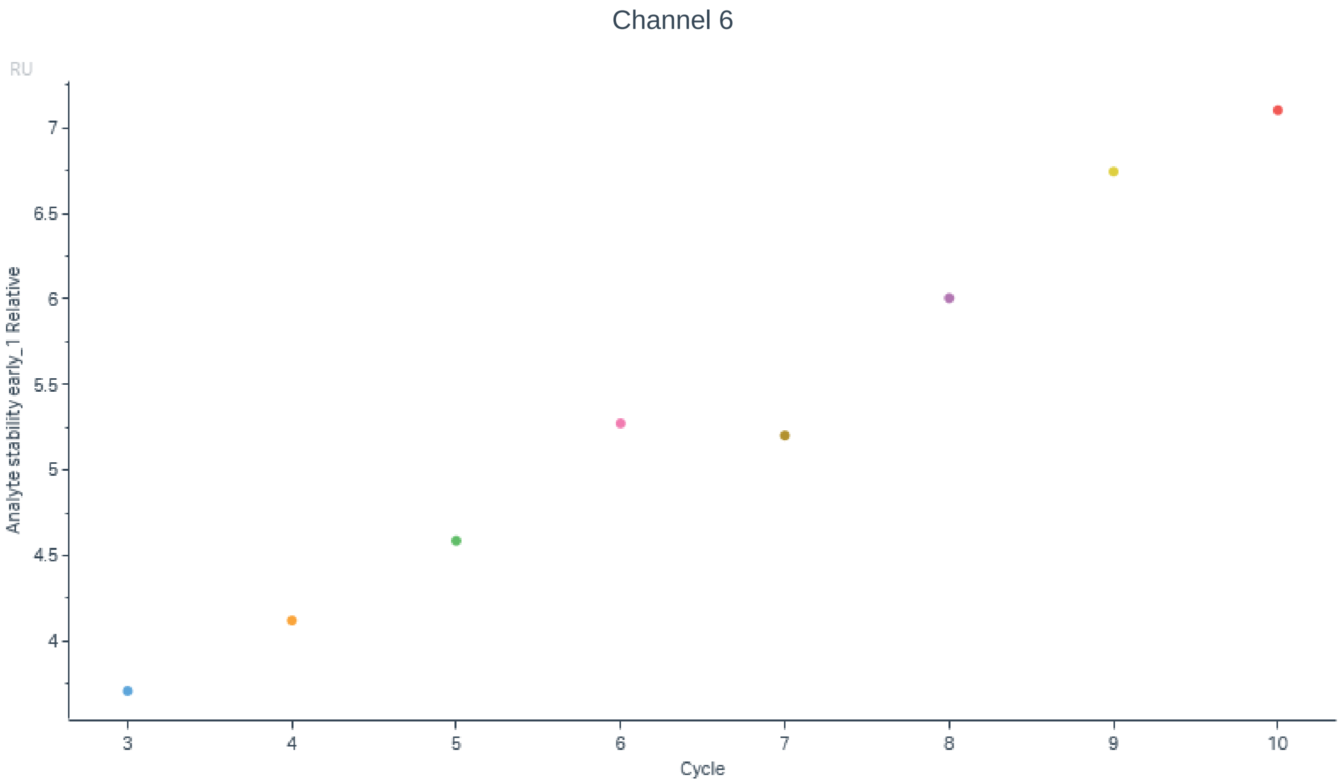
Channel 6
RU
Analyte stability early_1 Relative
7
6.5
6
5.5
5
4.5
4
3
4
5
6
7
8
9
10
Cycle

Channel 7

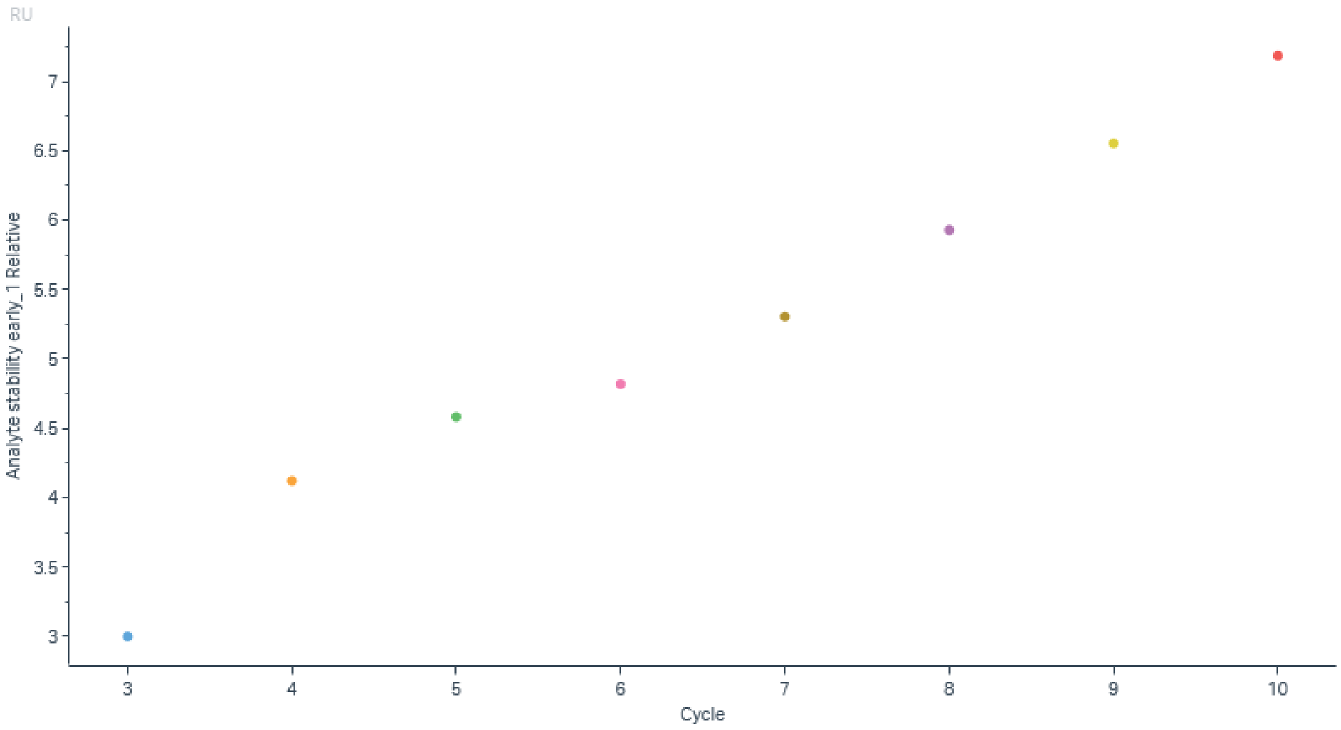
RU
Analyte stability early_1 Relative
7
6.5
6
5.5
5
4.5
4
3.5
3
3
4
5
6
7
8
9
10
Cycle

Plot table

#	Cycle	Channel	Sensorgram type	Analysis step purpose	Analysis step name	Excluded
1	3	5	Reference	Analysis	Analysis	
2	4	5	Reference	Analysis	Analysis	
3	5	5	Reference	Analysis	Analysis	
4	6	5	Reference	Analysis	Analysis	
5	7	5	Reference	Analysis	Analysis	
6	8	5	Reference	Analysis	Analysis	
7	9	5	Reference	Analysis	Analysis	
8	10	5	Reference	Analysis	Analysis	
9	3	6	Reference	Analysis	Analysis	
10	4	6	Reference	Analysis	Analysis	
11	5	6	Reference	Analysis	Analysis	
12	6	6	Reference	Analysis	Analysis	
13	7	6	Reference	Analysis	Analysis	
14	8	6	Reference	Analysis	Analysis	
15	9	6	Reference	Analysis	Analysis	
16	10	6	Reference	Analysis	Analysis	
17	3	7	Reference	Analysis	Analysis	
18	4	7	Reference	Analysis	Analysis	
19	5	7	Reference	Analysis	Analysis	
20	6	7	Reference	Analysis	Analysis	
21	7	7	Reference	Analysis	Analysis	
22	8	7	Reference	Analysis	Analysis	
23	9	7	Reference	Analysis	Analysis	
24	10	7	Reference	Analysis	Analysis	

Plot table (continued)

#	Curve markers	Analyte 1 Solution	Analyte stability early_1 Relative (RU)
1		FAB 1A12	6.4
2		FAB 1A12	6.3
3		FAB 1A12	6.8
4		FAB 1A12	7.2
5		FAB 1A12	7.6
6		FAB 1A12	8.7
7		FAB 1A12	10.3
8		FAB 1A12	10.9
9		FAB 1A12	3.7
10		FAB 1A12	4.1
11		FAB 1A12	4.6
12		FAB 1A12	5.3
13		FAB 1A12	5.2
14		FAB 1A12	6.0
15		FAB 1A12	6.7
16		FAB 1A12	7.1
17		FAB 1A12	3.0
18		FAB 1A12	4.1
19		FAB 1A12	4.6
20		FAB 1A12	4.8
21		FAB 1A12	5.3
22		FAB 1A12	5.9
23		FAB 1A12	6.6
24		FAB 1A12	7.2

Evaluation - Kinetics

Thumbnails

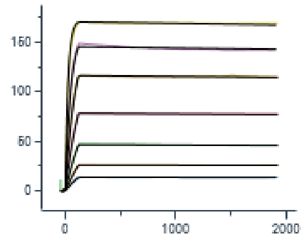

FAB 1A12; VB16T13 ECOLI; 1:1 binding

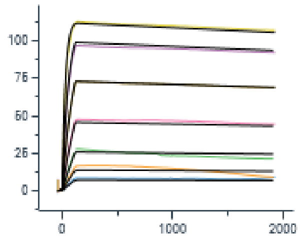

FAB 1A12; VB16T13 293F; 1:1 binding

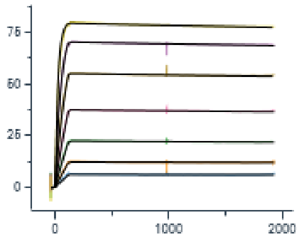

FAB 1A12; VB16T13 3T3; 1:1 binding

Charts

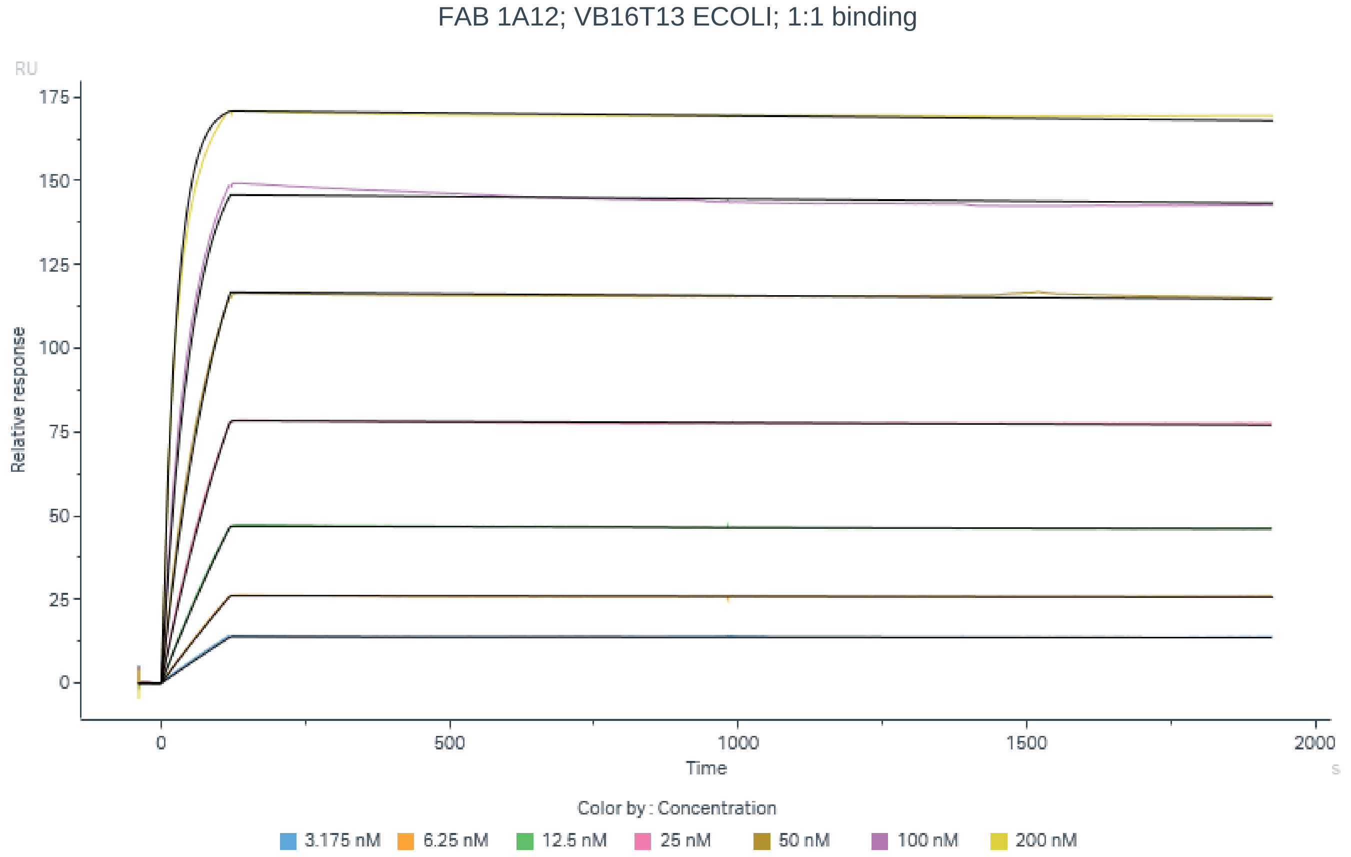
FAB 1A12; VB16T13 ECOLI; 1:1 binding
RU
Relative response
175
150
125
100
75
50
25
0
0
500
1000
1500
2000
Time
s
Color by : Concentration
3.175 nM
6.25 nM
12.5 nM
25 nM
50 nM
100 nM
200 nM

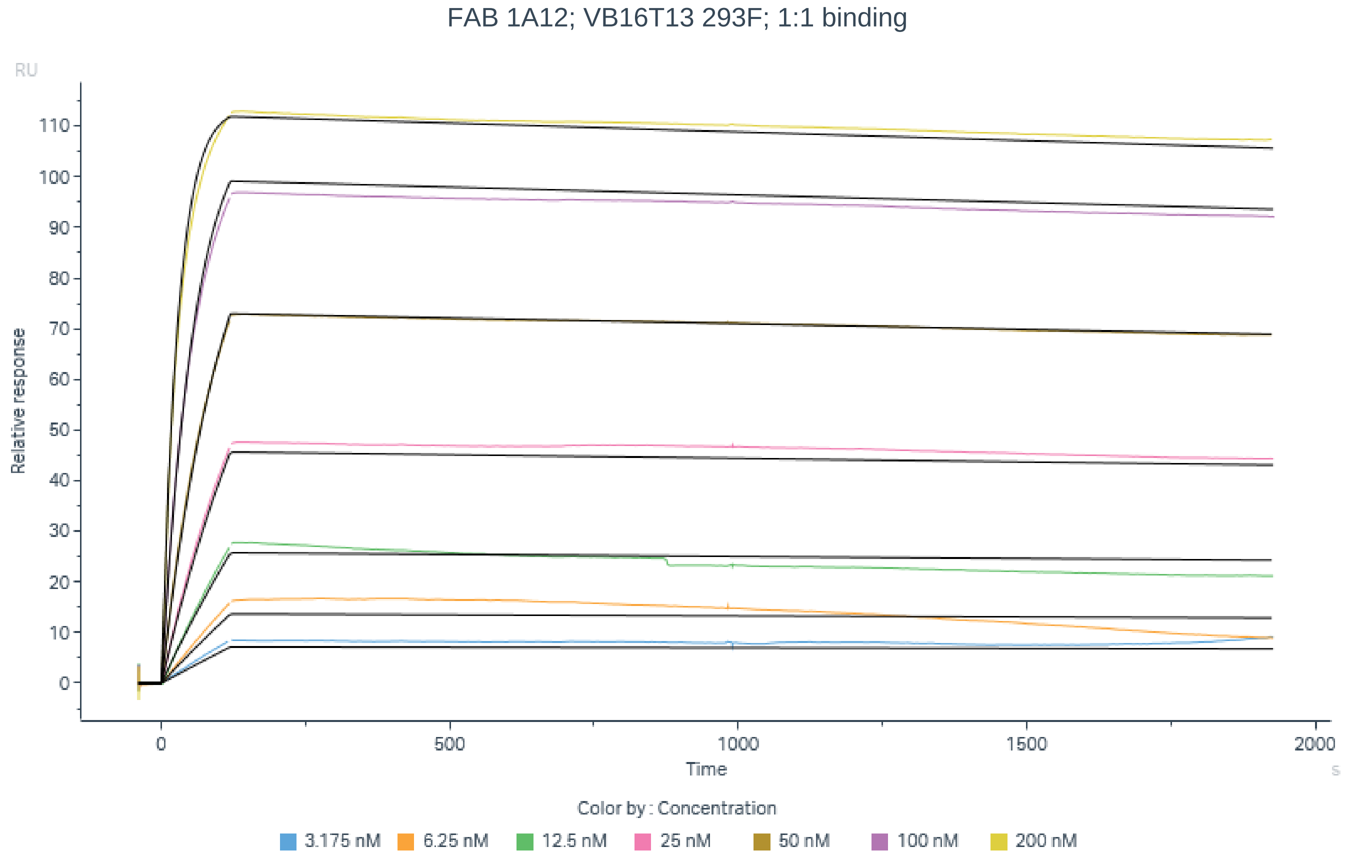
FAB 1A12; VB16T13 293F; 1:1 binding
RU
Relative response
110
100
90
80
70
60
50
40
30
20
10
0
0
500
1000
1500
2000
Time
s
Color by : Concentration
3.175 nM
6.25 nM
12.5 nM
25 nM
50 nM
100 nM
200 nM

FAB 1A12; VB16T13 3T3; 1:1 binding

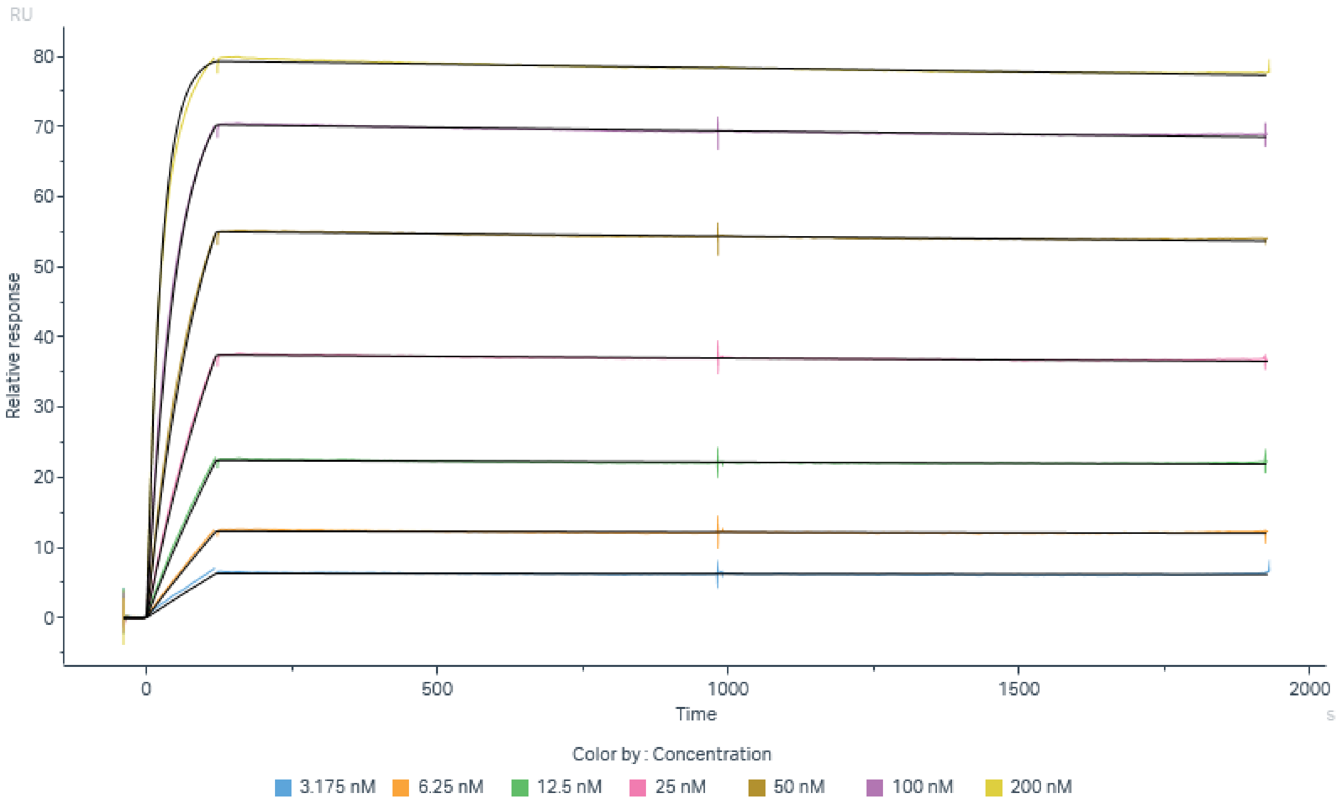

On-off rate chart

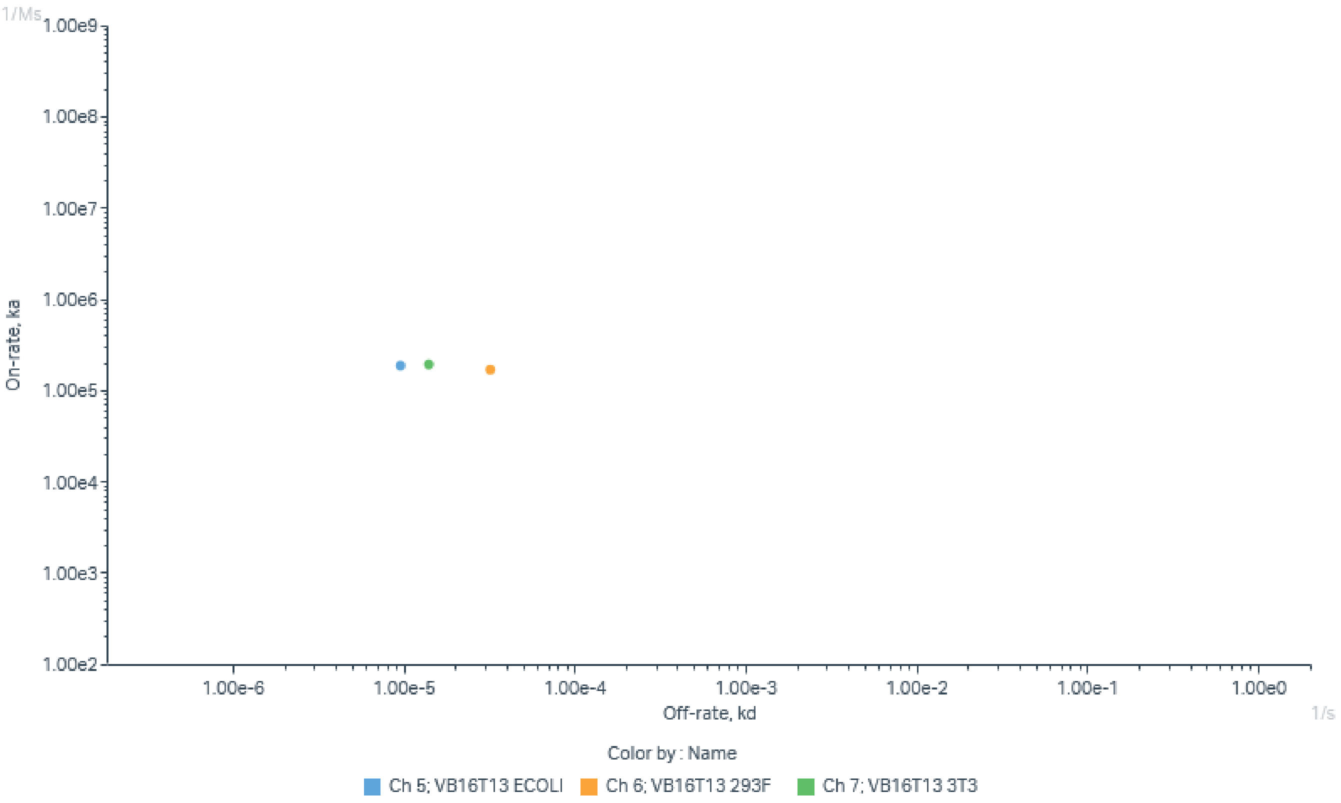

Result table

#	Group	General Kinetics model	Curve markers	Channel	Immobilized ligand	Injection variables Analyte 1 Solution
1	1	1:1 binding		5	VB16T13 ECOLI	FAB 1A12
2	2	1:1 binding		6	VB16T13 293F	FAB 1A12
3	3	1:1 binding		7	VB16T13 3T3	FAB 1A12

#	Quality Kinetics Chi² (RU²)	1:1 binding ka (1/Ms)	kd (1/s)	KD (M)	Rmax (RU)	tc
1	6.96e-01	1.90e+05	9.48e-06	5.00e-11		6.20e+12
2	2.66e+00	1.71e+05	3.18e-05	1.86e-10	1.14e+02	6.61e+12
3	6.57e-02	1.96e+05	1.39e-05	7.10e-11		1.04e+12
