# Supplementary material for: A circular RNA vaccine induces durable and cross-protective immunity against Neisseria meningitidis serogroup B in mice
Source: PLoS Pathog. 2026 May 11;22(5):e1013741. doi: 10.1371/journal.ppat.1013741 (PMC13160355; doi:10.1371/journal.ppat.1013741)
Supplement: S1 Data — Images files. SPR PDF files. CE5200 PDF file. S1D Fig report. S1A Fig sequencing file. (ZIP) [file ppat.1013741.s016.zip › Raw data/SPR PDF files/FAB 1E6-5 VB16T13 ECOLI-6 VB16T13 293F-7 VB16T13 3t3-20260205 252026 65844 PM.pdf]

Biacore Insight Evaluation

FAB 1E6-5 VB16T13 ECOLI-6 VB16T13 293F-7 VB16T13 3t3-20260205 2/5/2026 6:58:44 PM

Evaluation

Name	FAB 1E6-5 VB16T13 ECOLI-6 VB16T13 293F-7 VB16T13 3t3-20260205 2/5/2026 6:58:44 PM
Path	Root\FAB 1E6-5 VB16T13 ECOLI-6 VB16T13 293F-7 VB16T13 3t3-20260205 2/5/2026 6:58:44 PM
Modified by	DESKTOP-22AQC8N\Dell (DESKTOP-22AQC8N\Dell)
Date modified	2/6/2026 5:28:16 PM
Created with	Biacore Insight Evaluation 4.0.8.19879
Server name	.\SQLEXPRESS
Database id	Biacore Insight Database

Run(s) in this evaluation

Run 1

Name	FAB 1E6-5 VB16T13 ECOLI-6 VB16T13 293F-7 VB16T13 3t3-20260205 2/5/2026 6:58:44 PM
Path	Root\ZW\QIQI ZHANG\FAB 1E6-5 VB16T13 ECOLI-6 VB16T13 293F-7 VB16T13 3t3-20260205 2/5/2026 6:58:44 PM
Created by	DESKTOP-22AQC8N\Dell (DESKTOP-22AQC8N\Dell)
Method	FAB 1E6-5 VB16T13 ECOLI-6 VB16T13 293F-7 VB16T13 3t3-20260205
Instrument type/ID	Biacore 8K+/2902034
Cycles	10
Data collection rate	10 Hz
Running buffer	Buffer
Start time	2/6/2026 2:17:35 PM
End time	2/6/2026 4:40:31 PM

Chip information

Chip id	10/20/2025 1:22:02 PM
Chip type	CM5
Lot number	10369187
First dock date	10/20/2025 1:23:00 PM

Run 1 - Immobilization results

Flow cell 1

	Channel 1	Channel 2	Channel 3	Channel 4
Sensorgram type	Not used in run	Not used in run	Not used in run	Not used in run
Ligand			SA	SA
MW (Da)				
Level (RU)			6142.6	6088.1
Run name			Immobilization 1/8/2026 10:43:22 AM	Immobilization 1/8/2026 10:43:22 AM
Date and time			1/8/2026 10:44:03 AM	1/8/2026 10:44:03 AM

	Channel 5	Channel 6	Channel 7	Channel 8
Sensorgram type	Reference	Reference	Reference	Not used in run
Ligand	Activation/Deactivation	Activation/Deactivation	Activation/Deactivation	
MW (Da)				
Level (RU)				
Run name	Immobilization 20260205 12:22:47 PM 2/5/2026 6:14:22 PM	Immobilization 20260205 12:22:47 PM 2/5/2026 6:14:22 PM	Immobilization 20260205 12:22:47 PM 2/5/2026 6:14:22 PM	
Date and time	2/5/2026 6:14:26 PM	2/5/2026 6:14:26 PM	2/5/2026 6:14:26 PM	

Run 1 - Immobilization results

Flow cell 2

	Channel 1	Channel 2	Channel 3	Channel 4
Sensorgram type	Not used in run	Not used in run	Not used in run	Not used in run
Ligand	Ligand 1	Ligand 2	SA	SA
MW (Da)				
Level (RU)	3483.5	5468.2	5936.3	6024.2
Run name	Immobilization 10/20/2025 1:40:59 PM	Immobilization 10/20/2025 1:40:59 PM	Immobilization 1/8/2026 10:43:22 AM	Immobilization 1/8/2026 10:43:22 AM
Date and time	10/20/2025 1:41:04 PM	10/20/2025 1:41:04 PM	1/8/2026 10:44:03 AM	1/8/2026 10:44:03 AM

	Channel 5	Channel 6	Channel 7	Channel 8
Sensorgram type	Active	Active	Active	Not used in run
Ligand	VB16T13 ECOLI	VB16T13 293F	VB16T13 3T3	
MW (Da)				
Level (RU)	288.4	256.3	323.2	
Run name	Immobilization 20260205 12:22:47 PM 2/5/2026 6:14:22 PM	Immobilization 20260205 12:22:47 PM 2/5/2026 6:14:22 PM	Immobilization 20260205 12:22:47 PM 2/5/2026 6:14:22 PM	
Date and time	2/5/2026 6:14:26 PM	2/5/2026 6:14:26 PM	2/5/2026 6:14:26 PM	

Run 1 - Method summary

2 Startup

Analyte 1

Buffer, Contact time 120 s, Diss time 60 s, Flow rate 30 µl/min, Both flow cells

Regeneration 1

1.5, Contact time 30 s, Flow rate 30 µl/min, Both flow cells

8 Analysis

Analyte 1

Variable solution, Contact time 120 s, Diss time 600 s, Flow rate 30 µl/min, Variable conc, Both flow cells

Regeneration 1

1.5, Contact time 90 s, Flow rate 30 µl/min, Both flow cells

QC - Sensorgram

Thumbnails

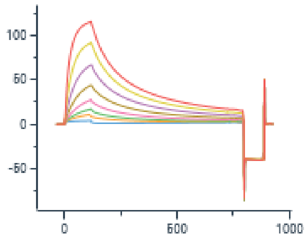

Channel 5

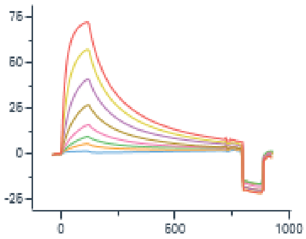

Channel 6

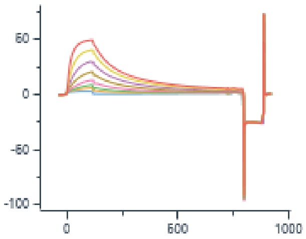

Channel 7

Charts

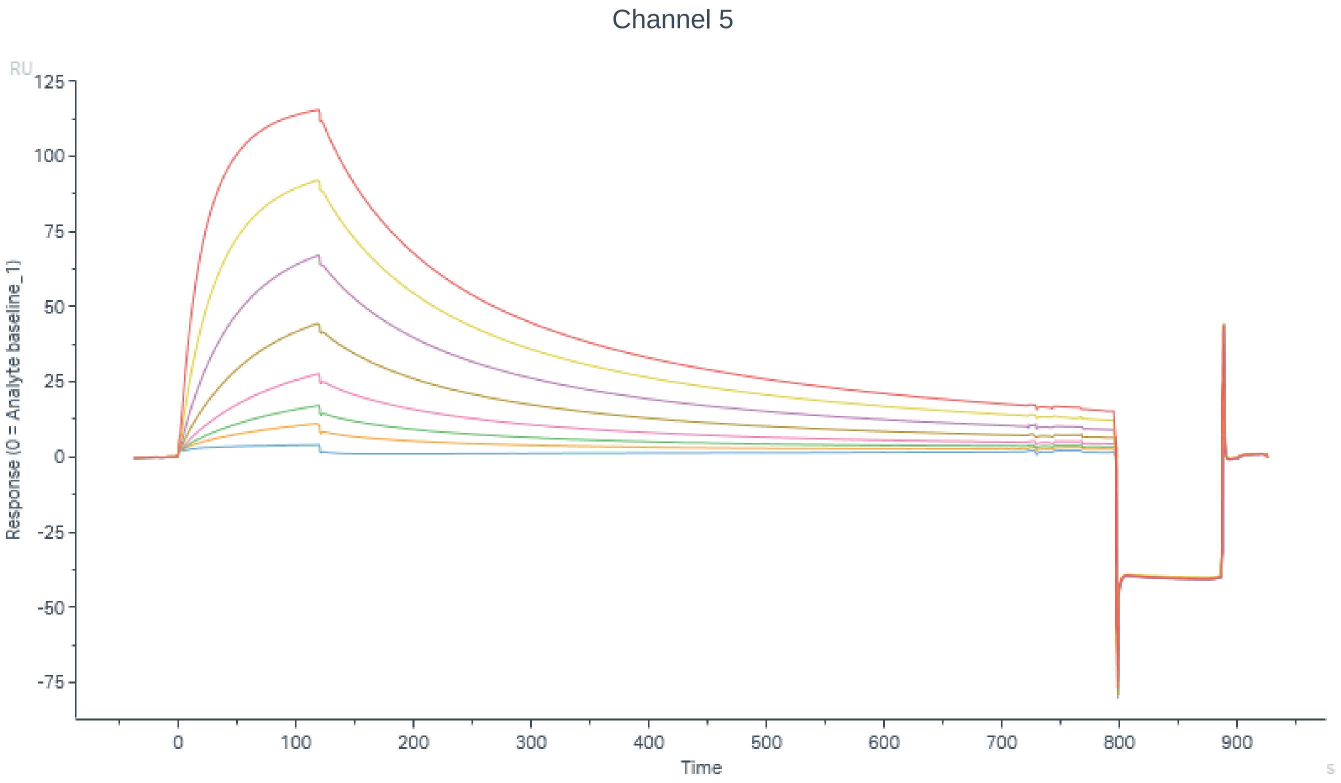
Channel 5
RU
125
100
75
50
25
0
-25
-50
-75
Response (0 = Analyte baseline_1)
0
100
200
300
400
500
600
700
800
900
Time
s

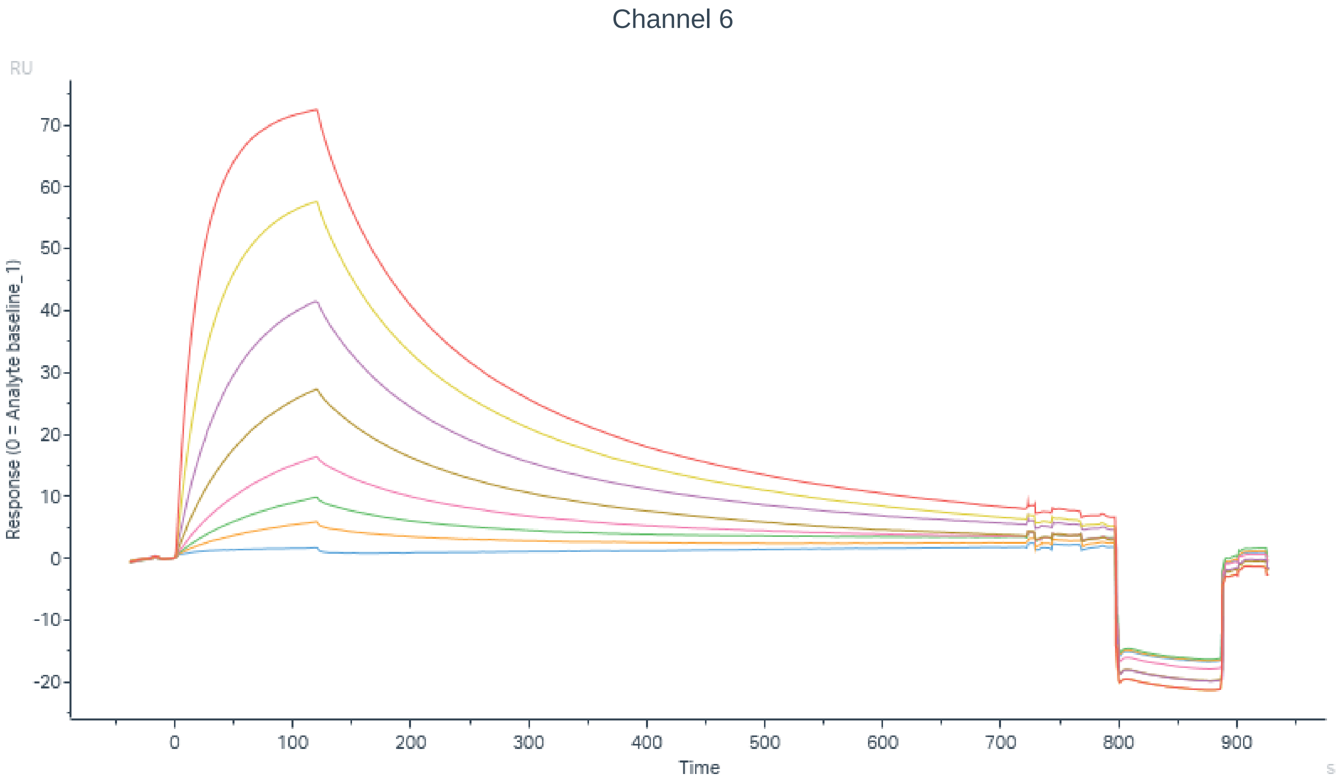
Channel 6
RU
70
60
50
40
30
20
10
0
-10
-20
Response (0 = Analyte baseline_1)
0
100
200
300
400
500
600
700
800
900
Time
s

Channel 7

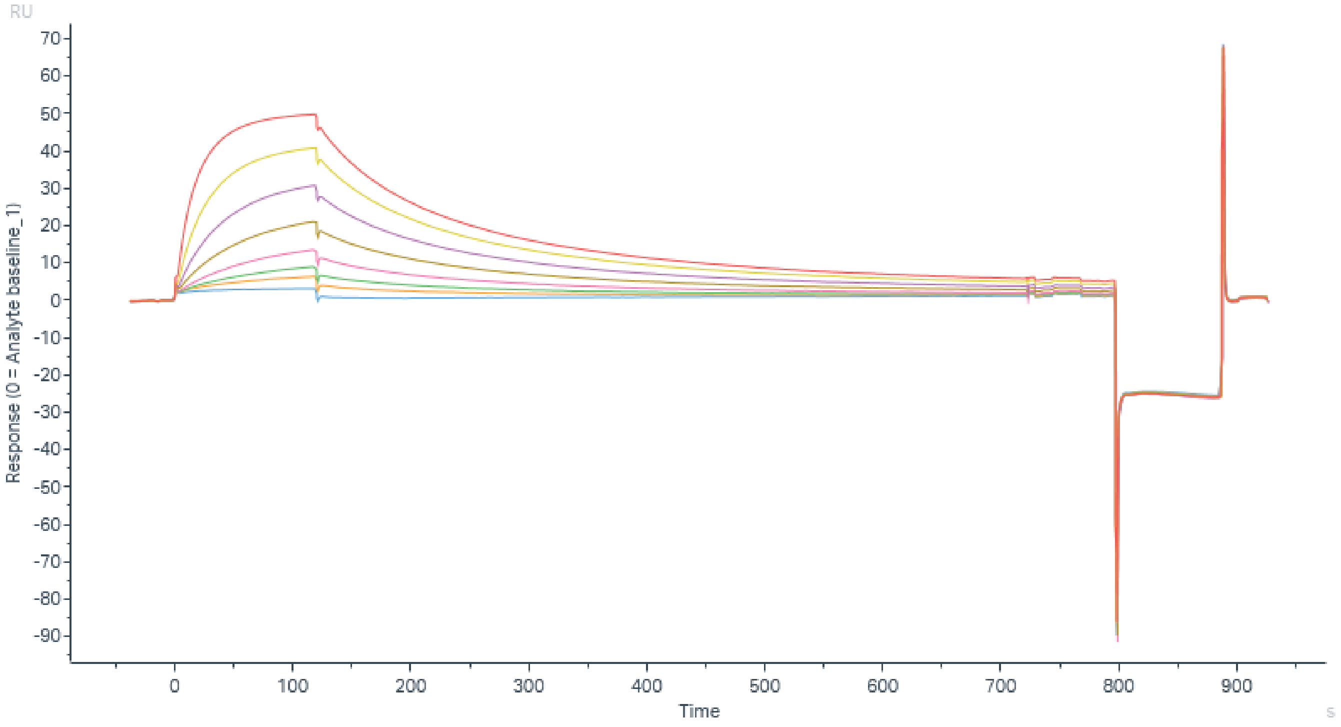
RU
70
60
50
40
30
20
10
0
-10
-20
-30
-40
-50
-60
-70
-80
-90
Response (0 = Analyte baseline_1)
0
100
200
300
400
500
600
700
800
900
Time
s

Sensorgram table

#	Cycle	Channel	Sensorgram type	Analysis step purpose	Analysis step name	Excluded
1	3	5	Reference subtracted	Analysis	Analysis	
2	4	5	Reference subtracted	Analysis	Analysis	
3	5	5	Reference subtracted	Analysis	Analysis	
4	6	5	Reference subtracted	Analysis	Analysis	
5	7	5	Reference subtracted	Analysis	Analysis	
6	8	5	Reference subtracted	Analysis	Analysis	
7	9	5	Reference subtracted	Analysis	Analysis	
8	10	5	Reference subtracted	Analysis	Analysis	
9	3	6	Reference subtracted	Analysis	Analysis	
10	4	6	Reference subtracted	Analysis	Analysis	
11	5	6	Reference subtracted	Analysis	Analysis	
12	6	6	Reference subtracted	Analysis	Analysis	
13	7	6	Reference subtracted	Analysis	Analysis	
14	8	6	Reference subtracted	Analysis	Analysis	
15	9	6	Reference subtracted	Analysis	Analysis	
16	10	6	Reference subtracted	Analysis	Analysis	
17	3	7	Reference subtracted	Analysis	Analysis	
18	4	7	Reference subtracted	Analysis	Analysis	
19	5	7	Reference subtracted	Analysis	Analysis	
20	6	7	Reference subtracted	Analysis	Analysis	
21	7	7	Reference subtracted	Analysis	Analysis	
22	8	7	Reference subtracted	Analysis	Analysis	
23	9	7	Reference subtracted	Analysis	Analysis	
24	10	7	Reference subtracted	Analysis	Analysis	

Sensorgram table (continued)

#	Curve markers	Analyte 1 Solution	Analyte 1 Concentration (nM)	Analyte binding late_1 Relative (RU)
1		FAB 1E6	0	4.0
2		FAB 1E6	3.175	11.0
3		FAB 1E6	6.25	16.9
4		FAB 1E6	12.5	27.2
5		FAB 1E6	25	44.0
6		FAB 1E6	50	66.5
7		FAB 1E6	100	91.6
8		FAB 1E6	200	115.2
9		FAB 1E6	0	1.7
10		FAB 1E6	3.175	5.8
11		FAB 1E6	6.25	9.7
12		FAB 1E6	12.5	16.1
13		FAB 1E6	25	26.9
14		FAB 1E6	50	41.2
15		FAB 1E6	100	57.3
16		FAB 1E6	200	72.3
17		FAB 1E6	0	3.1
18		FAB 1E6	3.175	6.3
19		FAB 1E6	6.25	8.9
20		FAB 1E6	12.5	13.4
21		FAB 1E6	25	20.9
22		FAB 1E6	50	30.5
23		FAB 1E6	100	40.7
24		FAB 1E6	200	49.7

QC - Baseline

Thumbnails

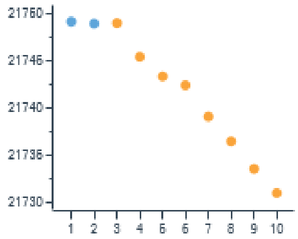

Channel 5

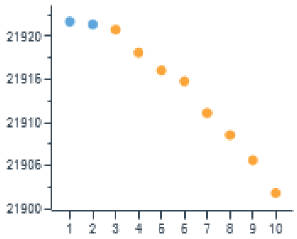

Channel 6

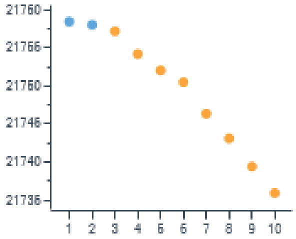

Channel 7

Charts

Channel 5

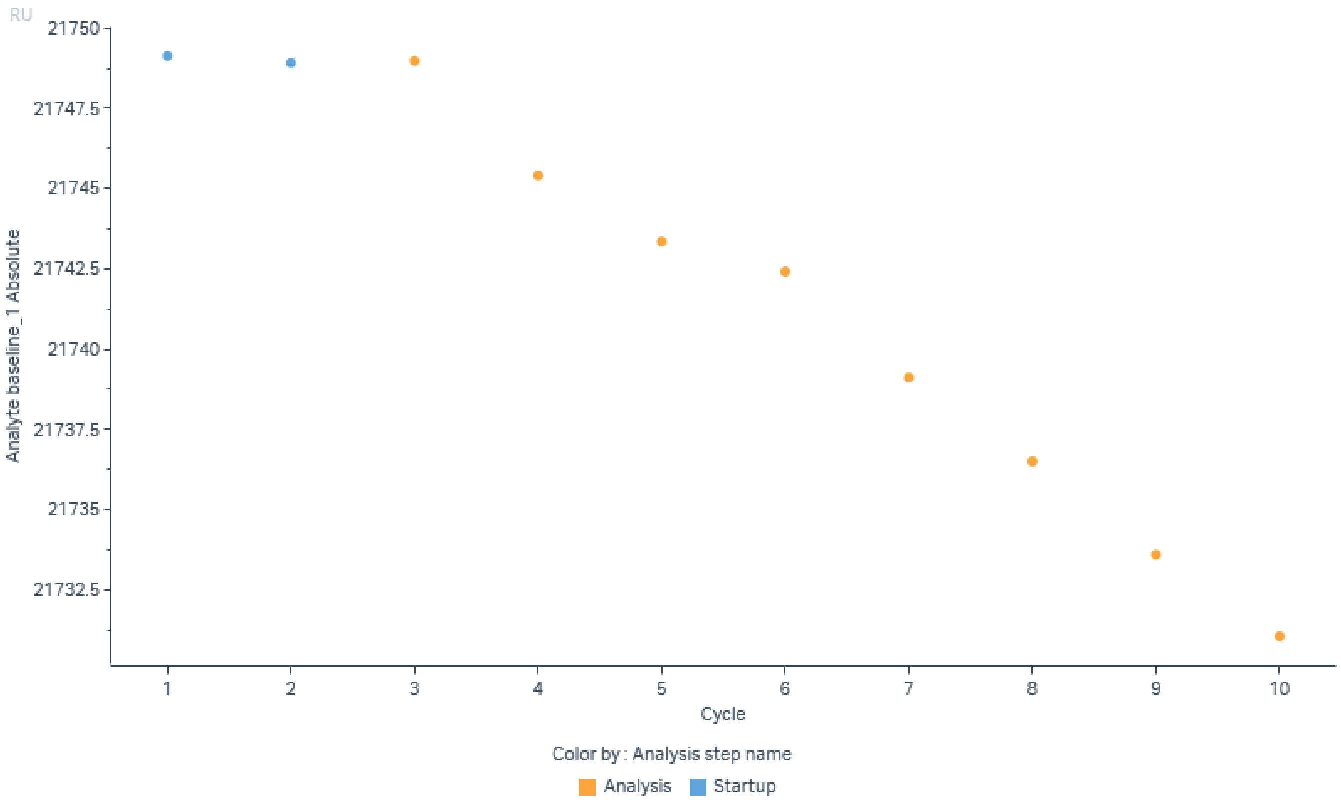

Channel 6

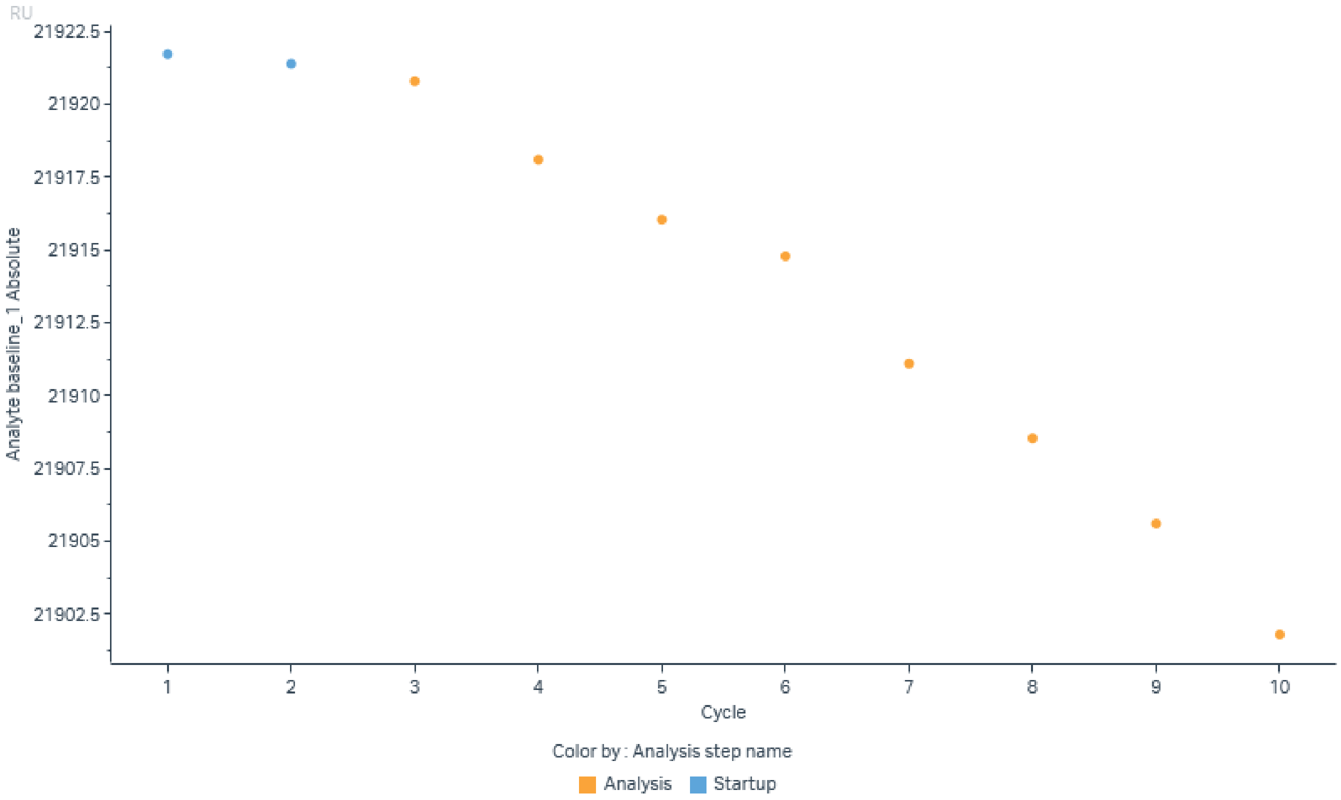

Channel 7

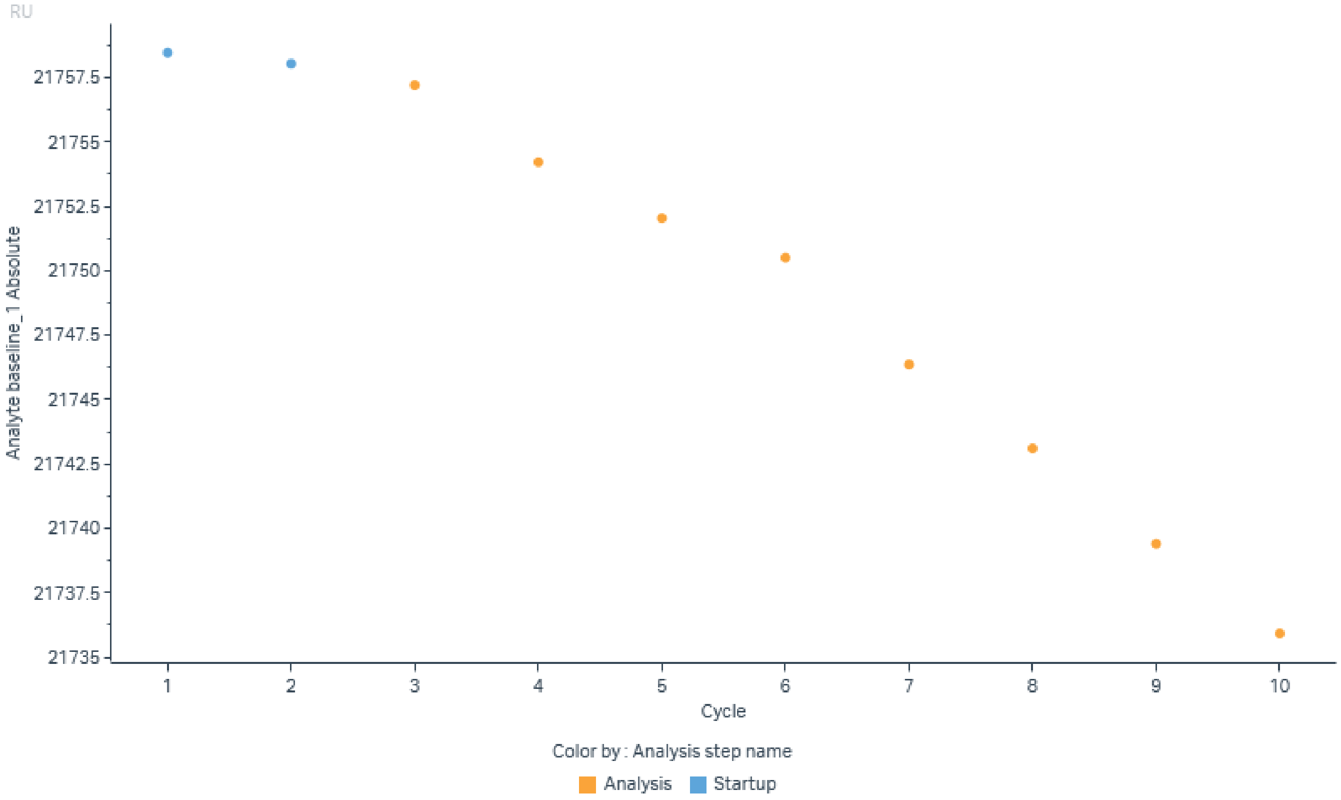
RU
Analyte baseline_1 Absolute
21757.5
21755
21752.5
21750
21747.5
21745
21742.5
21740
21737.5
21735
1
2
3
4
5
6
7
8
9
10
Cycle
Color by : Analysis step name
Analysis
Startup

Plot table

#	Cycle	Channel	Sensorgram type	Analysis step purpose	Analysis step name	Excluded
1	1	5	Active	Startup	Startup	
2	2	5	Active	Startup	Startup	
3	3	5	Active	Analysis	Analysis	
4	4	5	Active	Analysis	Analysis	
5	5	5	Active	Analysis	Analysis	
6	6	5	Active	Analysis	Analysis	
7	7	5	Active	Analysis	Analysis	
8	8	5	Active	Analysis	Analysis	
9	9	5	Active	Analysis	Analysis	
10	10	5	Active	Analysis	Analysis	
11	1	6	Active	Startup	Startup	
12	2	6	Active	Startup	Startup	
13	3	6	Active	Analysis	Analysis	
14	4	6	Active	Analysis	Analysis	
15	5	6	Active	Analysis	Analysis	
16	6	6	Active	Analysis	Analysis	
17	7	6	Active	Analysis	Analysis	
18	8	6	Active	Analysis	Analysis	
19	9	6	Active	Analysis	Analysis	
20	10	6	Active	Analysis	Analysis	
21	1	7	Active	Startup	Startup	
22	2	7	Active	Startup	Startup	
23	3	7	Active	Analysis	Analysis	
24	4	7	Active	Analysis	Analysis	
25	5	7	Active	Analysis	Analysis	
26	6	7	Active	Analysis	Analysis	
27	7	7	Active	Analysis	Analysis	
28	8	7	Active	Analysis	Analysis	
29	9	7	Active	Analysis	Analysis	
30	10	7	Active	Analysis	Analysis	

Plot table (continued)

#	Curve markers	Analyte 1 Solution	Analyte baseline_1 Absolute (RU)
1		Buffer	21749.1
2		Buffer	21748.9
3		FAB 1E6	21749.0
4		FAB 1E6	21745.4
5		FAB 1E6	21743.3
6		FAB 1E6	21742.4
7		FAB 1E6	21739.1
8		FAB 1E6	21736.5
9		FAB 1E6	21733.6
10		FAB 1E6	21731.0
11		Buffer	21921.7
12		Buffer	21921.4
13		FAB 1E6	21920.8
14		FAB 1E6	21918.1
15		FAB 1E6	21916.0
16		FAB 1E6	21914.8
17		FAB 1E6	21911.1
18		FAB 1E6	21908.5
19		FAB 1E6	21905.6
20		FAB 1E6	21901.8
21		Buffer	21758.5
22		Buffer	21758.0
23		FAB 1E6	21757.2
24		FAB 1E6	21754.2
25		FAB 1E6	21752.0
26		FAB 1E6	21750.5
27		FAB 1E6	21746.4
28		FAB 1E6	21743.1
29		FAB 1E6	21739.4
30		FAB 1E6	21735.9

QC - Binding to reference

Thumbnails

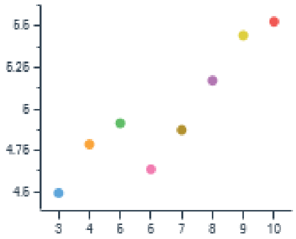

Channel 5

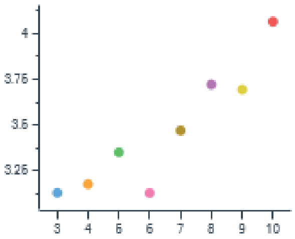

Channel 6

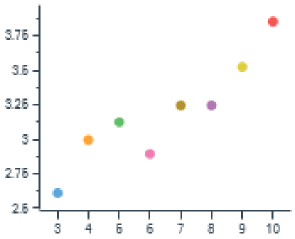

Channel 7

Charts

Channel 5

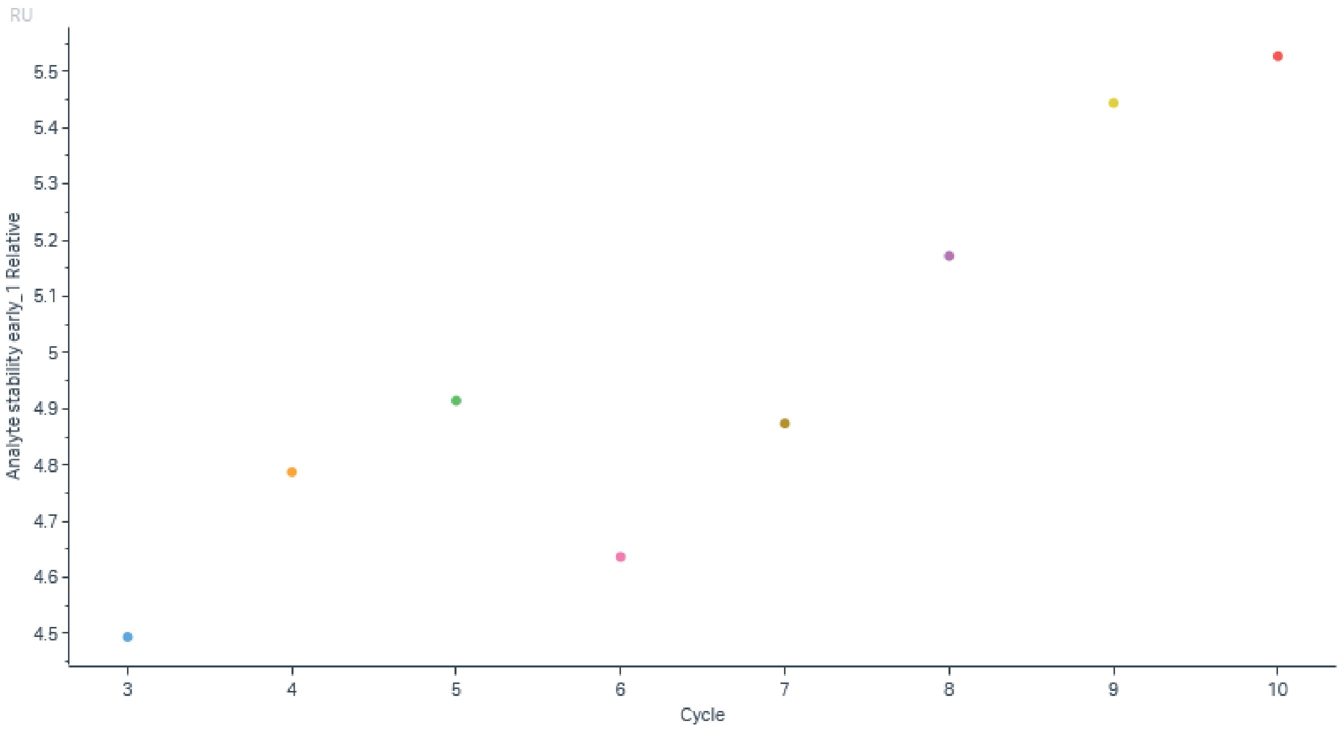

Channel 6

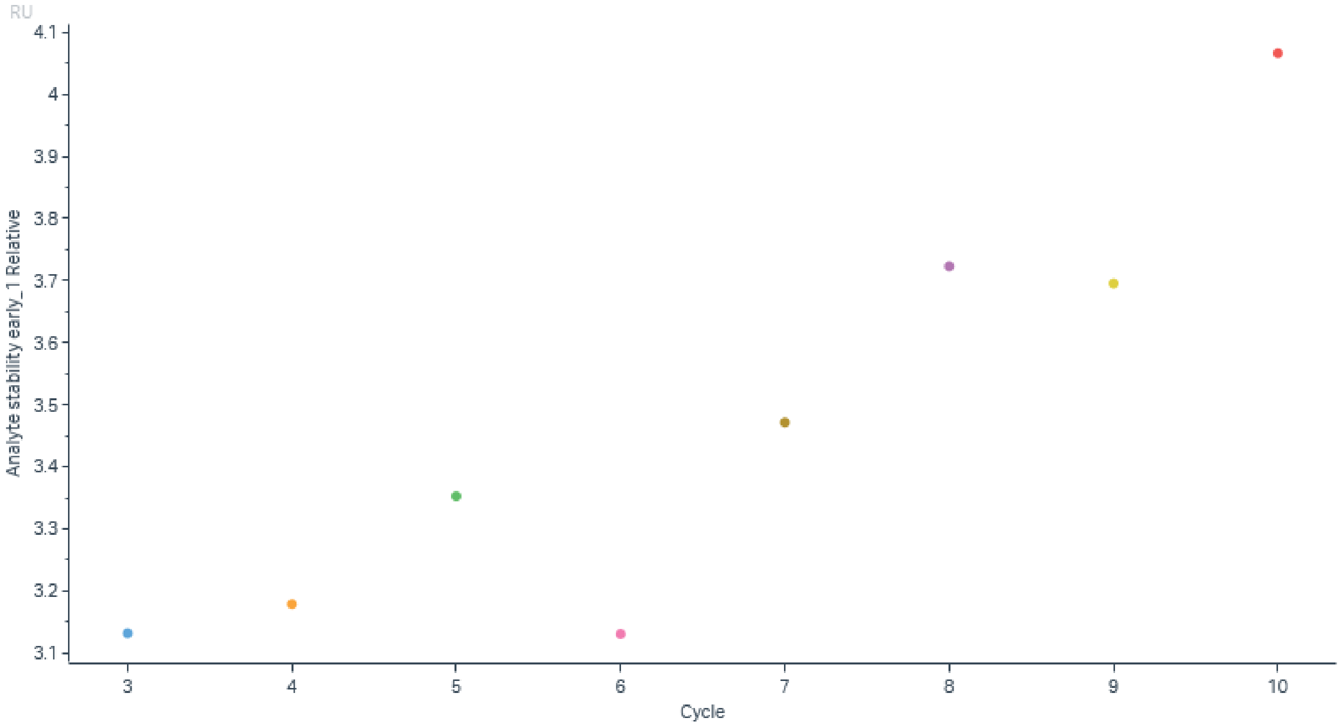

Channel 7

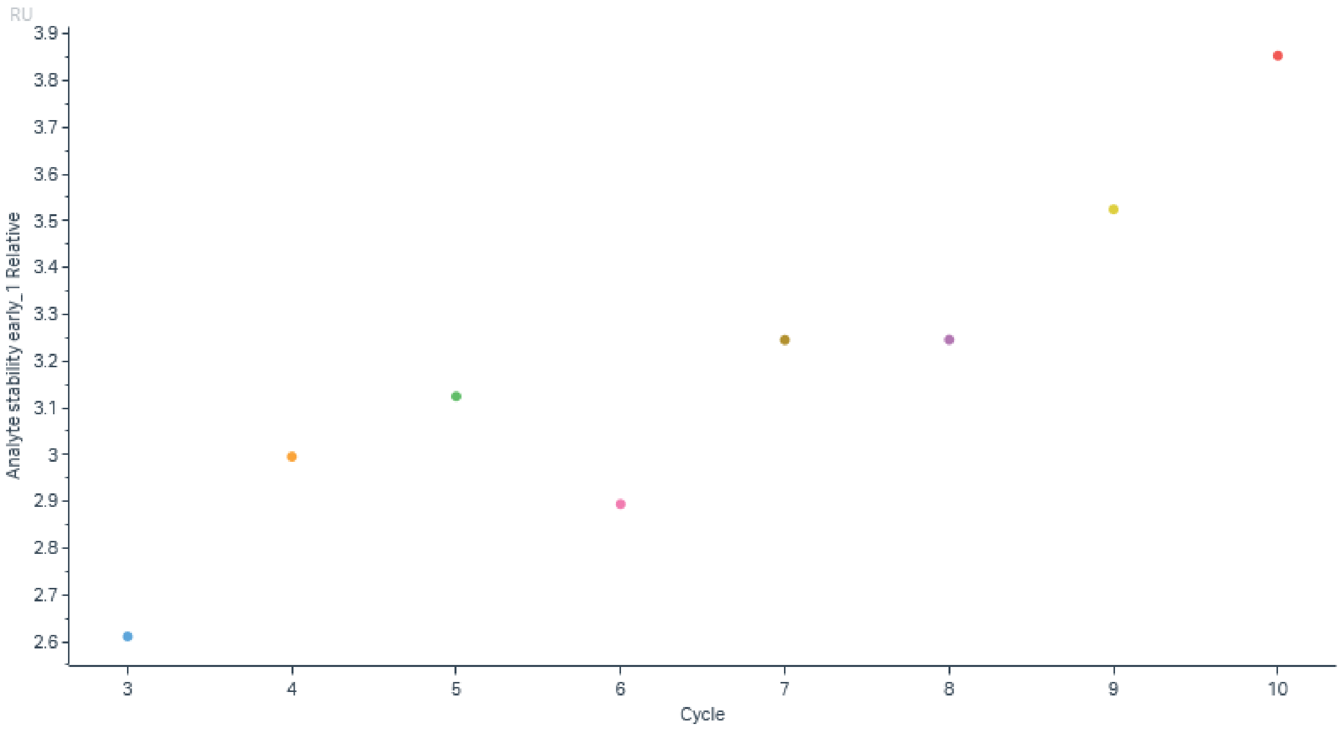
RU
3.9
3.8
3.7
3.6
3.5
3.4
3.3
3.2
3.1
3
2.9
2.8
2.7
2.6
Analyte stability early_1 Relative
3
4
5
6
7
8
9
10
Cycle

Plot table

#	Cycle	Channel	Sensorgram type	Analysis step purpose	Analysis step name	Excluded
1	3	5	Reference	Analysis	Analysis	
2	4	5	Reference	Analysis	Analysis	
3	5	5	Reference	Analysis	Analysis	
4	6	5	Reference	Analysis	Analysis	
5	7	5	Reference	Analysis	Analysis	
6	8	5	Reference	Analysis	Analysis	
7	9	5	Reference	Analysis	Analysis	
8	10	5	Reference	Analysis	Analysis	
9	3	6	Reference	Analysis	Analysis	
10	4	6	Reference	Analysis	Analysis	
11	5	6	Reference	Analysis	Analysis	
12	6	6	Reference	Analysis	Analysis	
13	7	6	Reference	Analysis	Analysis	
14	8	6	Reference	Analysis	Analysis	
15	9	6	Reference	Analysis	Analysis	
16	10	6	Reference	Analysis	Analysis	
17	3	7	Reference	Analysis	Analysis	
18	4	7	Reference	Analysis	Analysis	
19	5	7	Reference	Analysis	Analysis	
20	6	7	Reference	Analysis	Analysis	
21	7	7	Reference	Analysis	Analysis	
22	8	7	Reference	Analysis	Analysis	
23	9	7	Reference	Analysis	Analysis	
24	10	7	Reference	Analysis	Analysis	

Plot table (continued)

#	Curve markers	Analyte 1 Solution	Analyte stability early_1 Relative (RU)
1		FAB 1E6	4.5
2		FAB 1E6	4.8
3		FAB 1E6	4.9
4		FAB 1E6	4.6
5		FAB 1E6	4.9
6		FAB 1E6	5.2
7		FAB 1E6	5.4
8		FAB 1E6	5.5
9		FAB 1E6	3.1
10		FAB 1E6	3.2
11		FAB 1E6	3.4
12		FAB 1E6	3.1
13		FAB 1E6	3.5
14		FAB 1E6	3.7
15		FAB 1E6	3.7
16		FAB 1E6	4.1
17		FAB 1E6	2.6
18		FAB 1E6	3.0
19		FAB 1E6	3.1
20		FAB 1E6	2.9
21		FAB 1E6	3.2
22		FAB 1E6	3.2
23		FAB 1E6	3.5
24		FAB 1E6	3.9

Evaluation - Kinetics

Thumbnails

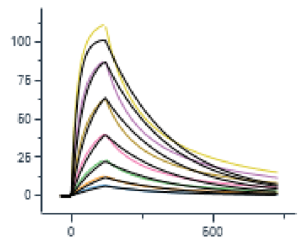

FAB 1E6; VB16T13 ECOLI; 1:1 binding

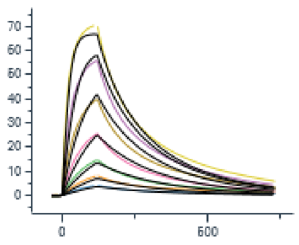

FAB 1E6; VB16T13 293F; 1:1 binding

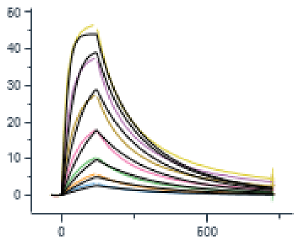

FAB 1E6; VB16T13 3T3; 1:1 binding

Charts

FAB 1E6; VB16T13 ECOLI; 1:1 binding

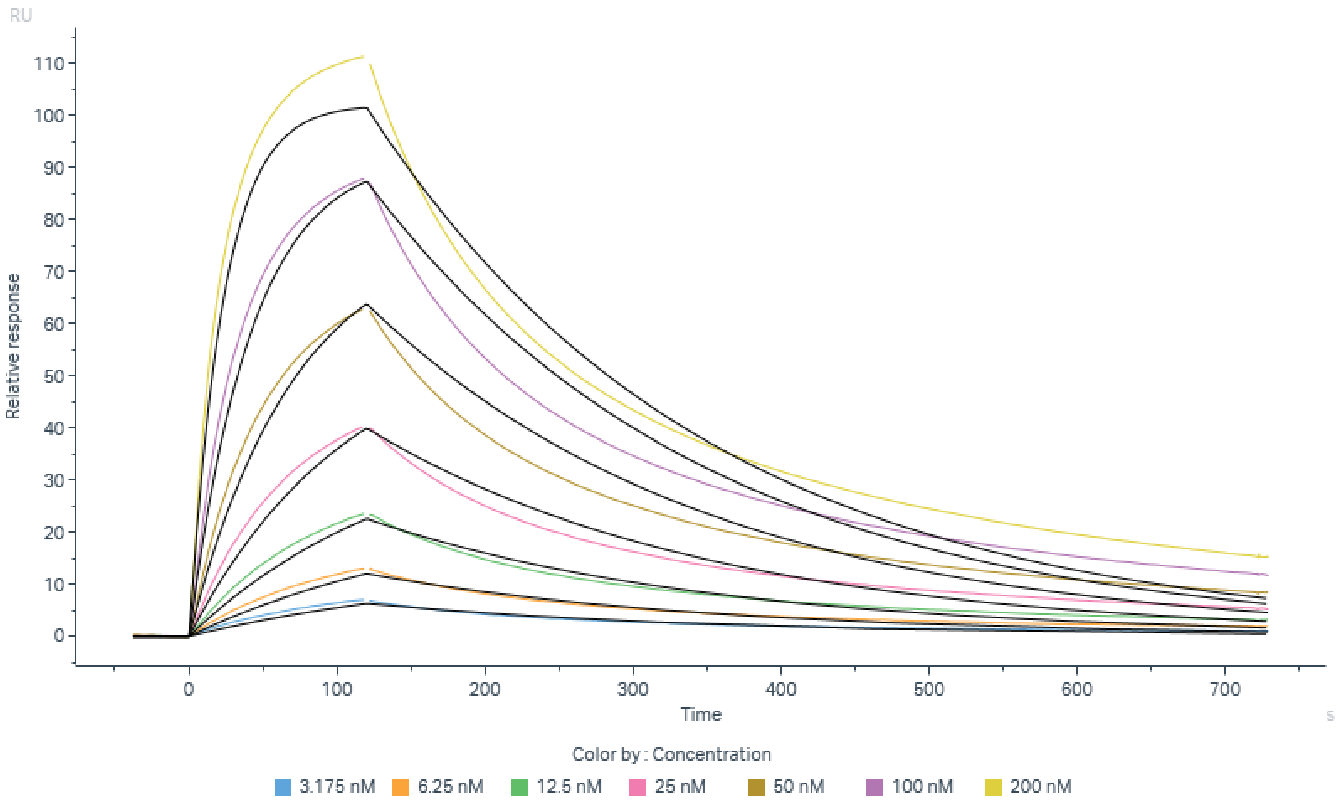

FAB 1E6; VB16T13 293F; 1:1 binding

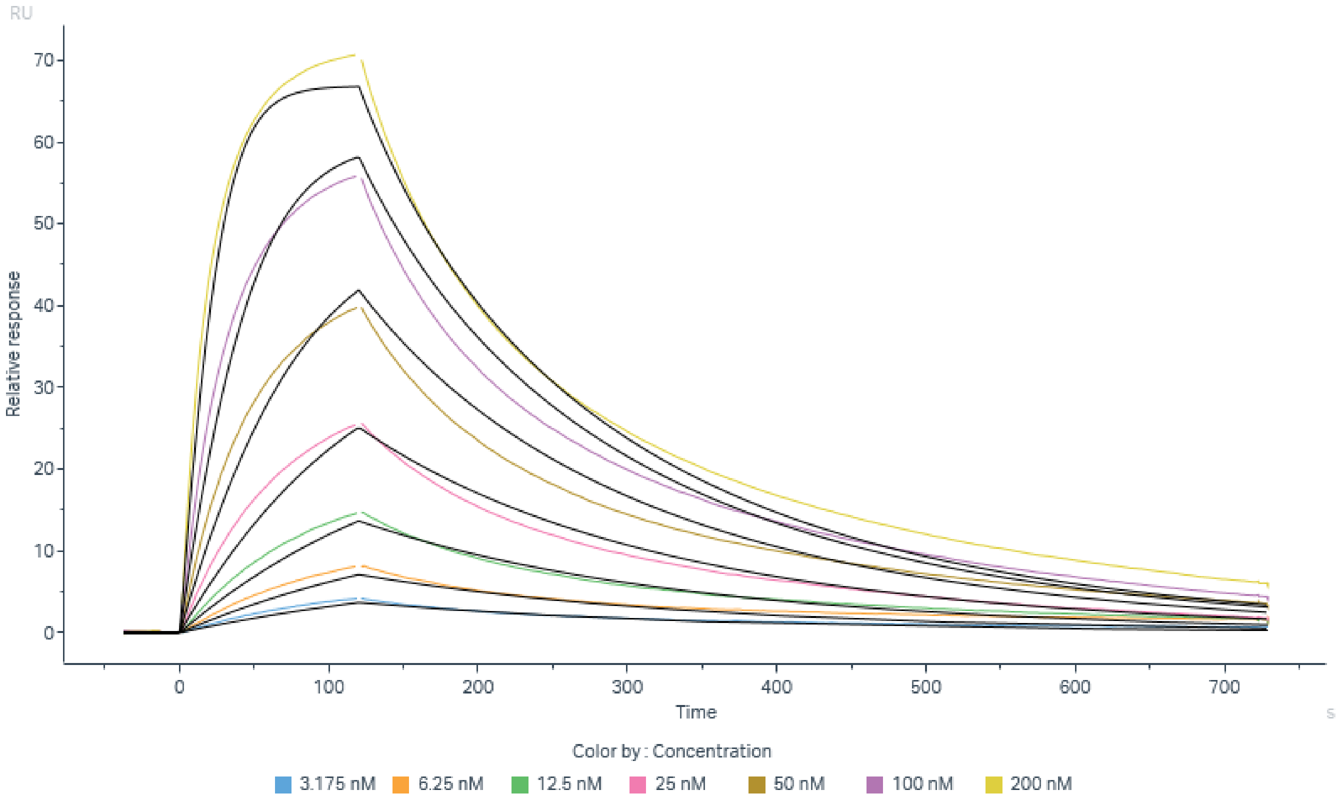

FAB 1E6; VB16T13 3T3; 1:1 binding

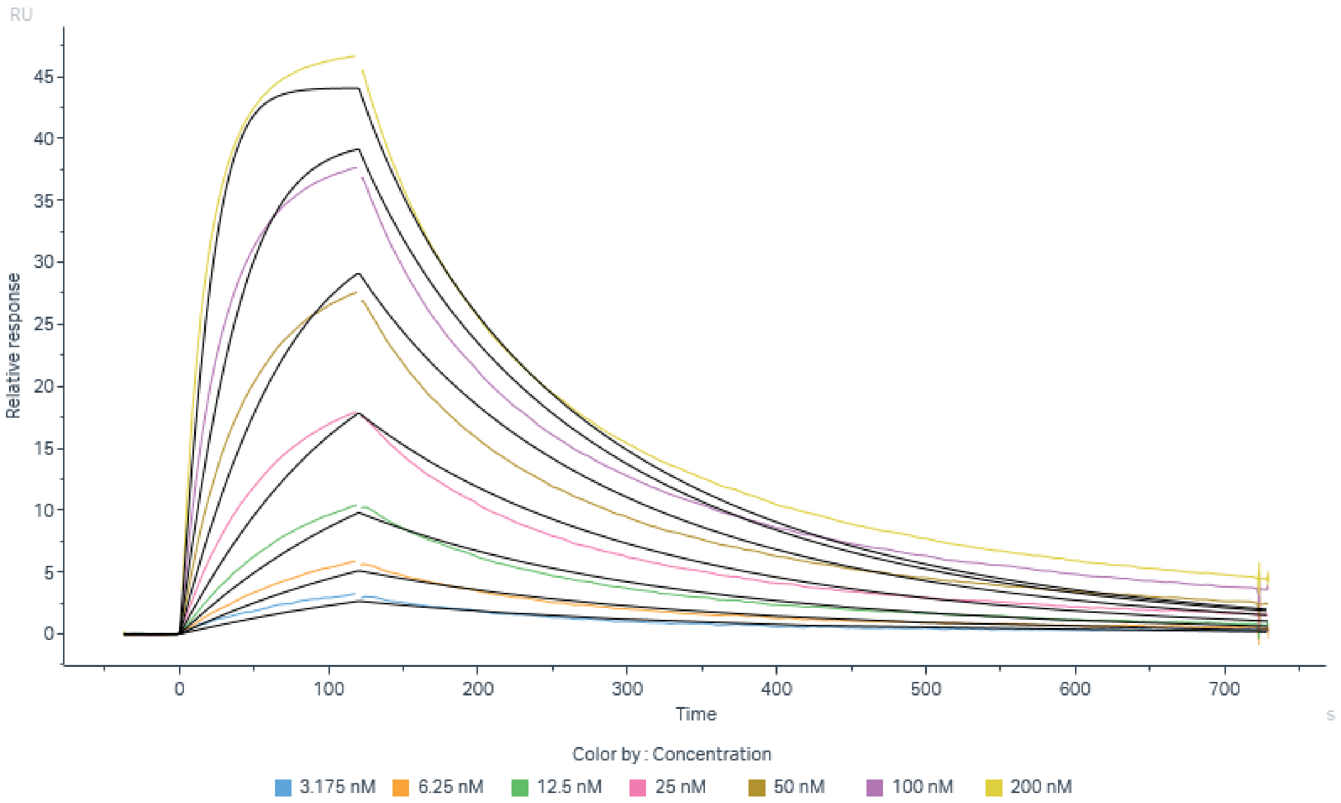

On-off rate chart

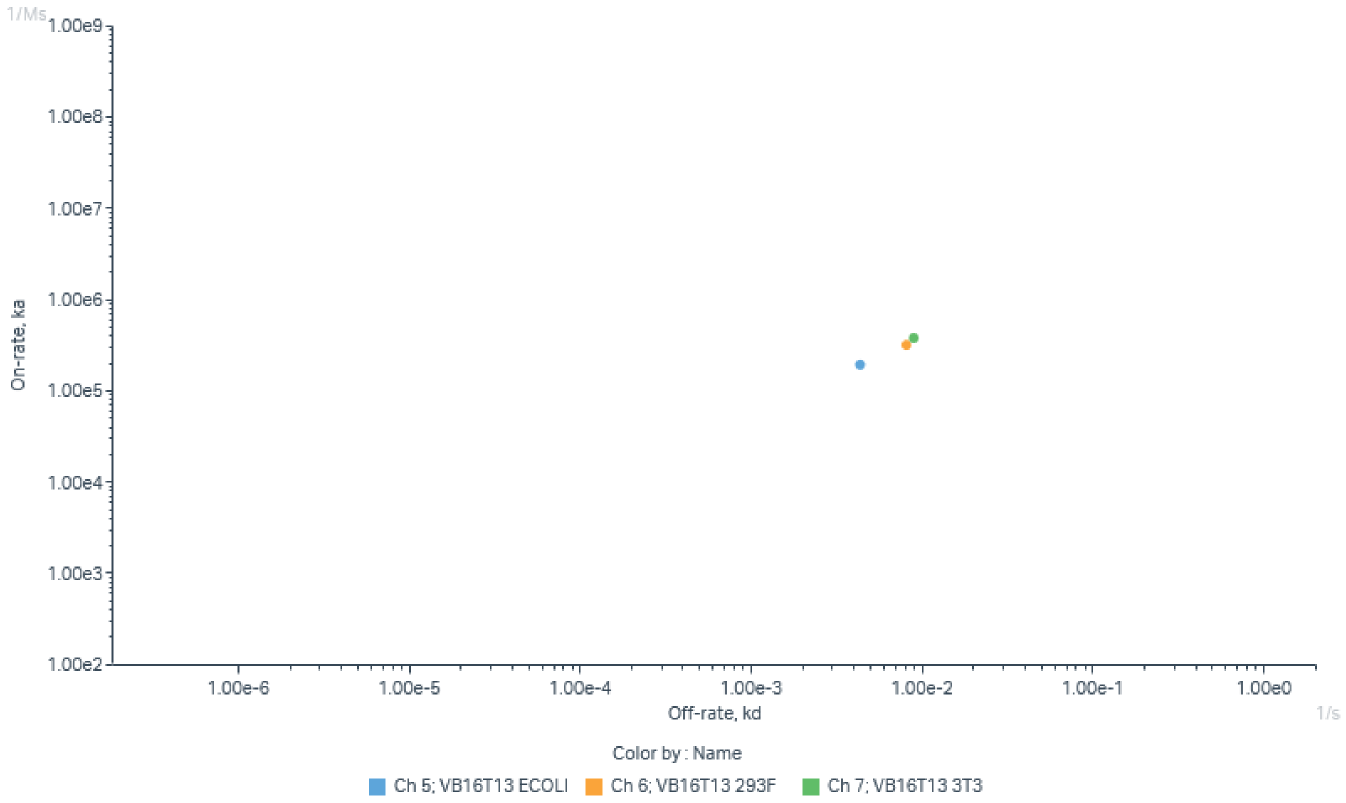

Result table

#	Group	General Kinetics model	Curve markers	Channel	Immobilized ligand	Injection variables Analyte 1 Solution
1	1	1:1 binding		5	VB16T13 ECOLI	FAB 1E6
2	2	1:1 binding		6	VB16T13 293F	FAB 1E6
3	3	1:1 binding		7	VB16T13 3T3	FAB 1E6

#	Quality Kinetics Chi² (RU²)	1:1 binding ka (1/Ms)	kd (1/s)	KD (M)	Rmax (RU)	tc
1	1.00e+01	1.94e+05	4.34e-03	2.24e-08	113.6	2.28e+09
2	2.24e+00	3.20e+05	8.09e-03	2.53e-08	75.3	8.48e+06
3	1.28e+00	3.80e+05	8.93e-03	2.35e-08	49.3	5.76e+06
