# Supplementary material for: A circular RNA vaccine induces durable and cross-protective immunity against Neisseria meningitidis serogroup B in mice
Source: PLoS Pathog. 2026 May 11;22(5):e1013741. doi: 10.1371/journal.ppat.1013741 (PMC13160355; doi:10.1371/journal.ppat.1013741)
Supplement: S1 Data — Images files. SPR PDF files. CE5200 PDF file. S1D Fig report. S1A Fig sequencing file. (ZIP) [file ppat.1013741.s016.zip › Raw data/SPR PDF files/FAB 4B3-5 VB16T13 ECOLI-6 VB16T13 293F-7 VB16T13 3t3-20260205 252026 65838 PM.pdf]

# Biacore Insight Evaluation

FAB 4B3-5 VB16T13 ECOLI-6 VB16T13 293F-7 VB16T13 3t3-20260205 2/5/2026 6:58:38 PM

## Evaluation

| | |
|---|---|
| Name | FAB 4B3-5 VB16T13 ECOLI-6 VB16T13 293F-7 VB16T13 3t3-20260205 2/5/2026 6:58:38 PM |
| Path | Root\FAB 4B3-5 VB16T13 ECOLI-6 VB16T13 293F-7 VB16T13 3t3-20260205 2/5/2026 6:58:38 PM |
| Modified by | DESKTOP-22AQC8N\Dell (DESKTOP-22AQC8N\Dell) |
| Date modified | 2/6/2026 5:27:11 PM |
| Created with | Biacore Insight Evaluation 4.0.8.19879 |
| Server name | .\SQLEXPRESS |
| Database id | Biacore Insight Database |

## Run(s) in this evaluation

## Run 1

| | |
|---|---|
| Name | FAB 4B3-5 VB16T13 ECOLI-6 VB16T13 293F-7 VB16T13 3t3-20260205 2/5/2026 6:58:38 PM |
| Path | Root\ZW\QIQI ZHANG\FAB 4B3-5 VB16T13 ECOLI-6 VB16T13 293F-7 VB16T13 3t3-20260205 2/5/2026 6:58:38 PM |
| Created by | DESKTOP-22AQC8N\Dell (DESKTOP-22AQC8N\Dell) |
| Method | FAB 4B3-5 VB16T13 ECOLI-6 VB16T13 293F-7 VB16T13 3t3-20260205 |
| Instrument type/ID | Biacore 8K+/2902034 |
| Cycles | 10 |
| Data collection rate | 10 Hz |
| Running buffer | Buffer |
| Start time | 2/6/2026 11:54:42 AM |
| End time | 2/6/2026 2:17:35 PM |

## Chip information

| | |
|---|---|
| Chip id | 10/20/2025 1:22:02 PM |
| Chip type | CM5 |
| Lot number | 10369187 |
| First dock date | 10/20/2025 1:23:00 PM |

## Run 1 - Immobilization results

Flow cell 1

| | Channel 1 | Channel 2 | Channel 3 | Channel 4 |
|---|---|---|---|---|
| Sensorgram type | Not used in run | Not used in run | Not used in run | Not used in run |
| Ligand | | | SA | SA |
| MW (Da) | | | | |
| Level (RU) | | | 6142.6 | 6088.1 |
| Run name | | | Immobilization 1/8/2026 10:43:22 AM | Immobilization 1/8/2026 10:43:22 AM |
| Date and time | | | 1/8/2026 10:44:03 AM | 1/8/2026 10:44:03 AM |

| | Channel 5 | Channel 6 | Channel 7 | Channel 8 |
|---|---|---|---|---|
| Sensorgram type | Reference | Reference | Reference | Not used in run |
| Ligand | Activation/Deactivation | Activation/Deactivation | Activation/Deactivation | |
| MW (Da) | | | | |
| Level (RU) | | | | |
| Run name | Immobilization 20260205 12:22:47 PM 2/5/2026 6:14:22 PM | Immobilization 20260205 12:22:47 PM 2/5/2026 6:14:22 PM | Immobilization 20260205 12:22:47 PM 2/5/2026 6:14:22 PM | |
| Date and time | 2/5/2026 6:14:26 PM | 2/5/2026 6:14:26 PM | 2/5/2026 6:14:26 PM | |

## Run 1 - Immobilization results

Flow cell 2

| | Channel 1 | Channel 2 | Channel 3 | Channel 4 |
|---|---|---|---|---|
| Sensorgram type | Not used in run | Not used in run | Not used in run | Not used in run |
| Ligand | Ligand 1 | Ligand 2 | SA | SA |
| MW (Da) | | | | |
| Level (RU) | 3483.5 | 5468.2 | 5936.3 | 6024.2 |
| Run name | Immobilization 10/20/2025 1:40:59 PM | Immobilization 10/20/2025 1:40:59 PM | Immobilization 1/8/2026 10:43:22 AM | Immobilization 1/8/2026 10:43:22 AM |
| Date and time | 10/20/2025 1:41:04 PM | 10/20/2025 1:41:04 PM | 1/8/2026 10:44:03 AM | 1/8/2026 10:44:03 AM |

| | Channel 5 | Channel 6 | Channel 7 | Channel 8 |
|---|---|---|---|---|
| Sensorgram type | Active | Active | Active | Not used in run |
| Ligand | VB16T13 ECOLI | VB16T13 293F | VB16T13 3T3 | |
| MW (Da) | | | | |
| Level (RU) | 288.4 | 256.3 | 323.2 | |
| Run name | Immobilization 20260205 12:22:47 PM 2/5/2026 6:14:22 PM | Immobilization 20260205 12:22:47 PM 2/5/2026 6:14:22 PM | Immobilization 20260205 12:22:47 PM 2/5/2026 6:14:22 PM | |
| Date and time | 2/5/2026 6:14:26 PM | 2/5/2026 6:14:26 PM | 2/5/2026 6:14:26 PM | |

# Run 1 - Method summary

## 2 Startup

### Analyte 1

Buffer, Contact time 120 s, Diss time 60 s, Flow rate 30 µl/min, Both flow cells

### Regeneration 1

1.5, Contact time 30 s, Flow rate 30 µl/min, Both flow cells

## 8 Analysis

### Analyte 1

Variable solution, Contact time 120 s, Diss time 600 s, Flow rate 30 µl/min, Variable conc, Both flow cells

### Regeneration 1

1.5, Contact time 90 s, Flow rate 30 µl/min, Both flow cells

# QC - Sensorgram

## Thumbnails

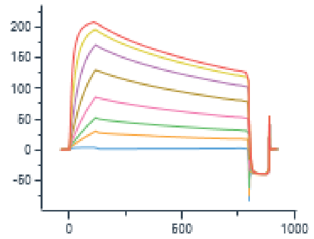


Channel 5

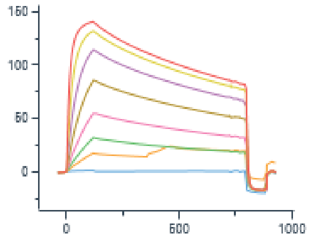


Channel 6

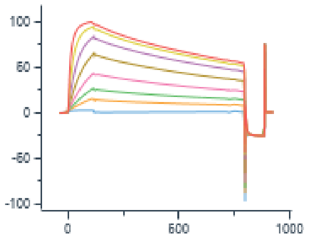


Channel 7

## Charts

Channel 5

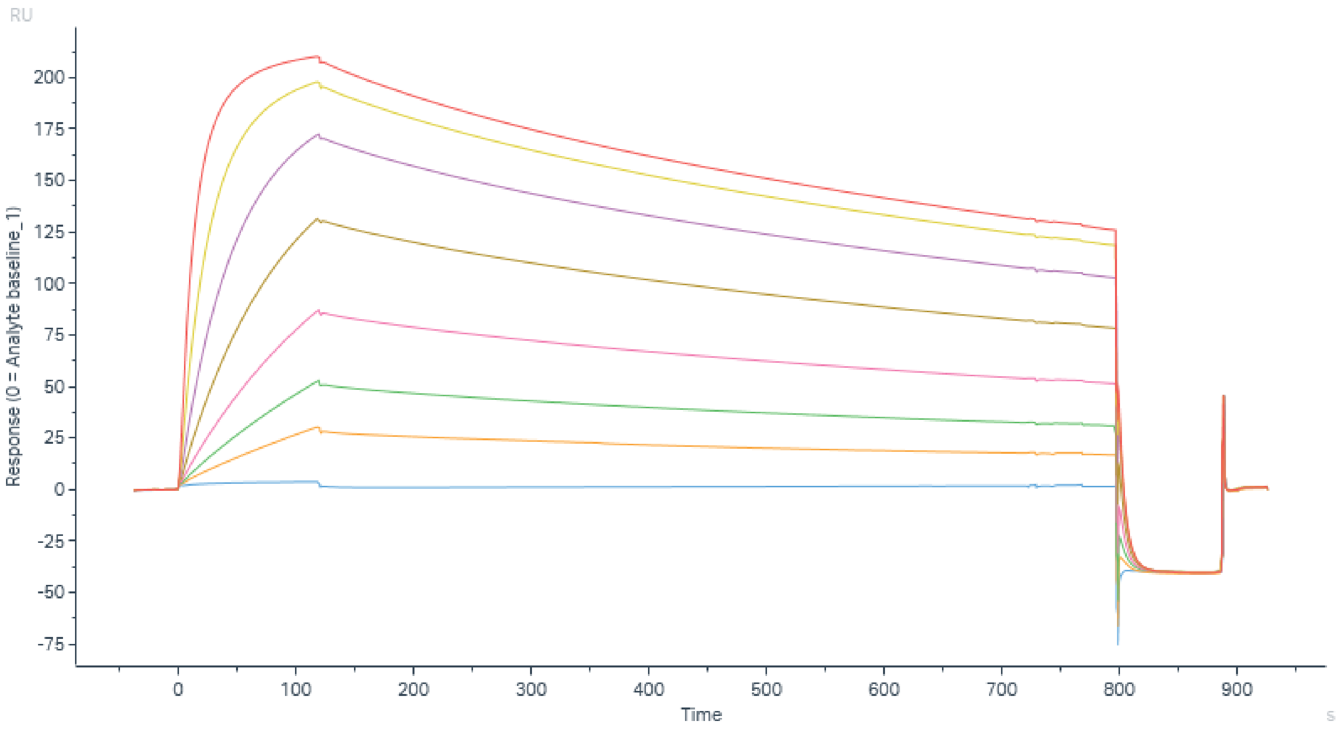


Channel 6

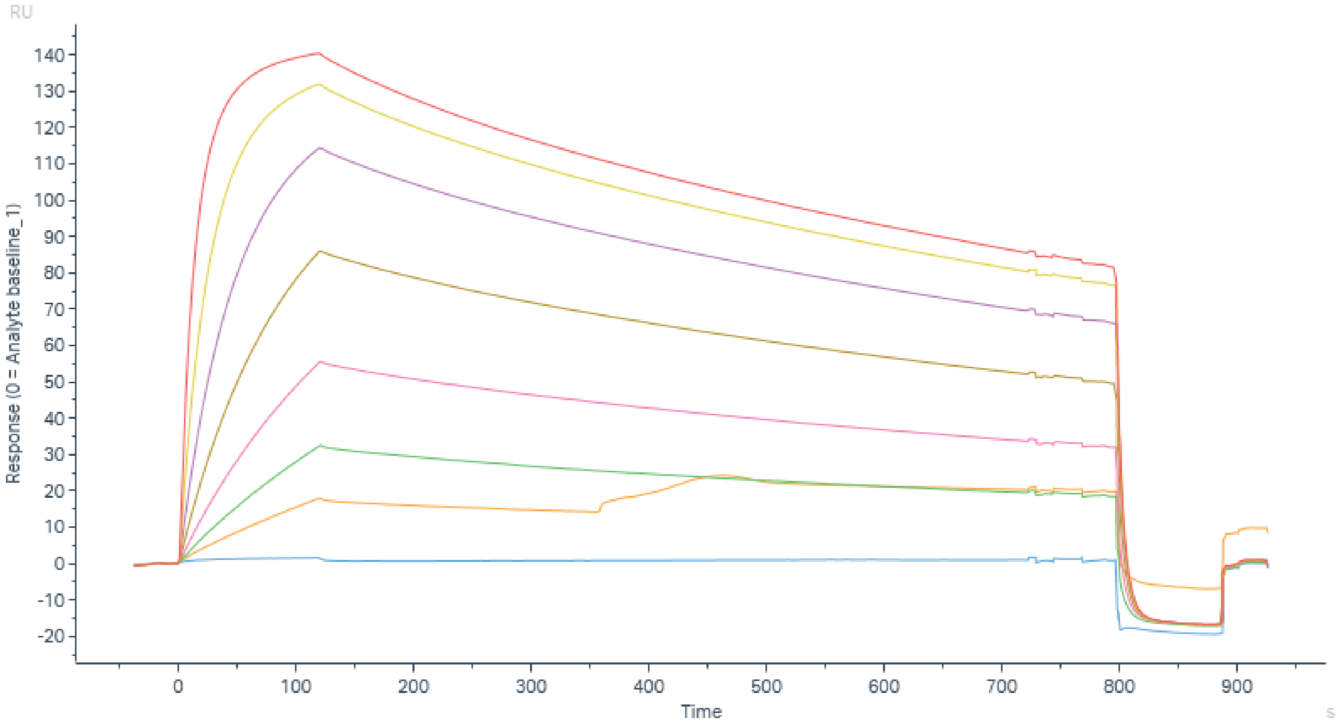

## Channel 7

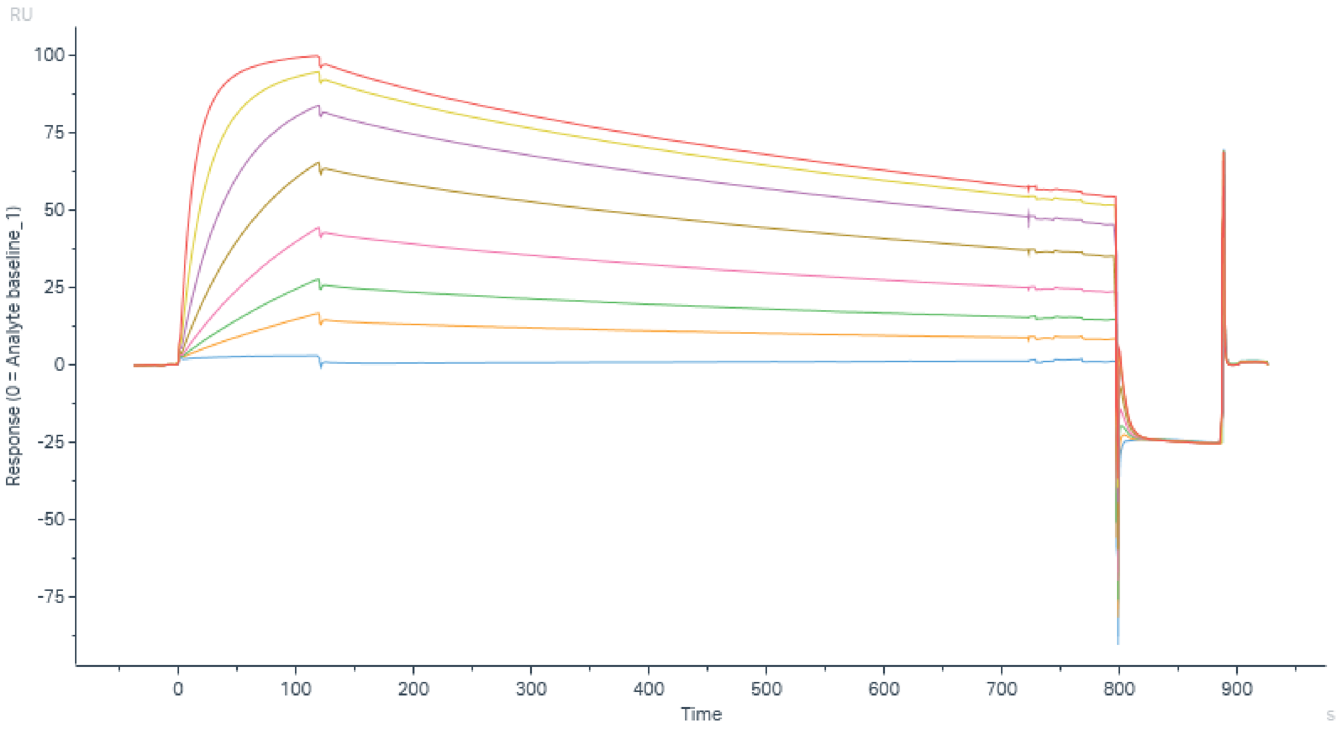
RU
100
75
50
25
0
-25
-50
-75
Response (0 = Analyte baseline_1)
0
100
200
300
400
500
600
700
800
900
Time
s

## Sensorgram table

| # | Cycle | Channel | Sensorgram type | Analysis step purpose | Analysis step name | Excluded |
|---|---|---|---|---|---|---|
| 1 | 3 | 5 | Reference subtracted | Analysis | Analysis | |
| 2 | 4 | 5 | Reference subtracted | Analysis | Analysis | |
| 3 | 5 | 5 | Reference subtracted | Analysis | Analysis | |
| 4 | 6 | 5 | Reference subtracted | Analysis | Analysis | |
| 5 | 7 | 5 | Reference subtracted | Analysis | Analysis | |
| 6 | 8 | 5 | Reference subtracted | Analysis | Analysis | |
| 7 | 9 | 5 | Reference subtracted | Analysis | Analysis | |
| 8 | 10 | 5 | Reference subtracted | Analysis | Analysis | |
| 9 | 3 | 6 | Reference subtracted | Analysis | Analysis | |
| 10 | 4 | 6 | Reference subtracted | Analysis | Analysis | |
| 11 | 5 | 6 | Reference subtracted | Analysis | Analysis | |
| 12 | 6 | 6 | Reference subtracted | Analysis | Analysis | |
| 13 | 7 | 6 | Reference subtracted | Analysis | Analysis | |
| 14 | 8 | 6 | Reference subtracted | Analysis | Analysis | |
| 15 | 9 | 6 | Reference subtracted | Analysis | Analysis | |
| 16 | 10 | 6 | Reference subtracted | Analysis | Analysis | |
| 17 | 3 | 7 | Reference subtracted | Analysis | Analysis | |
| 18 | 4 | 7 | Reference subtracted | Analysis | Analysis | |
| 19 | 5 | 7 | Reference subtracted | Analysis | Analysis | |
| 20 | 6 | 7 | Reference subtracted | Analysis | Analysis | |
| 21 | 7 | 7 | Reference subtracted | Analysis | Analysis | |
| 22 | 8 | 7 | Reference subtracted | Analysis | Analysis | |
| 23 | 9 | 7 | Reference subtracted | Analysis | Analysis | |
| 24 | 10 | 7 | Reference subtracted | Analysis | Analysis | |

## Sensorgram table (continued)

| # | Curve markers | Analyte 1 Solution | Analyte 1 Concentration (nM) | Analyte binding late_1 Relative (RU) |
|---|---|---|---|---|
| 1 | | FAB 4B3 | 0 | 3.7 |
| 2 | | FAB 4B3 | 3.175 | 29.7 |
| 3 | | FAB 4B3 | 6.25 | 51.5 |
| 4 | | FAB 4B3 | 12.5 | 85.2 |
| 5 | | FAB 4B3 | 25 | 129.8 |
| 6 | | FAB 4B3 | 50 | 171.0 |
| 7 | | FAB 4B3 | 100 | 197.3 |
| 8 | | FAB 4B3 | 200 | 209.9 |
| 9 | | FAB 4B3 | 0 | 1.6 |
| 10 | | FAB 4B3 | 3.175 | 17.4 |
| 11 | | FAB 4B3 | 6.25 | 31.3 |
| 12 | | FAB 4B3 | 12.5 | 53.9 |
| 13 | | FAB 4B3 | 25 | 84.2 |
| 14 | | FAB 4B3 | 50 | 113.2 |
| 15 | | FAB 4B3 | 100 | 131.5 |
| 16 | | FAB 4B3 | 200 | 140.3 |
| 17 | | FAB 4B3 | 0 | 2.9 |
| 18 | | FAB 4B3 | 3.175 | 16.3 |
| 19 | | FAB 4B3 | 6.25 | 27.0 |
| 20 | | FAB 4B3 | 12.5 | 43.4 |
| 21 | | FAB 4B3 | 25 | 64.3 |
| 22 | | FAB 4B3 | 50 | 83.0 |
| 23 | | FAB 4B3 | 100 | 94.2 |
| 24 | | FAB 4B3 | 200 | 99.6 |

# QC - Baseline

## Thumbnails

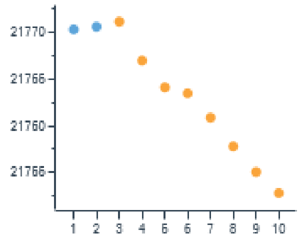

Channel 5

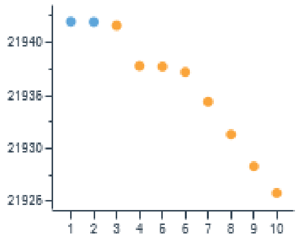

Channel 6

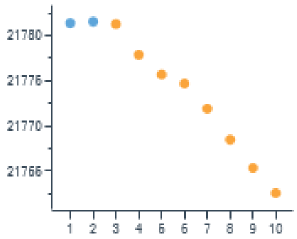

Channel 7

## Charts

Channel 5

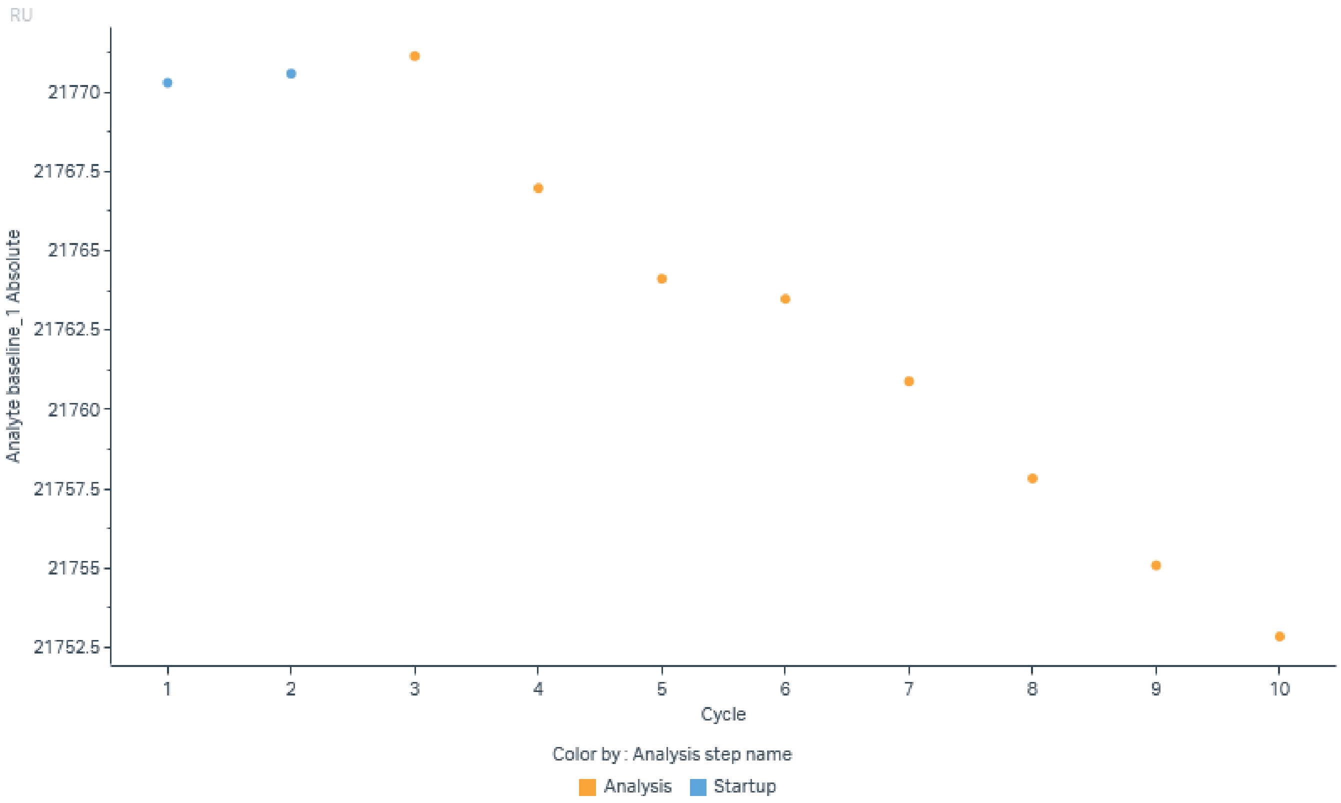


Channel 6

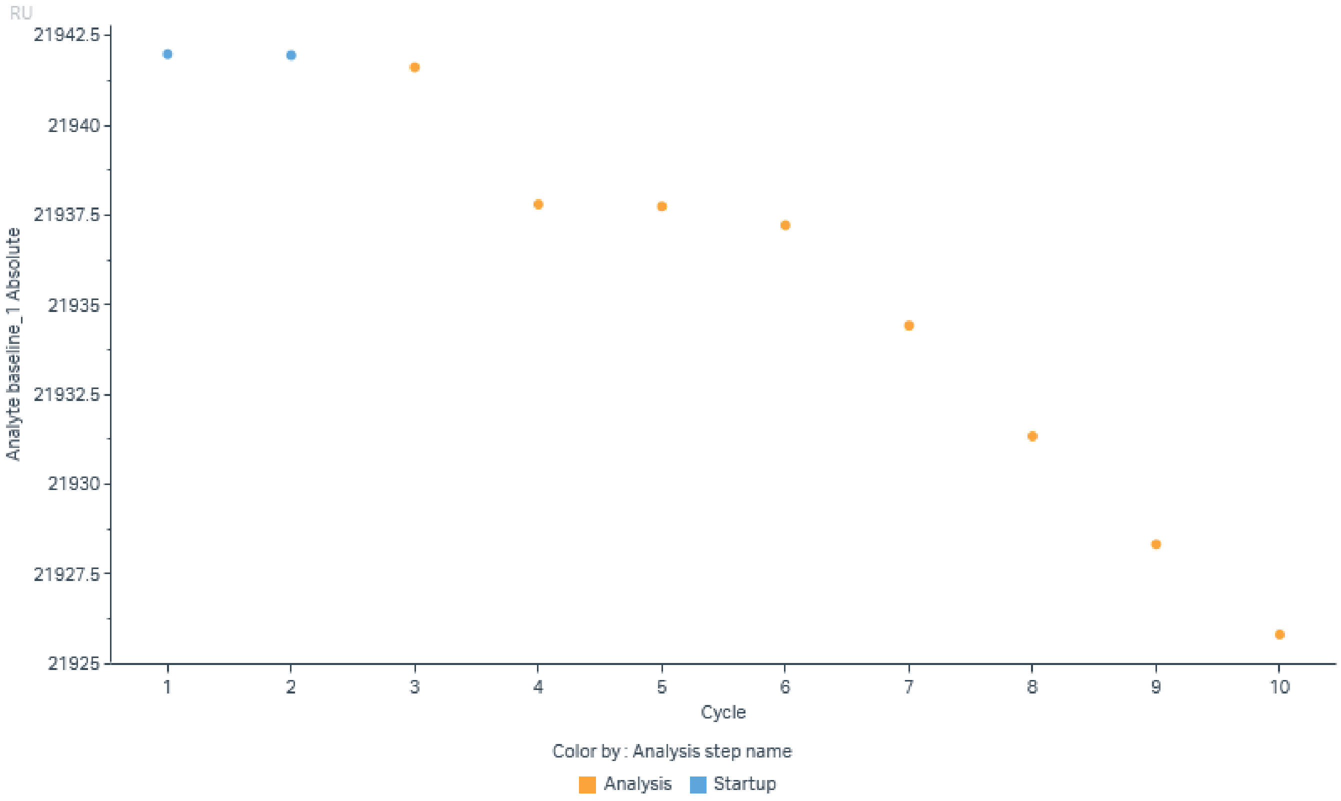

## Channel 7

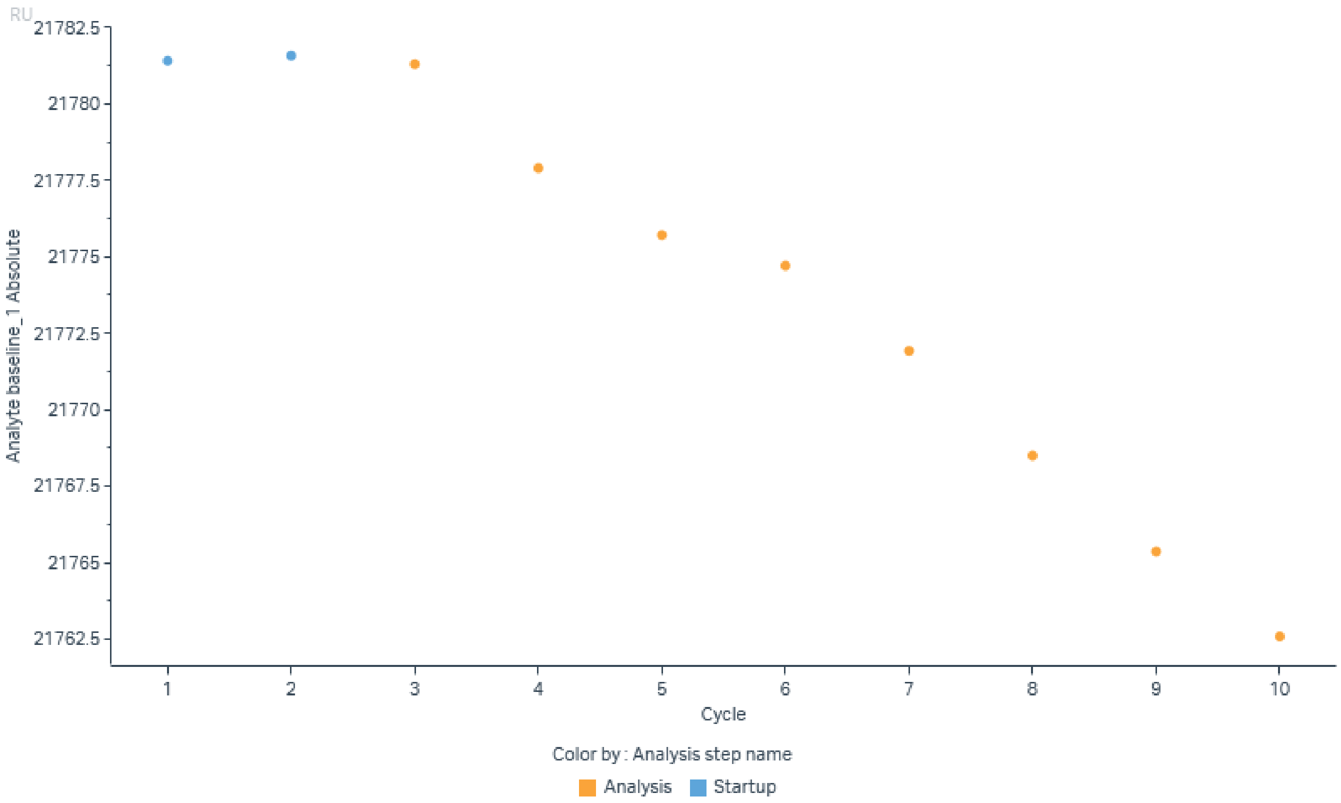
RU
21782.5
21780
21777.5
21775
21772.5
21770
21767.5
21765
21762.5
Analyte baseline_1 Absolute
1
2
3
4
5
6
7
8
9
10
Cycle
Color by : Analysis step name
Analysis
Startup

## Plot table

| # | Cycle | Channel | Sensorgram type | Analysis step purpose | Analysis step name | Excluded |
|---|---|---|---|---|---|---|
| 1 | 1 | 5 | Active | Startup | Startup | |
| 2 | 2 | 5 | Active | Startup | Startup | |
| 3 | 3 | 5 | Active | Analysis | Analysis | |
| 4 | 4 | 5 | Active | Analysis | Analysis | |
| 5 | 5 | 5 | Active | Analysis | Analysis | |
| 6 | 6 | 5 | Active | Analysis | Analysis | |
| 7 | 7 | 5 | Active | Analysis | Analysis | |
| 8 | 8 | 5 | Active | Analysis | Analysis | |
| 9 | 9 | 5 | Active | Analysis | Analysis | |
| 10 | 10 | 5 | Active | Analysis | Analysis | |
| 11 | 1 | 6 | Active | Startup | Startup | |
| 12 | 2 | 6 | Active | Startup | Startup | |
| 13 | 3 | 6 | Active | Analysis | Analysis | |
| 14 | 4 | 6 | Active | Analysis | Analysis | |
| 15 | 5 | 6 | Active | Analysis | Analysis | |
| 16 | 6 | 6 | Active | Analysis | Analysis | |
| 17 | 7 | 6 | Active | Analysis | Analysis | |
| 18 | 8 | 6 | Active | Analysis | Analysis | |
| 19 | 9 | 6 | Active | Analysis | Analysis | |
| 20 | 10 | 6 | Active | Analysis | Analysis | |
| 21 | 1 | 7 | Active | Startup | Startup | |
| 22 | 2 | 7 | Active | Startup | Startup | |
| 23 | 3 | 7 | Active | Analysis | Analysis | |
| 24 | 4 | 7 | Active | Analysis | Analysis | |
| 25 | 5 | 7 | Active | Analysis | Analysis | |
| 26 | 6 | 7 | Active | Analysis | Analysis | |
| 27 | 7 | 7 | Active | Analysis | Analysis | |
| 28 | 8 | 7 | Active | Analysis | Analysis | |
| 29 | 9 | 7 | Active | Analysis | Analysis | |
| 30 | 10 | 7 | Active | Analysis | Analysis | |

## Plot table (continued)

| # | Curve markers | Analyte 1 Solution | Analyte baseline_1 Absolute (RU) |
|---|---|---|---|
| 1 | | Buffer | 21770.3 |
| 2 | | Buffer | 21770.6 |
| 3 | | FAB 4B3 | 21771.1 |
| 4 | | FAB 4B3 | 21767.0 |
| 5 | | FAB 4B3 | 21764.1 |
| 6 | | FAB 4B3 | 21763.5 |
| 7 | | FAB 4B3 | 21760.9 |
| 8 | | FAB 4B3 | 21757.8 |
| 9 | | FAB 4B3 | 21755.1 |
| 10 | | FAB 4B3 | 21752.8 |
| 11 | | Buffer | 21942.0 |
| 12 | | Buffer | 21942.0 |
| 13 | | FAB 4B3 | 21941.6 |
| 14 | | FAB 4B3 | 21937.8 |
| 15 | | FAB 4B3 | 21937.7 |
| 16 | | FAB 4B3 | 21937.2 |
| 17 | | FAB 4B3 | 21934.4 |
| 18 | | FAB 4B3 | 21931.3 |
| 19 | | FAB 4B3 | 21928.3 |
| 20 | | FAB 4B3 | 21925.8 |
| 21 | | Buffer | 21781.4 |
| 22 | | Buffer | 21781.6 |
| 23 | | FAB 4B3 | 21781.3 |
| 24 | | FAB 4B3 | 21777.9 |
| 25 | | FAB 4B3 | 21775.7 |
| 26 | | FAB 4B3 | 21774.7 |
| 27 | | FAB 4B3 | 21771.9 |
| 28 | | FAB 4B3 | 21768.5 |
| 29 | | FAB 4B3 | 21765.4 |
| 30 | | FAB 4B3 | 21762.6 |

# QC - Binding to reference

## Thumbnails

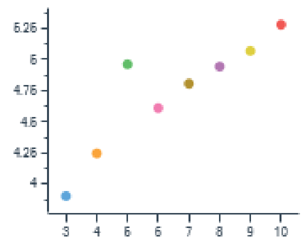


Channel 5

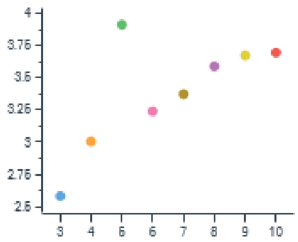


Channel 6

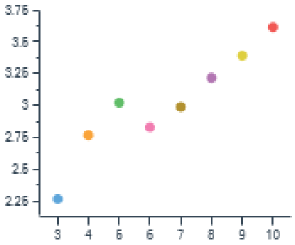


Channel 7

## Charts

Channel 5

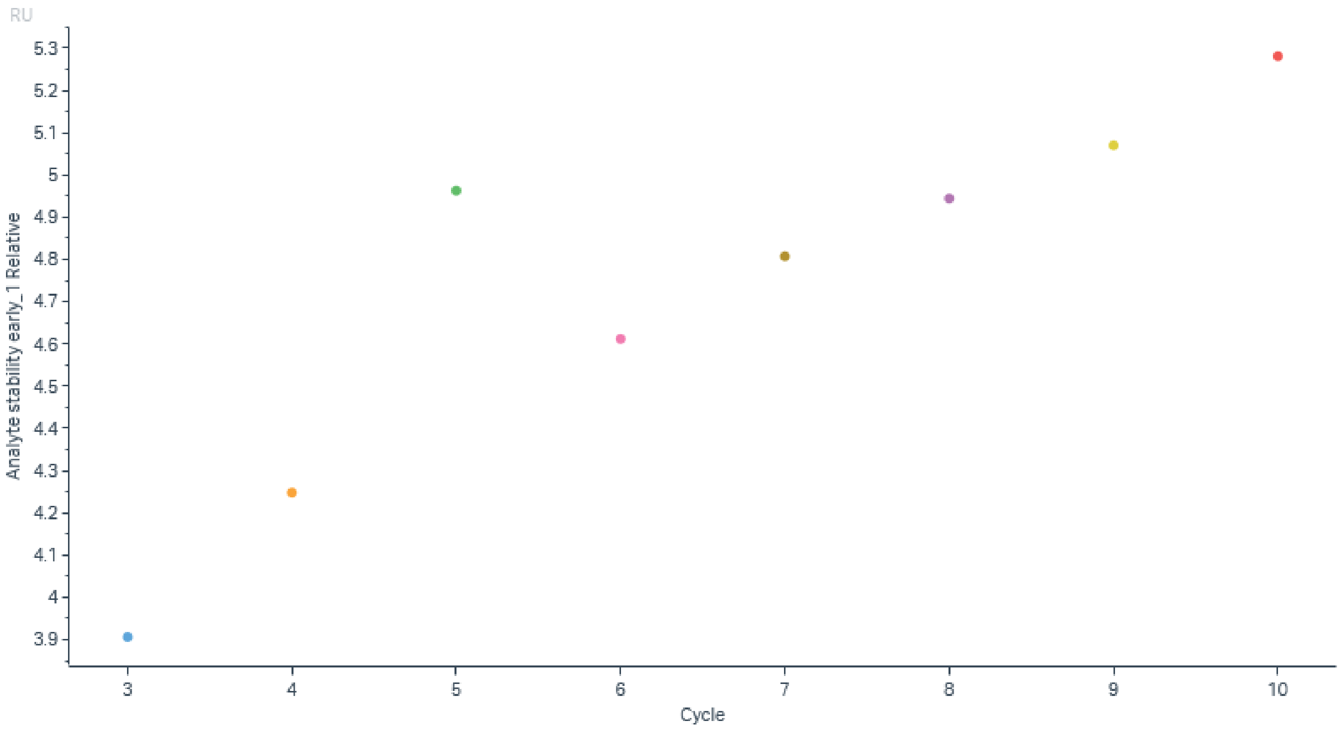


Channel 6

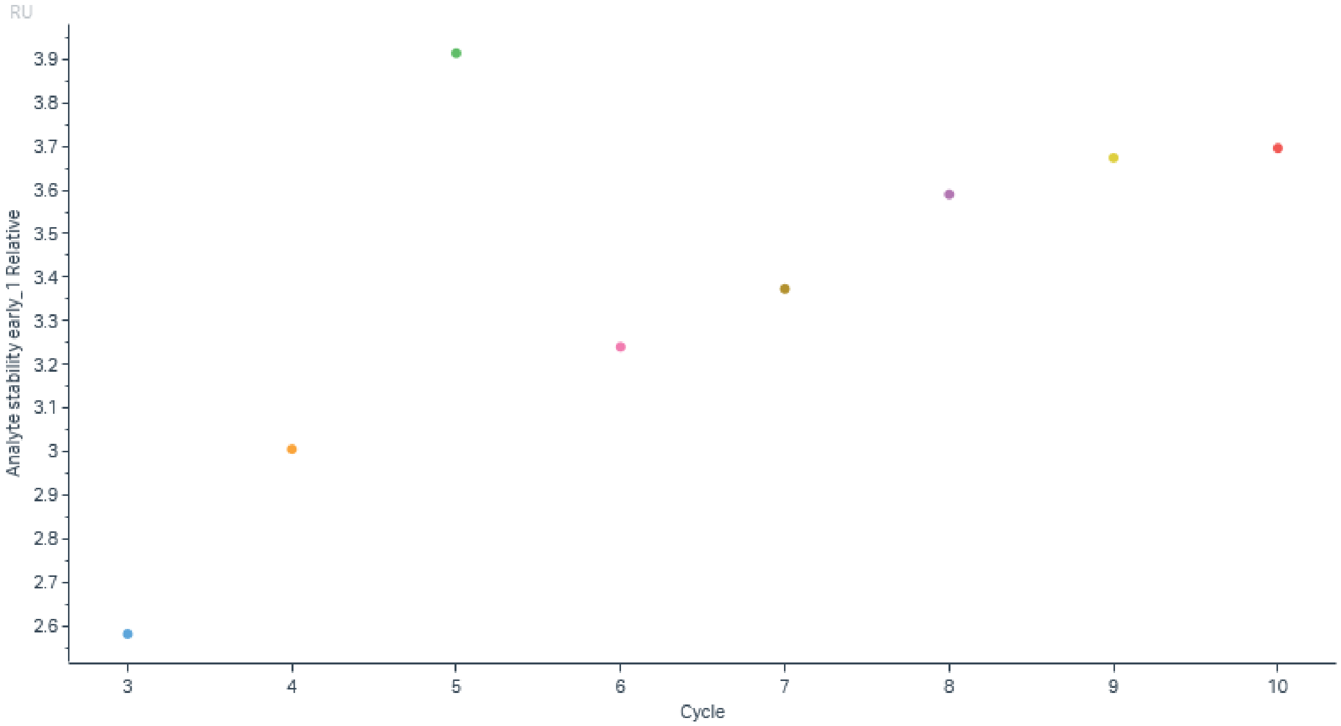

Channel 7

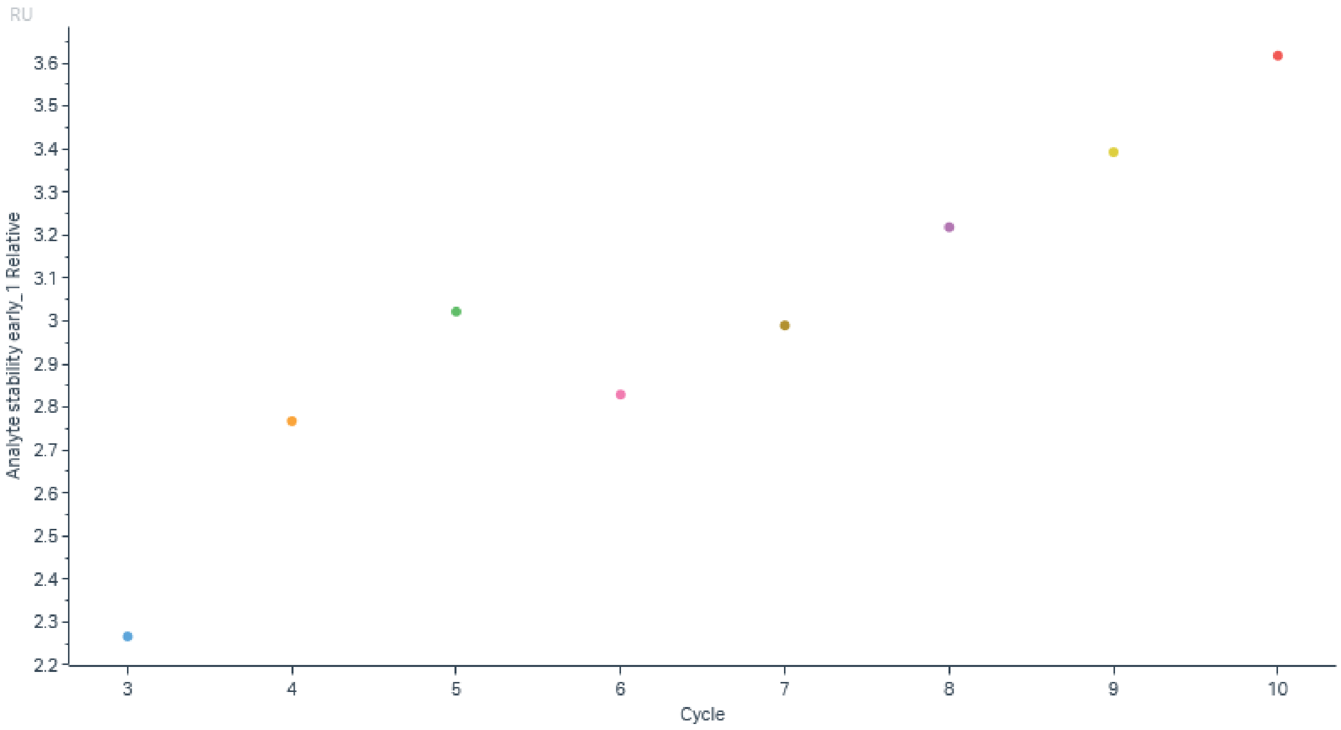
RU
Analyte stability early_1 Relative
3.6
3.5
3.4
3.3
3.2
3.1
3
2.9
2.8
2.7
2.6
2.5
2.4
2.3
2.2
3
4
5
6
7
8
9
10
Cycle

## Plot table

| # | Cycle | Channel | Sensorgram type | Analysis step purpose | Analysis step name | Excluded |
|---|---|---|---|---|---|---|
| 1 | 3 | 5 | Reference | Analysis | Analysis | |
| 2 | 4 | 5 | Reference | Analysis | Analysis | |
| 3 | 5 | 5 | Reference | Analysis | Analysis | |
| 4 | 6 | 5 | Reference | Analysis | Analysis | |
| 5 | 7 | 5 | Reference | Analysis | Analysis | |
| 6 | 8 | 5 | Reference | Analysis | Analysis | |
| 7 | 9 | 5 | Reference | Analysis | Analysis | |
| 8 | 10 | 5 | Reference | Analysis | Analysis | |
| 9 | 3 | 6 | Reference | Analysis | Analysis | |
| 10 | 4 | 6 | Reference | Analysis | Analysis | |
| 11 | 5 | 6 | Reference | Analysis | Analysis | |
| 12 | 6 | 6 | Reference | Analysis | Analysis | |
| 13 | 7 | 6 | Reference | Analysis | Analysis | |
| 14 | 8 | 6 | Reference | Analysis | Analysis | |
| 15 | 9 | 6 | Reference | Analysis | Analysis | |
| 16 | 10 | 6 | Reference | Analysis | Analysis | |
| 17 | 3 | 7 | Reference | Analysis | Analysis | |
| 18 | 4 | 7 | Reference | Analysis | Analysis | |
| 19 | 5 | 7 | Reference | Analysis | Analysis | |
| 20 | 6 | 7 | Reference | Analysis | Analysis | |
| 21 | 7 | 7 | Reference | Analysis | Analysis | |
| 22 | 8 | 7 | Reference | Analysis | Analysis | |
| 23 | 9 | 7 | Reference | Analysis | Analysis | |
| 24 | 10 | 7 | Reference | Analysis | Analysis | |

## Plot table (continued)

| # | Curve markers | Analyte 1 Solution | Analyte stability early_1 Relative (RU) |
|---|---|---|---|
| 1 | | FAB 4B3 | 3.9 |
| 2 | | FAB 4B3 | 4.2 |
| 3 | | FAB 4B3 | 5.0 |
| 4 | | FAB 4B3 | 4.6 |
| 5 | | FAB 4B3 | 4.8 |
| 6 | | FAB 4B3 | 4.9 |
| 7 | | FAB 4B3 | 5.1 |
| 8 | | FAB 4B3 | 5.3 |
| 9 | | FAB 4B3 | 2.6 |
| 10 | | FAB 4B3 | 3.0 |
| 11 | | FAB 4B3 | 3.9 |
| 12 | | FAB 4B3 | 3.2 |
| 13 | | FAB 4B3 | 3.4 |
| 14 | | FAB 4B3 | 3.6 |
| 15 | | FAB 4B3 | 3.7 |
| 16 | | FAB 4B3 | 3.7 |
| 17 | | FAB 4B3 | 2.3 |
| 18 | | FAB 4B3 | 2.8 |
| 19 | | FAB 4B3 | 3.0 |
| 20 | | FAB 4B3 | 2.8 |
| 21 | | FAB 4B3 | 3.0 |
| 22 | | FAB 4B3 | 3.2 |
| 23 | | FAB 4B3 | 3.4 |
| 24 | | FAB 4B3 | 3.6 |

# Evaluation - Kinetics

## Thumbnails

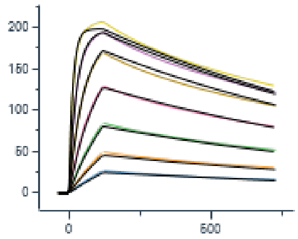


FAB 4B3; VB16T13 ECOLI; 1:1 binding

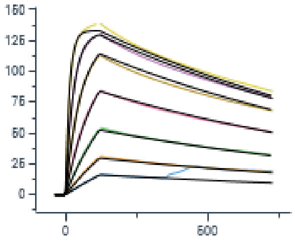


FAB 4B3; VB16T13 293F; 1:1 binding

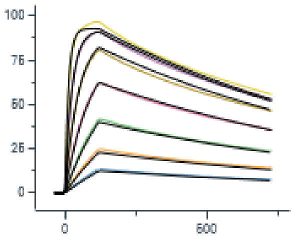


FAB 4B3; VB16T13 3T3; 1:1 binding

## Charts

FAB 4B3; VB16T13 ECOLI; 1:1 binding

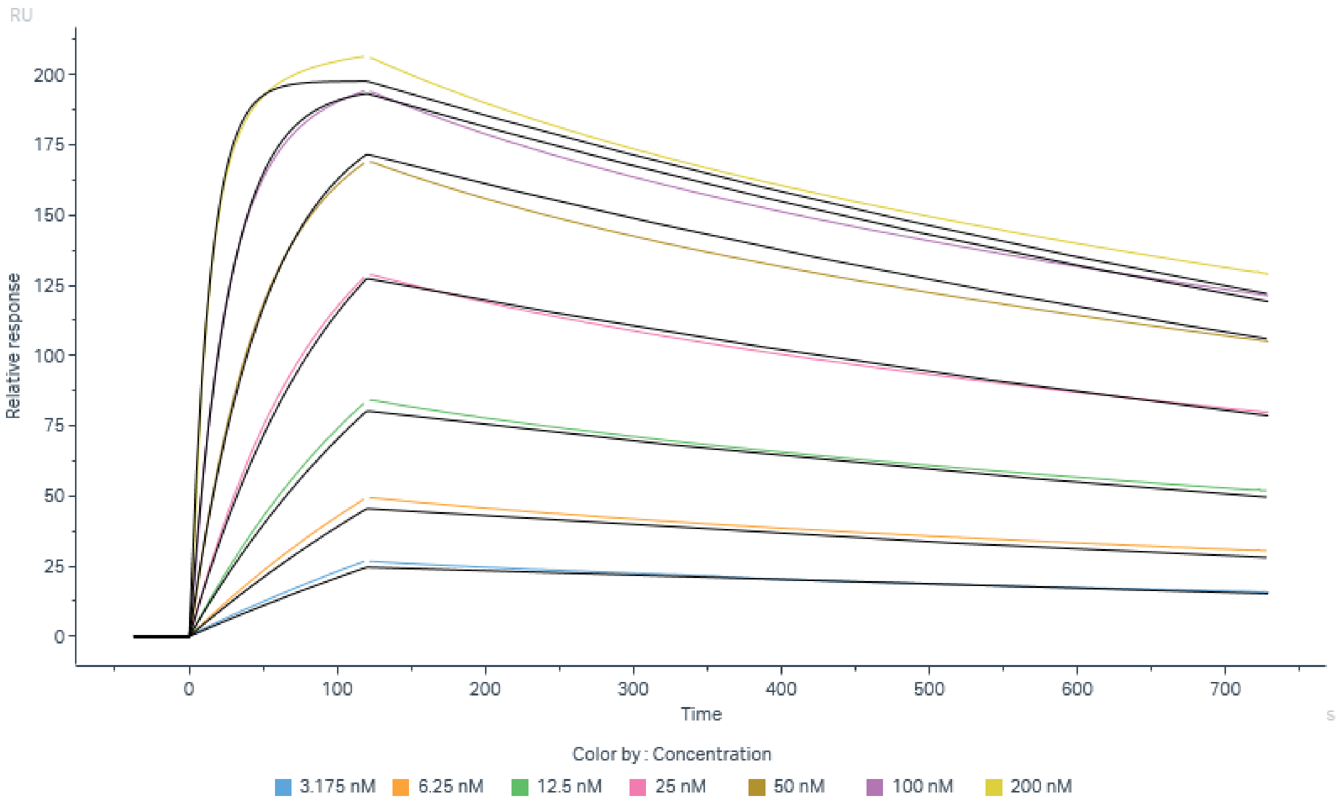


FAB 4B3; VB16T13 293F; 1:1 binding

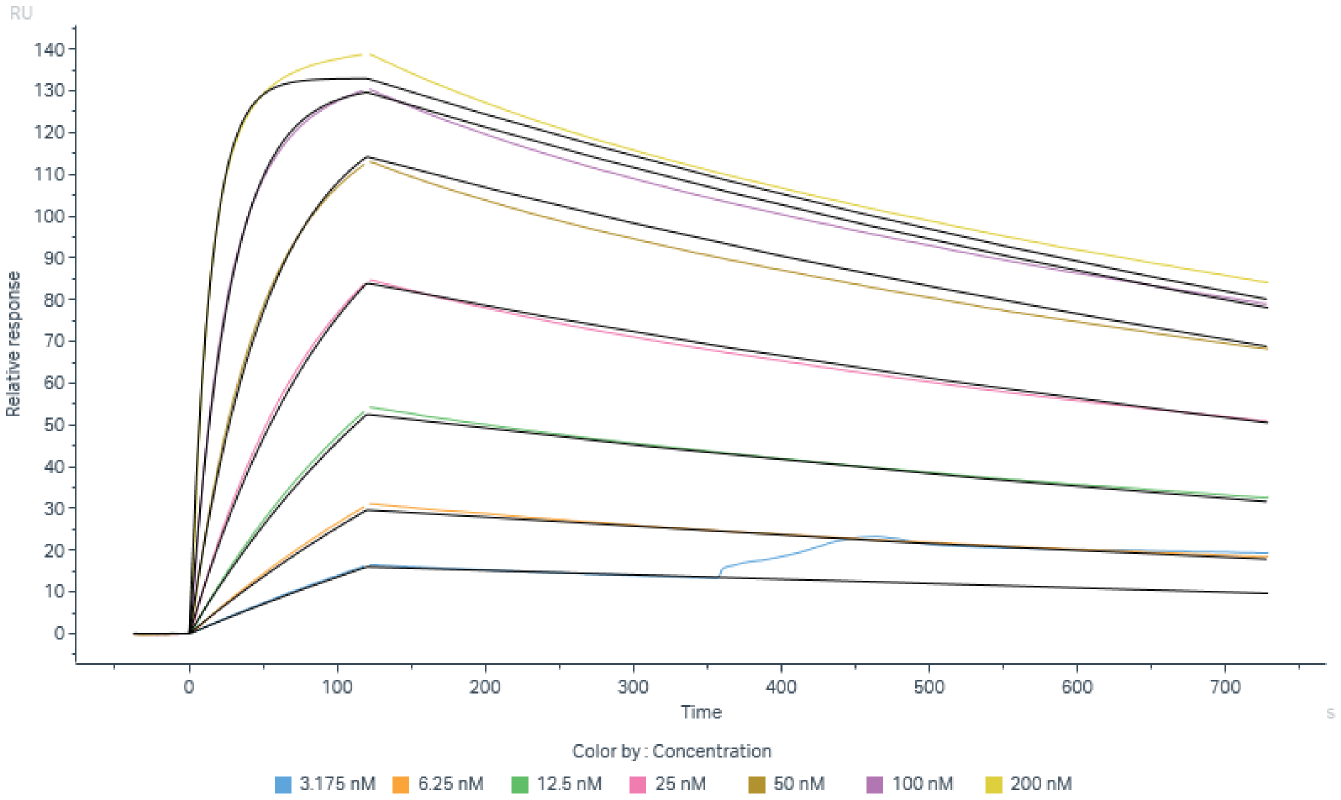

FAB 4B3; VB16T13 3T3; 1:1 binding

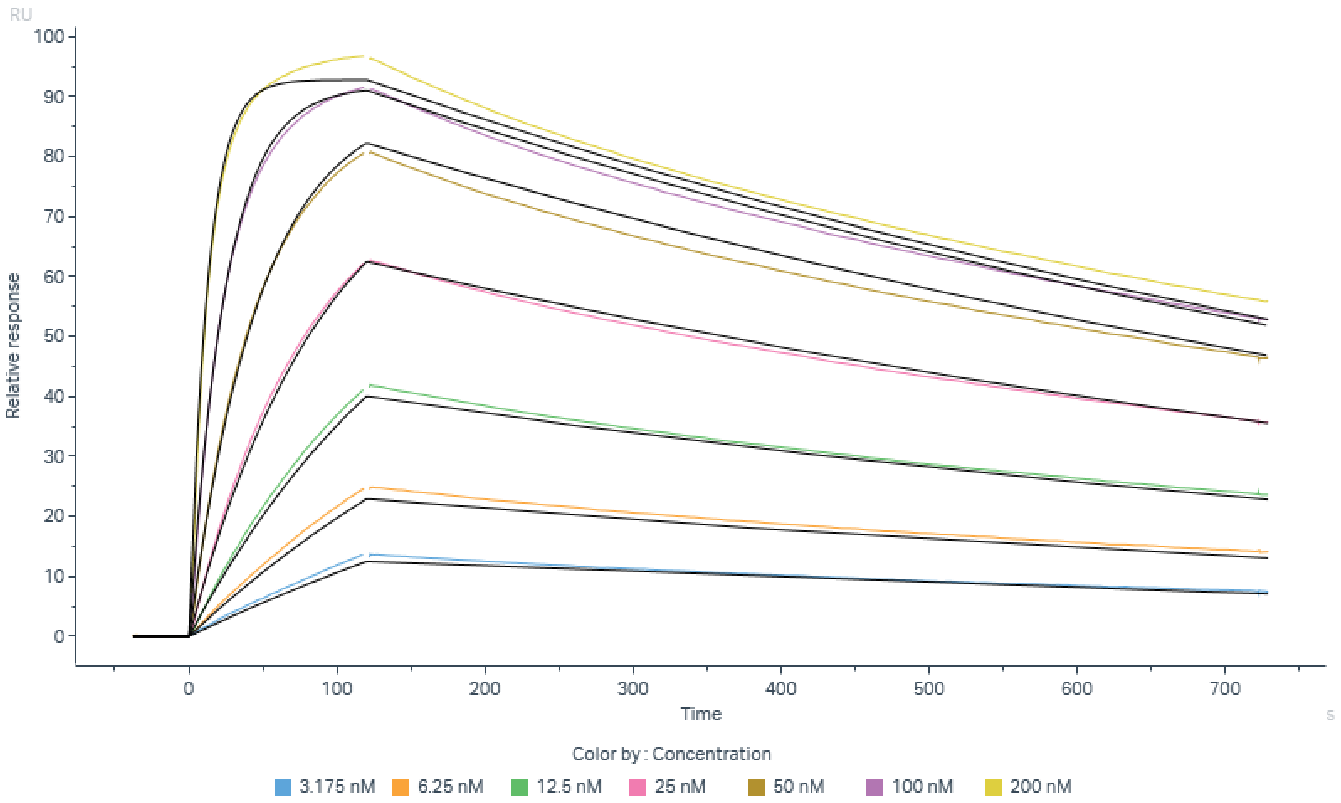


On-off rate chart

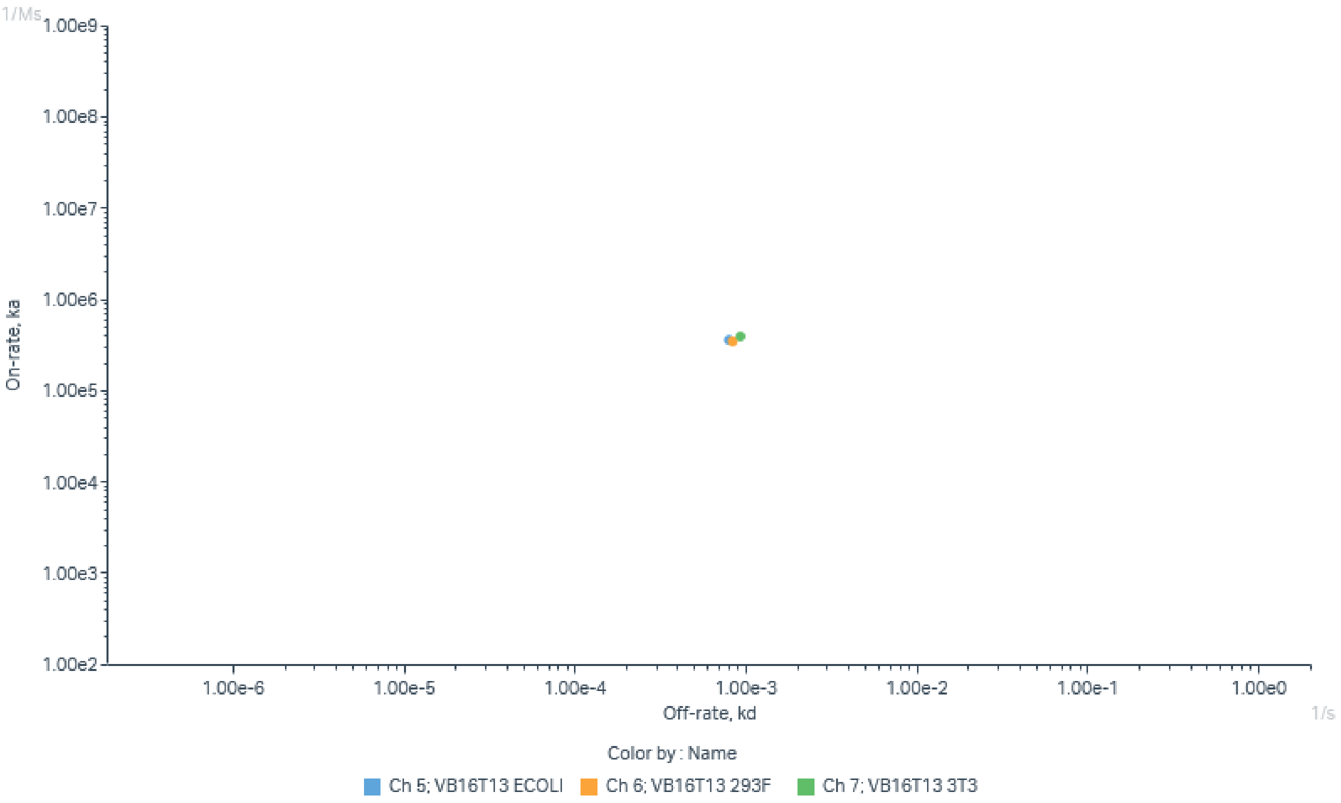

## Result table

| # | Group | General Kinetics model | Curve markers | Channel | Immobilized ligand | Injection variables Analyte 1 Solution |
|---|---|---|---|---|---|---|
| 1 | 1 | 1:1 binding | | 5 | VB16T13 ECOLI | FAB 4B3 |
| 2 | 2 | 1:1 binding | | 6 | VB16T13 293F | FAB 4B3 |
| 3 | 3 | 1:1 binding | | 7 | VB16T13 3T3 | FAB 4B3 |

| # | Quality Kinetics Chi² (RU²) | 1:1 binding ka (1/Ms) | kd (1/s) | KD (M) | Rmax (RU) | tc |
|---|---|---|---|---|---|---|
| 1 | 8.06e+00 | 3.62e+05 | 7.93e-04 | 2.19e-09 | 199.9 | 6.73e+12 |
| 2 | 8.40e+00 | 3.50e+05 | 8.34e-04 | 2.38e-09 | 134.6 | 8.98e+11 |
| 3 | 1.70e+00 | 3.97e+05 | 9.25e-04 | 2.33e-09 | 93.9 | 9.82e+11 |
