# Supplementary material for: A circular RNA vaccine induces durable and cross-protective immunity against Neisseria meningitidis serogroup B in mice
Source: PLoS Pathog. 2026 May 11;22(5):e1013741. doi: 10.1371/journal.ppat.1013741 (PMC13160355; doi:10.1371/journal.ppat.1013741)
Supplement: S1 Data — Images files. SPR PDF files. CE5200 PDF file. S1D Fig report. S1A Fig sequencing file. (ZIP) [file ppat.1013741.s016.zip › Raw data/SPR PDF files/FAB 7B10-5 VB16T13 ECOLI-6 VB16T13 293F-7 VB16T13 3t3-20260205 252026 65825 PM.pdf]

Biacore Insight Evaluation

FAB 7B10-5 VB16T13 ECOLI-6 VB16T13 293F-7 VB16T13 3t3-20260205 2/5/2026 6:58:25 PM

Evaluation

Name	FAB 7B10-5 VB16T13 ECOLI-6 VB16T13 293F-7 VB16T13 3t3-20260205 2/5/2026 6:58:25 PM
Path	Root\FAB 7B10-5 VB16T13 ECOLI-6 VB16T13 293F-7 VB16T13 3t3-20260205 2/5/2026 6:58:25 PM
Modified by	DESKTOP-22AQC8N\Dell (DESKTOP-22AQC8N\Dell)
Date modified	2/6/2026 5:22:21 PM
Created with	Biacore Insight Evaluation 4.0.8.19879
Server name	.\SQLEXPRESS
Database id	Biacore Insight Database

Run(s) in this evaluation

Run 1

Name	FAB 7B10-5 VB16T13 ECOLI-6 VB16T13 293F-7 VB16T13 3t3-20260205 2/5/2026 6:58:25 PM
Path	Root\ZW\QIQI ZHANG\FAB 7B10-5 VB16T13 ECOLI-6 VB16T13 293F-7 VB16T13 3t3-20260205 2/5/2026 6:58:25 PM
Created by	DESKTOP-22AQC8N\Dell (DESKTOP-22AQC8N\Dell)
Method	FAB 7B10-5 VB16T13 ECOLI-6 VB16T13 293F-7 VB16T13 3t3-20260205
Instrument type/ID	Biacore 8K+/2902034
Cycles	10
Data collection rate	10 Hz
Running buffer	Buffer
Start time	2/6/2026 4:46:16 AM
End time	2/6/2026 7:08:55 AM

Chip information

Chip id	10/20/2025 1:22:02 PM
Chip type	CM5
Lot number	10369187
First dock date	10/20/2025 1:23:00 PM

Run 1 - Immobilization results

Flow cell 1

	Channel 1	Channel 2	Channel 3	Channel 4
Sensorgram type	Not used in run	Not used in run	Not used in run	Not used in run
Ligand			SA	SA
MW (Da)				
Level (RU)			6142.6	6088.1
Run name			Immobilization 1/8/2026 10:43:22 AM	Immobilization 1/8/2026 10:43:22 AM
Date and time			1/8/2026 10:44:03 AM	1/8/2026 10:44:03 AM

	Channel 5	Channel 6	Channel 7	Channel 8
Sensorgram type	Reference	Reference	Reference	Not used in run
Ligand	Activation/Deactivation	Activation/Deactivation	Activation/Deactivation	
MW (Da)				
Level (RU)				
Run name	Immobilization 20260205 12:22:47 PM 2/5/2026 6:14:22 PM	Immobilization 20260205 12:22:47 PM 2/5/2026 6:14:22 PM	Immobilization 20260205 12:22:47 PM 2/5/2026 6:14:22 PM	
Date and time	2/5/2026 6:14:26 PM	2/5/2026 6:14:26 PM	2/5/2026 6:14:26 PM	

Run 1 - Immobilization results

Flow cell 2

	Channel 1	Channel 2	Channel 3	Channel 4
Sensorgram type	Not used in run	Not used in run	Not used in run	Not used in run
Ligand	Ligand 1	Ligand 2	SA	SA
MW (Da)				
Level (RU)	3483.5	5468.2	5936.3	6024.2
Run name	Immobilization 10/20/2025 1:40:59 PM	Immobilization 10/20/2025 1:40:59 PM	Immobilization 1/8/2026 10:43:22 AM	Immobilization 1/8/2026 10:43:22 AM
Date and time	10/20/2025 1:41:04 PM	10/20/2025 1:41:04 PM	1/8/2026 10:44:03 AM	1/8/2026 10:44:03 AM

	Channel 5	Channel 6	Channel 7	Channel 8
Sensorgram type	Active	Active	Active	Not used in run
Ligand	VB16T13 ECOLI	VB16T13 293F	VB16T13 3T3	
MW (Da)				
Level (RU)	288.4	256.3	323.2	
Run name	Immobilization 20260205 12:22:47 PM 2/5/2026 6:14:22 PM	Immobilization 20260205 12:22:47 PM 2/5/2026 6:14:22 PM	Immobilization 20260205 12:22:47 PM 2/5/2026 6:14:22 PM	
Date and time	2/5/2026 6:14:26 PM	2/5/2026 6:14:26 PM	2/5/2026 6:14:26 PM	

Run 1 - Method summary

2 Startup

Analyte 1

Buffer, Contact time 120 s, Diss time 60 s, Flow rate 30 µl/min, Both flow cells

Regeneration 1

1.5, Contact time 30 s, Flow rate 30 µl/min, Both flow cells

8 Analysis

Analyte 1

Variable solution, Contact time 120 s, Diss time 600 s, Flow rate 30 µl/min, Variable conc, Both flow cells

Regeneration 1

1.5, Contact time 90 s, Flow rate 30 µl/min, Both flow cells

QC - Sensorgram

Thumbnails

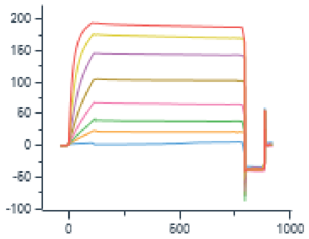

Channel 5

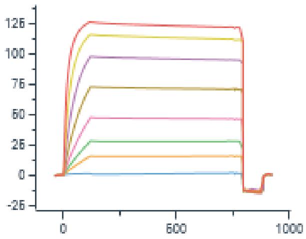

Channel 6

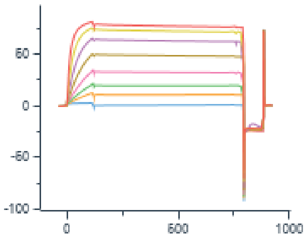

Channel 7

Charts

Channel 5

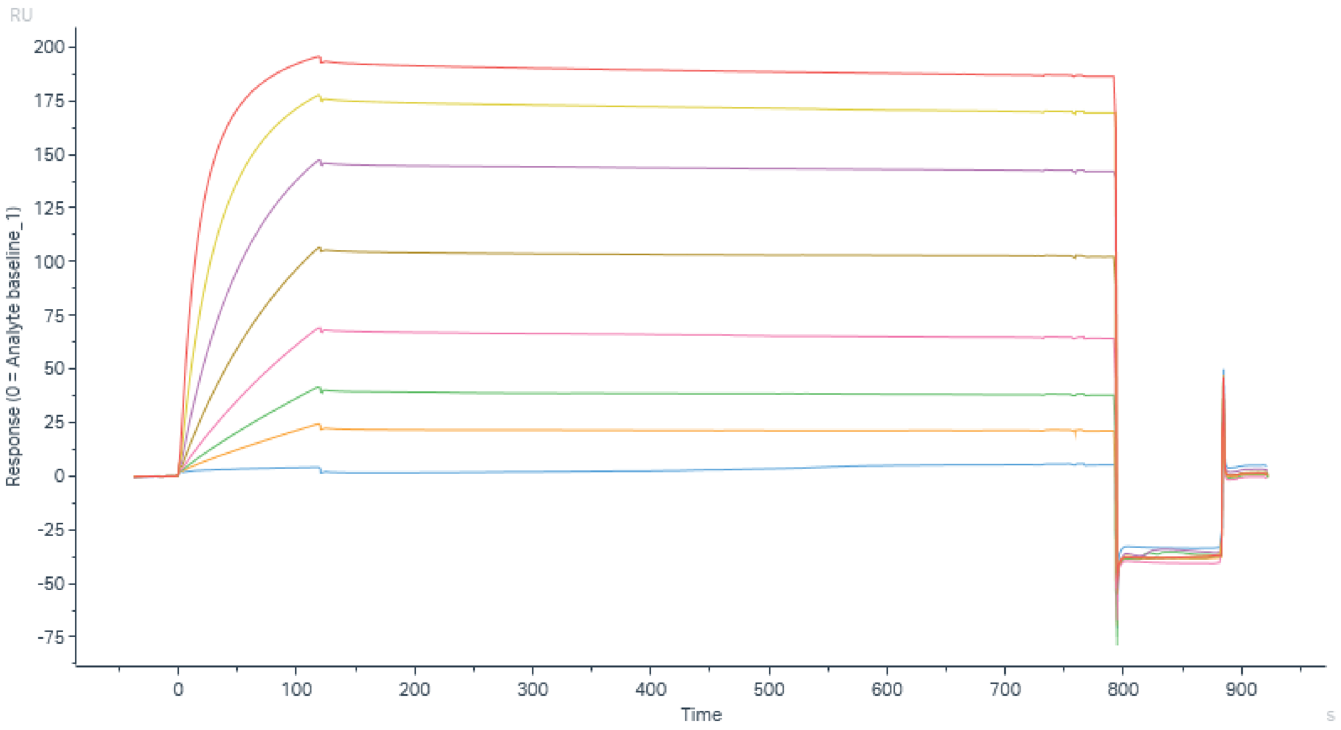

Channel 6

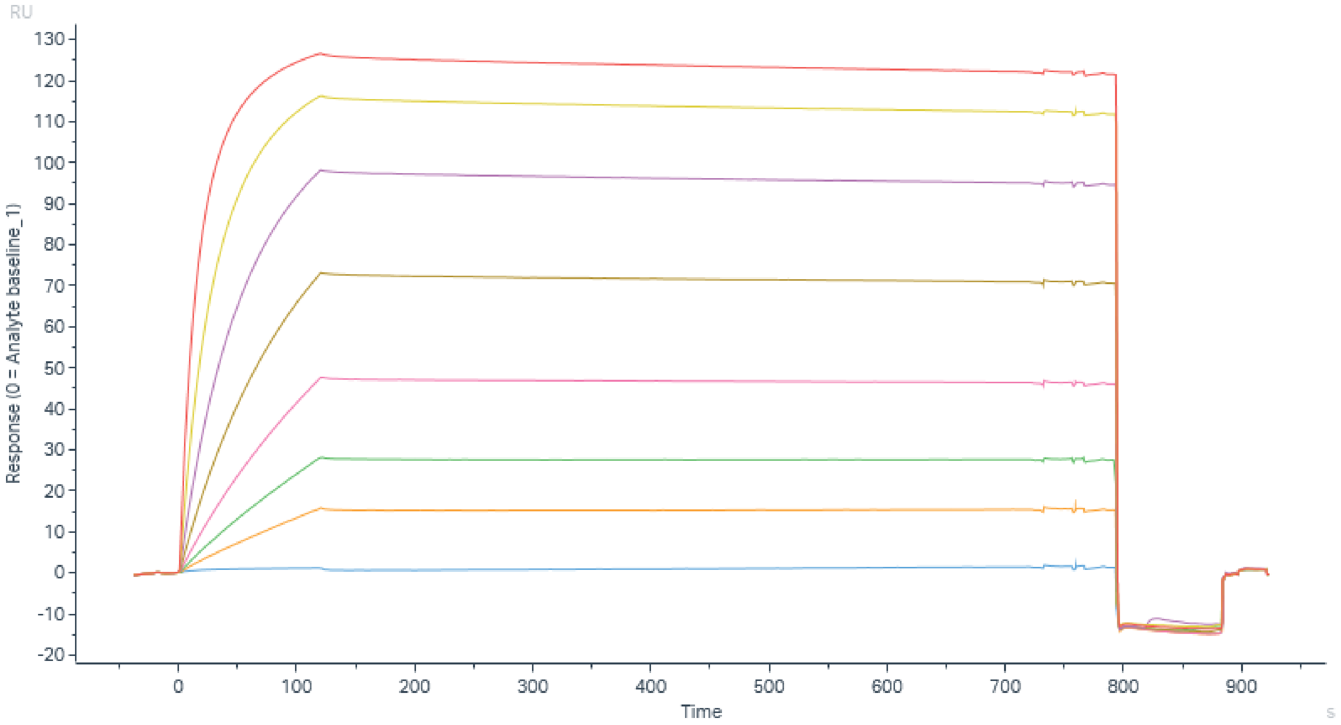

Channel 7

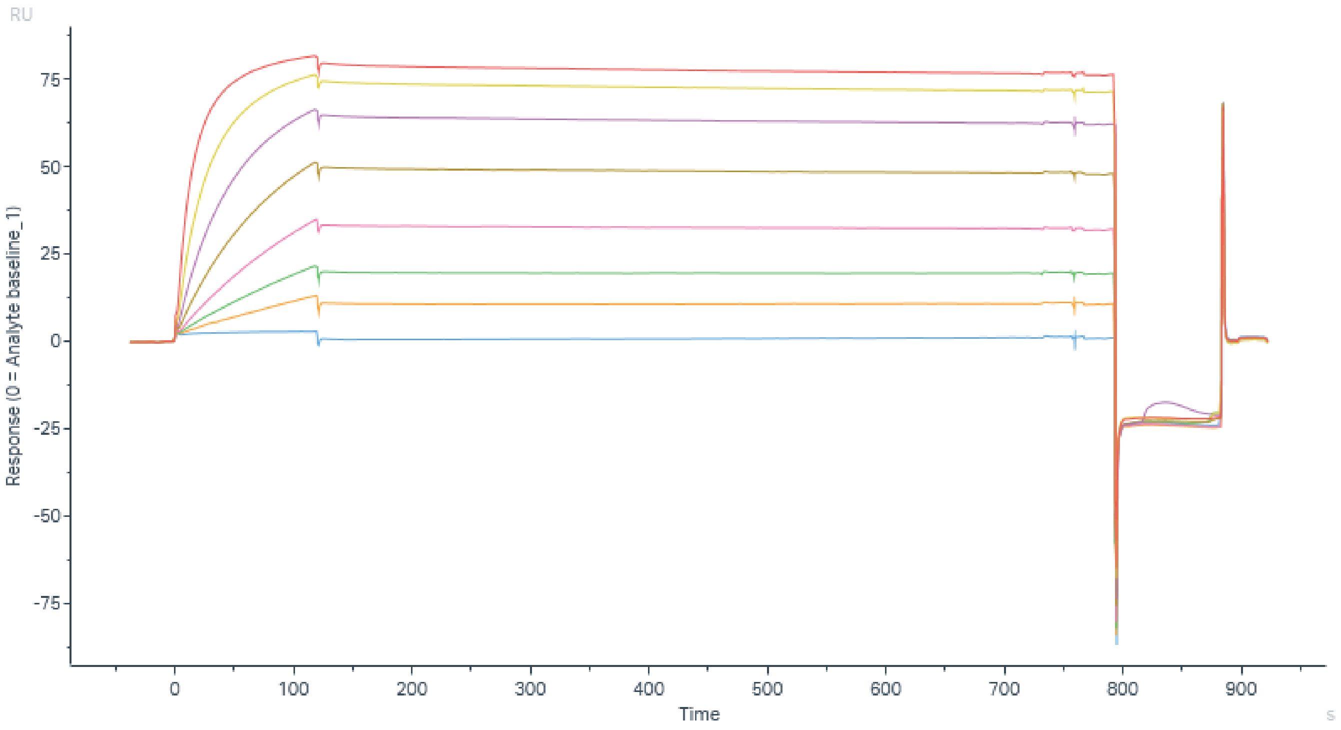
RU
75
50
25
0
-25
-50
-75
Response (0 = Analyte baseline_1)
0
100
200
300
400
500
600
700
800
900
Time
s

Sensorgram table

#	Cycle	Channel	Sensorgram type	Analysis step purpose	Analysis step name	Excluded
1	3	5	Reference subtracted	Analysis	Analysis	
2	4	5	Reference subtracted	Analysis	Analysis	
3	5	5	Reference subtracted	Analysis	Analysis	
4	6	5	Reference subtracted	Analysis	Analysis	
5	7	5	Reference subtracted	Analysis	Analysis	
6	8	5	Reference subtracted	Analysis	Analysis	
7	9	5	Reference subtracted	Analysis	Analysis	
8	10	5	Reference subtracted	Analysis	Analysis	
9	3	6	Reference subtracted	Analysis	Analysis	
10	4	6	Reference subtracted	Analysis	Analysis	
11	5	6	Reference subtracted	Analysis	Analysis	
12	6	6	Reference subtracted	Analysis	Analysis	
13	7	6	Reference subtracted	Analysis	Analysis	
14	8	6	Reference subtracted	Analysis	Analysis	
15	9	6	Reference subtracted	Analysis	Analysis	
16	10	6	Reference subtracted	Analysis	Analysis	
17	3	7	Reference subtracted	Analysis	Analysis	
18	4	7	Reference subtracted	Analysis	Analysis	
19	5	7	Reference subtracted	Analysis	Analysis	
20	6	7	Reference subtracted	Analysis	Analysis	
21	7	7	Reference subtracted	Analysis	Analysis	
22	8	7	Reference subtracted	Analysis	Analysis	
23	9	7	Reference subtracted	Analysis	Analysis	
24	10	7	Reference subtracted	Analysis	Analysis	

Sensorgram table (continued)

#	Curve markers	Analyte 1 Solution	Analyte 1 Concentration (nM)	Analyte binding late_1 Relative (RU)
1		FAB 7B10	0	4.2
2		FAB 7B10	3.175	23.9
3		FAB 7B10	6.25	40.8
4		FAB 7B10	12.5	67.8
5		FAB 7B10	25	104.9
6		FAB 7B10	50	145.8
7		FAB 7B10	100	176.7
8		FAB 7B10	200	195.1
9		FAB 7B10	0	1.2
10		FAB 7B10	3.175	15.2
11		FAB 7B10	6.25	27.2
12		FAB 7B10	12.5	46.1
13		FAB 7B10	25	71.3
14		FAB 7B10	50	96.7
15		FAB 7B10	100	115.4
16		FAB 7B10	200	126.2
17		FAB 7B10	0	2.8
18		FAB 7B10	3.175	12.8
19		FAB 7B10	6.25	21.2
20		FAB 7B10	12.5	34.2
21		FAB 7B10	25	50.6
22		FAB 7B10	50	65.9
23		FAB 7B10	100	76.1
24		FAB 7B10	200	81.6

QC - Baseline

Thumbnails

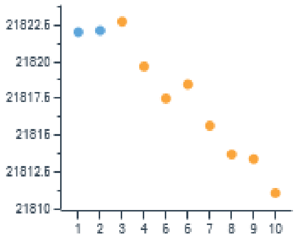

Channel 5

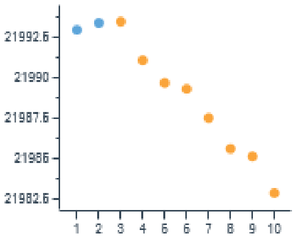

Channel 6

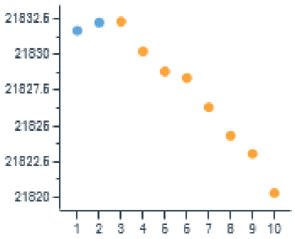

Channel 7

Charts

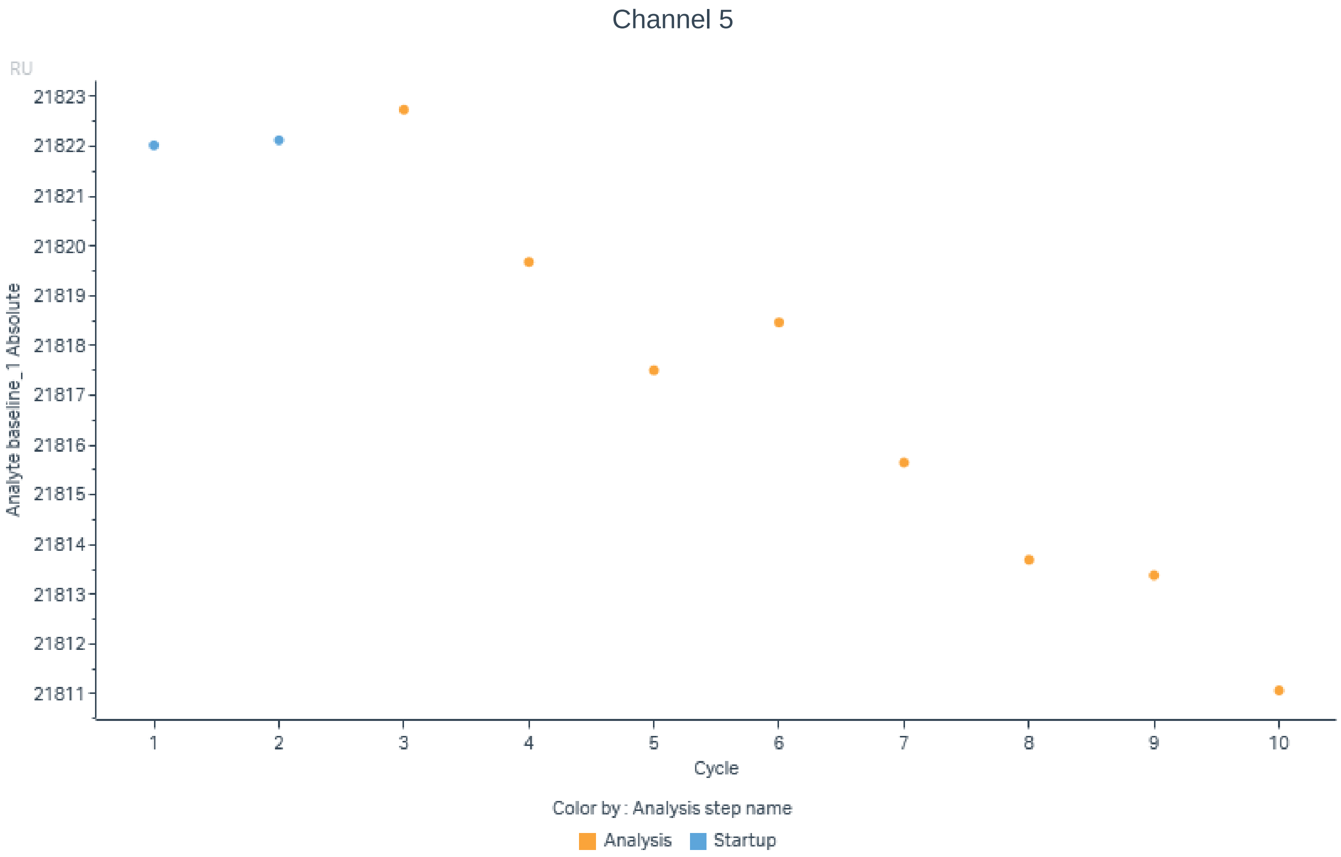
Channel 5
RU
21823
21822
21821
21820
21819
21818
21817
21816
21815
21814
21813
21812
21811
Analyte baseline_1 Absolute
1
2
3
4
5
6
7
8
9
10
Cycle
Color by : Analysis step name
Analysis
Startup

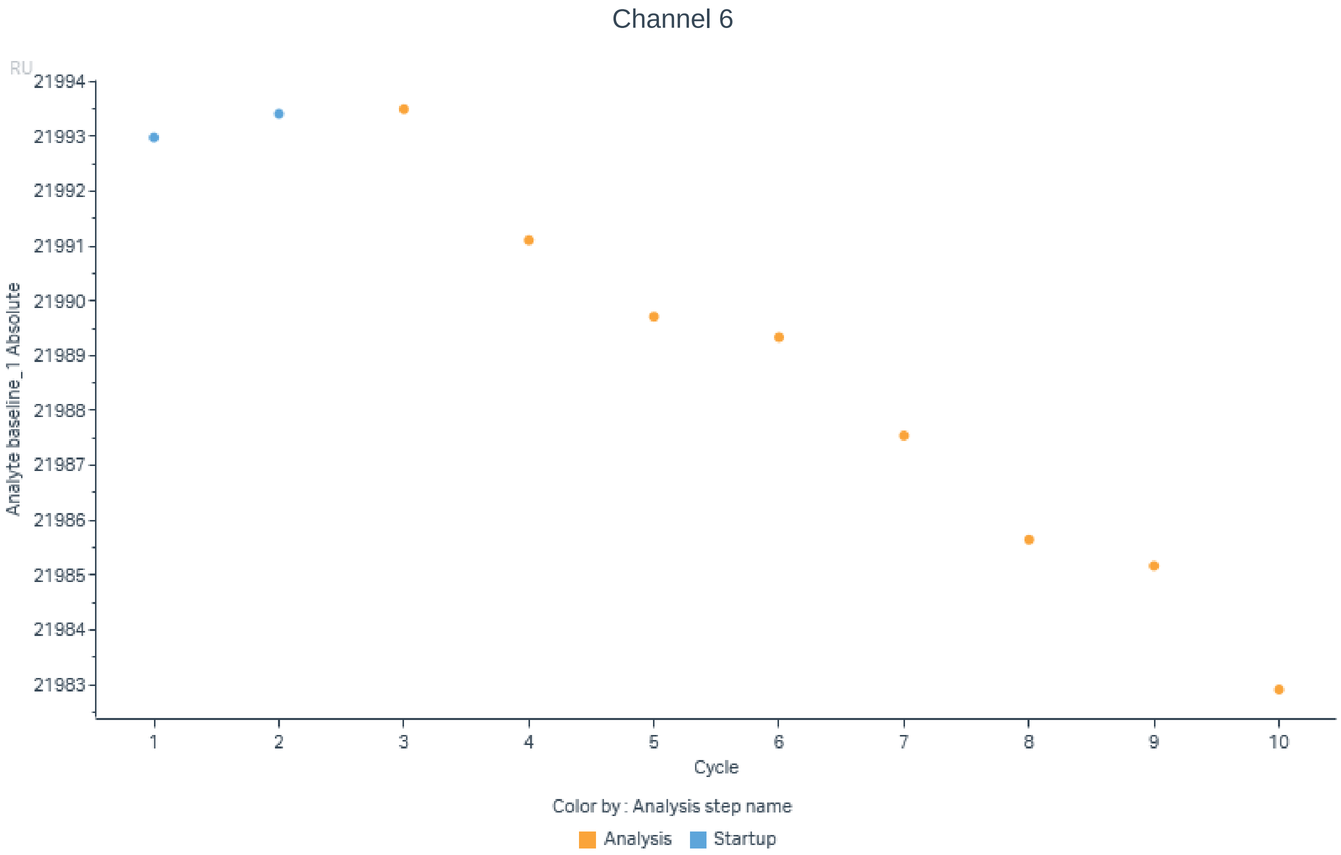
Channel 6
RU
21994
21993
21992
21991
21990
21989
21988
21987
21986
21985
21984
21983
Analyte baseline_1 Absolute
1
2
3
4
5
6
7
8
9
10
Cycle
Color by : Analysis step name
Analysis
Startup

Channel 7

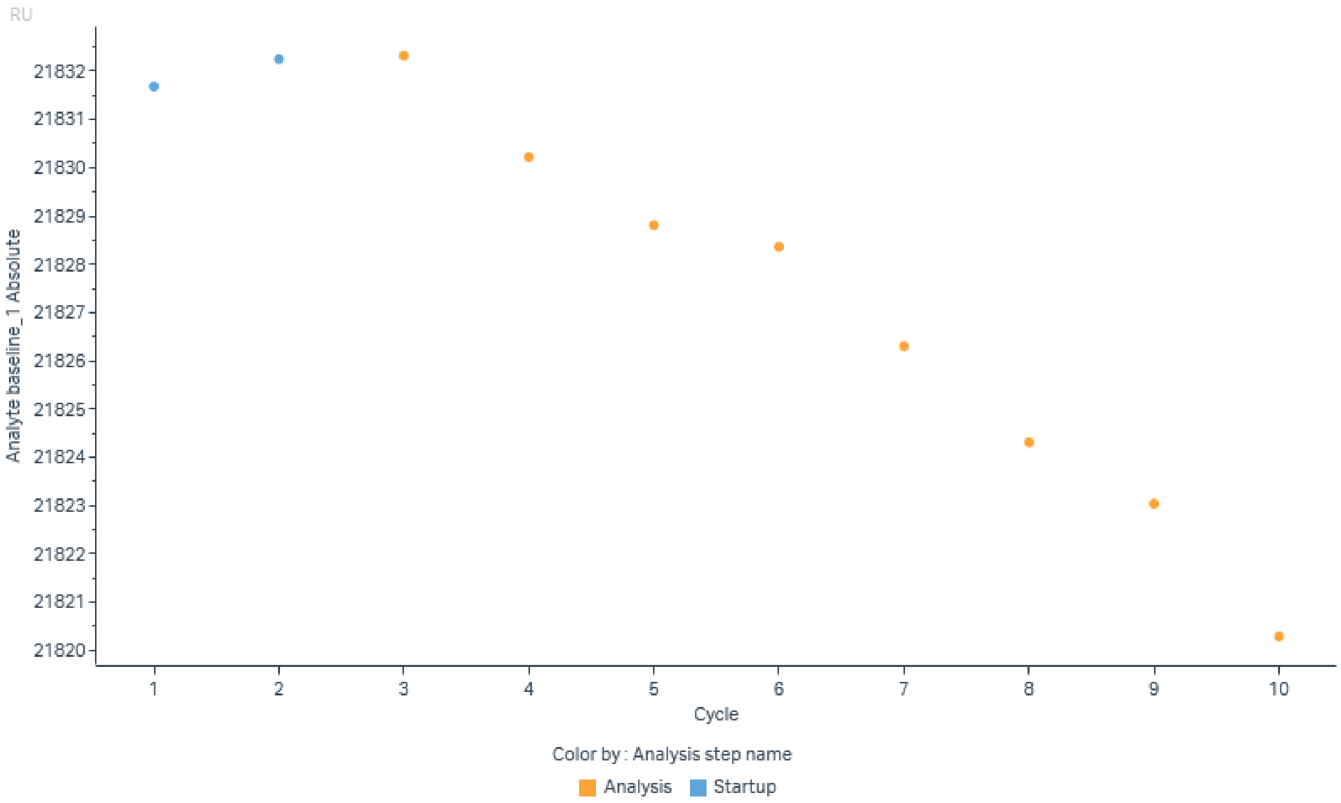
RU
Analyte baseline_1 Absolute
21832
21831
21830
21829
21828
21827
21826
21825
21824
21823
21822
21821
21820
1
2
3
4
5
6
7
8
9
10
Cycle
Color by : Analysis step name
Analysis
Startup

Plot table

#	Cycle	Channel	Sensorgram type	Analysis step purpose	Analysis step name	Excluded
1	1	5	Active	Startup	Startup	
2	2	5	Active	Startup	Startup	
3	3	5	Active	Analysis	Analysis	
4	4	5	Active	Analysis	Analysis	
5	5	5	Active	Analysis	Analysis	
6	6	5	Active	Analysis	Analysis	
7	7	5	Active	Analysis	Analysis	
8	8	5	Active	Analysis	Analysis	
9	9	5	Active	Analysis	Analysis	
10	10	5	Active	Analysis	Analysis	
11	1	6	Active	Startup	Startup	
12	2	6	Active	Startup	Startup	
13	3	6	Active	Analysis	Analysis	
14	4	6	Active	Analysis	Analysis	
15	5	6	Active	Analysis	Analysis	
16	6	6	Active	Analysis	Analysis	
17	7	6	Active	Analysis	Analysis	
18	8	6	Active	Analysis	Analysis	
19	9	6	Active	Analysis	Analysis	
20	10	6	Active	Analysis	Analysis	
21	1	7	Active	Startup	Startup	
22	2	7	Active	Startup	Startup	
23	3	7	Active	Analysis	Analysis	
24	4	7	Active	Analysis	Analysis	
25	5	7	Active	Analysis	Analysis	
26	6	7	Active	Analysis	Analysis	
27	7	7	Active	Analysis	Analysis	
28	8	7	Active	Analysis	Analysis	
29	9	7	Active	Analysis	Analysis	
30	10	7	Active	Analysis	Analysis	

Plot table (continued)

#	Curve markers	Analyte 1 Solution	Analyte baseline_1 Absolute (RU)
1		Buffer	21822.0
2		Buffer	21822.1
3		FAB 7B10	21822.7
4		FAB 7B10	21819.7
5		FAB 7B10	21817.5
6		FAB 7B10	21818.5
7		FAB 7B10	21815.6
8		FAB 7B10	21813.7
9		FAB 7B10	21813.4
10		FAB 7B10	21811.1
11		Buffer	21993.0
12		Buffer	21993.4
13		FAB 7B10	21993.5
14		FAB 7B10	21991.1
15		FAB 7B10	21989.7
16		FAB 7B10	21989.3
17		FAB 7B10	21987.5
18		FAB 7B10	21985.6
19		FAB 7B10	21985.2
20		FAB 7B10	21982.9
21		Buffer	21831.7
22		Buffer	21832.3
23		FAB 7B10	21832.3
24		FAB 7B10	21830.2
25		FAB 7B10	21828.8
26		FAB 7B10	21828.4
27		FAB 7B10	21826.3
28		FAB 7B10	21824.3
29		FAB 7B10	21823.0
30		FAB 7B10	21820.3

QC - Binding to reference

Thumbnails

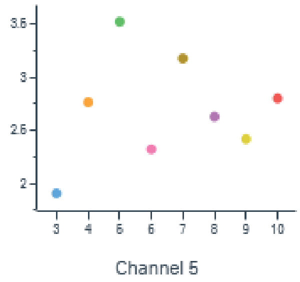

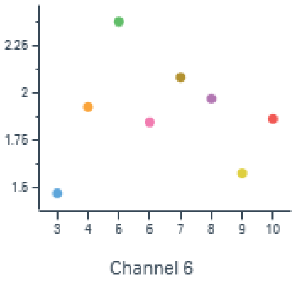

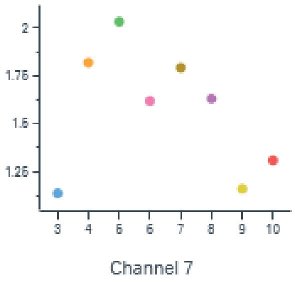

Charts

Channel 5

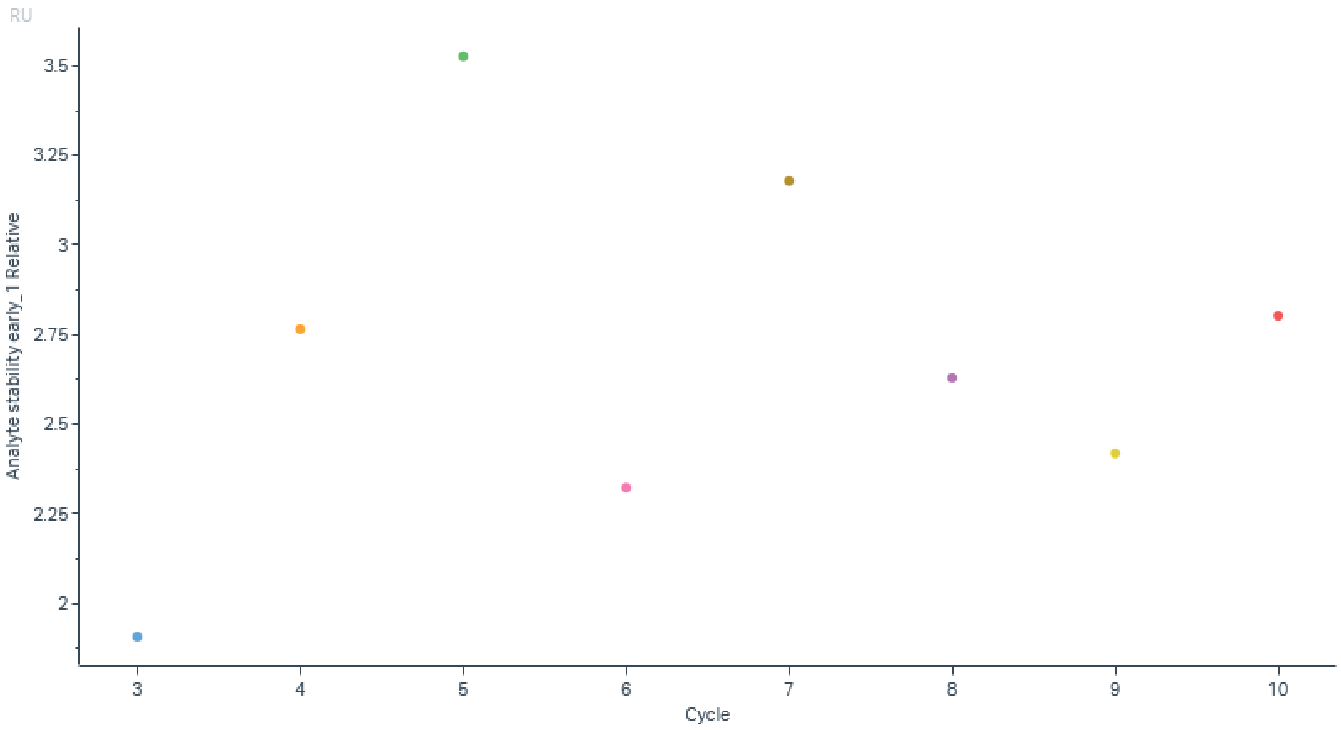

Channel 6

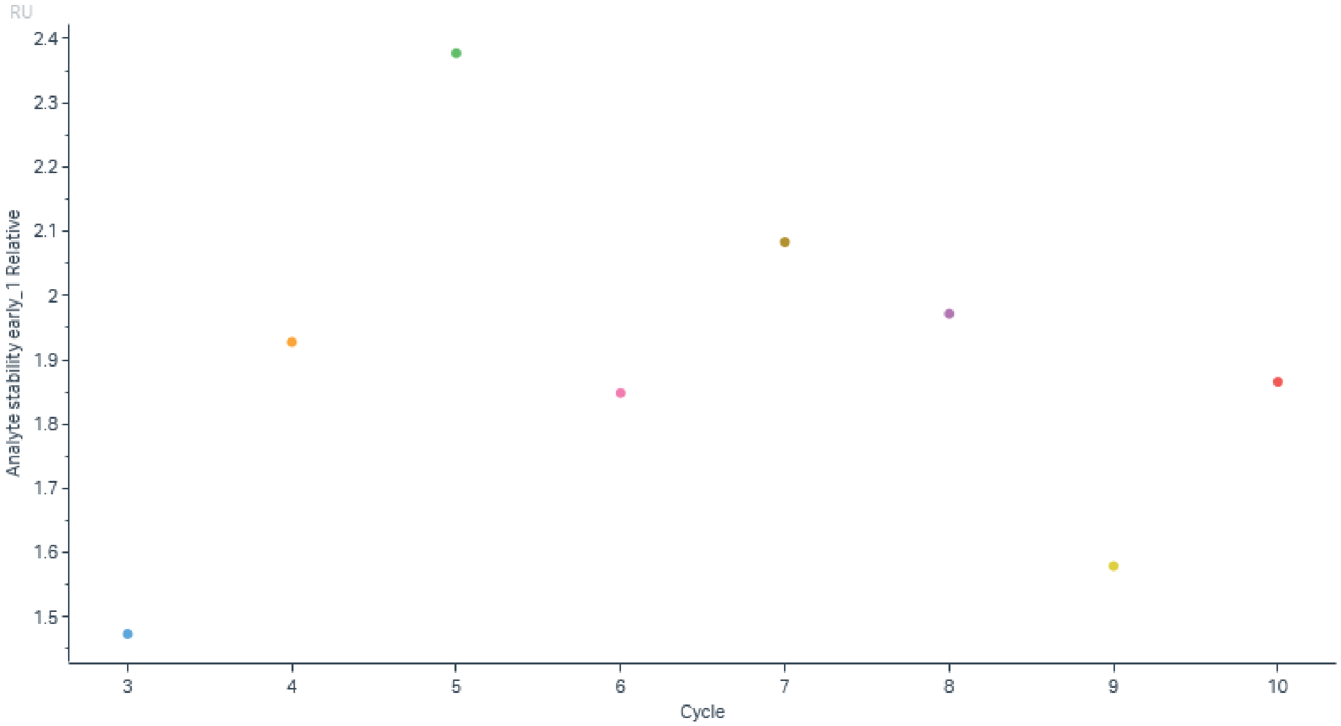

Channel 7

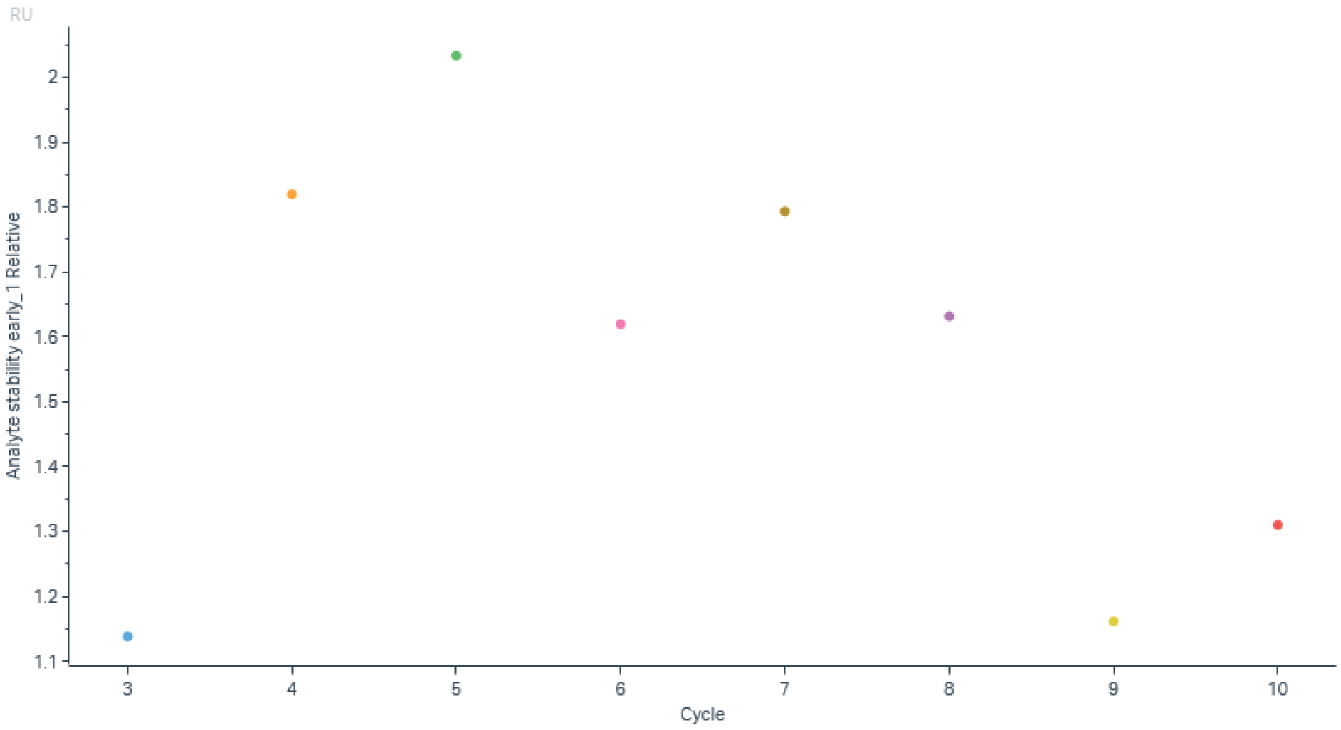
RU
Analyte stability early_1 Relative
2
1.9
1.8
1.7
1.6
1.5
1.4
1.3
1.2
1.1
3
4
5
6
7
8
9
10
Cycle

Plot table

#	Cycle	Channel	Sensorgram type	Analysis step purpose	Analysis step name	Excluded
1	3	5	Reference	Analysis	Analysis	
2	4	5	Reference	Analysis	Analysis	
3	5	5	Reference	Analysis	Analysis	
4	6	5	Reference	Analysis	Analysis	
5	7	5	Reference	Analysis	Analysis	
6	8	5	Reference	Analysis	Analysis	
7	9	5	Reference	Analysis	Analysis	
8	10	5	Reference	Analysis	Analysis	
9	3	6	Reference	Analysis	Analysis	
10	4	6	Reference	Analysis	Analysis	
11	5	6	Reference	Analysis	Analysis	
12	6	6	Reference	Analysis	Analysis	
13	7	6	Reference	Analysis	Analysis	
14	8	6	Reference	Analysis	Analysis	
15	9	6	Reference	Analysis	Analysis	
16	10	6	Reference	Analysis	Analysis	
17	3	7	Reference	Analysis	Analysis	
18	4	7	Reference	Analysis	Analysis	
19	5	7	Reference	Analysis	Analysis	
20	6	7	Reference	Analysis	Analysis	
21	7	7	Reference	Analysis	Analysis	
22	8	7	Reference	Analysis	Analysis	
23	9	7	Reference	Analysis	Analysis	
24	10	7	Reference	Analysis	Analysis	

Plot table (continued)

#	Curve markers	Analyte 1 Solution	Analyte stability early_1 Relative (RU)
1		FAB 7B10	1.9
2		FAB 7B10	2.8
3		FAB 7B10	3.5
4		FAB 7B10	2.3
5		FAB 7B10	3.2
6		FAB 7B10	2.6
7		FAB 7B10	2.4
8		FAB 7B10	2.8
9		FAB 7B10	1.5
10		FAB 7B10	1.9
11		FAB 7B10	2.4
12		FAB 7B10	1.8
13		FAB 7B10	2.1
14		FAB 7B10	2.0
15		FAB 7B10	1.6
16		FAB 7B10	1.9
17		FAB 7B10	1.1
18		FAB 7B10	1.8
19		FAB 7B10	2.0
20		FAB 7B10	1.6
21		FAB 7B10	1.8
22		FAB 7B10	1.6
23		FAB 7B10	1.2
24		FAB 7B10	1.3

Evaluation - Kinetics

Thumbnails

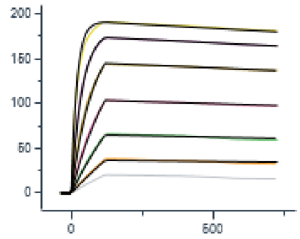

FAB 7B10; VB16T13 ECOLI; 1:1 binding

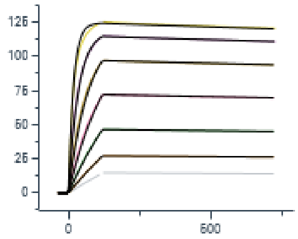

FAB 7B10; VB16T13 293F; 1:1 binding

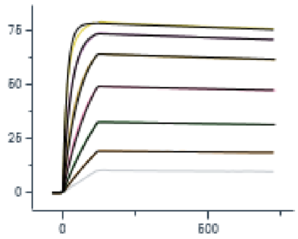

FAB 7B10; VB16T13 3T3; 1:1 binding

Charts

FAB 7B10; VB16T13 ECOLI; 1:1 binding

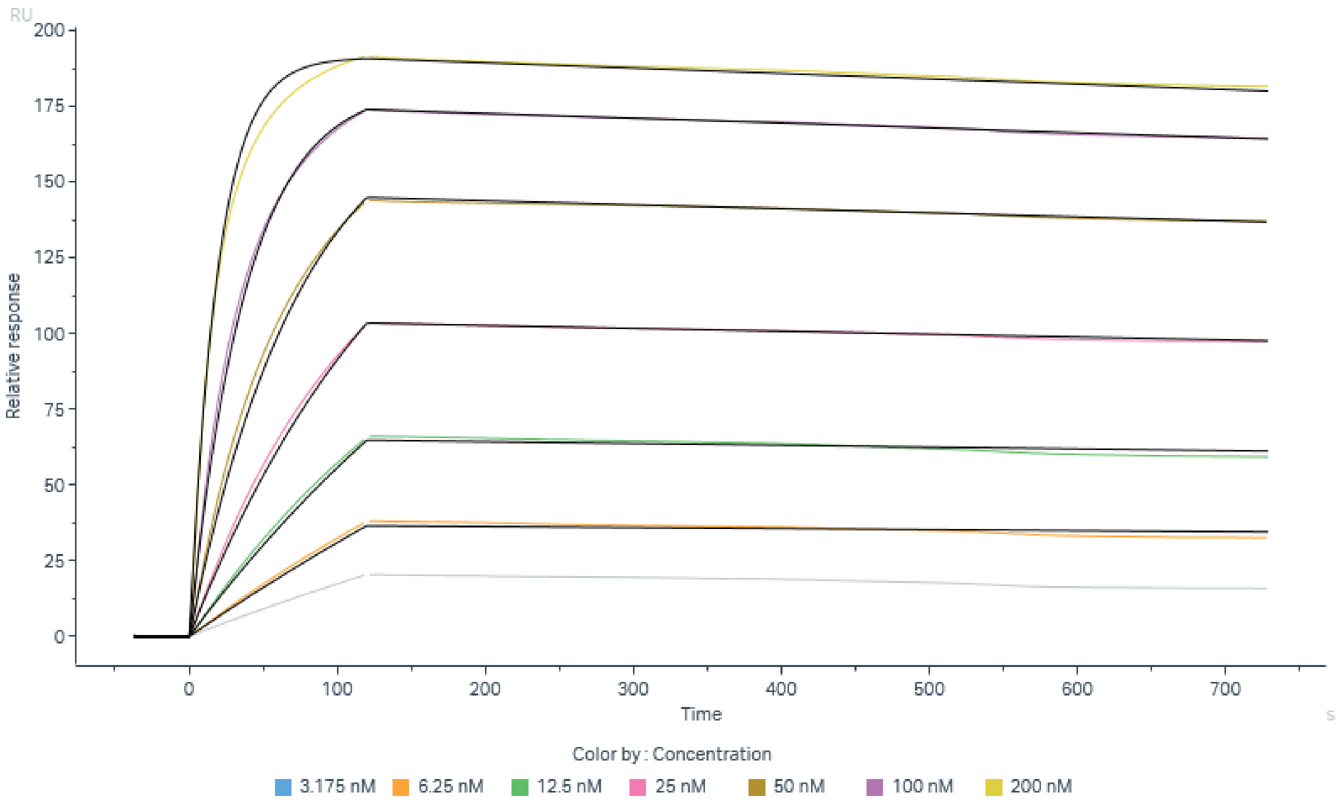

FAB 7B10; VB16T13 293F; 1:1 binding

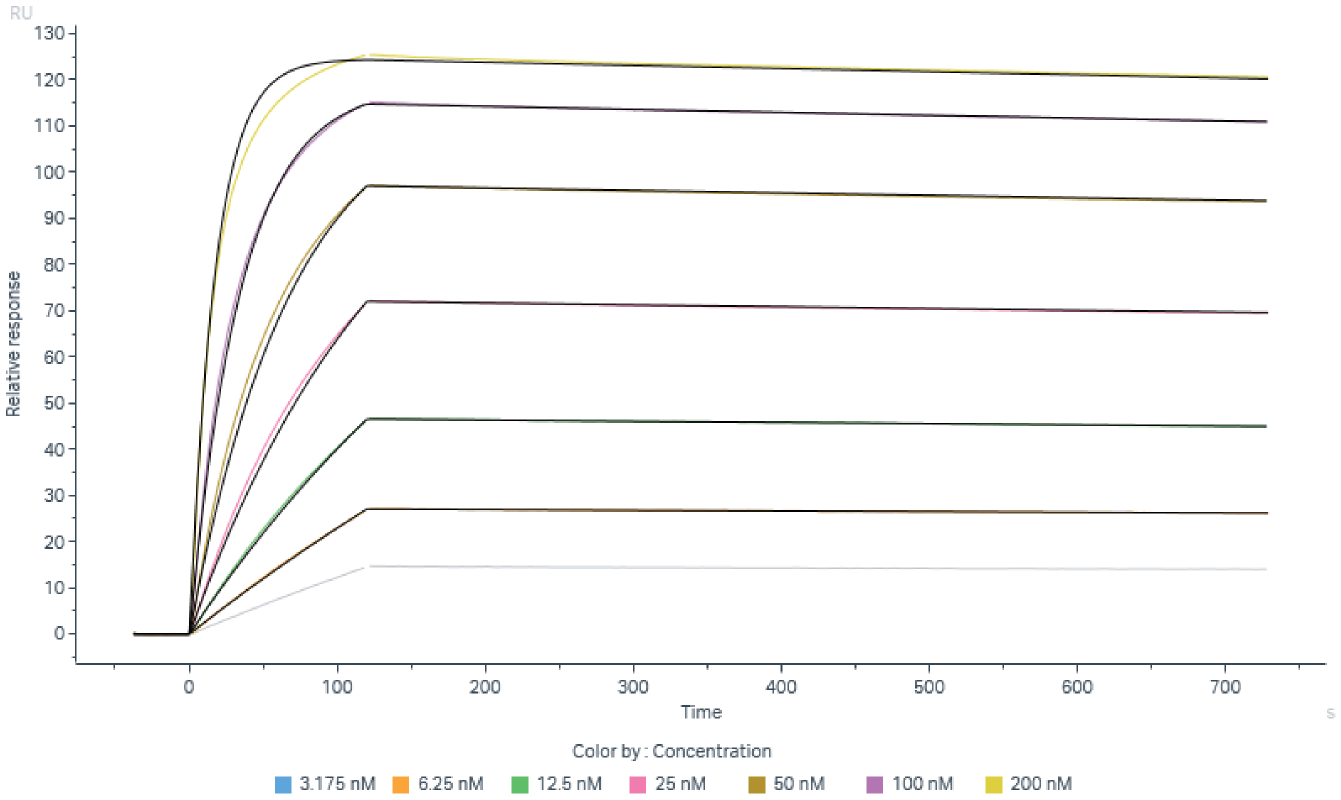

FAB 7B10; VB16T13 3T3; 1:1 binding

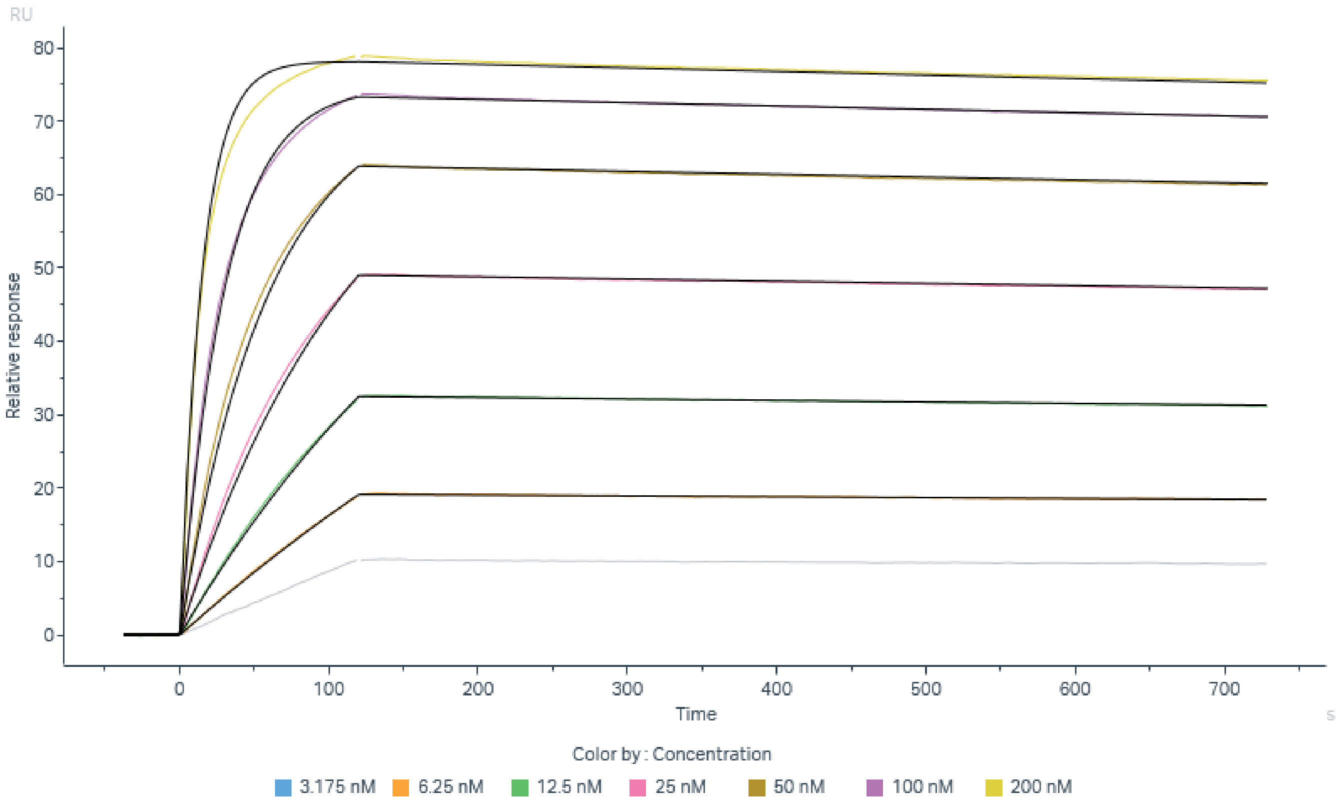

On-off rate chart

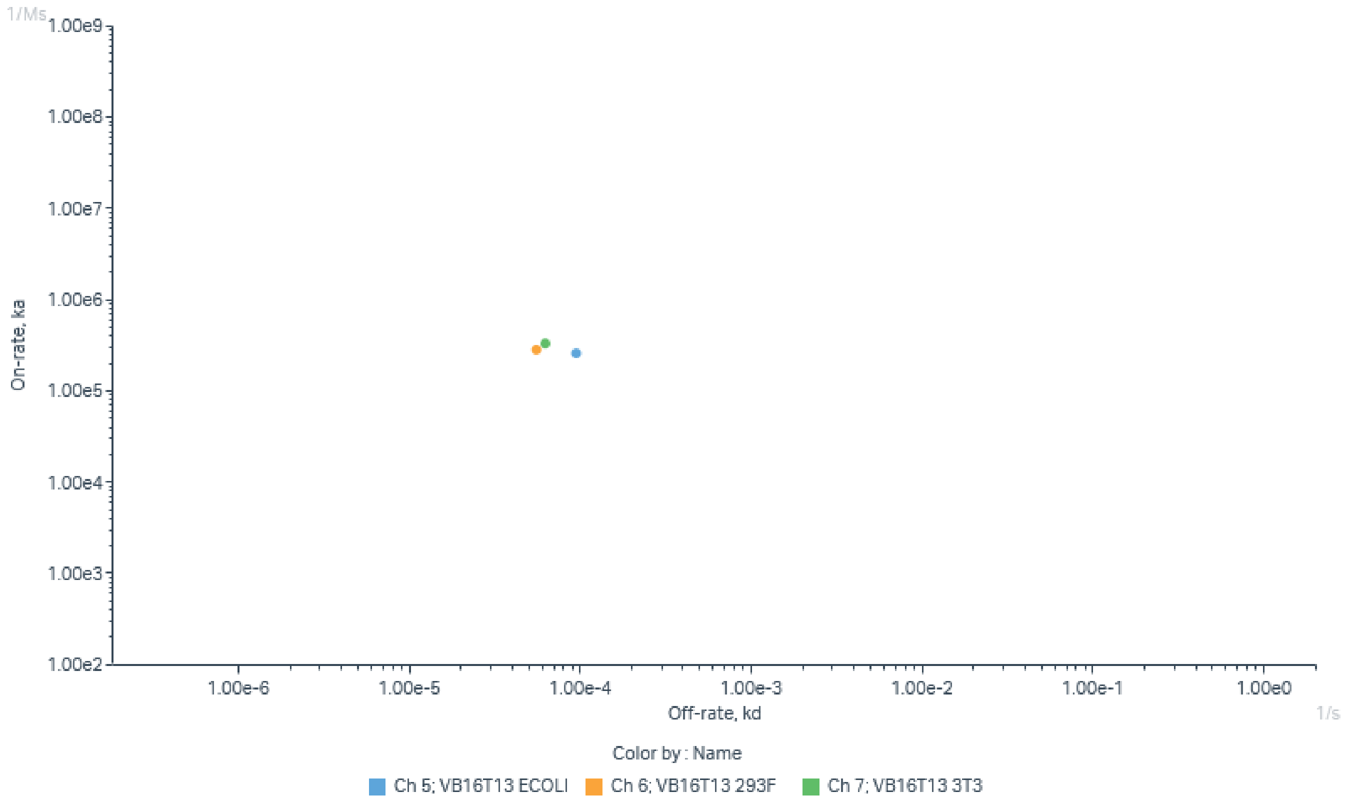

Result table

#	Group	General Kinetics model	Curve markers	Channel	Immobilized ligand	Injection variables Analyte 1 Solution
1	1	1:1 binding		5	VB16T13 ECOLI	FAB 7B10
2	2	1:1 binding		6	VB16T13 293F	FAB 7B10
3	3	1:1 binding		7	VB16T13 3T3	FAB 7B10

#	Quality Kinetics Chi² (RU²)	1:1 binding ka (1/Ms)	kd (1/s)	KD (M)	Rmax (RU)	tc
1	2.12e+00	2.59e+05	9.48e-05	3.66e-10		1.67e+12
2	7.46e-01	2.84e+05	5.53e-05	1.95e-10		3.02e+14
3	3.07e-01	3.32e+05	6.23e-05	1.88e-10		4.57e+10
